# Crystal structures of sixteen phosphane chalcogenide complexes of gold(I) chloride, bromide and iodide^1^

**DOI:** 10.1107/S2056989023010459

**Published:** 2024-01-01

**Authors:** Daniel Upmann, Peter G. Jones, Dirk Bockfeld, Eliza Târcoveanu

**Affiliations:** a Institut für Anorganische und Analytische Chemie, Technische Universität Braunschweig, Hagenring 30, D-38106 Braunschweig, Germany; Universität Greifswald, Germany

**Keywords:** crystal structure, gold(I) halides, phosphane chalcogenides, secondary inter­actions

## Abstract

The structures of 16 phosphane chalcogenide complexes of gold(I) halides, with the general formula *R* ^1^_3-_ *_n_R* ^2^_*n*_P*E*Au*X* (*R* ^1^ = *t*-butyl; *R* ^2^ = isopropyl; *n* = 0 to 3; *E* = S or Se; *X* = Cl, Br or I), are presented. The eight possible chlorido derivatives are: **1a**, *n* = 3, *E* = S; **2a**, *n* = 2, *E* = S; **3a**, *n* = 1, *E* = S; **4a**, *n* = 0, *E* = S; **5a**, *n* = 3, *E* = Se; **6a**, *n* = 2, *E* = Se; **7a**, *n* = 1, *E* = Se; and **8a**, *n* = 0, *E* = Se, and the corresponding bromido derivatives are **1b**–**8b** in the same order. However, **2a** and **2b** were badly disordered and **8a** was not obtained. The iodido derivatives are **2c**, **6c** and **7c** (numbered as for the series **a** and **b**). All structures are solvent-free and all have *Z*′ = 1 except for **6b** and **6c** (*Z*′ = 2). All mol­ecules show the expected linear geometry at gold and approximately tetra­hedral angles P—*E*—Au. The presence of bulky ligands forces some short intra­molecular contacts, in particular H⋯Au and H⋯*E*. The Au—*E* bond lengths have a slight but consistent tendency to be longer when *trans* to a softer *X* ligand, and *vice versa*. The five compounds **1a**, **5a**, **6a**, **1b** and **5b** form an isotypic set, despite the different alkyl groups in **6a**. Compounds **3a**/**3b**, **4b**/**8b** and **6b**/**6c** form isotypic pairs. The crystal packing can be analysed in terms of various types of secondary inter­actions, of which the most frequent are ‘weak’ hydrogen bonds from methine hydrogen atoms to the halogenido ligands. For the structure type **1a**, H⋯*X* and H⋯*E* contacts combine to form a layer structure. For **3a**/**3b**, the packing is almost featureless, but can be described in terms of a double-layer structure involving borderline H⋯Cl/Br and H⋯S contacts. In **4a** and **4b**/**8b**, which lack methine groups, C_meth­yl_—H⋯*X* contacts combine to form layer structures. In **7a**/**7b**, short C—H⋯*X* inter­actions form chains of mol­ecules that are further linked by association of short Au⋯Se contacts to form a layer structure. The packing of compound **6b/6c** can conveniently be analysed for each independent mol­ecule separately, because they occupy different regions of the cell. Mol­ecule 1 forms chains in which the mol­ecules are linked by a C_methine_⋯Au contact. The mol­ecules 2 associate *via* a short Se⋯Se contact and a short H⋯*X* contact to form a layer structure. The packing of compound **2c** can be described in terms of two short C_methine_—H⋯I contacts, which combine to form a corrugated ribbon structure. Compound **7c** is the only compound in this paper to feature Au⋯Au contacts, which lead to twofold-symmetric dimers. Apart from this, the packing is almost featureless, consisting of layers with only translation symmetry except for two very borderline Au⋯H contacts.

## Abstract 2

The structures of sixteen phosphane chalcogenide complexes of gold(I) halides are presented and compared.

## 1. Chemical context

Phosphane chalcogenides of the general formula *R*_3_P=*E*, where the *R* groups are alkyl or aryl (and may also be mixed) and *E* represents the chalcogenide, are well-known compounds that can act as ligands *via* the atom *E*. Diphos­phane mono- and dichalcogenides are also well-known, especially those of bis­(di­phenyl­phosphano)methane (‘dppm’) and bis­(di­phenyl­phosphano)ethane (‘dppe’). We note at the outset that the formulae of phosphane chalcogenides are traditionally written with a double bond P=*E* (as in this publication), but the concept of double bonds between elements of the third and higher periods has been the subject of much debate (see *e.g.* Schmøkel *et al.*, 2012 ▸), suggesting that the formulation P=*E*, though convenient, might be too simplistic (using a ‘resonance’ model, the alternative form *R*_3_P^+^—*E* ^−^ would also need to be considered).

Phosphane chalcogenides can form adducts with simple mol­ecules, in particular dihalogen mol­ecules X_2_, as seen in the pioneering work of du Mont and others, who showed that some adducts simply involved the atom sequence P—*E*—*X*—*X*, whereas others involved the formation of cations such as (iodo­seleno)­phospho­nium (*R*_3_PSeI)^+^ (*e.g.* Seppälä *et al.*, 1999 ▸; Jeske *et al.*, 1999 ▸; Hrib *et al.*, 2006 ▸; du Mont *et al.*, 2008 ▸).

According to the ‘hard/soft’ classification of Pearson (1963 ▸), phosphane oxides are hard ligands, whereas the sulfides, selenides and tellurides are soft. One would therefore expect that a soft metal such as gold [which corresponds to our major research inter­est, see *e.g.* Döring & Jones (2023 ▸)] would preferentially form complexes with phosphane sulfides, selen­ides or tellurides. Indeed, phosphane oxides form very few gold complexes; the only ‘simple’ compound of this type for which a structure has been determined is tris­(tri­fluoro­meth­yl)(tri­phenyl­phosphane oxide)gold(III), involving highly electron-withdrawing ligands (Pérez-Bítrian *et al.*, 2017 ▸). Nevertheless, there are two established examples of P=O groups coordinating to Au^I^; in a chelating carbene complex (Martinez *et al.*, 2021 ▸) and in a complex of a (*C*,*C′*,*O*)-chelating ligand *o*-(C_6_F_4_)P(=O)Ph(C_6_H_4_) (Bennett *et al.*, 2009 ▸). Phosphane tellurides, on the other hand, are difficult to handle because of their limited stability, but some complexes have been structurally established, notably with silver (Daniliuc *et al.*, 2007 ▸).

We have published an extensive series of structures involving phosphane sulfides and selenides, whether as compounds in their own right (*e.g.* diphosphane monochalcogenides; Taouss & Jones, 2013 ▸), as adducts with dihalogens (*e.g.* dppmSe_2_·2I_2_; Upmann & Jones, 2018 ▸), or as ligands (*e.g.* an unusual coord­ination polymer of dppmS_2_ with gold(I); Taouss *et al.*, 2020 ▸). Several of these have formed a series ‘phosphane chalcogenides and their metal complexes’, of which this paper is Part 6; the metal has so far always been gold, but we plan to publish related structures involving platinum and palladium. Mention should also be made of the work by Laguna and Gimeno on diphosphane sulfide and selenide derivatives bonded to organometallic gold moieties, often involving C_6_F_5_ ligands (see *e.g*. Álvarez *et al.*, 1998 ▸ or Canales *et al.*, 2007 ▸).

In a preliminary communication (Taouss & Jones, 2011 ▸), we reported that the oxidation of bromido­(tri­phenyl­phosphane sulfide)­gold(I) AuBr(SPPh_3_) with excess elemental bromine led to the unexpected ionic product (Ph_3_PSBr)(AuBr_4_), the (bromo­thio)­phospho­nium cation of which contains an unprecedented P—S—Br group. Similarly, in a second communication, concerning the oxidation of tri­alkyl­phosphane complexes (*R*_3_P=*E*)AuCl (*R*_3_ = various combinations of *i*-propyl and *t*-butyl; *E* = S, Se) with the Cl_2_-equivalent PhICl_2_, we established the main products, depending on the amount of oxidizing agent used, to be the expected (*R*_3_P=*E*)AuCl_3_ or the halogenido­phospho­nium salts (*R*_3_P*E*Cl)(AuCl_4_), with novel P—*E*—Cl groups in the cation (Upmann & Jones, 2013 ▸). Similar results were obtained for the AuBr analogues, but were not reported at the time. We now intend to present the full structural details of the studies with tri­alkyl­phosphane chalcogenides, beginning in this paper with the halogenido-gold(I) starting materials (^*t*^Bu_3-_ *_n_^i^* Pr_*n*_P=*E*)Au*X*, of which there are sixteen possible permutations of *n*, *E* and *X* (for *X* = Cl or Br). The chlorido derivatives are: **1a**, *n* = 3, *E* = S; **2a**, *n* = 2, *E* = S; **3a**, *n* = 1, *E* = S; **4a**, *n* = 0, *E* = S; **5a**, *n* = 3, *E* = Se; **6a**, *n* = 2, *E* = Se; **7a**, *n* = 1, *E* = Se; and **8a**, *n* = 0, *E* = Se, and the corresponding bromido derivatives are **1b**–**8b** in the same order. However, we obtained only thirteen usable structures; compound **8a** was obtained, but decomposed rapidly, whereas **2a** and **2b** proved to be severely disordered. The structures of **3a**, **6a** and **7a** were briefly presented in our communication (Upmann & Jones, 2013 ▸), but have been re-refined using a much more recent version of *SHELXL* (2019 rather than 1997; Sheldrick, 2015 ▸) and are discussed in more detail here. 
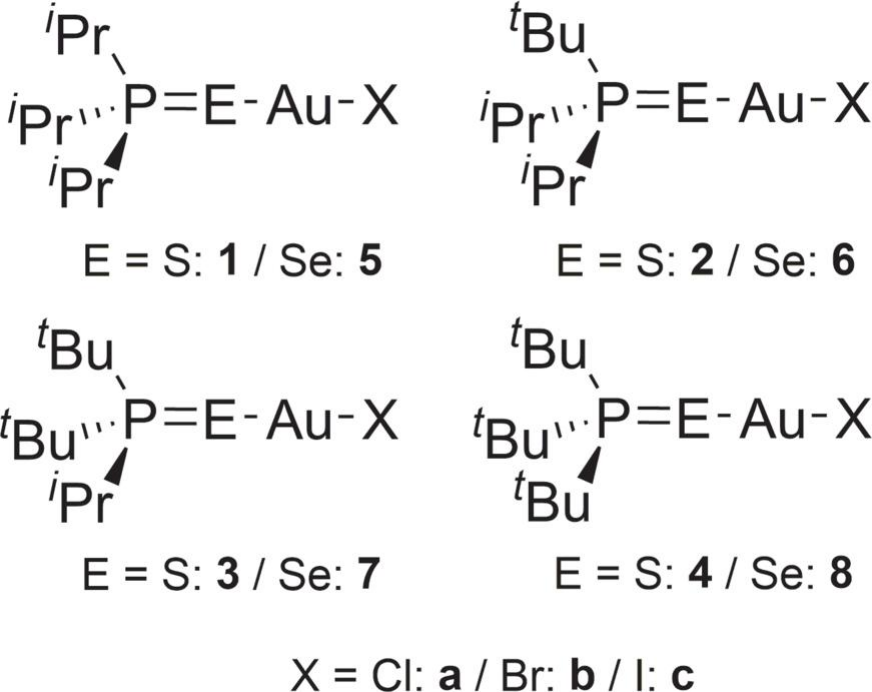


The synthesis of the chlorido- and bromido­gold(I) derivatives is straightforward and involves reacting the appropriate phosphane with the well-known starting materials AuCl(tht) and AuBr(tht), where ‘tht’ is the easily replaceable ligand tetra­hydro­thio­phene (see Section 5). The synthesis of iodido­gold(I) derivatives is less simple, because the starting material AuI(tht) does not exist as such; a compound with the same stoichiometry can be synthesized, but has the ionic composition [Au(tht)_2_][AuI_2_] (Ahrland *et al.*, 1985 ▸) and is thus not suitable as a starting material for the synthesis of complexes *L*AuI (*L* = any neutral ligand). Nevertheless we succeeded in synthesizing three iodido derivatives **2c**, **6c** and **7c** (numbered as for the series **a** and **b**) by stirring a solution of the chlorido derivative in di­chloro­methane with an aqueous solution of potassium iodide (Upmann, 2015 ▸), and in determining their structures. This gave a total of sixteen structures, which are reported here. It may be useful to summarize the various types of heavy-atom sequence: **1a**, **3a** and **4a** have the sequence P—S—Au—Cl; **1b**, **3b** and **4b** have P—S—Au—Br; **5a**, **6a** and **7a** have P—Se—Au—Cl; **5b**, **6b**, **7b** and **8b** have P—Se—Au—Br; **2c** has P—S—Au—I; and **6c** and **7c** have P—Se—Au—I.

The corresponding trihalogenido-gold(III) complexes will be discussed in the next paper in this series. There are however no tri­iodido derivatives, because the oxidizing power of elemental iodine is not sufficient to generate these from the gold(I) starting materials.

## 2. Structural commentary

**Figure 1:**
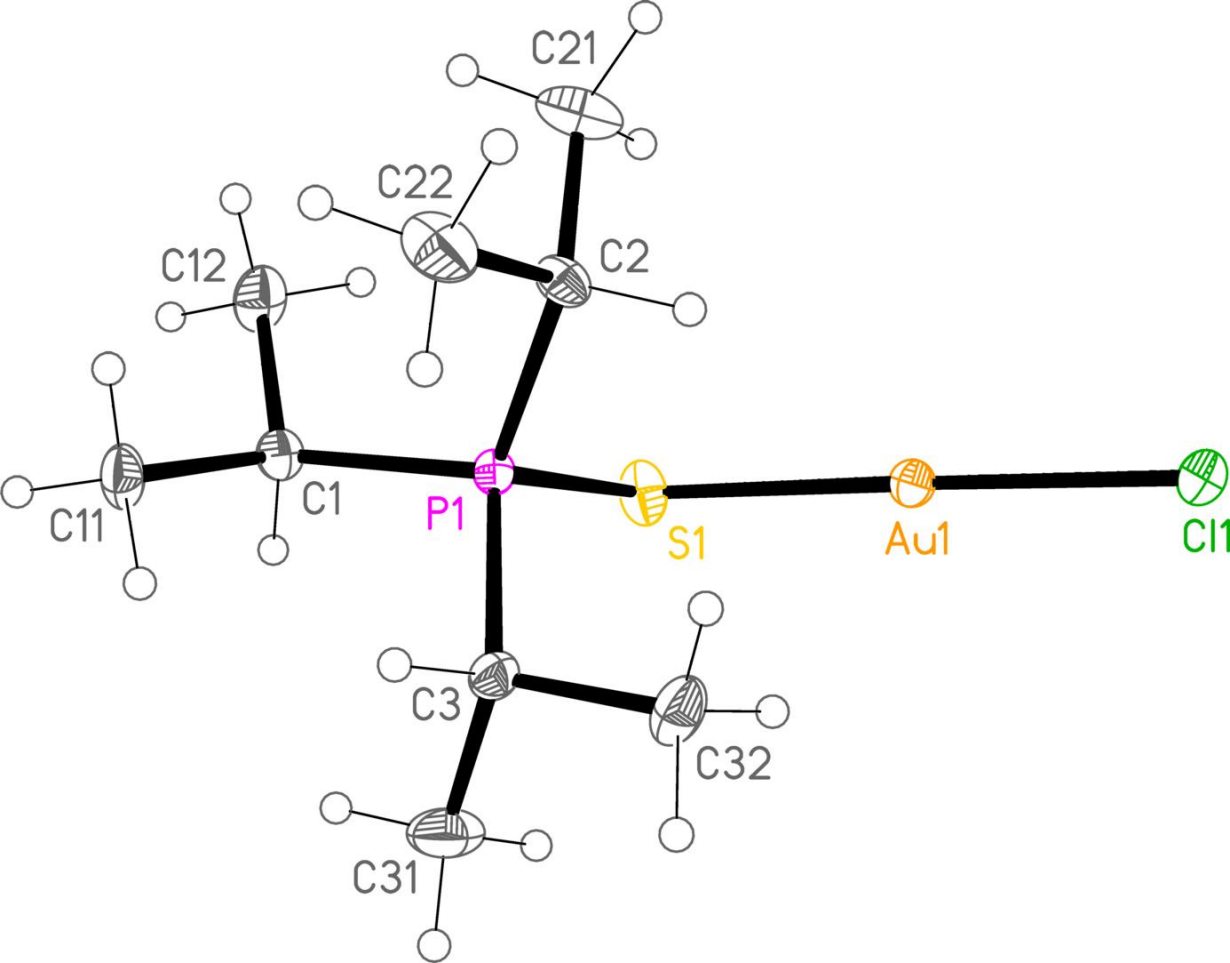
The structure of compound **1a** in the crystal. Ellipsoids represent 50% probability levels.

*General comments*: The mol­ecular structures are shown in Figs. 1 ▸–16 ▸ ▸ ▸ ▸ ▸ ▸ ▸ ▸ ▸ ▸ ▸ ▸ ▸ ▸ ▸; selected mol­ecular dimensions are given in Tables 1 ▸–16 ▸ ▸ ▸ ▸ ▸ ▸ ▸ ▸ ▸ ▸ ▸ ▸ ▸ ▸ ▸. As expected, all compounds show linear coordination geometry (angles *ca* 173–178°) at the gold(I) centres, and angles of *ca* 101–109° at the chalcogenide atoms. All compounds crystallize solvent-free and with one mol­ecule in the asymmetric unit except for **6b** and **6c**, which have *Z*′ = 2; a least-squares fit of the two independent mol­ecules (excluding hydrogen atoms) gave an r.m.s. deviation of 0.11 Å for **6b** and 0.13 Å for **6c**. Structures such as the **c** series in this paper, of the type *R*_3_P*E*AuI, where *R*_3_ represents any combination of alkyl or aryl groups, were represented until now only by the iodido derivative Ph_3_PSeAuI; our attempts to obtain analogous sulfur compounds led mostly to disordered structures in which the *R*_3_P=S—Au—I mol­ecule was overlaid by a di­iodine adduct of the type *R*_3_P=S⋯I—I (Taouss *et al.*, 2015 ▸).

*Isotypy*: In an extensive series of closely analogous structures, several would be expected to be isotypic. Indeed, the five compounds **1a**, **5a**, **6a**, **1b** and **5b** form an isotypic set, despite the different alkyl groups in **6a**. Compounds **3a**/**3b**, **4b**/**8b** and **6b**/**6c** also form isotypic pairs.

*Bond lengths and angles (1). P—E—Au—X groups*: The bond lengths among the heavy atoms are remarkably constant for the various classes. Thus the P—S and P—Se bond lengths lie in the ranges 2.0322–2.0482 (av. 2.0368) Å and 2.1860–2.2027 (av. 2.1938) Å, respectively, both markedly lengthened with respect to the ‘standard’ bond lengths of *ca* 1.95 and 2.11 Å respectively in the free ligands (as would be expected; see Section 4). The bond lengths at the gold atoms might however be expected to show some *trans* influences, and there are indeed some weak trends. Thus Au—S bond lengths lie in the range 2.2673–2.2959 (av. 2.2760) Å, with separate averages of 2.2692 Å *trans* to Cl, 2.2761 Å *trans* to Br and 2.2959 Å (one value only) *trans* to I, which may indicate a weak correlation of the Au—S bond length with the softness of the halogen atom. A similar weak effect is observed for Au—Se; overall 2.3696–2.4040 (av. 2.3845) Å, with subset averages 2.3727 Å *trans* to Cl, 2.3813 Å *trans* to Br and 2.4017 Å *trans* to I. Similarly, the Au—Cl bond lengths (2.2761–2.2898, av. 2.2840 Å) are marginally shorter *trans* to S (av. 2.2800 Å) than *trans* to Se (av. 2.2879 Å), and the Au—Br bond lengths (2.3896–2.4040, av. 2.3979 Å) are similarly just shorter *trans* to S (av. 2.3928 Å) than *trans* to Se (av. 2.4010 Å). The four Au—I bond lengths are almost constant (2.5437–2.5509, av. 2.5489 Å), but are too few to provide reliable trends.

The angles P—*E*—Au lie in the range 104.56–107.77 (av. 106.17)° for *E* = S and 100.57–106.91 (av. 103.86)° for *E* = Se; this might be taken to indicate a slightly lower involvement of the *s* valence orbital for Se than for S, but the ranges overlap considerably. The angles at gold are all close to linearity (173.96–177.70°) and show no clear trends.

*Bond lengths and angles (2). Phosphane ligands*: The central atoms of the alkyl groups are numbered C1, C2, C3, such that the *t*-butyl groups are assigned the lowest numbers. In phosphanes involving both types of alkyl groups, the carbon atom anti­periplanar to Au across the Au—*E*—P—C sequence generally belongs to an *i*-propyl group (the exceptions are **3b** and **3a**). The phosphane groups involve bulky substituents, especially for tri-*t*-butyl­phosphane; accordingly, most C—P—C bond angles at phospho­rus are greater than the ideal tetra­hedral value. One compensation for this lies in a narrow *E*—P—C angle to the carbon atom anti­periplanar to *E*, with values as low as 101° (but this effect is less pronounced for **2c**, **6b** and **6c**). The steric crowding is also reflected, especially for the tri-*t*-butyl­phosphane derivatives **4a**, **4b** and **8b**, in several short intra­molecular C—H⋯*E* and C—H⋯Au contacts (the latter with H⋯Au as short as 2.63 Å), which are listed for convenience in the tables of hydrogen bonds, even if this description of the contacts may be inappropriate.

*Mol­ecular volumes*: The change in mol­ecular volume (cell volume/*Z*) on changing the elements *E* or *X* (for the same phosphane) is calculated for six pairs S/Se as 2.6–5.7, av. 4.7 Å^3^ (the pair **4b**/**8b** is the outlier, at 2.6 Å^3^); for six pairs Cl/Br as 9.4–13.8, av. 11.4 Å^3^; and for two pairs Br/I as 15.3 and 16.9, av. 16.1 Å^3^. The expected changes, using the room-temperature values of Hofmann (2002 ▸) (S 25.5, Se 30.3, Cl 25.8, Br 32.7, I 46.2 Å^3^), which were fitted to unit-cell volumes of 182239 structures, would be 5.1 for S/Se, 6.9 for Cl/Br and 13.5 for Br/I. Except for Cl/Br, the expected and observed volume changes fit reasonably well, although any particular atomic volume must vary considerably with the chemical environment. Any effects of thermal contraction should be minimal; Hofmann (2002 ▸) calculated an overall thermal expansion coefficient of *ca* 10 ^−4^ K^−1^.

## 3. Supra­molecular features

The mol­ecular packing might in principle involve any of the following types of secondary inter­action: (1) ‘Weak’ hydrogen bonds C—H⋯*E* or C—H⋯halogen; see *e.g.* Brammer (2003 ▸) for the concept of hydrogen bonding to metal-bonded halogen atoms. (2) Weak hydrogen bonds C—H⋯Au, although such contacts may simply be attributable to the steric accessibility of linearly coordinated Au^I^ centres; see Schmidbaur *et al.* (2014 ▸) and Schmidbaur (2019 ▸). The most probable hydrogen-bond donors would be the methine hydrogens of the isopropyl groups; du Mont’s group was able to show the importance of such inter­actions in determining the mol­ecular form of some selenium dibromide adducts (Hrib *et al.*, 2006 ▸). Hydrogen bonds are given in Tables 17 ▸–32 ▸ ▸ ▸ ▸ ▸ ▸ ▸ ▸ ▸ ▸ ▸ ▸ ▸ ▸ ▸; these include intra­molecular contacts (see above) and, for completeness, several borderline contacts that are not all discussed below. Symmetry operators, not given explicitly in the following discussion, may also be found in these Tables. (3) Halogen–halogen contacts (see *e.g.* Metrangelo, 2008 ▸) or other ‘soft–soft’ contacts involving the atoms *E* or *X*. (4) Au⋯Au contacts, known as aurophilic contacts; these are a frequent feature of simple Au^I^ derivatives and have been reviewed by Schmidbaur & Schier (2008 ▸, 2012 ▸). (5) Au⋯*E* or Au⋯*X* contacts. In all packing diagrams presented here, hydrogen atoms not involved in hydrogen bonding are omitted for clarity, and the atom labels indicate the asymmetric unit. Since X-ray methods reveal short inter­molecular contacts, but not the corresponding energies, the following descriptions of mol­ecular packing in terms of particular secondary contacts must to some extent be subjective.

The packing of compound **1a** (and by extension the four structures that are isotypic to **1a**) does indeed involve both methine hydrogen atoms, which form hydrogen bonds H3⋯Cl1 *via* the 2_1_ screw axis and H1⋯S1 (rather long, but acceptably linear) *via* an inversion centre. These combine to form a layer structure parallel to (10 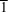 ) (Fig. 17 ▸).

The packing of compounds **3a** and **3b** is almost featureless; the shortest H⋯Cl/Br distances are 3.03/3.12 Å, respectively, and the shortest H⋯S contacts, over inversion centres, have very narrow C—H⋯S angles. The disorder of **3a** would make any dimensions involving the disordered *t*-butyl hydrogens unreliable. For the sake of completeness, we present a view of the double-layer structure of **3b** parallel to the *bc* plane (Fig. 18 ▸); there are two such double layers per cell.

Two short C_meth­yl_—H⋯Cl inter­actions based on translations combine in compound **4a** to form a layer structure parallel to the *ac* plane (Fig. 19 ▸). Similarly, the two shortest C_meth­yl_—H⋯Br contacts in compound **4b** (and the isotypic **8b**) combine *via* the *c* glide and an inversion centre to form a layer structure parallel to the *bc* plane (Fig. 20 ▸). All these structures lack methine hydrogen atoms.

In the packing of compound **7a**, the methine hydrogen atom is involved in a short C—H⋯Cl hydrogen bond, forming chains of mol­ecules *via* the 2_1_ screw axis. The chains are linked to form a layer structure (Fig. 21 ▸) parallel to the *bc* plane by association of Au—Se moieties across an inversion centre, forming a planar Au_2_Se_2_ quadrilateral with Au⋯Se = 3.4748 (3) Å, Au—Se⋯Au′ = 106.51 (1) and Se⋯Au′—Se′ = 73.49 (1)° (where the primes indicate the inversion operator −*x*, 1 − *y*, −*z*). For the isotypic **7b**, the corresponding dimensions are 3.4305 (5) Å, 103.28 (1) and 76.72 (1)°.

The packing of compound **6b** (and the isotypic **6c**) can conveniently be analysed for each of the two independent mol­ecules separately, because they occupy different regions of the cell, with mol­ecule 1 (based on Au1) at *x* ≃ 0 and mol­ecule 2 (based on Au2) at *x* ≃ 0.5. Mol­ecule 1 forms chains parallel to the *b* axis (Fig. 22 ▸) in which the mol­ecules are linked *via* the 2_1_ screw axis by the C_methine_⋯gold contact C2—H2⋯Au1, with H⋯Au 2.98 Å. Such contacts are not infrequent for gold(I) derivatives; whether they represent genuine hydrogen bonds (to the most electronegative metal), or simply reflect the sterically exposed nature of the *E*—Au—*X* moiety, is a moot point. The mol­ecules 2 associate *via* a short Se2⋯Se2 contact [3.5565 (5) Å, operator 1 − *x*, 1 − *y*, 1 − *z*; the corresponding Au2⋯Se2 distance of 3.9480 (3) Å is much longer] and a short H2⋯Br5 contact to form a layer structure parallel to the *bc* plane (Fig. 23 ▸). The layers are connected by the contacts H6⋯Br1, H42*A*⋯Br1 and H3⋯Br2. In **6c**, the Se2⋯Se2 contact distance is 3.6157 (6) Å, but the associated Au2⋯Se2 contact of 3.7695 (3) Å is much shorter than in **6b**.

The packing of compound **2c** can be described in terms of its two shortest C—H⋯I contacts, which as expected involve methine hydrogens. These combine *via* the 2_1_ screw axis and an inversion centre to form a corrugated ribbon structure parallel to the *b* axis (Fig. 24 ▸). The significance of C—H⋯I contacts may however not be great. The hydrogen atom H2 also forms a short (and more linear) contact to the gold atom of the same AuI moiety, so that the inter­action might be described as the three-centre type C—H⋯(Au, I).

The major packing feature of **7c** is the formation of twofold-symmetric dimers (Fig. 25 ▸) *via* a short aurophilic contact Au1⋯Au1(−*y*, −*x*, 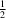 − *z*) = 3.0914 (6) Å; **7c** is the only compound in this paper to feature such contacts. The linear groupings Se—Au—I of the two mol­ecules are mutually rotated, with torsion angles I1—Au1⋯Au1′—I1′ = 117.89 (4)°, Se1—Au1⋯Au1′—Se1′ = 123.62 (6)° and I1—Au1⋯Au1′—Se1′ = −59.25 (3)° (primes represent the symmetry-equivalent atoms). Apart from this, the packing is almost featureless, consisting of layers (at *z* ≃ 0, 0.25, 0.5 and 0.75) with only translation symmetry and few short contacts except for two very borderline Au⋯H (Fig. 26 ▸). The shortest H⋯I contacts are 3.20 Å.

## 4. Database survey

The searches employed the routine ConQuest (Bruno *et al.*, 2002 ▸), part of Version 2022.3.0 of the CSD (Groom *et al.*, 2016 ▸).

A search for all phosphane sulfides of the type C_3_P=S, with coordination numbers of 4 and 1 respectively for the phospho­rus and sulfur atoms, gave 1259 hits, with 1318 P—S bond lengths, average 1.954 (23) Å. An analogous search for C_3_P=Se gave 398 hits, 603 P—Se bond lengths, av. 2.109 (14) Å. Separate searches for triaryl- and tri­alkyl­phosphane chalcogenides showed no significant differences in the average bond length.

Similar searches for phosphane chalogenide complexes with transition metals (with coordination number 2 at the atom *E*), gave for *E* = S 559 hits, 909 P—S bond lengths, average 2.009 (22) Å and for *E* = Se 114 hits, 184 P—Se bond lengths, average 2.158 (23) Å. The differences between average coordinated and uncoordinated P—*E* bond lengths are thus 0.055 Å for *E* = S and 0.049 Å for *E* = Se.

As mentioned above, there are few structures containing the moiety ‘C_3_P*E*AuBr’. A database search found the following three structures (excluding our own work, which was cited above): Cy_3_PSeAuBr (Cy = cyclo­hexyl; refcode QUTNUO; Hussain & Isab, 2000 ▸); Ph_3_PSAuBr (ADOLUA; Hussain *et al.*, 2001 ▸); and Ph_3_PSeAuBr (MIVXOE; Hussain & Isab, 2001 ▸).

## 5. Synthesis and crystallization

For most of the compounds, the syntheses can be found in the PhD thesis of D. Upmann (Upmann, 2015 ▸). The following do not appear there:

Compound **4a**. Solutions of AuCl(tht) (200 mg, 0.624 mmol) and ^*t*^Bu_3_PS (146.2 mg; 0.624 mmol), each in 5 mL of di­chloro­methane, were combined and stirred for 30 min at room temperature. The solution quickly turned orange. The solvent was removed under vacuum and the solid residue washed with *n*-pentane and dried under vacuum. Recrystallization from di­chloro­methane/*n*-pentane gave a pale-yellow crystalline solid. Yield: 205.1 mg (0.439 mmol, 70%). ^31^P-NMR (81 MHz, CDCl_3_, 300 K) δ (ppm): 86.5 (*s*). Elemental analysis (%): calc.: C 30.88, H 5.83, S 6.87; found: C 29.36, H 5.49, S 7.34. Single crystals were obtained by liquid diffusion of *n*-pentane into a solution of **4a** in di­chloro­methane.

Compound **1b**. Solutions of AuBr(tht) (250 mg; 0.695 mmol) and ^*i*^Pr_3_PS (131.7 mg; 0.685 mmol), each in 5 mL of di­chloro­methane were combined and stirred for 10 min at RT; the solution was pale yellow. The solvent was removed under vacuum and the solid residue washed with *n*-pentane and dried under vacuum, giving **1b** as a colourless solid without further purification. Yield: 269.6 mg (0.575 mmol, 84%). ^31^P-NMR (81 MHz, CDCl_3_, 300 K) δ (ppm): 76.1 (*s*). Elemental analysis (%): calc.: C 23.04, H 4.51, S 6.83; found: C 23.18, H 4.57, S 7.31. Single crystals were obtained by liquid diffusion of *n*-pentane into a solution of **1b** in di­chloro­methane.

Compound **4b**. Solutions of AuBr(tht) (400 mg; 1.096 mmol) and ^*t*^Bu_3_PS (256.8 mg; 1.096 mmol), each in 5 mL of di­chloro­methane, were combined and stirred for 10 min at RT. The solution quickly turned red, which was surprising in view of the expected colourless product. The product was precipitated by the addition of *n*-pentane. The remaining red solution was pipetted off and discarded, and the solid dried under vacuum. Yield: 368.1 mg (0.720 mmol; 66% assuming the correct product). Despite several attempts using slightly varied conditions, the product always consisted of a mixture of colourless and red crystals. The amount of the latter was small, but prevented the recording of satisfactory elemental analyses. ^31^P-NMR (81 MHz, CDCl_3_, 300 K) δ (ppm): 86.8 (*s*) for the major product and 134.6 (s) (with a relative integrated intensity of *ca* 1–2%) for the red product. The crystal structure of the latter (to be reported elsewhere) showed it to be the gold(III) di-*t*-butyl­dithio­phosphinate complex Au(^*t*^Bu_2_PS_2_)Br_2._

Compound **5b**. Solutions of AuBr(tht) (250 mg; 0.695 mmol) and ^*i*^Pr_3_PSe (163.8 mg; 0.685 mmol), each in 5 mL of di­chloro­methane, were combined and stirred for 10 min at RT. The pale-yellow solution was then evaporated to dryness under vacuum to give the product as a beige-coloured solid without further purification. Yield 289.8 mg (0.562 mmol, 82%). ^31^P-NMR (81 MHz, CDCl_3_, 300 K) δ (ppm): 71.3 (*s*, ^1^ *J*_P–Se_ = 535 Hz). Elemental analysis (%): calc.: C 20.95, H 4.10; found: C 21.15, H 4.06. Single crystals were obtained by liquid diffusion of *n*-pentane into a solution of **5b** in di­chloro­methane.

Compound **8b**. Solutions of AuBr(tht) (400 mg; 1.096 mmol) and ^*t*^Bu_3_PSe (308.2 mg; 1.096 mmol), each in 5 mL of di­chloro­methane, were combined and stirred for 10 min at r.t. The product was precipitated by the addition of *n*-pentane. The solution was pipetted off and discarded and the remaining solid dried under vacuum to obtain the product as a beige-coloured solid. Yield 237.0 mg (0.425 mmol, 39%). ^31^P-NMR (81 MHz, CDCl_3_, 300 K) δ (ppm): 86.5 (*s*, ^1^ *J*_P–Se_ = 552 Hz). Elemental analysis (%): calc.: C 25.82, H 4.88; found: C 25.79, H 4.87. Single crystals were obtained by liquid diffusion of *n*-pentane into a solution of **8b** in deuterated chloro­form. Alternative syntheses with lower concentrations but longer reaction times gave better yields, but with unsatisfactory elemental analyses.

## 6. Refinement

Details of the measurements and refinements are given in Table 33 ▸. Structures were refined anisotropically on *F* ^2^. Methine hydrogen atoms were included at calculated positions and refined using a riding model with C—H = 1.00 Å and *U*_iso_(H) = 1.2*U*_eq_(C). Methyl groups were refined, using the command "AFIX 137", as idealized rigid groups allowed to rotate but not tip, with C—H = 0.98 Å, H—C—H = 109.5° and *U*_iso_(H) = 1.5*U*_eq_(C). This command determines the initial hydrogen positions (before refinement) by analysis of maxima in the residual electron density at suitable C—H distances, and these peaks may not be entirely reliable in the presence of a very heavy atom (although in general the refinement seemed to proceed satisfactorily), so that any postulated hydrogen bonds involving methyl hydrogen atoms should be inter­preted with caution.

**Figure 2:**
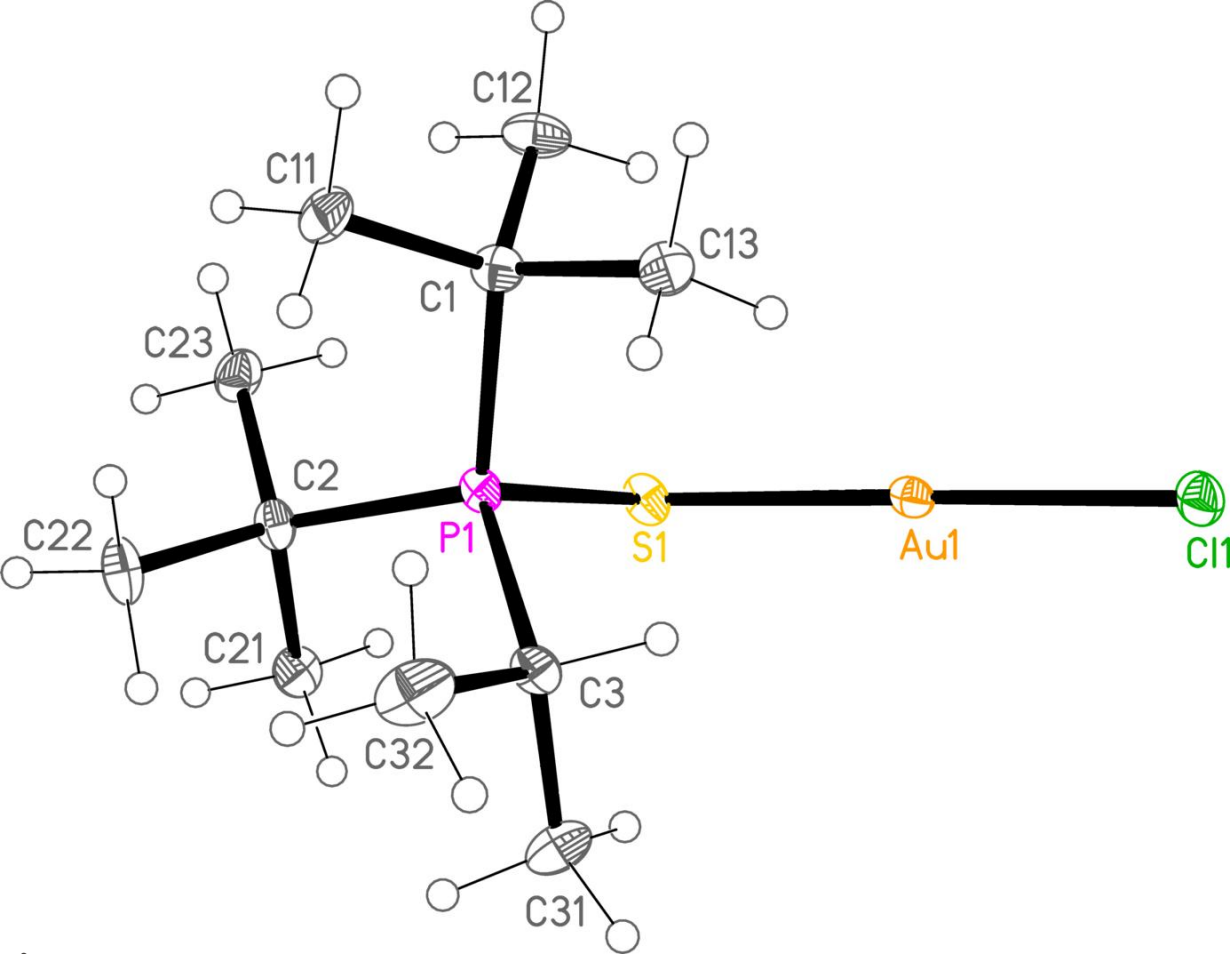
The structure of compound **3a** in the crystal. Ellipsoids represent 30% probability levels. Only the major disorder components of the *t*-butyl groups are shown.

**Figure 17:**
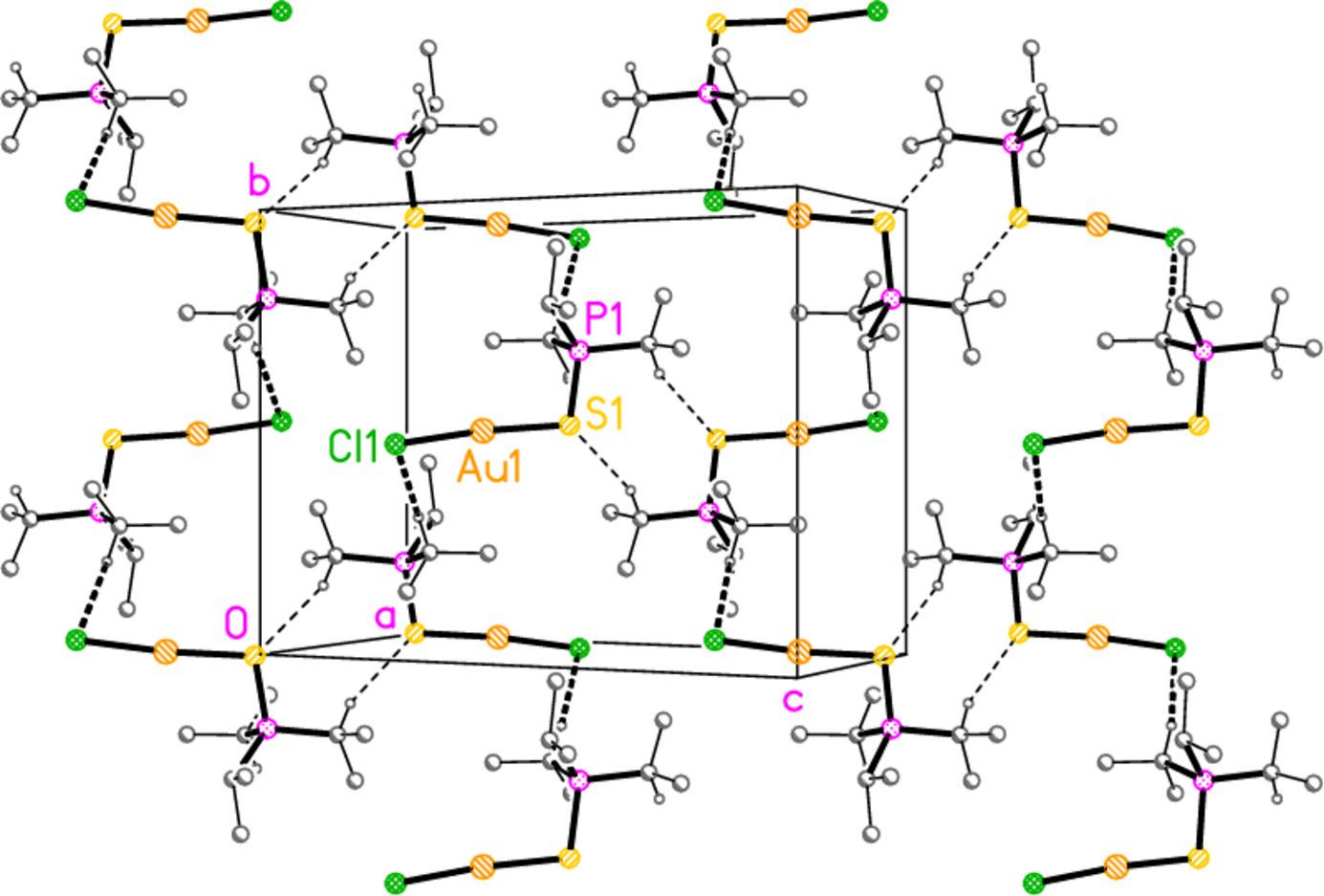
Packing diagram of compound **1a**, viewed perpendicular to (10 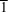 ). Hydrogen bonds are indicated by thin (H⋯S) or thick (H⋯Cl) dashed lines.

**Figure 18:**
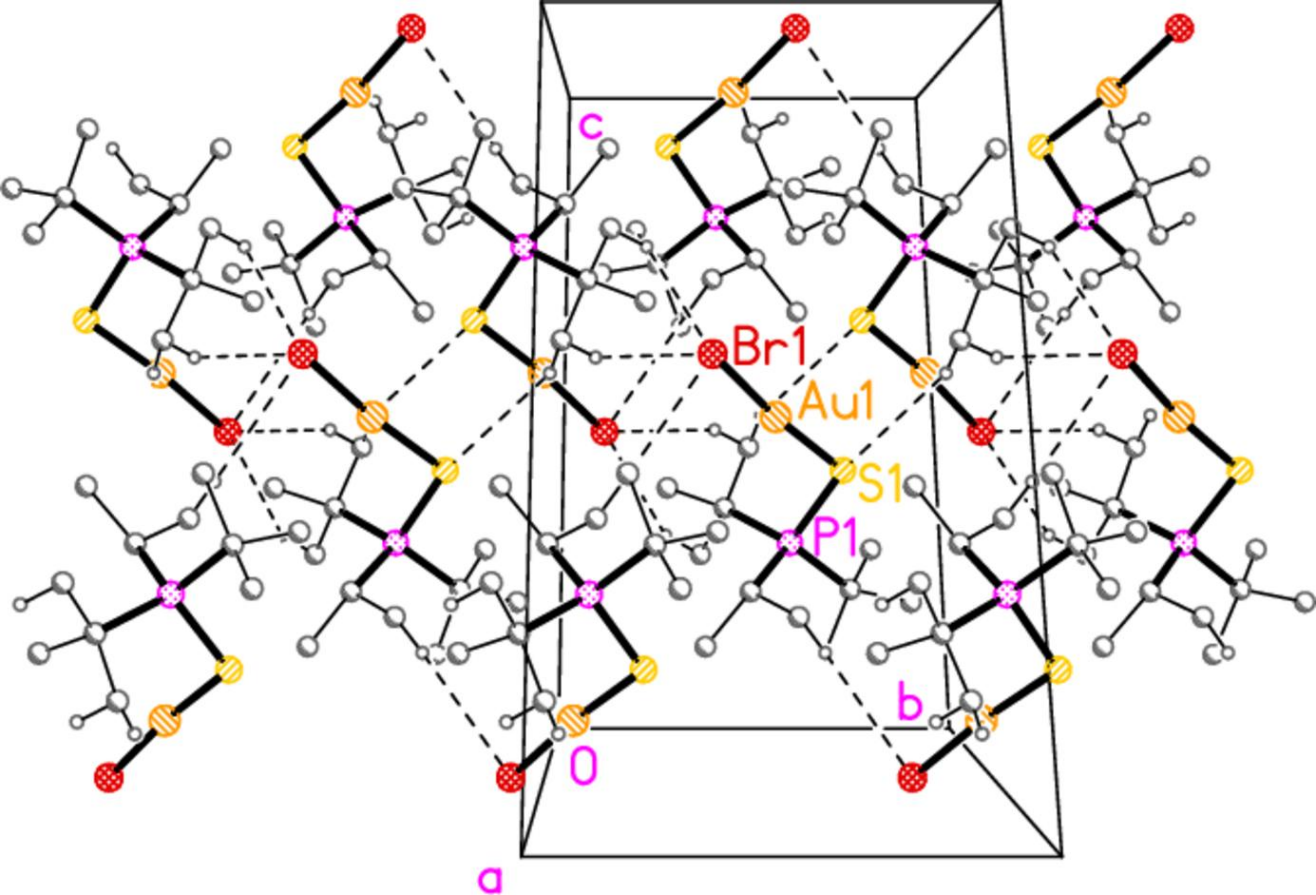
Packing diagram of compound **3b**: a double layer in the region *x* ≃ 0.25, viewed parallel to the *a* axis. The H⋯S and the three shortest H⋯Br contacts are indicated by dashed lines.

**Figure 19:**
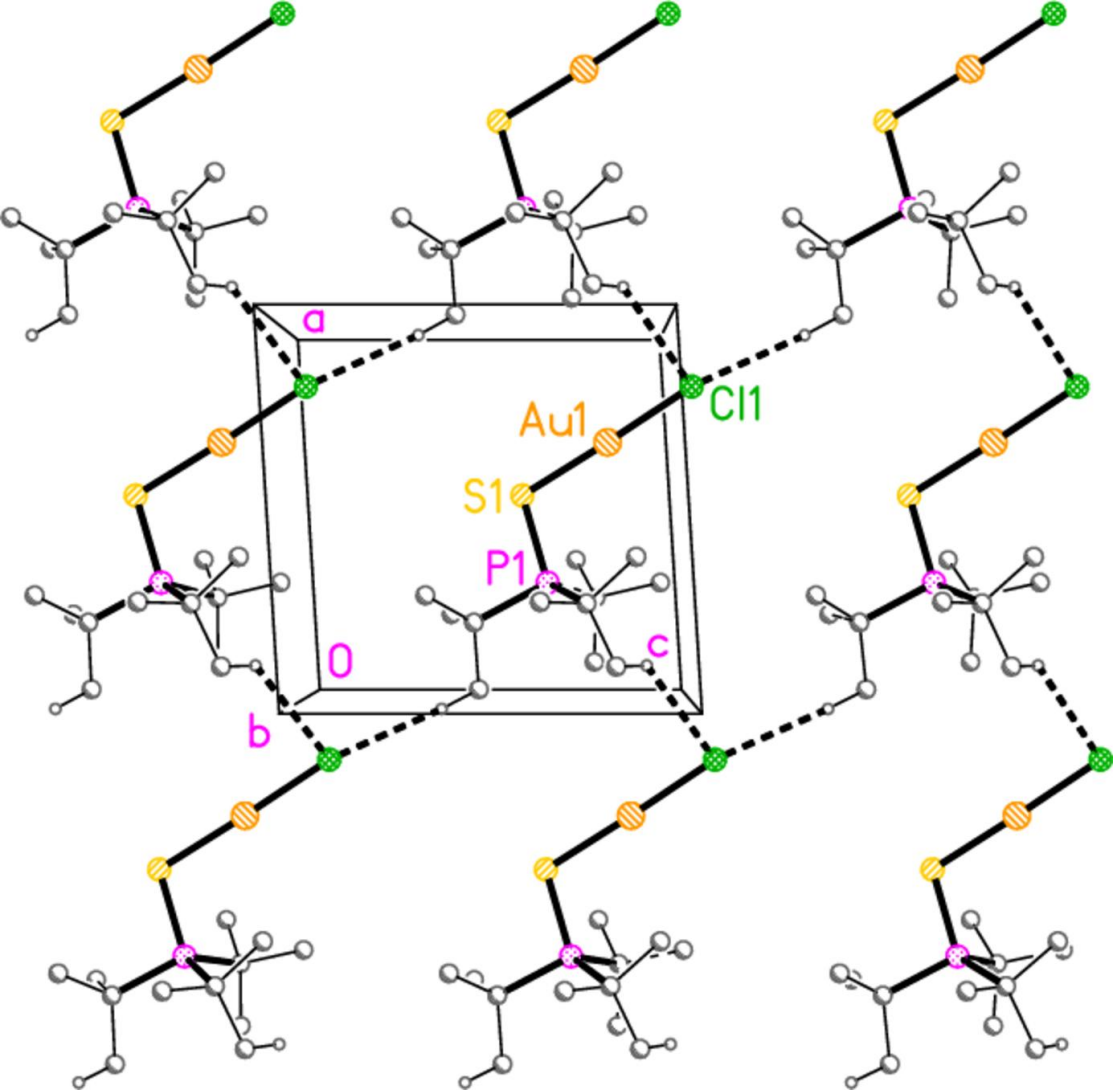
Packing diagram of compound **4a**, viewed parallel to the *b* axis in the region *y* ≃ 0.4. Hydrogen bonds are indicated by thick dashed lines.

**Figure 20:**
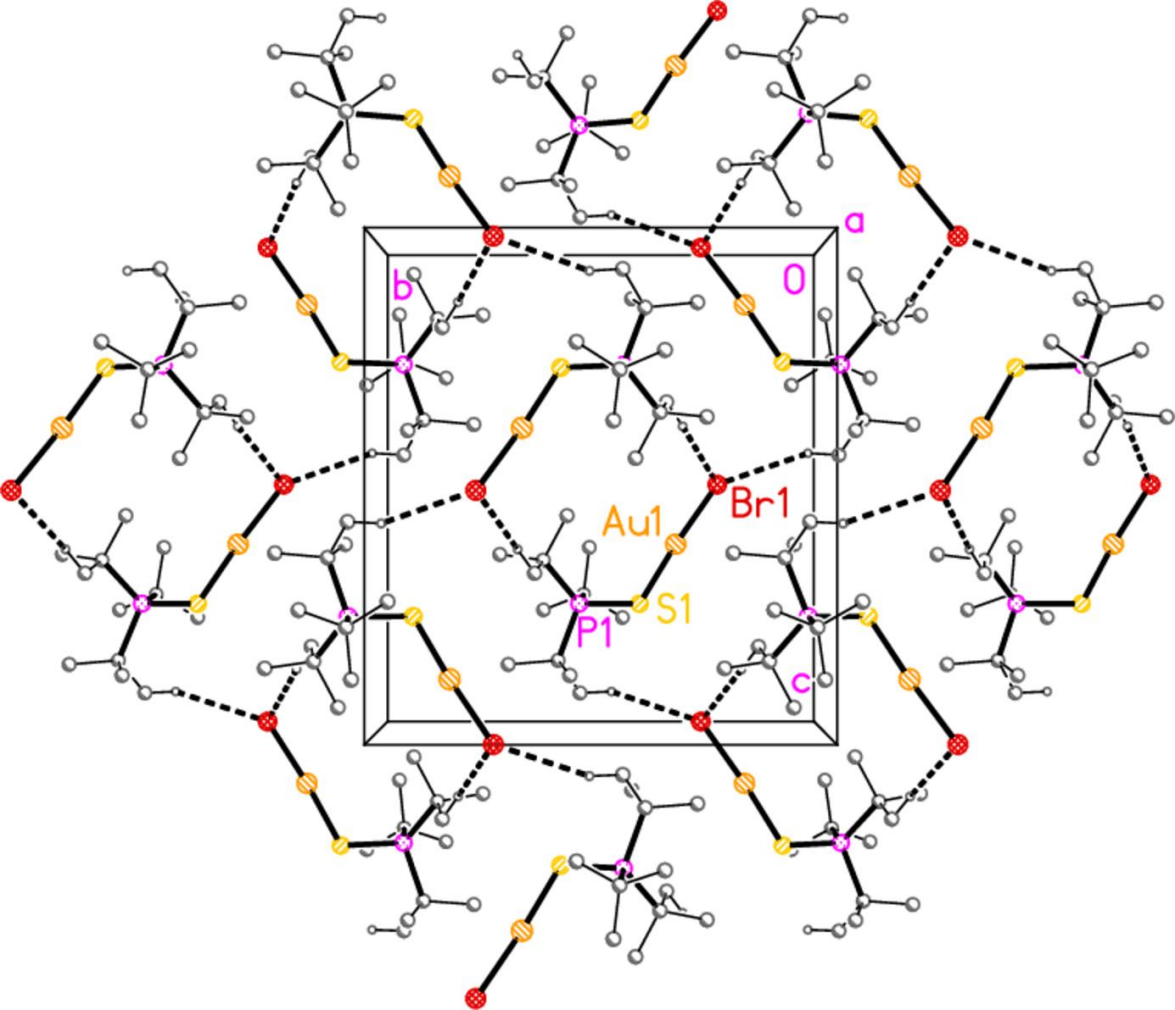
Packing diagram of compound **4b**, viewed parallel to the *a* axis. Hydrogen bonds are indicated by thick dashed lines.

**Figure 21:**
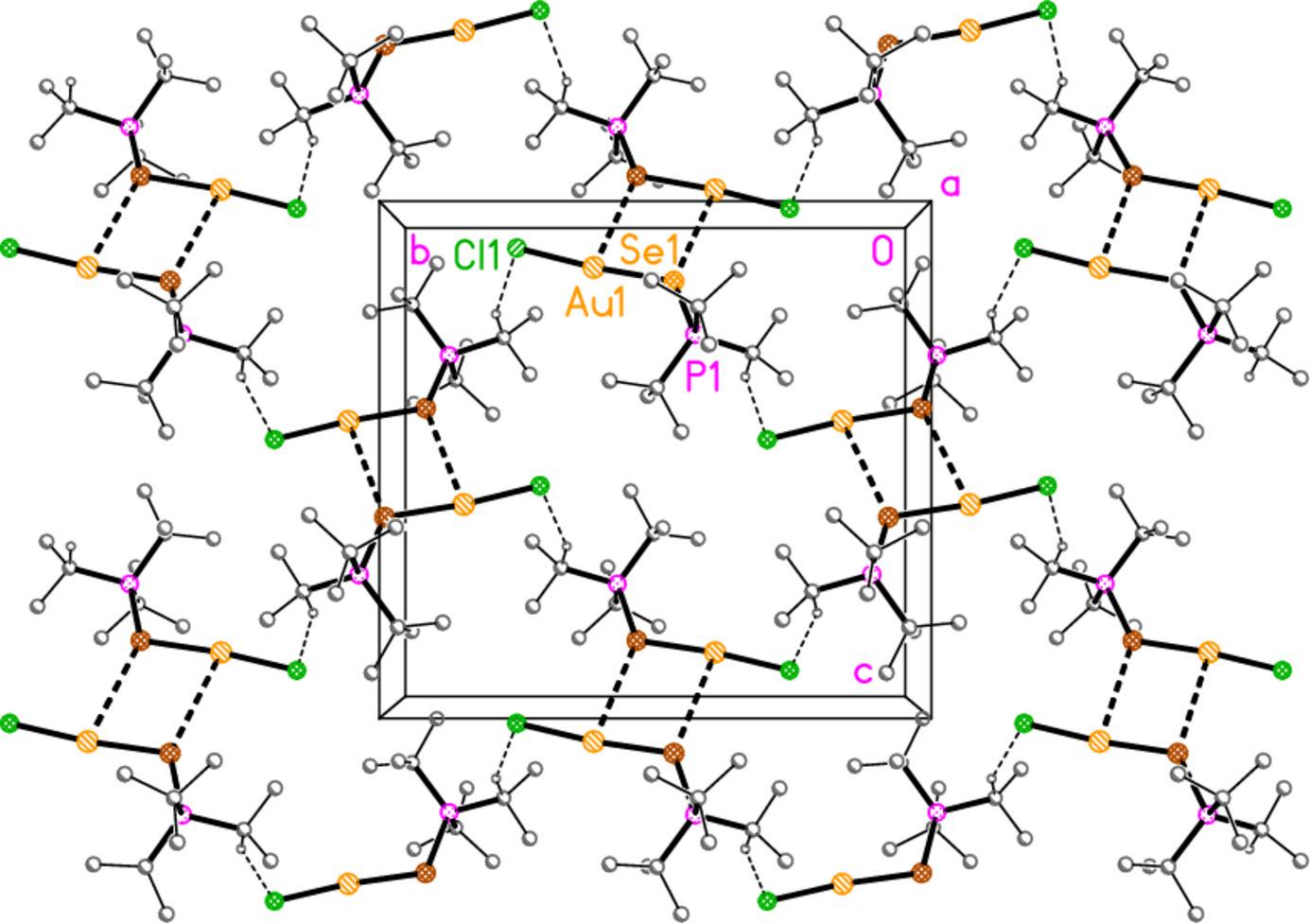
Packing diagram of compound **7a**, viewed parallel to the *a* axis in the region *x* ≃ 0. Dashed lines indicate Au⋯Se contacts (thick) and hydrogen bonds (thin).

**Figure 22:**
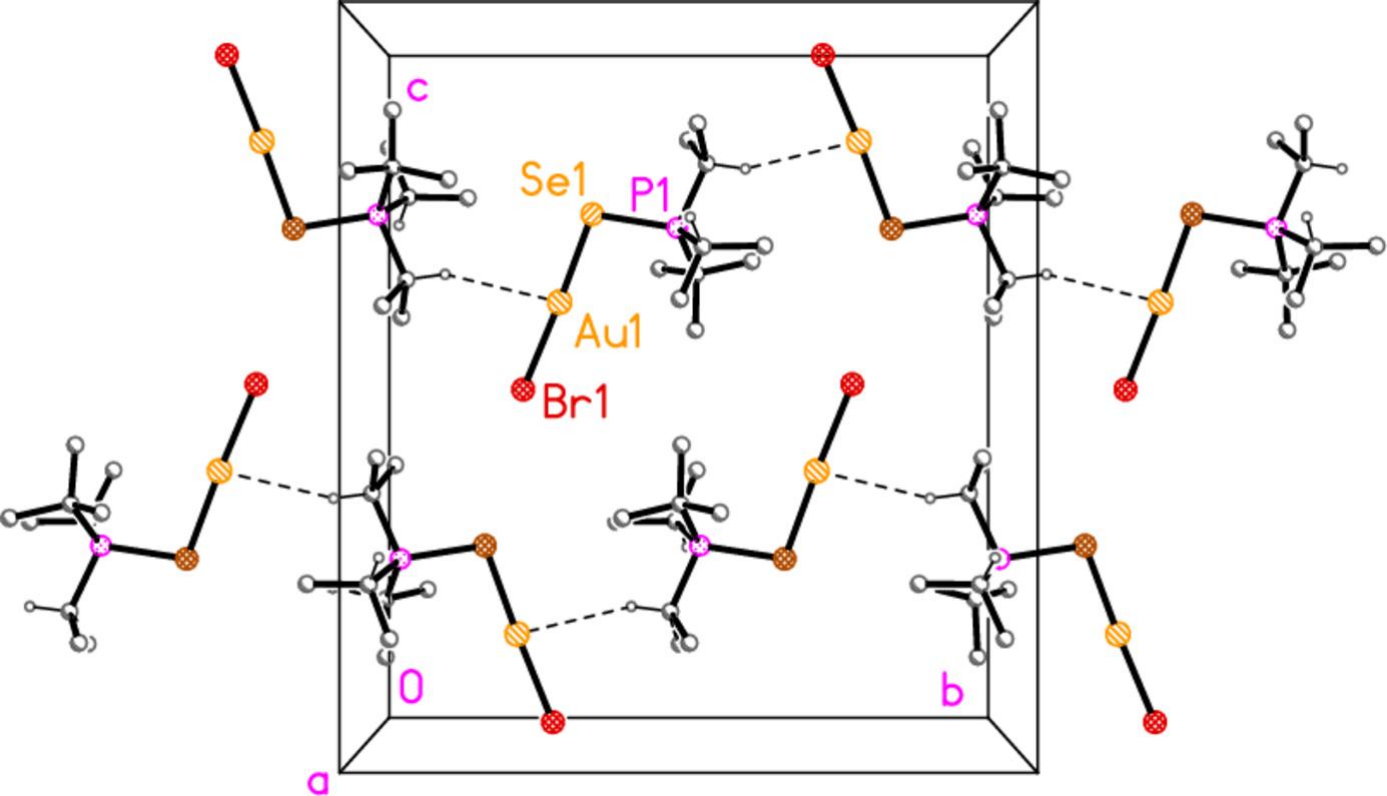
Packing diagram of compound **6b**, mol­ecule 1 only, viewed parallel to the *a* axis in the region *x* ≃ 0. Dashed lines indicate the H⋯Au contacts.

**Figure 23:**
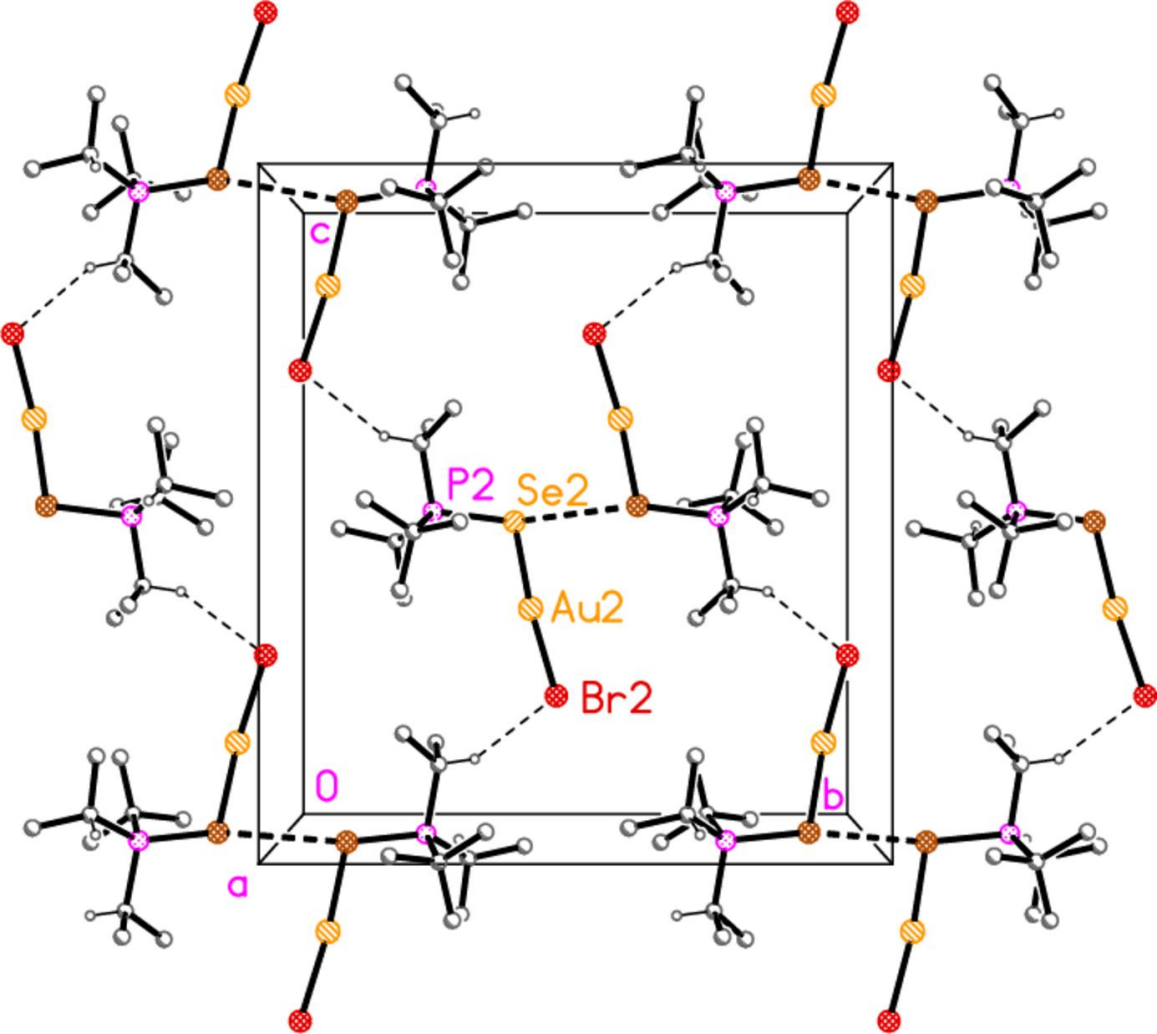
Packing diagram of compound **6b**, mol­ecule 2 only, viewed parallel to the *a* axis in the region *x* ≃ 0.5. Dashed lines indicate Se⋯Se contacts (thick) or H⋯Br contacts (thin).

**Figure 24:**
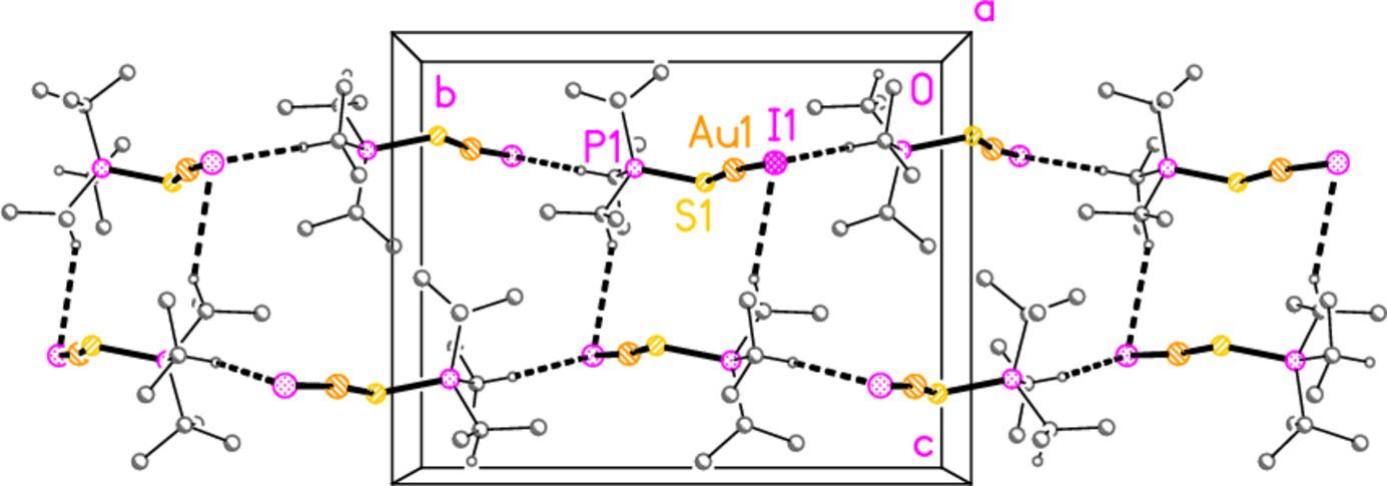
Packing diagram of compound **2c**, viewed parallel to the *a* axis. Dashed lines indicate H⋯I contacts. The H⋯Au contacts (see text) are not shown explicitly.

**Figure 25:**
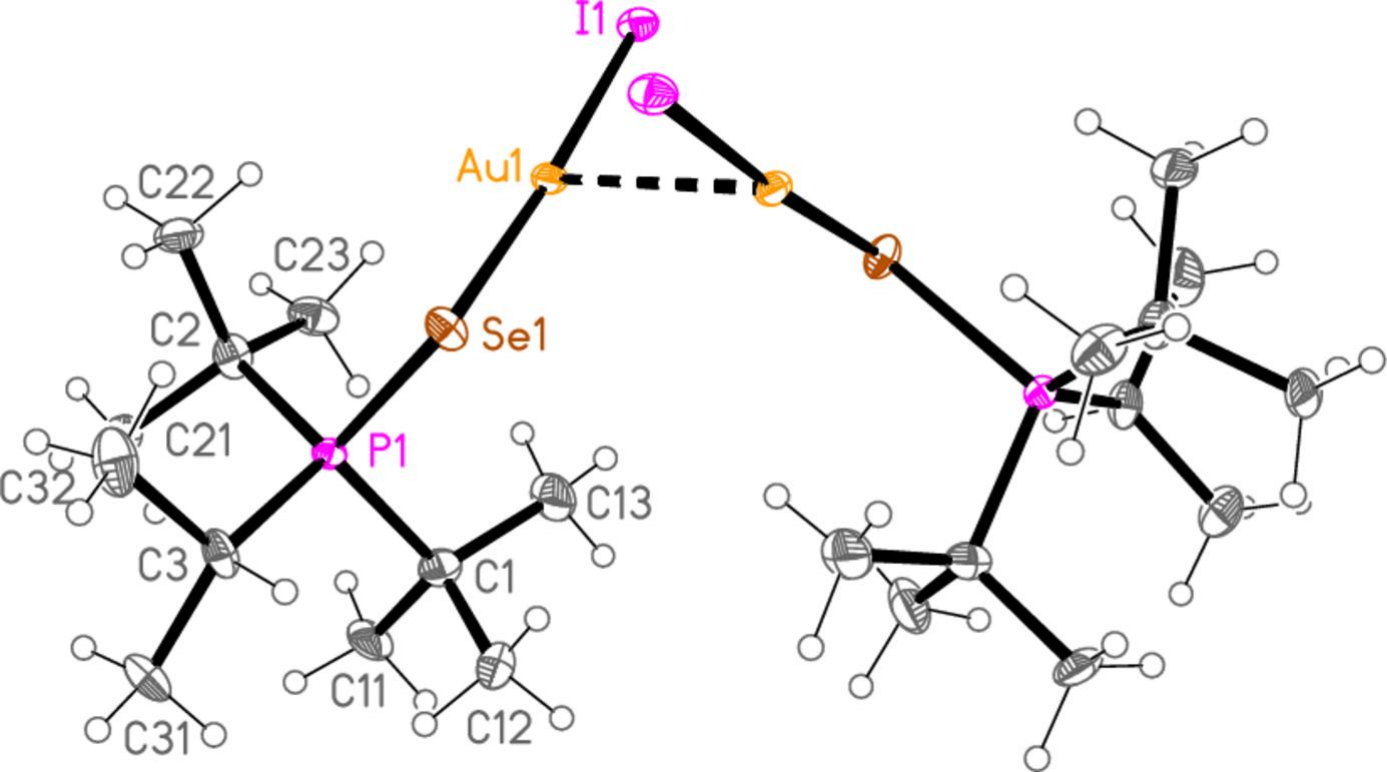
The dimer of compound **7c**; the thick dashed line indicates the aurophilic contact.

**Figure 26:**
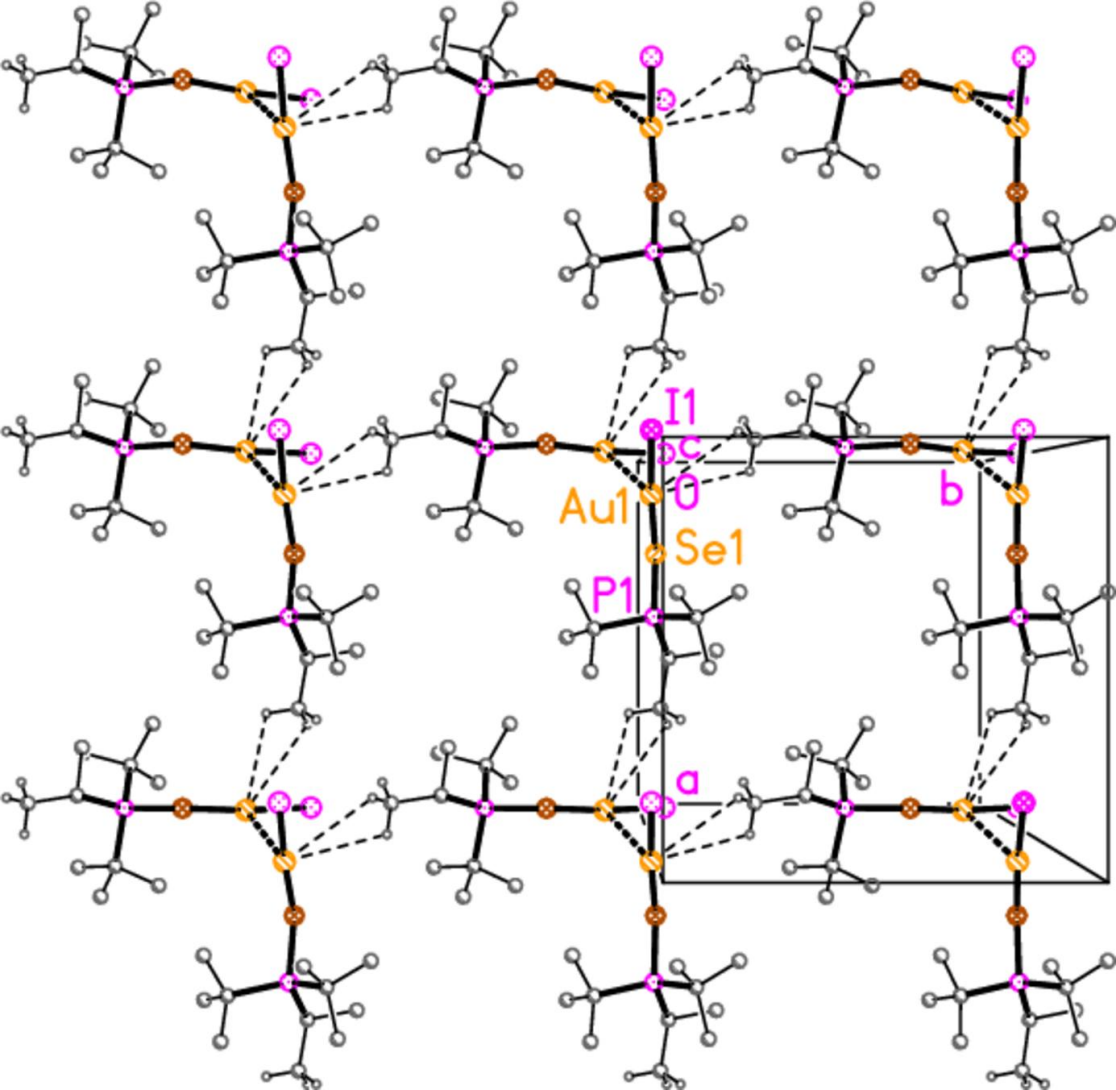
Packing diagram of the **7c** dimers, viewed parallel to the *c* axis in the region *z* ≃ 0.25. The dashed lines indicate borderline H⋯Au contacts.

*Special features*: The *t*-butyl groups of **3a** are rotationally disordered, whereby the smaller components have occupation factors 0.147 (8) at C1 and 0.178 (9) at C2. Appropriate restraints (SAME, SADI, SIMU) were applied to improve refinement stability, but the dimensions of disordered groups (and especially of the smaller components) should be inter­preted with caution. Associated with the disorder, the *U* values for **3a** are generally higher than for the other structures, so that the ellipsoids in Fig. 2 ▸ are drawn with 30% probability levels. Compounds **4a** and **7c** crystallize by chance in Sohncke space groups; the structures were refined as inversion twins, with the relative volumes of the smaller components refining to 0.493 (8) and 0.086 (9) respectively. Compound **4b** was refined as a pseudo-merohedric twin (with twin matrix 1 0 0 / 0 −1 0 / 0 0 −1); the relative volume of the smaller component refined to 0.2150 (11). Compound **6c** was refined in a non-standard monoclinic setting, with β slightly less than 90°, to facilitate comparison with the isotypic **6b**, which has β slightly greater than 90°; coordinates for both structures are then closely similar. The data for compound **7c** are only of moderate quality; eight badly-fitting reflections were omitted, and the displacement factors of the carbon atoms were restrained to be approximately isotropic (command ‘ISOR $C 0.01’).

The structures of **2a** and **2b** appear to be isotypic to each other and to **3a** and **3b**, but were severely disordered, by rotation or exchange (or both) of the alkyl groups. They are not further discussed here.

## Supplementary Material

Crystal structure: contains datablock(s) 1a, 3a, 4a, 5a, 6a, 7a, 1b, 3b, 4b, 5b, 6b, 7b, 8b, 2c, 6c, 7c, global. DOI: 10.1107/S2056989023010459/yz2044sup1.cif

Structure factors: contains datablock(s) 1a. DOI: 10.1107/S2056989023010459/yz20441asup2.hkl

Structure factors: contains datablock(s) 3a. DOI: 10.1107/S2056989023010459/yz20443asup3.hkl

Structure factors: contains datablock(s) 4a. DOI: 10.1107/S2056989023010459/yz20444asup4.hkl

Structure factors: contains datablock(s) 5a. DOI: 10.1107/S2056989023010459/yz20445asup5.hkl

Structure factors: contains datablock(s) 6a. DOI: 10.1107/S2056989023010459/yz20446asup6.hkl

Structure factors: contains datablock(s) 7a. DOI: 10.1107/S2056989023010459/yz20447asup7.hkl

Structure factors: contains datablock(s) 1b. DOI: 10.1107/S2056989023010459/yz20441bsup8.hkl

Structure factors: contains datablock(s) 3b. DOI: 10.1107/S2056989023010459/yz20443bsup9.hkl

Structure factors: contains datablock(s) 4b. DOI: 10.1107/S2056989023010459/yz20444bsup10.hkl

Structure factors: contains datablock(s) 5b. DOI: 10.1107/S2056989023010459/yz20445bsup11.hkl

Structure factors: contains datablock(s) 6b. DOI: 10.1107/S2056989023010459/yz20446bsup12.hkl

Structure factors: contains datablock(s) 7b. DOI: 10.1107/S2056989023010459/yz20447bsup13.hkl

Structure factors: contains datablock(s) 8b. DOI: 10.1107/S2056989023010459/yz20448bsup14.hkl

Structure factors: contains datablock(s) 2c. DOI: 10.1107/S2056989023010459/yz20442csup15.hkl

Structure factors: contains datablock(s) 6c. DOI: 10.1107/S2056989023010459/yz20446csup16.hkl

Structure factors: contains datablock(s) 7c. DOI: 10.1107/S2056989023010459/yz20447csup17.hkl

CCDC references: 2156388, 2156389, 2156780, 2156783, 2156784, 2156785, 2156875, 2156877, 2156887, 2156888, 2156890, 2314467, 2314468, 2314469, 2314470, 2314471

Additional supporting information:  crystallographic information; 3D view; checkCIF report

## Figures and Tables

**Figure 3:**
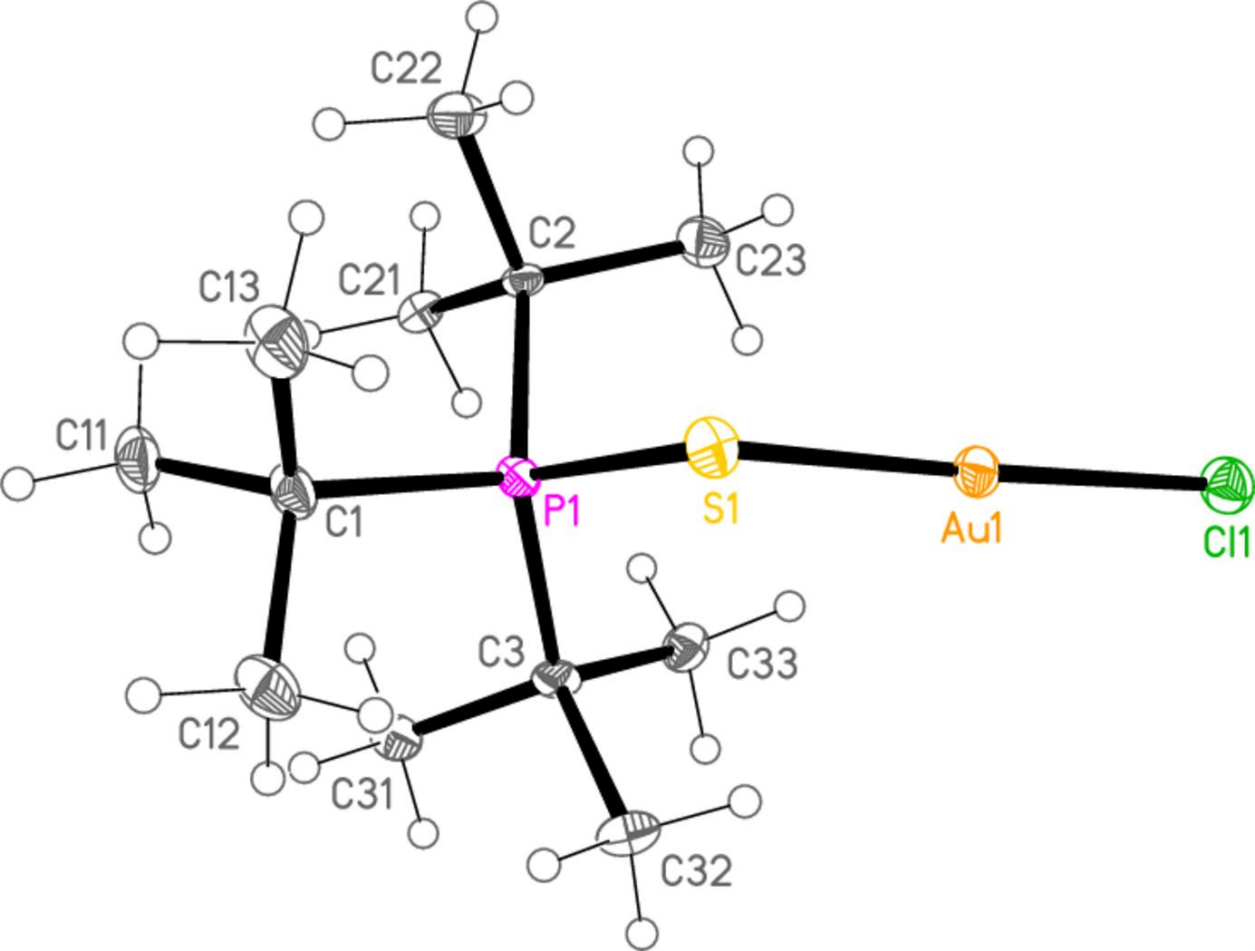
The structure of compound **4a** in the crystal. Ellipsoids represent 50% probability levels.

**Figure 4:**
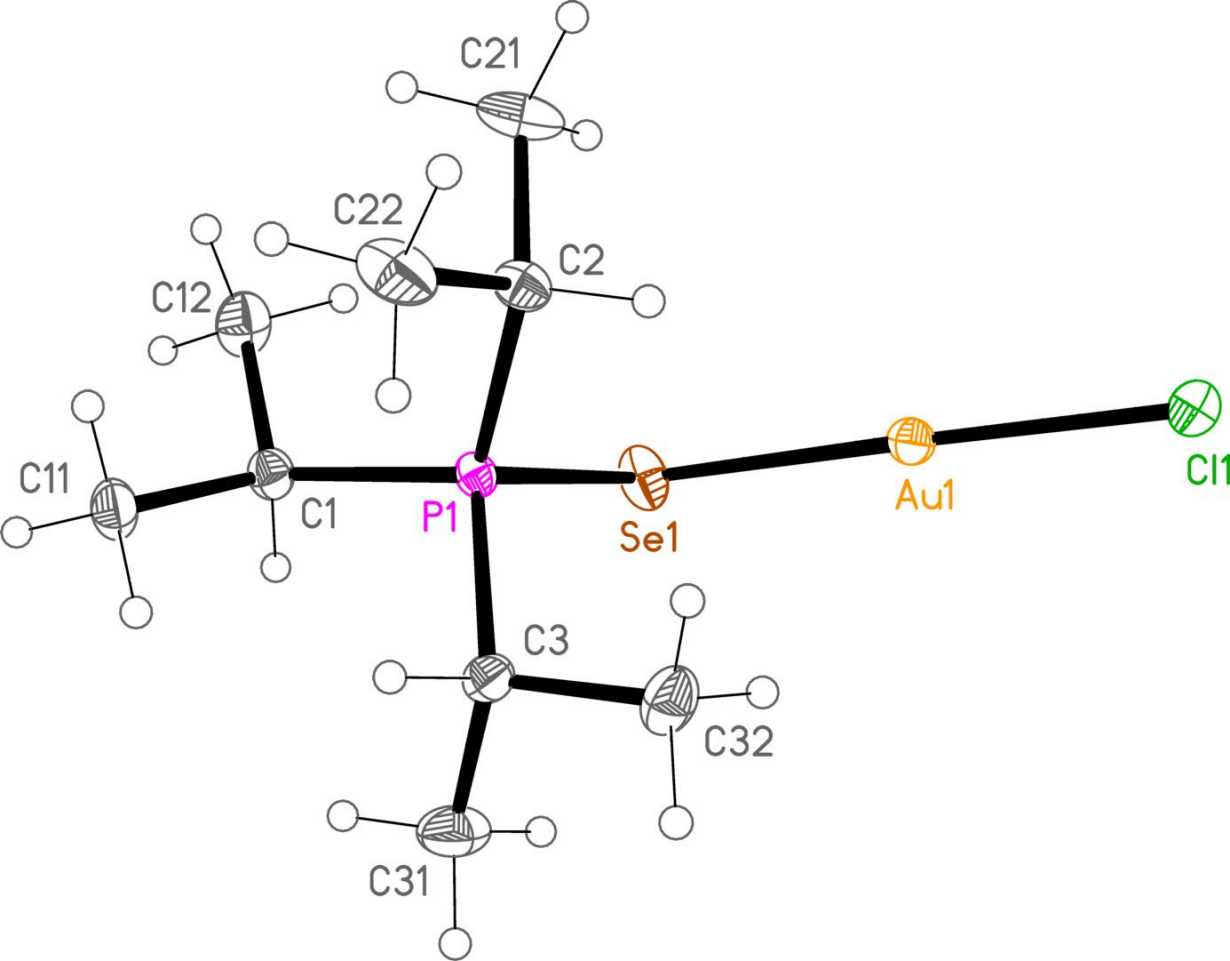
The structure of compound **5a** in the crystal. Ellipsoids represent 50% probability levels.

**Figure 5:**
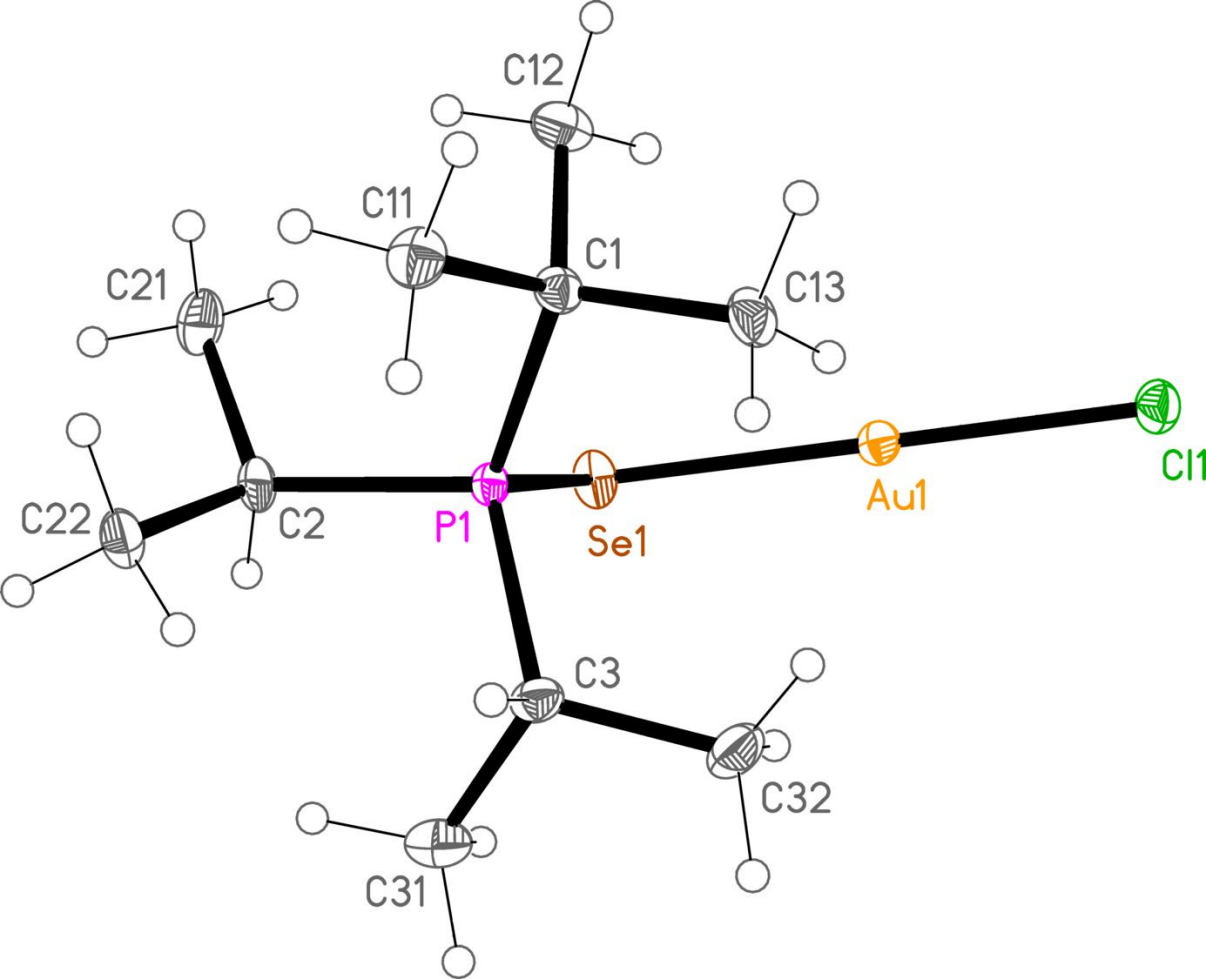
The structure of compound **6a** in the crystal. Ellipsoids represent 50% probability levels.

**Figure 6:**
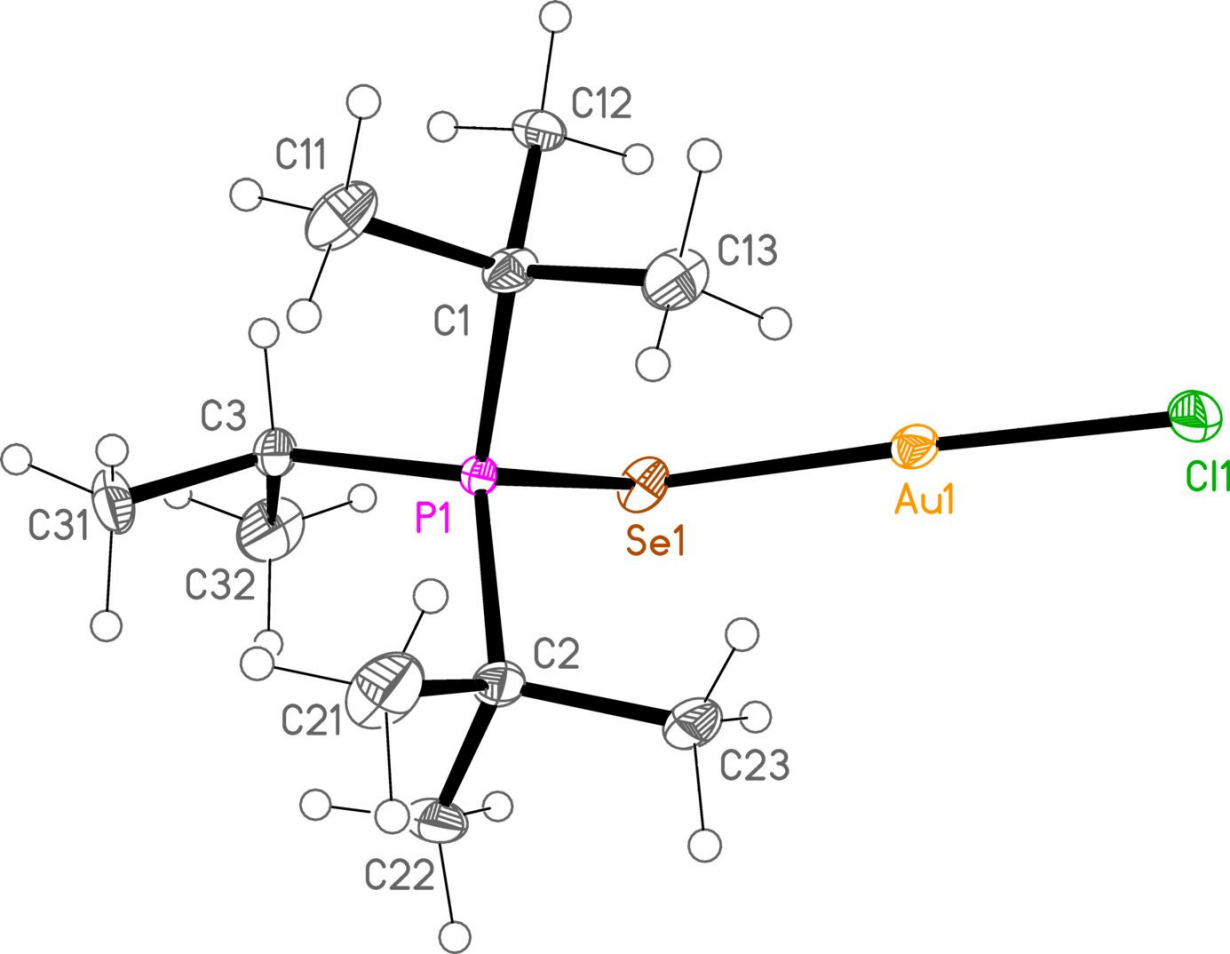
The structure of compound **7a** in the crystal. Ellipsoids represent 50% probability levels.

**Figure 7:**
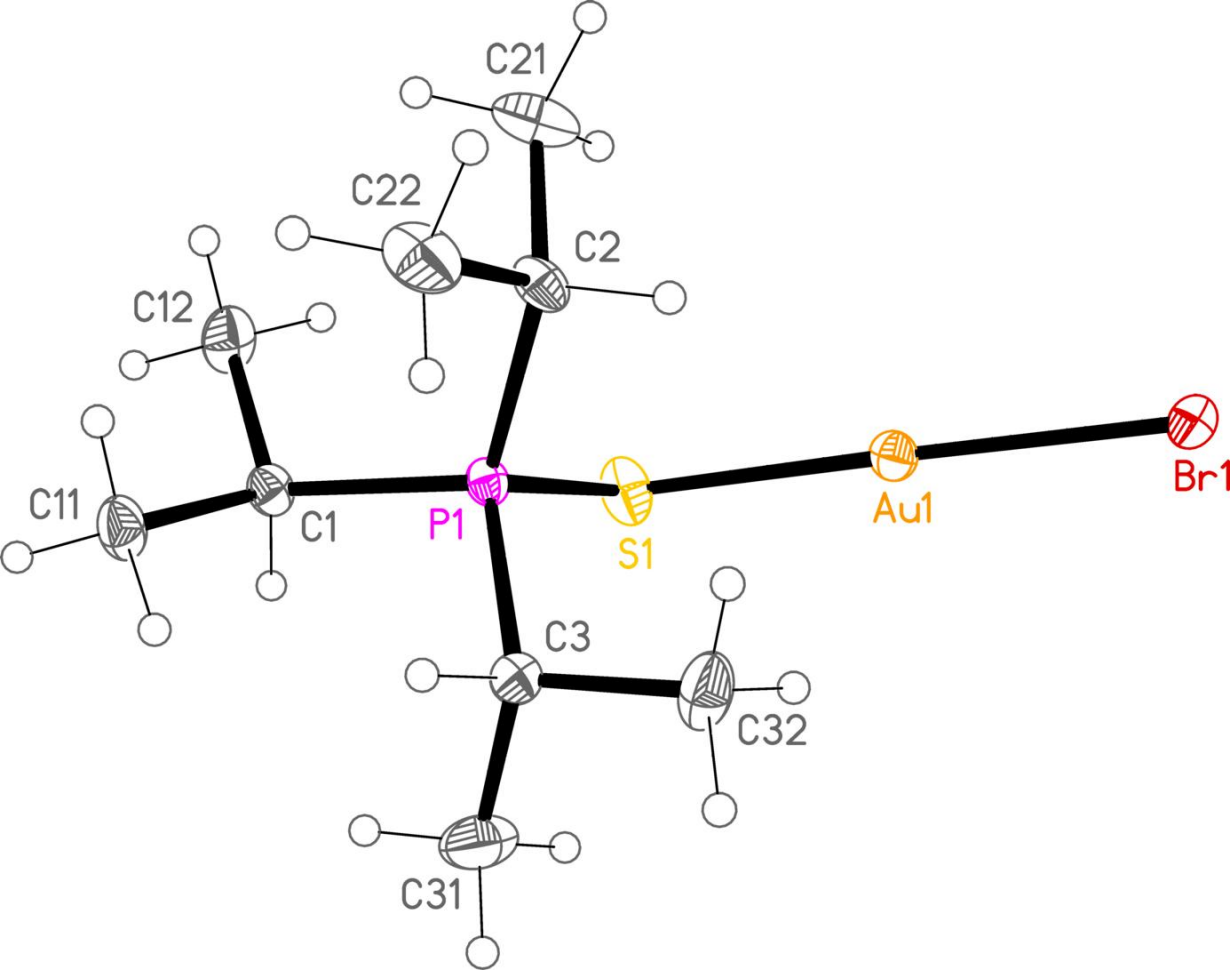
The structure of compound **1b** in the crystal. Ellipsoids represent 50% probability levels.

**Figure 8:**
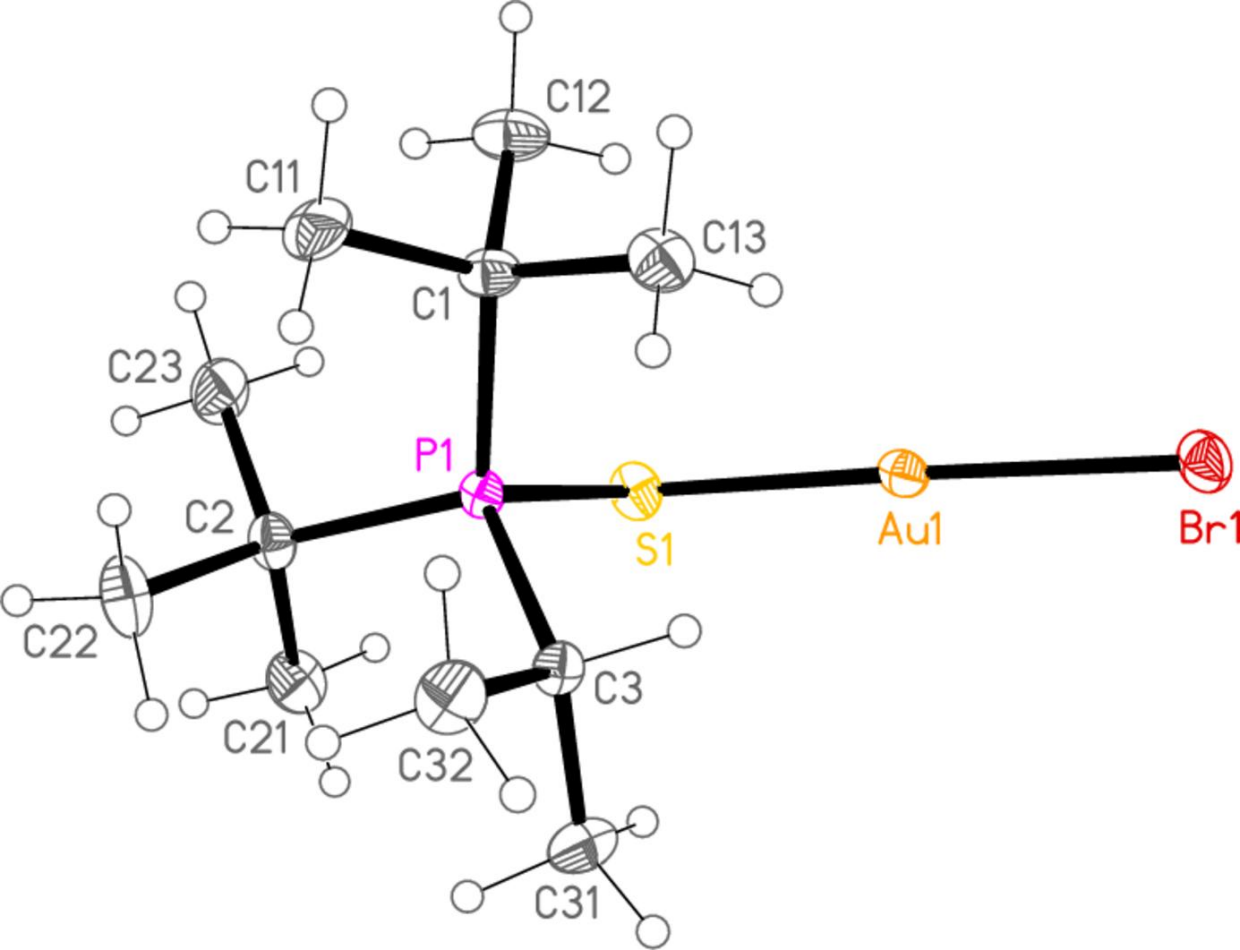
The structure of compound **3b** in the crystal. Ellipsoids represent 50% probability levels.

**Figure 9:**
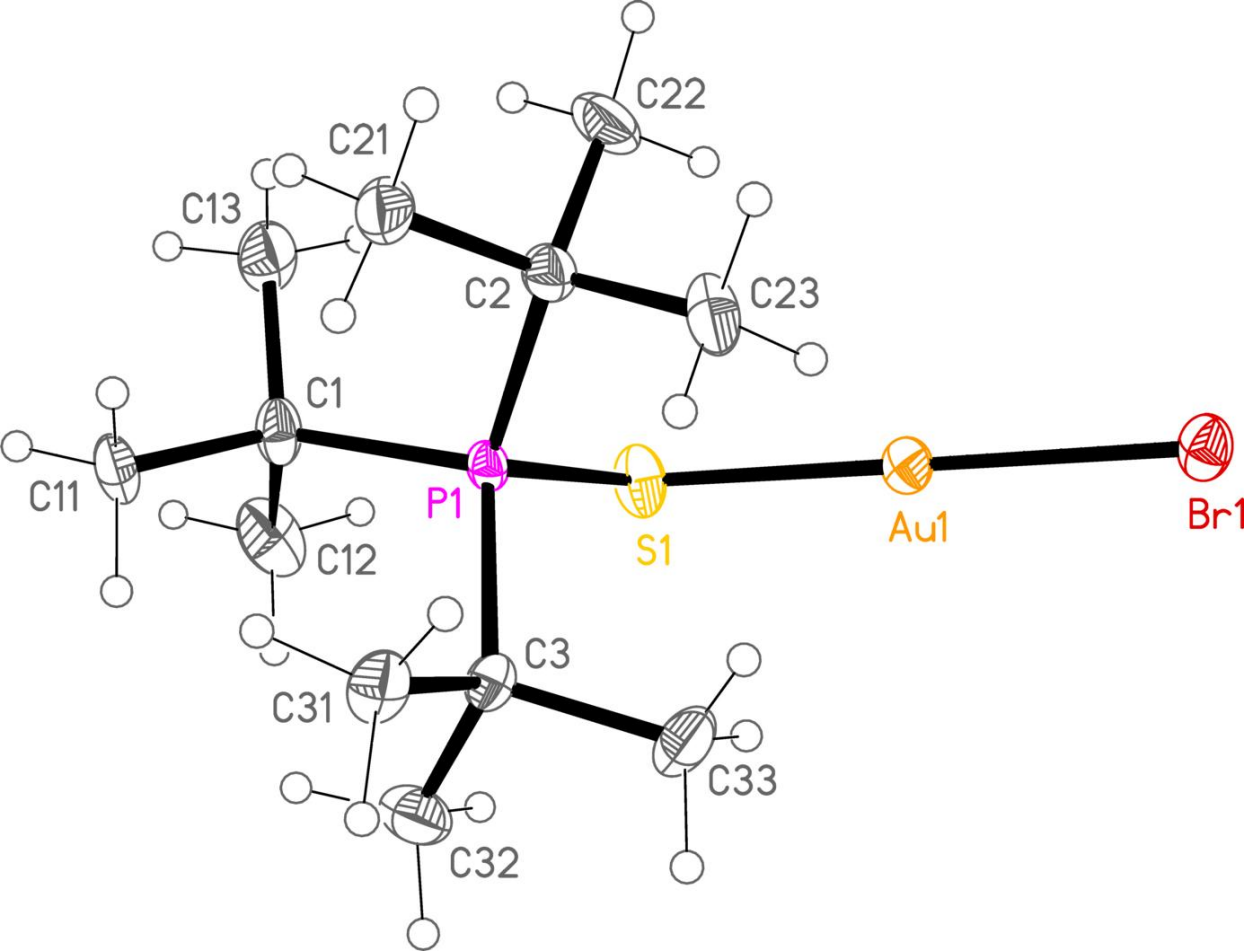
The structure of compound **4b** in the crystal. Ellipsoids represent 50% probability levels.

**Figure 10:**
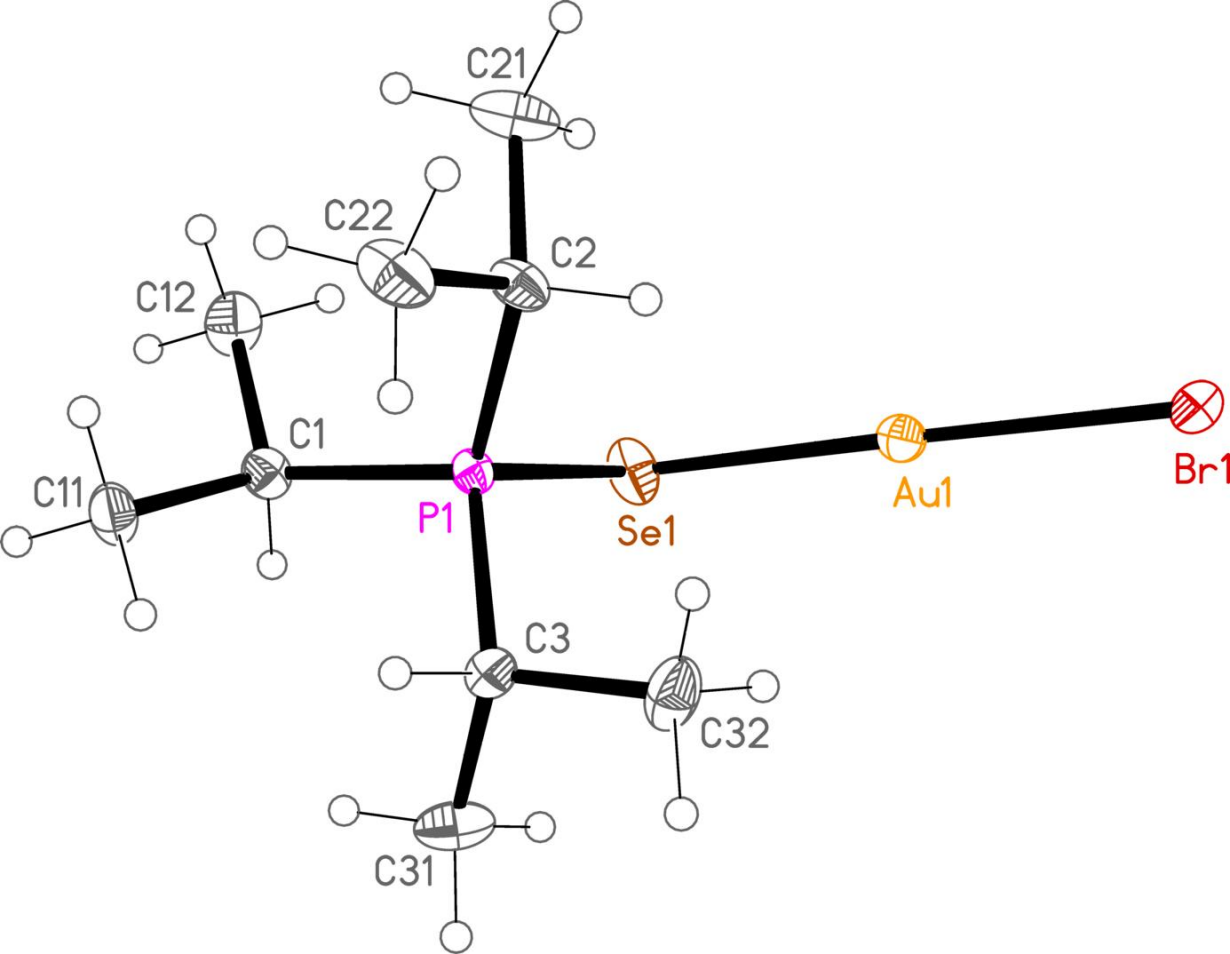
The structure of compound **5b** in the crystal. Ellipsoids represent 50% probability levels.

**Figure 11:**
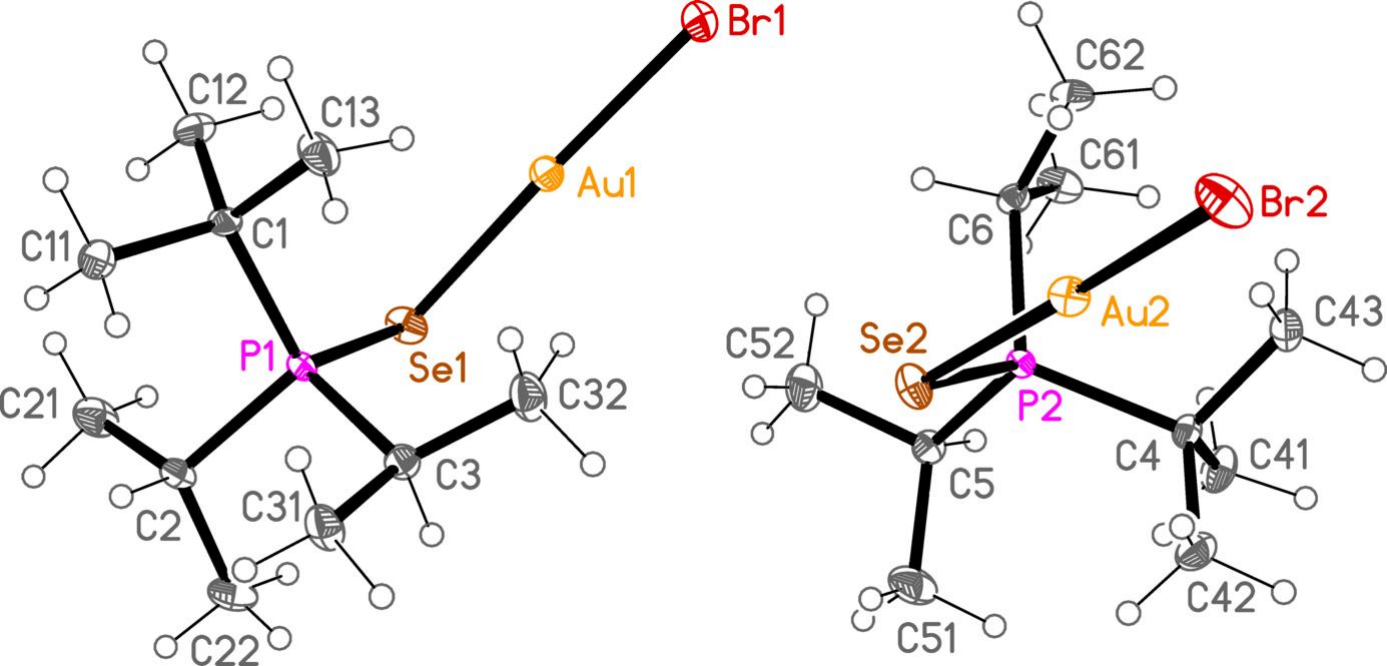
The structure of compound **6b** in the crystal. Ellipsoids represent 50% probability levels.

**Figure 12:**
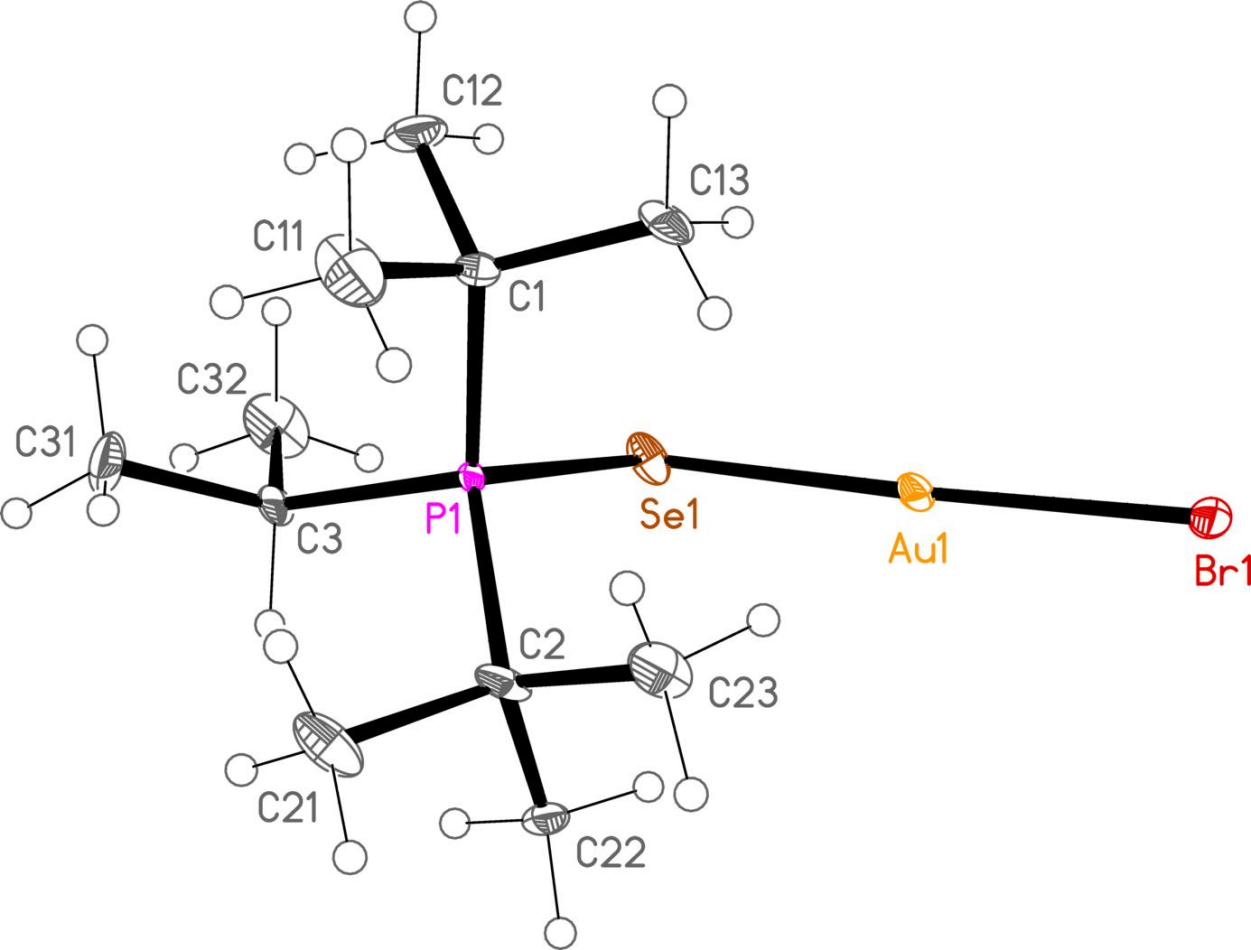
The structure of compound **7b** in the crystal. Ellipsoids represent 50% probability levels.

**Figure 13:**
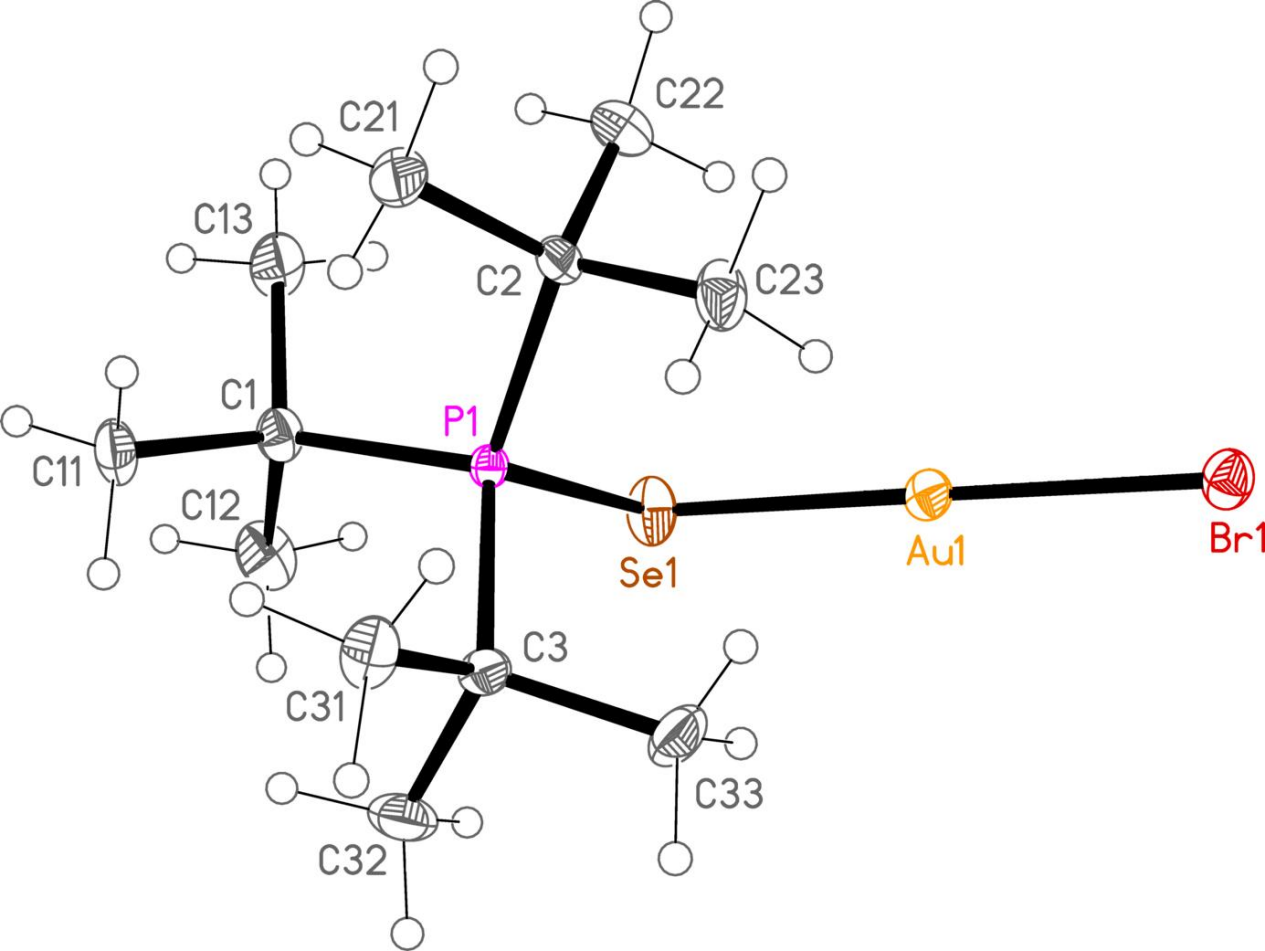
The structure of compound **8b** in the crystal. Ellipsoids represent 50% probability levels.

**Figure 14:**
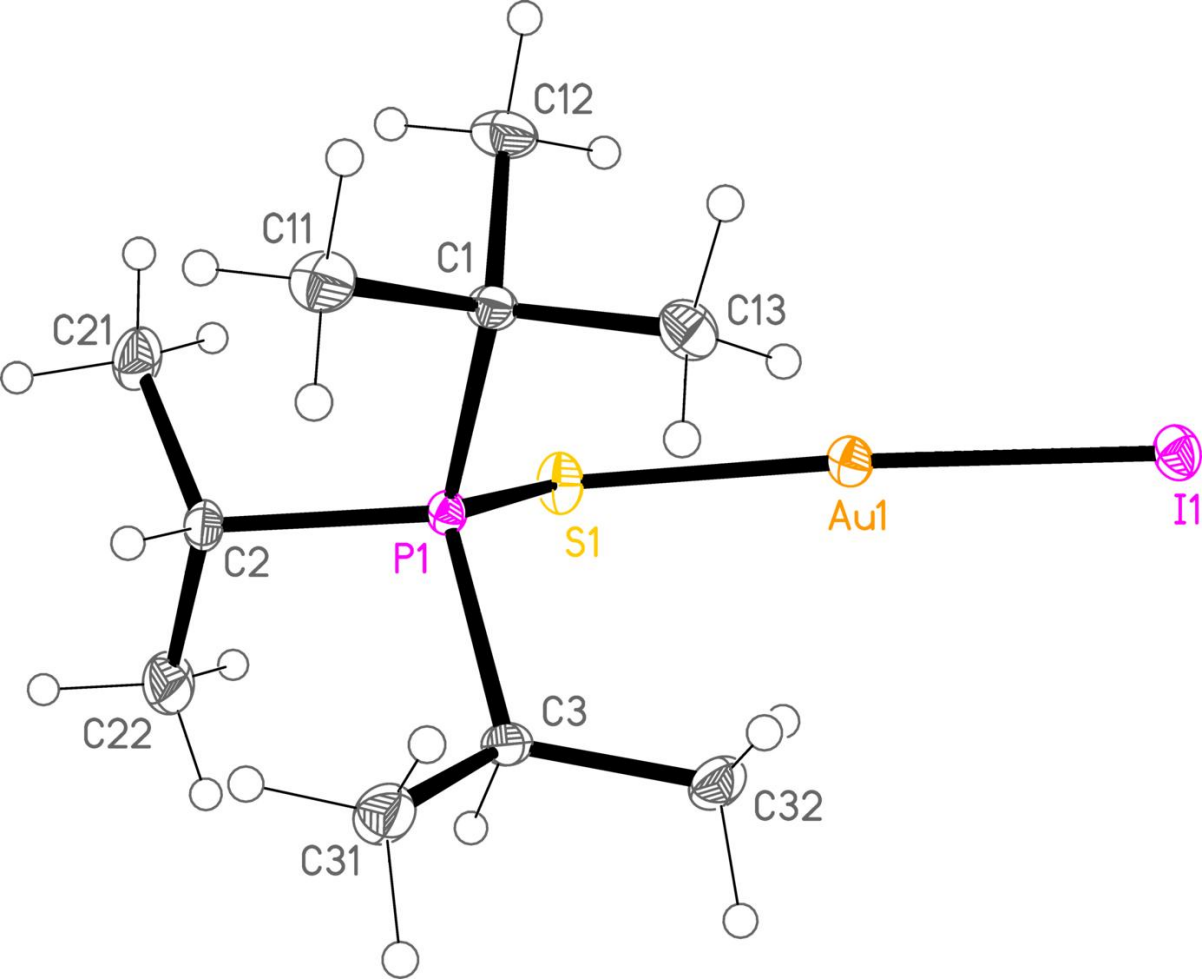
The structure of compound **2c** in the crystal. Ellipsoids represent 50% probability levels.

**Figure 15:**
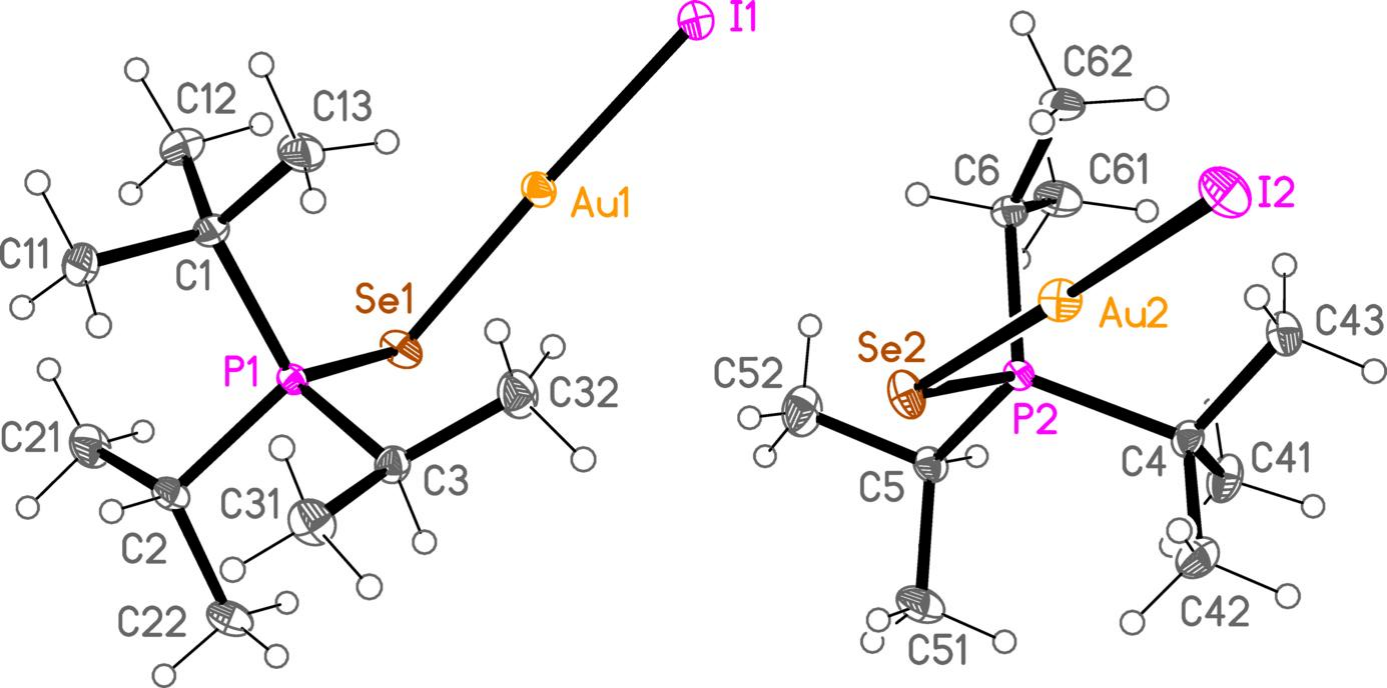
The structure of compound **6c** in the crystal. Ellipsoids represent 50% probability levels.

**Figure 16:**
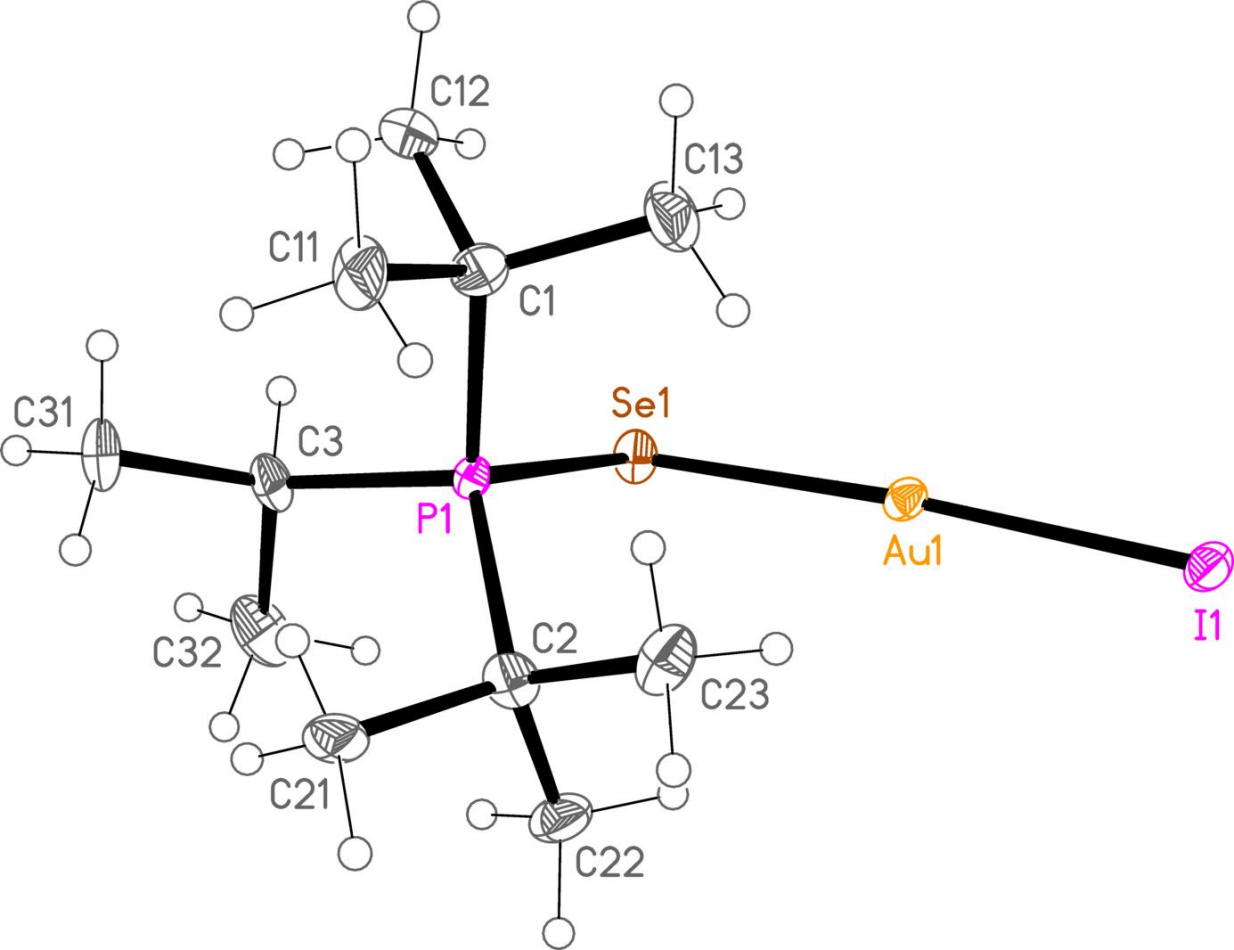
The structure of compound **7c** in the crystal. Ellipsoids represent 50% probability levels.

**Table 1:** Selected geometric parameters (Å, °) for **1a** [Chem scheme1]

Au1—S1	2.2711 (5)	P1—S1	2.0332 (6)
Au1—Cl1	2.2820 (5)		
			
S1—Au1—Cl1	175.217 (16)	C3—P1—S1	112.67 (6)
C3—P1—C1	106.34 (8)	C1—P1—S1	104.69 (6)
C3—P1—C2	107.41 (9)	C2—P1—S1	110.96 (6)
C1—P1—C2	114.79 (9)	P1—S1—Au1	104.56 (2)
			
C3—P1—S1—Au1	−71.54 (7)	C2—P1—S1—Au1	48.95 (7)
C1—P1—S1—Au1	173.32 (6)		

**Table 2:** Selected geometric parameters (Å, °) for **3a** [Chem scheme1]

Au1—S1	2.2674 (8)	P1—S1	2.0351 (11)
Au1—Cl1	2.2762 (9)		
			
S1—Au1—Cl1	174.71 (2)	C3—P1—S1	108.67 (10)
C3—P1—C2	113.13 (12)	C2—P1—S1	103.85 (9)
C3—P1—C1	107.61 (12)	C1—P1—S1	109.91 (9)
C2—P1—C1	113.55 (13)	P1—S1—Au1	107.40 (4)
			
C3—P1—S1—Au1	−51.44 (10)	C1—P1—S1—Au1	66.07 (10)
C2—P1—S1—Au1	−172.13 (9)		

**Table 3:** Selected geometric parameters (Å, °) for **4a** [Chem scheme1]

Au1—S1	2.2692 (10)	P1—S1	2.0482 (12)
Au1—Cl1	2.2820 (8)		
			
S1—Au1—Cl1	177.70 (15)	C3—P1—S1	111.3 (3)
C3—P1—C1	111.1 (4)	C1—P1—S1	102.45 (12)
C3—P1—C2	111.7 (2)	C2—P1—S1	109.1 (3)
C1—P1—C2	110.8 (4)	P1—S1—Au1	105.86 (5)
			
C3—P1—S1—Au1	53.2 (3)	C2—P1—S1—Au1	−70.5 (3)
C1—P1—S1—Au1	172.0 (4)		

**Table 4:** Selected geometric parameters (Å, °) for **5a** [Chem scheme1]

Au1—Cl1	2.2862 (8)	P1—Se1	2.1868 (8)
Au1—Se1	2.3745 (3)		
			
Cl1—Au1—Se1	174.03 (2)	C1—P1—Se1	104.38 (10)
C1—P1—C3	106.51 (14)	C3—P1—Se1	112.30 (10)
C1—P1—C2	115.25 (14)	C2—P1—Se1	110.93 (10)
C3—P1—C2	107.49 (14)	P1—Se1—Au1	102.89 (2)
			
C1—P1—Se1—Au1	172.39 (10)	C2—P1—Se1—Au1	47.67 (11)
C3—P1—Se1—Au1	−72.63 (11)		

**Table 5:** Selected geometric parameters (Å, °) for **6a** [Chem scheme1]

Au1—Cl1	2.2877 (7)	P1—Se1	2.1947 (7)
Au1—Se1	2.3696 (3)		
			
Cl1—Au1—Se1	176.13 (2)	C3—P1—Se1	110.99 (10)
C3—P1—C2	105.85 (13)	C2—P1—Se1	102.23 (10)
C3—P1—C1	110.98 (13)	C1—P1—Se1	114.09 (9)
C2—P1—C1	112.11 (13)	P1—Se1—Au1	103.21 (2)
			
C3—P1—Se1—Au1	−74.90 (10)	C1—P1—Se1—Au1	51.36 (10)
C2—P1—Se1—Au1	172.61 (10)		

**Table 6:** Selected geometric parameters (Å, °) for **7a** [Chem scheme1]

Au1—Cl1	2.2898 (6)	P1—Se1	2.2027 (6)
Au1—Se1	2.3740 (3)		
			
Cl1—Au1—Se1	174.465 (18)	C3—P1—Se1	103.38 (9)
C3—P1—C2	112.52 (11)	C2—P1—Se1	109.00 (8)
C3—P1—C1	105.33 (11)	C1—P1—Se1	111.54 (8)
C2—P1—C1	114.50 (12)	P1—Se1—Au1	106.911 (18)
			
C3—P1—Se1—Au1	166.02 (8)	C1—P1—Se1—Au1	53.31 (8)
C2—P1—Se1—Au1	−74.09 (9)		

**Table 7:** Selected geometric parameters (Å, °) for **1b** [Chem scheme1]

Au1—S1	2.2763 (6)	P1—S1	2.0325 (8)
Au1—Br1	2.3963 (3)		
			
S1—Au1—Br1	175.134 (15)	C3—P1—S1	112.94 (8)
C3—P1—C2	107.21 (10)	C2—P1—S1	111.22 (8)
C3—P1—C1	106.45 (10)	C1—P1—S1	104.27 (8)
C2—P1—C1	114.79 (11)	P1—S1—Au1	104.61 (3)
			
C3—P1—S1—Au1	−70.35 (8)	C1—P1—S1—Au1	174.50 (8)
C2—P1—S1—Au1	50.25 (9)		

**Table 8:** Selected geometric parameters (Å, °) for **3b** [Chem scheme1]

Au1—S1	2.2731 (5)	S1—P1	2.0333 (7)
Au1—Br1	2.3896 (3)		
			
S1—Au1—Br1	174.382 (13)	C2—P1—C1	113.42 (9)
P1—S1—Au1	106.84 (3)	C3—P1—S1	108.72 (6)
C3—P1—C2	113.35 (9)	C2—P1—S1	103.79 (6)
C3—P1—C1	107.53 (9)	C1—P1—S1	109.91 (7)
			
Au1—S1—P1—C3	−50.79 (7)	Au1—S1—P1—C1	66.65 (7)
Au1—S1—P1—C2	−171.73 (7)		

**Table 9:** Selected geometric parameters (Å, °) for **4b** [Chem scheme1]

Au1—S1	2.2791 (19)	P1—S1	2.043 (2)
Au1—Br1	2.3925 (8)		
			
S1—Au1—Br1	176.04 (5)	C2—P1—S1	111.2 (2)
C2—P1—C1	111.3 (3)	C1—P1—S1	102.7 (2)
C2—P1—C3	110.5 (3)	C3—P1—S1	109.9 (2)
C1—P1—C3	111.0 (3)	P1—S1—Au1	107.77 (9)
			
C2—P1—S1—Au1	48.4 (3)	C3—P1—S1—Au1	−74.3 (3)
C1—P1—S1—Au1	167.6 (3)		

**Table 10:** Selected geometric parameters (Å, °) for **5b** [Chem scheme1]

Au1—Se1	2.3779 (2)	P1—Se1	2.1860 (5)
Au1—Br1	2.4004 (2)		
			
Se1—Au1—Br1	173.957 (8)	C3—P1—Se1	112.54 (7)
C3—P1—C1	106.54 (9)	C1—P1—Se1	103.85 (6)
C3—P1—C2	107.42 (9)	C2—P1—Se1	111.11 (6)
C1—P1—C2	115.41 (9)	P1—Se1—Au1	102.931 (14)
			
C3—P1—Se1—Au1	−71.46 (7)	C2—P1—Se1—Au1	49.05 (7)
C1—P1—Se1—Au1	173.72 (7)		

**Table 11:** Selected geometric parameters (Å, °) for **6b** [Chem scheme1]

Au1—Se1	2.3872 (3)	Au2—Se2	2.3848 (3)
Au1—Br1	2.4036 (3)	Au2—Br2	2.4040 (3)
Se1—P1	2.1911 (7)	Se2—P2	2.1875 (7)
			
Se1—Au1—Br1	177.083 (11)	C2—P1—Se1	105.63 (9)
P1—Se1—Au1	101.29 (2)	C3—P1—Se1	109.60 (9)
C2—P1—C3	104.54 (12)	C1—P1—Se1	113.64 (9)
C2—P1—C1	108.77 (12)	Se2—Au2—Br2	174.050 (11)
C3—P1—C1	113.93 (13)	P2—Se2—Au2	103.59 (2)
			
Au1—Se1—P1—C2	174.51 (9)	Au1—Se1—P1—C1	55.36 (9)
Au1—Se1—P1—C3	−73.38 (10)		

**Table 12:** Selected geometric parameters (Å, °) for **7b** [Chem scheme1]

Au1—Se1	2.3761 (5)	P1—Se1	2.2013 (11)
Au1—Br1	2.4009 (5)		
			
Se1—Au1—Br1	175.696 (17)	C3—P1—Se1	103.58 (16)
C3—P1—C1	105.4 (2)	C1—P1—Se1	111.39 (16)
C3—P1—C2	112.6 (2)	C2—P1—Se1	108.73 (16)
C1—P1—C2	114.5 (2)	P1—Se1—Au1	105.58 (3)
			
C3—P1—Se1—Au1	165.57 (15)	C2—P1—Se1—Au1	−74.43 (17)
C1—P1—Se1—Au1	52.70 (15)		

**Table 13:** Selected geometric parameters (Å, °) for **8b** [Chem scheme1]

Au1—Se1	2.3805 (3)	P1—Se1	2.2008 (7)
Au1—Br1	2.3961 (3)		
			
Se1—Au1—Br1	175.936 (11)	C3—P1—Se1	109.31 (9)
C3—P1—C2	111.30 (13)	C2—P1—Se1	111.01 (10)
C3—P1—C1	111.06 (13)	C1—P1—Se1	102.43 (9)
C2—P1—C1	111.39 (13)	P1—Se1—Au1	104.44 (2)
			
C3—P1—Se1—Au1	−74.10 (10)	C1—P1—Se1—Au1	168.05 (9)
C2—P1—Se1—Au1	49.06 (10)		

**Table 14:** Selected geometric parameters (Å, °) for (2c)

Au1—S1	2.2959 (6)	S1—P1	2.0322 (8)
Au1—I1	2.5437 (2)		
			
S1—Au1—I1	173.747 (16)	C3—P1—C1	113.74 (11)
P1—S1—Au1	106.08 (3)	C2—P1—S1	105.26 (8)
C2—P1—C3	104.91 (11)	C3—P1—S1	110.25 (8)
C2—P1—C1	109.11 (10)	C1—P1—S1	112.90 (8)
			
Au1—S1—P1—C2	178.84 (8)	Au1—S1—P1—C1	59.91 (8)
Au1—S1—P1—C3	−68.53 (8)		

**Table 15:** Selected geometric parameters (Å, °) for **6c** [Chem scheme1]

Au1—Se1	2.4040 (3)	Au2—Se2	2.4002 (3)
Au1—I1	2.5508 (2)	Au2—I2	2.5503 (2)
Se1—P1	2.1938 (8)	Se2—P2	2.1890 (8)
			
Se1—Au1—I1	177.832 (10)	Se2—Au2—I2	175.009 (10)
P1—Se1—Au1	100.57 (2)	P2—Se2—Au2	103.30 (2)
C3—P1—C2	104.65 (13)	C5—P2—C6	104.86 (13)
C3—P1—C1	113.83 (14)	C5—P2—C4	109.11 (13)
C2—P1—C1	108.98 (13)	C6—P2—C4	113.70 (13)
C3—P1—Se1	109.71 (10)	C5—P2—Se2	105.35 (10)
C2—P1—Se1	105.13 (10)	C6—P2—Se2	109.60 (10)
C1—P1—Se1	113.77 (10)	C4—P2—Se2	113.51 (9)
			
Au1—Se1—P1—C3	−72.96 (10)	Au2—Se2—P2—C5	169.01 (9)
Au1—Se1—P1—C2	175.01 (9)	Au2—Se2—P2—C6	−78.65 (10)
Au1—Se1—P1—C1	55.84 (10)	Au2—Se2—P2—C4	49.69 (10)

**Table 16:** Selected geometric parameters (Å, °) for **7c** [Chem scheme1]

Au1—Se1	2.4014 (11)	Au1—Au1^i^	3.0914 (8)
Au1—I1	2.5509 (8)	P1—Se1	2.198 (3)
			
Se1—Au1—I1	176.56 (4)	C2—P1—C1	113.0 (5)
Se1—Au1—Au1^i^	78.63 (3)	C3—P1—Se1	100.9 (4)
I1—Au1—Au1^i^	103.36 (2)	C2—P1—Se1	112.8 (4)
C3—P1—C2	112.3 (5)	C1—P1—Se1	109.3 (4)
C3—P1—C1	107.7 (5)	P1—Se1—Au1	103.94 (8)
			
C3—P1—Se1—Au1	−169.6 (4)	I1—Au1—Au1^i^—I1^i^	117.89 (4)
C2—P1—Se1—Au1	−49.5 (4)	Se1—Au1—Au1^i^—Se1^i^	123.62 (6)
C1—P1—Se1—Au1	77.1 (4)	I1—Au1—Au1^i^—Se1^i^	−59.25 (3)

Symmetry code: (i) 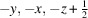 .

**Table 17:** Hydrogen-bond geometry (Å, °) for **1a** [Chem scheme1]

*D*—H⋯*A*	*D*—H	H⋯*A*	*D*⋯*A*	*D*—H⋯*A*
C32—H32*C*⋯Au1	0.98	2.83	3.615 (2)	138
C3—H3⋯Cl1^i^	1.00	2.77	3.6734 (19)	151
C12—H12*B*⋯Au1^ii^	0.98	3.02	3.8290 (19)	141
C1—H1⋯S1^iii^	1.00	2.99	3.9487 (19)	162

Symmetry codes: (i) 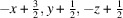 ; (ii) 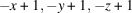 ; (iii) 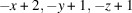 .

**Table 18:** Hydrogen-bond geometry (Å, °) for **3a** [Chem scheme1]

*D*—H⋯*A*	*D*—H	H⋯*A*	*D*⋯*A*	*D*—H⋯*A*
C13—H13*A*⋯Au1	0.98	2.71	3.611 (3)	154
C12—H12*C*⋯S1	0.98	2.78	3.291 (4)	113
C21—H21*B*⋯S1	0.98	2.72	3.211 (5)	111

**Table 19:** Hydrogen-bond geometry (Å, °) for **4a** [Chem scheme1]

*D*—H⋯*A*	*D*—H	H⋯*A*	*D*⋯*A*	*D*—H⋯*A*
C11—H11*A*⋯Cl1^i^	0.98	2.80	3.765 (4)	170
C21—H21*A*⋯Cl1^ii^	0.98	2.86	3.575 (7)	130
C23—H23*C*⋯Au1	0.98	2.76	3.352 (4)	120
C33—H33*C*⋯Au1	0.98	2.73	3.599 (4)	148
C13—H13*C*⋯S1	0.98	2.74	3.197 (5)	109
C32—H32*C*⋯S1	0.98	2.90	3.344 (6)	109

Symmetry codes: (i) 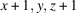 ; (ii) 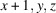 .

**Table 20:** Hydrogen-bond geometry (Å, °) for **5a** [Chem scheme1]

*D*—H⋯*A*	*D*—H	H⋯*A*	*D*⋯*A*	*D*—H⋯*A*
C32—H32*C*⋯Au1	0.98	2.91	3.700 (3)	138
C3—H3⋯Cl1^i^	1.00	2.81	3.715 (3)	151
C12—H12*B*⋯Au1^ii^	0.98	3.07	3.864 (3)	139
C1—H1⋯Se1^iii^	1.00	2.99	3.955 (3)	162

Symmetry codes: (i) 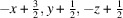 ; (ii) 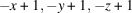 ; (iii) 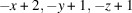 .

**Table 21:** Hydrogen-bond geometry (Å, °) for **6a** [Chem scheme1]

*D*—H⋯*A*	*D*—H	H⋯*A*	*D*⋯*A*	*D*—H⋯*A*
C13—H13*B*⋯Au1	0.98	2.73	3.630 (3)	154
C3—H3⋯Au1^i^	1.00	3.01	3.861 (3)	144
C3—H3⋯Cl1^i^	1.00	2.71	3.669 (3)	160
C2—H2⋯Se1^ii^	1.00	3.09	3.982 (3)	150
C13—H13*A*⋯Cl1^iii^	0.98	2.87	3.839 (3)	169
C22—H22*A*⋯Cl1^iv^	0.98	2.87	3.802 (3)	159

Symmetry codes: (i) 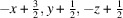 ; (ii) 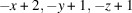 ; (iii) 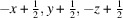 ; (iv) 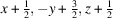 .

**Table 22:** Hydrogen-bond geometry (Å, °) for **7a** [Chem scheme1]

*D*—H⋯*A*	*D*—H	H⋯*A*	*D*⋯*A*	*D*—H⋯*A*
C13—H13*B*⋯Au1	0.98	2.80	3.712 (3)	155
C23—H23*B*⋯Au1	0.98	2.89	3.615 (3)	131
C32—H32*B*⋯Se1	0.98	2.62	3.228 (3)	120
C3—H3⋯Cl1^i^	1.00	2.68	3.599 (3)	154

Symmetry code: (i) 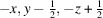 .

**Table 23:** Hydrogen-bond geometry (Å, °) for **1b** [Chem scheme1]

*D*—H⋯*A*	*D*—H	H⋯*A*	*D*⋯*A*	*D*—H⋯*A*
C3—H3⋯Br1^i^	1.00	2.87	3.756 (2)	149
C21—H21*C*⋯Br1^ii^	0.98	3.04	3.878 (3)	144
C32—H32*C*⋯Au1	0.98	2.83	3.611 (2)	137
C1—H1⋯S1^iii^	1.00	2.96	3.911 (2)	159
C12—H12*B*⋯S1	0.98	2.95	3.495 (2)	116
C21—H21*B*⋯S1	0.98	2.95	3.512 (3)	117

Symmetry codes: (i) 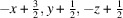 ; (ii) 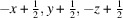 ; (iii) 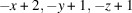 .

**Table 24:** Hydrogen-bond geometry (Å, °) for **3b** [Chem scheme1]

*D*—H⋯*A*	*D*—H	H⋯*A*	*D*⋯*A*	*D*—H⋯*A*
C13—H13*B*⋯Au1	0.98	2.75	3.633 (2)	151
C23—H23*A*⋯S1	0.98	2.99	3.515 (2)	114
C21—H21*C*⋯S1	0.98	2.70	3.216 (2)	114
C11—H11*C*⋯Br1^i^	0.98	3.12	4.019 (2)	154
C12—H12*B*⋯S1^ii^	0.98	2.94	3.603 (2)	126
C12—H12*C*⋯Br1^i^	0.98	3.12	4.039 (2)	158
C31—H31*C*⋯Br1^iii^	0.98	3.12	4.073 (2)	165
C23—H23*C*⋯Br1^iv^	0.98	3.15	4.072 (2)	158

Symmetry codes: (i) 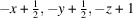 ; (ii) 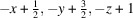 ; (iii) 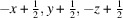 ; (iv) 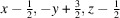 .

**Table 25:** Hydrogen-bond geometry (Å, °) for **4b** [Chem scheme1]

*D*—H⋯*A*	*D*—H	H⋯*A*	*D*⋯*A*	*D*—H⋯*A*
C13—H13*B*⋯Br1^i^	0.98	2.98	3.882 (8)	153
C32—H32*A*⋯Br1^ii^	0.98	3.00	3.936 (8)	161
C33—H33*B*⋯Au1	0.98	2.82	3.523 (8)	129
C23—H23*B*⋯Au1	0.98	2.66	3.541 (7)	150
C12—H12*C*⋯S1	0.98	2.63	3.169 (9)	115
C33—H33*B*⋯S1	0.98	2.94	3.487 (8)	116
C22—H22*C*⋯S1	0.98	2.91	3.377 (9)	110

Symmetry codes: (i) 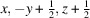 ; (ii) 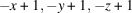 .

**Table 26:** Hydrogen-bond geometry (Å, °) for **5b** [Chem scheme1]

*D*—H⋯*A*	*D*—H	H⋯*A*	*D*⋯*A*	*D*—H⋯*A*
C3—H3⋯Br1^i^	1.00	2.90	3.792 (2)	149
C1—H1⋯Se1^ii^	1.00	2.97	3.9210 (19)	159
C12—H12*B*⋯Se1	0.98	2.99	3.568 (2)	119
C21—H21*B*⋯Se1	0.98	3.05	3.633 (2)	120

Symmetry codes: (i) 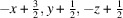 ; (ii) 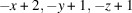 .

**Table 27:** Hydrogen-bond geometry (Å, °) for **6b** [Chem scheme1]

*D*—H⋯*A*	*D*—H	H⋯*A*	*D*⋯*A*	*D*—H⋯*A*
C13—H13*A*⋯Au1	0.98	2.84	3.719 (3)	149
C32—H32*B*⋯Au1	0.98	2.72	3.623 (3)	154
C43—H43*A*⋯Au2	0.98	2.87	3.744 (3)	148
C62—H62*B*⋯Au2	0.98	2.85	3.766 (3)	155
C5—H5⋯Br2^i^	1.00	2.79	3.702 (3)	152
C6—H6⋯Br1	1.00	2.96	3.906 (3)	157
C3—H3⋯Br2^ii^	1.00	3.08	3.850 (3)	134
C42—H42*A*⋯Br1^iii^	0.98	3.02	3.959 (3)	161
C2—H2⋯Au1^iv^	1.00	2.98	3.929 (3)	158

Symmetry codes: (i) 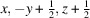 ; (ii) 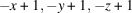 ; (iii) 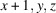 ; (iv) 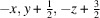 .

**Table 28:** Hydrogen-bond geometry (Å, °) for **7b** [Chem scheme1]

*D*—H⋯*A*	*D*—H	H⋯*A*	*D*⋯*A*	*D*—H⋯*A*
C13—H13*B*⋯Au1	0.98	2.74	3.650 (6)	155
C23—H23*B*⋯Au1	0.98	2.90	3.592 (5)	128
C32—H32*B*⋯Se1	0.98	2.64	3.233 (6)	120
C3—H3⋯Br1^i^	1.00	2.76	3.677 (4)	153
C13—H13*A*⋯Br1^ii^	0.98	2.97	3.928 (5)	167
C21—H21*B*⋯Br1^iii^	0.98	3.07	3.697 (6)	123
C22—H22*C*⋯Br1^iv^	0.98	3.10	3.921 (5)	142

Symmetry codes: (i) 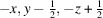 ; (ii) 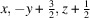 ; (iii) 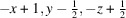 ; (iv) 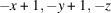 .

**Table 29:** Hydrogen-bond geometry (Å, °) for **8b** [Chem scheme1]

*D*—H⋯*A*	*D*—H	H⋯*A*	*D*⋯*A*	*D*—H⋯*A*
C13—H13*B*⋯Br1^i^	0.98	3.03	3.946 (3)	156
C32—H32*A*⋯Br1^ii^	0.98	2.98	3.905 (3)	159
C33—H33*B*⋯Au1	0.98	2.87	3.573 (3)	130
C23—H23*B*⋯Au1	0.98	2.68	3.582 (3)	153
C12—H12*C*⋯Se1	0.98	2.71	3.251 (3)	115
C33—H33*B*⋯Se1	0.98	3.00	3.574 (3)	119
C22—H22*C*⋯Se1	0.98	2.97	3.462 (3)	112

Symmetry codes: (i) 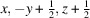 ; (ii) 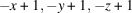 .

**Table 30:** Hydrogen-bond geometry (Å, °) for **2c** [Chem scheme1]

*D*—H⋯*A*	*D*—H	H⋯*A*	*D*⋯*A*	*D*—H⋯*A*
C13—H13*B*⋯Au1	0.98	2.85	3.724 (3)	149
C32—H32*B*⋯Au1	0.98	2.63	3.539 (3)	154
C32—H32*C*⋯Au1^i^	0.98	2.87	3.709 (3)	144
C3—H3⋯I1^i^	1.00	3.18	4.056 (2)	147
C2—H2⋯I1^ii^	1.00	3.22	3.973 (2)	133
C2—H2⋯Au1^ii^	1.00	3.24	4.237 (3)	179

Symmetry codes: (i) 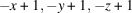 ; (ii) 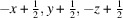 .

**Table 31:** Hydrogen-bond geometry (Å, °) for **6c** [Chem scheme1]

*D*—H⋯*A*	*D*—H	H⋯*A*	*D*⋯*A*	*D*—H⋯*A*
C13—H13*A*⋯Au1	0.98	2.84	3.718 (3)	149
C32—H32*B*⋯Au1	0.98	2.70	3.606 (3)	154
C43—H43*A*⋯Au2	0.98	2.86	3.727 (3)	148
C62—H62*B*⋯Au2	0.98	2.90	3.783 (3)	151
C5—H5⋯I2^i^	1.00	2.94	3.842 (3)	151
C6—H6⋯I1	1.00	3.03	3.948 (3)	153
C3—H3⋯I2^ii^	1.00	3.14	3.949 (3)	139
C42—H42*A*⋯I1^iii^	0.98	3.15	4.090 (3)	162
C2—H2⋯Au1^iv^	1.00	3.06	4.002 (3)	157

Symmetry codes: (i) 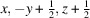 ; (ii) 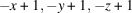 ; (iii) 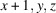 ; (iv) 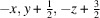 .

**Table 32:** Hydrogen-bond geometry (Å, °) for **7c** [Chem scheme1]

*D*—H⋯*A*	*D*—H	H⋯*A*	*D*⋯*A*	*D*—H⋯*A*
C23—H23*C*⋯Au1	0.98	2.83	3.664 (12)	144
C32—H32*B*⋯Se1	0.98	2.88	3.483 (13)	121
C11—H11*C*⋯I1^ii^	0.98	3.22	4.088 (13)	148
C21—H21*C*⋯I1^iii^	0.98	3.21	4.008 (12)	140
C22—H22*C*⋯I1^iii^	0.98	3.30	4.183 (12)	152
C31—H31*A*⋯I1^iv^	0.98	3.25	4.166 (11)	156
C31—H31*A*⋯Au1^iv^	0.98	3.19	3.642 (12)	110

Symmetry codes: (ii) 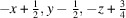 ; (iii) 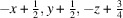 ; (iv) 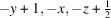 .

**Table:** 

	**1a**	**3a**	**4a**	**5a**
Crystal data				
Chemical formula	[AuCl(C_9_H_21_PS)]	[AuCl(C_11_H_25_PS)]	[AuCl(C_12_H_27_PS)]	[AuCl(C_9_H_21_PSe)]
*M*_r_	424.70	452.76	466.78	471.60
Crystal system, space group	Monoclinic, *P*2_1_/*n*	Monoclinic, *C*2/*c*	Monoclinic, *P*2_1_	Monoclinic, *P*2_1_/*n*
Temperature (K)	100	100	100	100
*a*, *b*, *c* (Å)	8.05892 (17), 11.1342 (2), 15.0596 (4)	26.953 (3), 8.1226 (4), 18.690 (2)	8.45319 (17), 10.9040 (3), 8.7166 (2)	8.0938 (2), 11.3088 (4), 14.9798 (6)
α, β, γ (°)	90, 97.004 (2), 90	90, 132.03 (2), 90	90, 93.583 (2), 90	90, 96.403 (2), 90
*V* (Å^3^)	1341.21 (5)	3039.0 (9)	801.87 (3)	1362.56 (8)
*Z*	4	8	2	4
Radiation type	Mo *K*α	Mo *K*α	Mo *K*α	Mo *K*α
μ (mm^−1^)	11.40	10.07	9.55	13.74
Crystal size (mm)	0.4 × 0.2 × 0.2	0.3 × 0.2 × 0.02	0.12 × 0.04 × 0.04	0.25 × 0.2 × 0.2

Data collection				
Diffractometer	Oxford Diffraction Xcalibur, Eos	Oxford Diffraction Xcalibur, Eos	Oxford Diffraction Xcalibur, Eos	Oxford Diffraction Xcalibur, Eos
Absorption correction	Multi-scan (*CrysAlis PRO*; Rigaku OD, 2020 ▸)	Multi-scan (*CrysAlis PRO*; Rigaku OD, 2020 ▸)	Multi-scan (*CrysAlis PRO*; Rigaku OD, 2020 ▸)	Multi-scan (*CrysAlis PRO*; Rigaku OD, 2020 ▸)
*T*_min_, *T*_max_	0.389, 1.000	0.204, 1.000	0.707, 1.000	0.672, 1.000
No. of measured, independent and observed [*I* > 2σ(*I*)] reflections	39799, 4047, 3612	40680, 4529, 4056	22238, 4617, 4382	39551, 4086, 3254
*R*_int_	0.029	0.040	0.036	0.052
(sin θ/λ)_max_ (Å^−1^)	0.722	0.722	0.720	0.723

Refinement				
*R*[*F* ^2^ > 2σ(*F* ^2^)], *wR*(*F* ^2^), *S*	0.015, 0.032, 1.08	0.021, 0.043, 1.05	0.019, 0.033, 1.04	0.025, 0.038, 1.06
No. of reflections	4047	4529	4617	4086
No. of parameters	125	170	156	125
No. of restraints	0	78	1	0
H-atom treatment	H-atom parameters constrained	H-atom parameters constrained	H-atom parameters constrained	H-atom parameters constrained
Δρ_max_, Δρ_min_ (e Å^−3^)	1.33, −0.75	1.36, −1.24	0.62, −0.75	0.63, −0.80
Extinction method	*SHELXL2019*/3 (Sheldrick, 2015 ▸), *F*_c_^*^ = *kF*_c_[1 + 0.001*xF*_c_^2^λ^3^/sin(2θ)]^-1/4^	None	*SHELXL2019*/3 (Sheldrick, 2015 ▸), *F*_c_^*^ = *kF*_c_[1 + 0.001*xF*_c_^2^λ^3^/sin(2θ)]^-1/4^	*SHELXL2019*/3 (Sheldrick, 2015 ▸), *F*_c_^*^ = *kF*_c_[1 + 0.001*xF*_c_^2^λ^3^/sin(2θ)]^-1/4^
Extinction coefficient	0.00169 (5)	–	0.00088 (16)	0.00043 (3)
Absolute structure	–	–	Refined as an inversion twin	–
Absolute structure parameter	–	–	0.493 (8)	–

**Table:** 

	**6a**	**7a**	**1b**	**3b**
Crystal data				
Chemical formula	[AuCl(C_10_H_23_PSe)]	[AuCl(C_11_H_25_PSe)]	[AuBr(C_9_H_21_PS)]	[AuBr(C_11_H_25_PS)]
*M*_r_	485.63	499.66	469.16	497.22
Crystal system, space group	Monoclinic, *P*2_1_/*n*	Monoclinic, *P*2_1_/*c*	Monoclinic, *P*2_1_/*n*	Monoclinic, *C*2/*c*
Temperature (K)	100	100	100	101
*a*, *b*, *c* (Å)	8.2215 (2), 11.3519 (3), 15.2400 (4)	7.64505 (10), 14.6437 (2), 13.7211 (2)	8.1898 (2), 11.1421 (3), 15.3064 (4)	27.3157 (10), 8.16931 (13), 18.8362 (7)
α, β, γ (°)	90, 92.389 (4), 90	90, 90.4954 (12), 90	90, 97.394 (2), 90	90, 132.187 (7), 90
*V* (Å^3^)	1421.11 (6)	1536.05 (4)	1385.11 (6)	3114.5 (3)
*Z*	4	4	4	8
Radiation type	Mo *K*α	Mo *K*α	Mo *K*α	Mo *K*α
μ (mm^−1^)	13.18	12.20	13.73	12.22
Crystal size (mm)	0.35 × 0.2 × 0.2	0.3 × 0.2 × 0.08	0.13 × 0.08 × 0.06	0.3 × 0.2 × 0.1

Data collection				
Diffractometer	Oxford Diffraction Xcalibur, Eos	Oxford Diffration Xcalibur, Eos	Oxford Diffraction Xcalibur, Eos	Oxford Diffraction Xcalibur, Eos
Absorption correction	Multi-scan (*CrysAlis PRO*; Rigaku OD, 2020 ▸)	Multi-scan (*CrysAlis PRO*; Rigaku OD, 2020 ▸)	Multi-scan (*CrysAlis PRO*; Rigaku OD, 2020 ▸)	Multi-scan (*CrysAlis PRO*; Rigaku OD, 2020 ▸)
*T*_min_, *T*_max_	0.368, 1.000	0.178, 1.000	0.568, 1.000	0.300, 1.000
No. of measured, independent and observed [*I* > 2σ(*I*)] reflections	36891, 4214, 3424	64129, 4654, 4270	51956, 4194, 3563	72942, 4712, 4383
*R*_int_	0.038	0.047	0.040	0.034
(sin θ/λ)_max_ (Å^−1^)	0.719	0.722	0.722	0.722

Refinement				
*R*[*F* ^2^ > 2σ(*F* ^2^)], *wR*(*F* ^2^), *S*	0.021, 0.044, 1.06	0.020, 0.042, 1.06	0.019, 0.032, 1.06	0.015, 0.032, 1.08
No. of reflections	4214	4654	4194	4712
No. of parameters	135	145	125	145
No. of restraints	0	0	0	0
H-atom treatment	H-atom parameters constrained	H-atom parameters constrained	H-atom parameters constrained	H-atom parameters constrained
Δρ_max_, Δρ_min_ (e Å^−3^)	1.74, −1.12	2.04, −1.36	0.88, −0.79	1.22, −0.87
Extinction method	*SHELXL2019*/3 (Sheldrick, 2015 ▸), *F*_c_^*^ = *kF*_c_[1 + 0.001*xF*_c_^2^λ^3^/sin(2θ)]^-1/4^	*SHELXL2019*/3 (Sheldrick, 2015 ▸), *F*_c_^*^ = *kF*_c_[1 + 0.001*xF*_c_^2^λ^3^/sin(2θ)]^-1/4^	*SHELXL2019*/3 (Sheldrick, 2015 ▸), *F*_c_^*^ = *kF*_c_[1 + 0.001*xF*_c_^2^λ^3^/sin(2θ)]^-1/4^	*SHELXL2019*/3 (Sheldrick, 2015 ▸), *F*_c_^*^ = *kF*_c_[1 + 0.001*xF*_c_^2^λ^3^/sin(2θ)]^-1/4^
Extinction coefficient	0.00065 (4)	0.00130 (6)	0.00097 (4)	0.000358 (12)

**Table:** 

	**4b**	**5b**	**6b**	**7b**
Crystal data				
Chemical formula	[AuBr(C_12_H_27_PS)]	[AuBr(C_9_H_21_PSe)]	[AuBr(C_10_H_23_PSe)]	[AuBr(C_11_H_25_PSe)]
*M*_r_	511.24	516.06	530.09	544.11
Crystal system, space group	Monoclinic, *P*2_1_/*c*	Monoclinic, *P*2_1_/*n*	Monoclinic, *P*2_1_/*c*	Monoclinic, *P*2_1_/*c*
Temperature (K)	100	100	100	100
*a*, *b*, *c* (Å)	8.3107 (6), 13.4820 (5), 14.7591 (9)	8.22500 (14), 11.31793 (17), 15.2065 (3)	11.5037 (2), 15.2440 (2), 16.8366 (2)	7.66804 (8), 14.77026 (16), 13.94963 (15)
α, β, γ (°)	90, 90.424 (6), 90	90, 96.6895 (16), 90	90, 90.053 (2), 90	90, 90.4697 (10), 90
*V* (Å^3^)	1653.64 (17)	1405.93 (4)	2952.52 (7)	1579.87 (3)
*Z*	4	4	8	4
Radiation type	Mo *K*α	Mo *K*α	Mo *K*α	Mo *K*α
μ (mm^−1^)	11.51	15.97	15.21	14.22
Crystal size (mm)	0.15 × 0.04 × 0.04	0.16 × 0.15 × 0.15	0.3 × 0.15 × 0.15	0.4 × 0.35 × 0.25

Data collection				
Diffractometer	Oxford Diffraction Xcalibur, Eos	Oxford Diffraction Xcalibur, Eos	Oxford Diffraction Xcalibur, Eos	Oxford Diffraction Xcalibur, Eos
Absorption correction	Multi-scan (*CrysAlis PRO*; Rigaku OD, 2020 ▸)	Multi-scan (*CrysAlis PRO*; Rigaku OD, 2020 ▸)	Multi-scan (*CrysAlis PRO*; Rigaku OD, 2020 ▸)	Multi-scan (*CrysAlis PRO*; Rigaku OD, 2020 ▸)
*T*_min_, *T*_max_	0.445, 1.000	0.529, 1.000	0.427, 1.000	0.070, 0.125
No. of measured, independent and observed [*I* > 2σ(*I*)] reflections	44298, 4445, 3863	63480, 4276, 3812	117465, 8952, 8049	86680, 4774, 4380
*R*_int_	0.079	0.029	0.044	0.072
(sin θ/λ)_max_ (Å^−1^)	0.685	0.721	0.722	0.723

Refinement				
*R*[*F* ^2^ > 2σ(*F* ^2^)], *wR*(*F* ^2^), *S*	0.037, 0.084, 1.04	0.015, 0.029, 1.07	0.022, 0.040, 1.09	0.033, 0.083, 1.07
No. of reflections	4445	4276	8952	4774
No. of parameters	155	125	267	145
No. of restraints	0	0	0	0
H-atom treatment	H-atom parameters constrained	H-atom parameters constrained	H-atom parameters constrained	H-atom parameters constrained
Δρ_max_, Δρ_min_ (e Å^−3^)	2.68, −1.32	1.09, −0.94	1.84, −1.22	3.43, −2.67
Extinction method	None	*SHELXL2019*/3 (Sheldrick, 2015 ▸), *F*_c_^*^ = *kF*_c_[1 + 0.001*xF*_c_^2^λ^3^/sin(2θ)]^-1/4^	None	*SHELXL2019*/3 (Sheldrick, 2015 ▸), *F*_c_^*^ = *kF*_c_[1 + 0.001*xF*_c_^2^λ^3^/sin(2θ)]^-1/4^
Extinction coefficient	–	0.00114 (3)	–	0.00170 (12)

**Table:** 

	**8b**	**2c**	**6c**	**7c**
Crystal data				
Chemical formula	[AuBr(C_12_H_27_PSe)]	[AuI(C_10_H_23_PS)]	[AuI(C_10_H_23_PSe)]	[AuI(C_11_H_25_PSe)]
*M*_r_	558.14	530.18	577.08	591.10
Crystal system, space group	Monoclinic, *P*2_1_/*c*	Monoclinic, *P*2_1_/*n*	Monoclinic, *P*2_1_/*c*	Tetragonal, *P*4_3_2_1_2
Temperature (K)	100	100	100	100
*a*, *b*, *c* (Å)	8.29705 (16), 13.6959 (3), 14.6444 (3)	8.6010 (2), 15.0435 (3), 11.7218 (2)	11.70073 (16), 15.4167 (2), 17.0480 (2)	10.7755 (2), 10.7755 (2), 28.3769 (5)
α, β, γ (°)	90, 90.0892 (18), 90	90, 91.202 (2), 90	90, 89.6296 (12), 90	90, 90, 90
*V* (Å^3^)	1664.11 (5)	1516.34 (5)	3075.16 (7)	3294.86 (14)
*Z*	4	4	8	8
Radiation type	Mo *K*α	Mo *K*α	Mo *K*α	Mo *K*α
μ (mm^−1^)	13.50	11.95	14.02	13.09
Crystal size (mm)	0.2 × 0.1 × 0.1	0.25 × 0.2 × 0.15	0.2 × 0.15 × 0.05	0.35 × 0.2 × 0.15

Data collection				
Diffractometer	Oxford Diffraction Xcalibur, Eos	Oxford Diffraction Xcalibur, Eos	Oxford Diffraction Xcalibur, Eos	Oxford Diffraction Xcalibur, Eos
Absorption correction	Multi-scan (*CrysAlis PRO*; Rigaku OD, 2020 ▸)	Multi-scan (*CrysAlis PRO*; Rigaku OD, 2020 ▸)	Multi-scan (*CrysAlis PRO*; Rigaku OD, 2020 ▸)	Multi-scan (*CrysAlis PRO*; Rigaku OD, 2020 ▸)
*T*_min_, *T*_max_	0.398, 1.000	0.435, 1.000	0.218, 1.000	0.092, 0.244
No. of measured, independent and observed [*I* > 2σ(*I*)] reflections	48617, 5005, 4493	54148, 4580, 4213	124405, 9346, 8443	140787, 4829, 4768
*R*_int_	0.041	0.044	0.042	0.044
(sin θ/λ)_max_ (Å^−1^)	0.722	0.722	0.721	0.704

Refinement				
*R*[*F* ^2^ > 2σ(*F* ^2^)], *wR*(*F* ^2^), *S*	0.023, 0.043, 1.11	0.018, 0.033, 1.12	0.022, 0.040, 1.06	0.032, 0.078, 1.42
No. of reflections	5005	4580	9346	4829
No. of parameters	154	135	267	145
No. of restraints	0	0	0	66
H-atom treatment	H-atom parameters constrained	H-atom parameters constrained	H-atom parameters constrained	H-atom parameters constrained
Δρ_max_, Δρ_min_ (e Å^−3^)	1.39, −0.91	0.92, −0.69	2.96, −1.28	1.40, −1.51
Extinction method	None	*SHELXL2019*/3 (Sheldrick, 2015 ▸), *F*_c_^*^ = *kF*_c_[1 + 0.001*xF*_c_^2^λ^3^/sin(2θ)]^-1/4^	None	None
Extinction coefficient	–	0.00148 (4)	–	–
Absolute structure	–	–	–	Refined as an inversion twin
Absolute structure parameter	–	–	–	0.086 (12)

Computer programs: *CrysAlis PRO* (Rigaku OD, 2020 ▸), *SHELXS97* (Sheldrick, 2008 ▸), *SHELXL2019/3* (Sheldrick, 2015 ▸) and *XP* (Bruker, 1998 ▸).

## supplementary crystallographic information

### Chlorido(tripropylphosphane sulfide-κS)gold (1a) Crystal data

**Table:**

[AuCl(C_9_H_21_PS)]	*F*(000) = 808
*M**_r_* = 424.70	*D*_x_ = 2.103 Mg m^−^^3^
Monoclinic, *P*2_1_/*n*	Mo *K*α radiation, λ = 0.71073 Å
*a* = 8.05892 (17) Å	Cell parameters from 14045 reflections
*b* = 11.1342 (2) Å	θ = 2.3–30.8°
*c* = 15.0596 (4) Å	µ = 11.40 mm^−^^1^
β = 97.004 (2)°	*T* = 100 K
*V* = 1341.21 (5) Å^3^	Block, colourless
*Z* = 4	0.4 × 0.2 × 0.2 mm

### Chlorido(tripropylphosphane sulfide-κS)gold (1a) Data collection

**Table:**

Oxford Diffraction Xcalibur, Eos diffractometer	4047 independent reflections
Radiation source: fine-focus sealed tube	3612 reflections with *I* > 2σ(*I*)
Detector resolution: 16.1419 pixels mm^-1^	*R*_int_ = 0.029
ω scan	θ_max_ = 30.9°, θ_min_ = 2.3°
Absorption correction: multi-scan CrysAlisPro, Version 1.171.35.21 (Rigaku OD, 2020)	*h* = −11→11
*T*_min_ = 0.389, *T*_max_ = 1.000	*k* = −15→15
39799 measured reflections	*l* = −20→20

### Chlorido(tripropylphosphane sulfide-κS)gold (1a) Refinement

**Table:**

Refinement on *F*^2^	Secondary atom site location: difference Fourier map
Least-squares matrix: full	Hydrogen site location: inferred from neighbouring sites
*R*[*F*^2^ > 2σ(*F*^2^)] = 0.015	H-atom parameters constrained
*wR*(*F*^2^) = 0.032	*w* = 1/[σ^2^(*F*_o_^2^) + (0.0111*P*)^2^ + 0.883*P*] where *P* = (*F*_o_^2^ + 2*F*_c_^2^)/3
*S* = 1.08	(Δ/σ)_max_ = 0.002
4047 reflections	Δρ_max_ = 1.33 e Å^−^^3^
125 parameters	Δρ_min_ = −0.75 e Å^−^^3^
0 restraints	Extinction correction: SHELXL-2019/3 (Sheldrick, 2015), Fc^*^=kFc[1+0.001xFc^2^λ^3^/sin(2θ)]^-1/4^
Primary atom site location: structure-invariant direct methods	Extinction coefficient: 0.00169 (5)

### Chlorido(tripropylphosphane sulfide-κS)gold (1a) Special details

**Table:**

Geometry. All esds (except the esd in the dihedral angle between two l.s. planes) are estimated using the full covariance matrix. The cell esds are taken into account individually in the estimation of esds in distances, angles and torsion angles; correlations between esds in cell parameters are only used when they are defined by crystal symmetry. An approximate (isotropic) treatment of cell esds is used for estimating esds involving l.s. planes.

### Chlorido(tripropylphosphane sulfide-κS)gold (1a) Fractional atomic coordinates and isotropic or equivalent isotropic displacement parameters (Å^2^)

**Table:**

	*x*	*y*	*z*	*U*_iso_*/*U*_eq_	
Au1	0.50329 (2)	0.50334 (2)	0.30141 (2)	0.01432 (3)	
Cl1	0.30499 (6)	0.47296 (4)	0.18188 (3)	0.02028 (9)	
P1	0.78099 (5)	0.68964 (4)	0.42190 (3)	0.01098 (9)	
S1	0.69932 (6)	0.51693 (4)	0.42283 (4)	0.01897 (10)	
C1	0.9200 (2)	0.70619 (16)	0.52682 (12)	0.0147 (3)	
H1	1.000594	0.637699	0.528670	0.018*	
C2	0.6047 (2)	0.79477 (16)	0.40785 (13)	0.0154 (4)	
H2	0.542816	0.777882	0.347485	0.018*	
C3	0.9086 (2)	0.71884 (17)	0.33196 (12)	0.0153 (3)	
H3	0.955630	0.801612	0.341415	0.018*	
C11	1.0268 (2)	0.82109 (18)	0.53105 (13)	0.0194 (4)	
H11A	0.955951	0.890946	0.538644	0.029*	
H11B	1.077490	0.829499	0.475430	0.029*	
H11C	1.115123	0.815969	0.581775	0.029*	
C12	0.8306 (3)	0.69331 (19)	0.61062 (13)	0.0223 (4)	
H12A	0.912987	0.675916	0.662427	0.033*	
H12B	0.749564	0.627477	0.602008	0.033*	
H12C	0.772311	0.768311	0.620949	0.033*	
C21	0.4790 (3)	0.7753 (2)	0.47522 (16)	0.0287 (5)	
H21A	0.527202	0.804115	0.534274	0.043*	
H21B	0.453446	0.689493	0.478704	0.043*	
H21C	0.376102	0.819815	0.455734	0.043*	
C22	0.6600 (3)	0.92622 (17)	0.40615 (16)	0.0264 (5)	
H22A	0.562907	0.977030	0.386965	0.040*	
H22B	0.743040	0.935382	0.364215	0.040*	
H22C	0.709377	0.950386	0.466181	0.040*	
C31	1.0567 (3)	0.6320 (2)	0.33596 (15)	0.0286 (5)	
H31A	1.015170	0.550101	0.324260	0.043*	
H31B	1.121047	0.635606	0.395463	0.043*	
H31C	1.128551	0.654776	0.290711	0.043*	
C32	0.8074 (3)	0.71657 (18)	0.23950 (13)	0.0217 (4)	
H32A	0.882117	0.729766	0.193739	0.033*	
H32B	0.722677	0.780119	0.235633	0.033*	
H32C	0.752369	0.638374	0.229744	0.033*	

### Chlorido(tripropylphosphane sulfide-κS)gold (1a) Atomic displacement parameters (Å^2^)

**Table:**

	*U*^11^	*U*^22^	*U*^33^	*U*^12^	*U*^13^	*U*^23^
Au1	0.01461 (4)	0.01095 (4)	0.01748 (5)	−0.00199 (2)	0.00225 (3)	−0.00205 (2)
Cl1	0.0205 (2)	0.0221 (2)	0.0179 (2)	−0.00076 (18)	0.00087 (17)	−0.00512 (18)
P1	0.0115 (2)	0.0092 (2)	0.0119 (2)	0.00053 (15)	−0.00008 (16)	0.00056 (16)
S1	0.0207 (2)	0.0102 (2)	0.0243 (3)	−0.00212 (16)	−0.00425 (19)	0.00323 (17)
C1	0.0154 (8)	0.0151 (8)	0.0130 (9)	0.0007 (6)	−0.0006 (7)	0.0023 (7)
C2	0.0125 (8)	0.0132 (8)	0.0202 (10)	0.0022 (6)	0.0012 (7)	0.0030 (7)
C3	0.0164 (9)	0.0172 (9)	0.0125 (9)	−0.0015 (7)	0.0030 (7)	−0.0014 (7)
C11	0.0193 (9)	0.0250 (10)	0.0129 (9)	−0.0077 (8)	−0.0016 (7)	−0.0005 (8)
C12	0.0275 (10)	0.0261 (10)	0.0128 (9)	−0.0061 (8)	0.0007 (8)	0.0034 (8)
C21	0.0218 (10)	0.0305 (12)	0.0362 (13)	0.0098 (8)	0.0134 (9)	0.0098 (10)
C22	0.0209 (10)	0.0130 (9)	0.0453 (14)	0.0032 (7)	0.0036 (9)	0.0021 (9)
C31	0.0246 (11)	0.0361 (12)	0.0269 (12)	0.0100 (9)	0.0098 (9)	−0.0014 (9)
C32	0.0291 (10)	0.0229 (10)	0.0130 (9)	−0.0063 (8)	0.0017 (8)	−0.0014 (7)

### Chlorido(tripropylphosphane sulfide-κS)gold (1a) Geometric parameters (Å, º)

**Table:**

Au1—S1	2.2711 (5)	C11—H11C	0.9800
Au1—Cl1	2.2820 (5)	C12—H12A	0.9800
P1—C3	1.8269 (18)	C12—H12B	0.9800
P1—C1	1.8312 (19)	C12—H12C	0.9800
P1—C2	1.8330 (18)	C21—H21A	0.9800
P1—S1	2.0332 (6)	C21—H21B	0.9800
C1—C12	1.534 (3)	C21—H21C	0.9800
C1—C11	1.539 (3)	C22—H22A	0.9800
C1—H1	1.0000	C22—H22B	0.9800
C2—C22	1.531 (3)	C22—H22C	0.9800
C2—C21	1.534 (3)	C31—H31A	0.9800
C2—H2	1.0000	C31—H31B	0.9800
C3—C32	1.526 (3)	C31—H31C	0.9800
C3—C31	1.531 (3)	C32—H32A	0.9800
C3—H3	1.0000	C32—H32B	0.9800
C11—H11A	0.9800	C32—H32C	0.9800
C11—H11B	0.9800		
			
S1—Au1—Cl1	175.217 (16)	H11B—C11—H11C	109.5
C3—P1—C1	106.34 (8)	C1—C12—H12A	109.5
C3—P1—C2	107.41 (9)	C1—C12—H12B	109.5
C1—P1—C2	114.79 (9)	H12A—C12—H12B	109.5
C3—P1—S1	112.67 (6)	C1—C12—H12C	109.5
C1—P1—S1	104.69 (6)	H12A—C12—H12C	109.5
C2—P1—S1	110.96 (6)	H12B—C12—H12C	109.5
P1—S1—Au1	104.56 (2)	C2—C21—H21A	109.5
C12—C1—C11	111.10 (16)	C2—C21—H21B	109.5
C12—C1—P1	113.67 (13)	H21A—C21—H21B	109.5
C11—C1—P1	113.55 (13)	C2—C21—H21C	109.5
C12—C1—H1	105.9	H21A—C21—H21C	109.5
C11—C1—H1	105.9	H21B—C21—H21C	109.5
P1—C1—H1	105.9	C2—C22—H22A	109.5
C22—C2—C21	111.30 (17)	C2—C22—H22B	109.5
C22—C2—P1	112.91 (13)	H22A—C22—H22B	109.5
C21—C2—P1	113.48 (13)	C2—C22—H22C	109.5
C22—C2—H2	106.2	H22A—C22—H22C	109.5
C21—C2—H2	106.2	H22B—C22—H22C	109.5
P1—C2—H2	106.2	C3—C31—H31A	109.5
C32—C3—C31	110.65 (16)	C3—C31—H31B	109.5
C32—C3—P1	112.77 (13)	H31A—C31—H31B	109.5
C31—C3—P1	111.41 (14)	C3—C31—H31C	109.5
C32—C3—H3	107.2	H31A—C31—H31C	109.5
C31—C3—H3	107.2	H31B—C31—H31C	109.5
P1—C3—H3	107.2	C3—C32—H32A	109.5
C1—C11—H11A	109.5	C3—C32—H32B	109.5
C1—C11—H11B	109.5	H32A—C32—H32B	109.5
H11A—C11—H11B	109.5	C3—C32—H32C	109.5
C1—C11—H11C	109.5	H32A—C32—H32C	109.5
H11A—C11—H11C	109.5	H32B—C32—H32C	109.5
			
C3—P1—S1—Au1	−71.54 (7)	S1—P1—C2—C22	−179.64 (13)
C1—P1—S1—Au1	173.32 (6)	C3—P1—C2—C21	176.07 (15)
C2—P1—S1—Au1	48.95 (7)	C1—P1—C2—C21	−65.92 (17)
C3—P1—C1—C12	175.97 (14)	S1—P1—C2—C21	52.51 (16)
C2—P1—C1—C12	57.35 (16)	C1—P1—C3—C32	−176.22 (13)
S1—P1—C1—C12	−64.55 (14)	C2—P1—C3—C32	−52.86 (15)
C3—P1—C1—C11	47.67 (15)	S1—P1—C3—C32	69.64 (14)
C2—P1—C1—C11	−70.95 (15)	C1—P1—C3—C31	58.65 (16)
S1—P1—C1—C11	167.15 (12)	C2—P1—C3—C31	−177.99 (14)
C3—P1—C2—C22	−56.09 (17)	S1—P1—C3—C31	−55.49 (15)
C1—P1—C2—C22	61.92 (17)		

### Chlorido(tripropylphosphane sulfide-κS)gold (1a) Hydrogen-bond geometry (Å, º)

**Table:**

*D*—H···*A*	*D*—H	H···*A*	*D*···*A*	*D*—H···*A*
C32—H32*C*···Au1	0.98	2.83	3.615 (2)	138
C3—H3···Cl1^i^	1.00	2.77	3.6734 (19)	151
C12—H12*B*···Au1^ii^	0.98	3.02	3.8290 (19)	141
C1—H1···S1^iii^	1.00	2.99	3.9487 (19)	162

Symmetry codes: (i) −*x*+3/2, *y*+1/2, −*z*+1/2; (ii) −*x*+1, −*y*+1, −*z*+1; (iii) −*x*+2, −*y*+1, −*z*+1.

### [Bis(tert-butyl)(propan-2-yl)tripropylphosphane sulfide-κS]chloridogold (3a) Crystal data

**Table:**

[AuCl(C_11_H_25_PS)]	*F*(000) = 1744
*M**_r_* = 452.76	*D*_x_ = 1.979 Mg m^−^^3^
Monoclinic, *C*2/*c*	Mo *K*α radiation, λ = 0.71073 Å
*a* = 26.953 (3) Å	Cell parameters from 12955 reflections
*b* = 8.1226 (4) Å	θ = 2.2–30.8°
*c* = 18.690 (2) Å	µ = 10.07 mm^−^^1^
β = 132.03 (2)°	*T* = 100 K
*V* = 3039.0 (9) Å^3^	Plate, colourless
*Z* = 8	0.3 × 0.2 × 0.02 mm

### [Bis(tert-butyl)(propan-2-yl)tripropylphosphane sulfide-κS]chloridogold (3a) Data collection

**Table:**

Oxford Diffraction Xcalibur, Eos diffractometer	4529 independent reflections
Radiation source: Enhance (Mo) X-ray Source	4056 reflections with *I* > 2σ(*I*)
Detector resolution: 16.1419 pixels mm^-1^	*R*_int_ = 0.040
ω scan	θ_max_ = 30.9°, θ_min_ = 2.2°
Absorption correction: multi-scan CrysAlisPro, Version 1.171.35.11 (Rigaku OD, 2020)	*h* = −37→37
*T*_min_ = 0.204, *T*_max_ = 1.000	*k* = −11→11
40680 measured reflections	*l* = −26→26

### [Bis(tert-butyl)(propan-2-yl)tripropylphosphane sulfide-κS]chloridogold (3a) Refinement

**Table:**

Refinement on *F*^2^	Primary atom site location: structure-invariant direct methods
Least-squares matrix: full	Secondary atom site location: difference Fourier map
*R*[*F*^2^ > 2σ(*F*^2^)] = 0.021	Hydrogen site location: inferred from neighbouring sites
*wR*(*F*^2^) = 0.043	H-atom parameters constrained
*S* = 1.05	*w* = 1/[σ^2^(*F*_o_^2^) + (0.0157*P*)^2^ + 6.2005*P*] where *P* = (*F*_o_^2^ + 2*F*_c_^2^)/3
4529 reflections	(Δ/σ)_max_ = 0.001
170 parameters	Δρ_max_ = 1.36 e Å^−^^3^
78 restraints	Δρ_min_ = −1.24 e Å^−^^3^

### [Bis(tert-butyl)(propan-2-yl)tripropylphosphane sulfide-κS]chloridogold (3a) Special details

**Table:**

Geometry. All esds (except the esd in the dihedral angle between two l.s. planes) are estimated using the full covariance matrix. The cell esds are taken into account individually in the estimation of esds in distances, angles and torsion angles; correlations between esds in cell parameters are only used when they are defined by crystal symmetry. An approximate (isotropic) treatment of cell esds is used for estimating esds involving l.s. planes.

### [Bis(tert-butyl)(propan-2-yl)tripropylphosphane sulfide-κS]chloridogold (3a) Fractional atomic coordinates and isotropic or equivalent isotropic displacement parameters (Å^2^)

**Table:**

	*x*	*y*	*z*	*U*_iso_*/*U*_eq_	Occ. (<1)
Au1	0.32887 (2)	0.54466 (2)	0.46742 (2)	0.01987 (4)	
Cl1	0.42308 (4)	0.38796 (9)	0.55516 (5)	0.02908 (15)	
P1	0.15826 (3)	0.59393 (8)	0.27777 (4)	0.01738 (13)	
S1	0.24118 (3)	0.72088 (8)	0.38824 (5)	0.02335 (14)	
C1	0.13908 (14)	0.4284 (3)	0.32684 (19)	0.0225 (5)	
C11	0.06755 (17)	0.3691 (6)	0.2540 (3)	0.0290 (9)	0.847 (9)
H11A	0.057685	0.331580	0.195778	0.043*	0.847 (9)
H11B	0.037391	0.459592	0.237535	0.043*	0.847 (9)
H11C	0.061235	0.277812	0.281494	0.043*	0.847 (9)
C12	0.1559 (2)	0.4909 (4)	0.4184 (3)	0.0310 (11)	0.847 (9)
H12A	0.147793	0.403018	0.445448	0.047*	0.847 (9)
H12B	0.127596	0.585835	0.402471	0.047*	0.847 (9)
H12C	0.202939	0.523741	0.465680	0.047*	0.847 (9)
C13	0.18629 (17)	0.2802 (4)	0.3579 (3)	0.0249 (9)	0.847 (9)
H13A	0.232622	0.318836	0.400694	0.037*	0.847 (9)
H13B	0.174423	0.229112	0.300618	0.037*	0.847 (9)
H13C	0.181568	0.199069	0.391795	0.037*	0.847 (9)
C11'	0.0776 (9)	0.317 (3)	0.2493 (13)	0.024 (5)*	0.153 (9)
H11D	0.039415	0.386784	0.199724	0.036*	0.153 (9)
H11E	0.088681	0.245270	0.219642	0.036*	0.153 (9)
H11F	0.066178	0.249856	0.280053	0.036*	0.153 (9)
C12'	0.1148 (12)	0.507 (2)	0.3779 (15)	0.026 (5)*	0.153 (9)
H12D	0.076991	0.580571	0.332791	0.040*	0.153 (9)
H12E	0.100971	0.418165	0.397060	0.040*	0.153 (9)
H12F	0.151613	0.568554	0.435114	0.040*	0.153 (9)
C13'	0.1969 (8)	0.318 (2)	0.4020 (14)	0.027 (5)*	0.153 (9)
H13D	0.236039	0.384878	0.451407	0.041*	0.153 (9)
H13E	0.184803	0.250250	0.431880	0.041*	0.153 (9)
H13F	0.207307	0.245664	0.371470	0.041*	0.153 (9)
C2	0.09199 (13)	0.7560 (3)	0.20743 (18)	0.0219 (5)	
C21	0.1201 (2)	0.9037 (5)	0.1914 (4)	0.0262 (9)	0.811 (9)
H21A	0.136234	0.864949	0.160325	0.039*	0.811 (9)
H21B	0.157175	0.953919	0.253578	0.039*	0.811 (9)
H21C	0.084796	0.985527	0.150029	0.039*	0.811 (9)
C22	0.03084 (18)	0.6918 (4)	0.1070 (2)	0.0288 (11)	0.811 (9)
H22A	−0.005123	0.773497	0.074585	0.043*	0.811 (9)
H22B	0.015555	0.588383	0.113760	0.043*	0.811 (9)
H22C	0.043136	0.672644	0.068851	0.043*	0.811 (9)
C23	0.0735 (2)	0.8143 (6)	0.2636 (3)	0.0286 (9)	0.811 (9)
H23A	0.113890	0.850524	0.327795	0.043*	0.811 (9)
H23B	0.052645	0.723936	0.269993	0.043*	0.811 (9)
H23C	0.042127	0.906481	0.229654	0.043*	0.811 (9)
C21'	0.1003 (11)	0.877 (2)	0.1553 (15)	0.033 (5)*	0.189 (9)
H21D	0.146645	0.915615	0.198596	0.049*	0.189 (9)
H21E	0.089075	0.823807	0.098989	0.049*	0.189 (9)
H21F	0.070433	0.971235	0.134166	0.049*	0.189 (9)
C22'	0.0170 (6)	0.6928 (19)	0.1380 (11)	0.029 (4)*	0.189 (9)
H22D	0.012043	0.613932	0.172462	0.044*	0.189 (9)
H22E	−0.012964	0.786483	0.116839	0.044*	0.189 (9)
H22F	0.005678	0.639056	0.081662	0.044*	0.189 (9)
C23'	0.0917 (10)	0.869 (2)	0.2779 (12)	0.028 (4)*	0.189 (9)
H23D	0.086463	0.799541	0.315260	0.042*	0.189 (9)
H23E	0.133965	0.929841	0.321932	0.042*	0.189 (9)
H23F	0.054531	0.947630	0.239483	0.042*	0.189 (9)
C3	0.17547 (14)	0.4914 (3)	0.20790 (19)	0.0218 (5)	
H3	0.212816	0.413408	0.254624	0.026*	
C31	0.20342 (17)	0.6053 (4)	0.1780 (2)	0.0338 (7)	
H31A	0.223139	0.539669	0.158451	0.051*	
H31B	0.237802	0.675957	0.232417	0.051*	
H31C	0.167393	0.673746	0.123890	0.051*	
C32	0.12067 (18)	0.3834 (5)	0.1238 (2)	0.0438 (9)	
H32A	0.082518	0.452154	0.073295	0.066*	
H32B	0.106455	0.302660	0.145940	0.066*	
H32C	0.137721	0.325790	0.097968	0.066*	

### [Bis(tert-butyl)(propan-2-yl)tripropylphosphane sulfide-κS]chloridogold (3a) Atomic displacement parameters (Å^2^)

**Table:**

	*U*^11^	*U*^22^	*U*^33^	*U*^12^	*U*^13^	*U*^23^
Au1	0.01700 (6)	0.02399 (6)	0.01309 (5)	−0.00739 (4)	0.00780 (4)	−0.00284 (4)
Cl1	0.0202 (3)	0.0347 (4)	0.0228 (3)	−0.0020 (3)	0.0104 (3)	0.0018 (3)
P1	0.0175 (3)	0.0158 (3)	0.0137 (3)	−0.0042 (2)	0.0084 (3)	−0.0043 (2)
S1	0.0222 (3)	0.0192 (3)	0.0166 (3)	−0.0073 (3)	0.0080 (3)	−0.0064 (2)
C1	0.0246 (14)	0.0203 (12)	0.0219 (13)	−0.0064 (10)	0.0153 (12)	−0.0048 (10)
C11	0.0204 (18)	0.032 (2)	0.033 (2)	−0.0073 (16)	0.0172 (17)	−0.0025 (16)
C12	0.046 (3)	0.0288 (18)	0.0264 (19)	−0.0084 (17)	0.027 (2)	−0.0056 (14)
C13	0.0250 (18)	0.0203 (16)	0.0268 (19)	−0.0054 (13)	0.0163 (16)	−0.0015 (13)
C2	0.0194 (13)	0.0182 (12)	0.0188 (12)	0.0004 (10)	0.0090 (11)	−0.0028 (10)
C21	0.031 (2)	0.0192 (17)	0.028 (2)	0.0062 (15)	0.020 (2)	0.0041 (16)
C22	0.0202 (18)	0.0265 (18)	0.0207 (17)	0.0023 (14)	0.0059 (15)	−0.0027 (13)
C23	0.025 (2)	0.033 (2)	0.033 (2)	0.0042 (18)	0.0214 (19)	−0.0011 (17)
C3	0.0253 (14)	0.0204 (12)	0.0170 (12)	−0.0008 (11)	0.0130 (12)	−0.0040 (10)
C31	0.0386 (19)	0.0336 (16)	0.0463 (19)	−0.0041 (14)	0.0354 (17)	−0.0082 (14)
C32	0.042 (2)	0.061 (2)	0.0363 (18)	−0.0250 (18)	0.0292 (17)	−0.0301 (17)

### [Bis(tert-butyl)(propan-2-yl)tripropylphosphane sulfide-κS]chloridogold (3a) Geometric parameters (Å, º)

**Table:**

Au1—S1	2.2674 (8)	C2—C22	1.539 (4)
Au1—Cl1	2.2762 (9)	C2—C21	1.553 (4)
P1—C3	1.851 (3)	C2—C22'	1.587 (11)
P1—C2	1.873 (3)	C2—C23'	1.612 (12)
P1—C1	1.885 (3)	C21—H21A	0.9800
P1—S1	2.0351 (11)	C21—H21B	0.9800
C1—C13'	1.510 (12)	C21—H21C	0.9800
C1—C11	1.512 (4)	C22—H22A	0.9800
C1—C12	1.534 (4)	C22—H22B	0.9800
C1—C13	1.555 (4)	C22—H22C	0.9800
C1—C11'	1.564 (13)	C23—H23A	0.9800
C1—C12'	1.606 (12)	C23—H23B	0.9800
C11—H11A	0.9800	C23—H23C	0.9800
C11—H11B	0.9800	C21'—H21D	0.9800
C11—H11C	0.9800	C21'—H21E	0.9800
C12—H12A	0.9800	C21'—H21F	0.9800
C12—H12B	0.9800	C22'—H22D	0.9800
C12—H12C	0.9800	C22'—H22E	0.9800
C13—H13A	0.9800	C22'—H22F	0.9800
C13—H13B	0.9800	C23'—H23D	0.9800
C13—H13C	0.9800	C23'—H23E	0.9800
C11'—H11D	0.9800	C23'—H23F	0.9800
C11'—H11E	0.9800	C3—C31	1.516 (4)
C11'—H11F	0.9800	C3—C32	1.521 (4)
C12'—H12D	0.9800	C3—H3	1.0000
C12'—H12E	0.9800	C31—H31A	0.9800
C12'—H12F	0.9800	C31—H31B	0.9800
C13'—H13D	0.9800	C31—H31C	0.9800
C13'—H13E	0.9800	C32—H32A	0.9800
C13'—H13F	0.9800	C32—H32B	0.9800
C2—C21'	1.504 (12)	C32—H32C	0.9800
C2—C23	1.508 (4)		
			
S1—Au1—Cl1	174.71 (2)	C22'—C2—C23'	98.8 (9)
C3—P1—C2	113.13 (12)	C21'—C2—P1	115.5 (9)
C3—P1—C1	107.61 (12)	C23—C2—P1	109.6 (2)
C2—P1—C1	113.55 (13)	C22—C2—P1	110.93 (19)
C3—P1—S1	108.67 (10)	C21—C2—P1	108.6 (2)
C2—P1—S1	103.85 (9)	C22'—C2—P1	116.1 (6)
C1—P1—S1	109.91 (9)	C23'—C2—P1	110.6 (7)
P1—S1—Au1	107.40 (4)	C2—C21—H21A	109.5
C11—C1—C12	110.8 (3)	C2—C21—H21B	109.5
C11—C1—C13	108.7 (3)	H21A—C21—H21B	109.5
C12—C1—C13	106.5 (3)	C2—C21—H21C	109.5
C13'—C1—C11'	108.1 (11)	H21A—C21—H21C	109.5
C13'—C1—C12'	105.1 (10)	H21B—C21—H21C	109.5
C11'—C1—C12'	100.3 (11)	C2—C22—H22A	109.5
C13'—C1—P1	115.4 (8)	C2—C22—H22B	109.5
C11—C1—P1	112.9 (2)	H22A—C22—H22B	109.5
C12—C1—P1	109.55 (19)	C2—C22—H22C	109.5
C13—C1—P1	108.1 (2)	H22A—C22—H22C	109.5
C11'—C1—P1	115.3 (9)	H22B—C22—H22C	109.5
C12'—C1—P1	111.2 (7)	C2—C23—H23A	109.5
C1—C11—H11A	109.5	C2—C23—H23B	109.5
C1—C11—H11B	109.5	H23A—C23—H23B	109.5
H11A—C11—H11B	109.5	C2—C23—H23C	109.5
C1—C11—H11C	109.5	H23A—C23—H23C	109.5
H11A—C11—H11C	109.5	H23B—C23—H23C	109.5
H11B—C11—H11C	109.5	C2—C21'—H21D	109.5
C1—C12—H12A	109.5	C2—C21'—H21E	109.5
C1—C12—H12B	109.5	H21D—C21'—H21E	109.5
H12A—C12—H12B	109.5	C2—C21'—H21F	109.5
C1—C12—H12C	109.5	H21D—C21'—H21F	109.5
H12A—C12—H12C	109.5	H21E—C21'—H21F	109.5
H12B—C12—H12C	109.5	C2—C22'—H22D	109.5
C1—C13—H13A	109.5	C2—C22'—H22E	109.5
C1—C13—H13B	109.5	H22D—C22'—H22E	109.5
H13A—C13—H13B	109.5	C2—C22'—H22F	109.5
C1—C13—H13C	109.5	H22D—C22'—H22F	109.5
H13A—C13—H13C	109.5	H22E—C22'—H22F	109.5
H13B—C13—H13C	109.5	C2—C23'—H23D	109.5
C1—C11'—H11D	109.5	C2—C23'—H23E	109.5
C1—C11'—H11E	109.5	H23D—C23'—H23E	109.5
H11D—C11'—H11E	109.5	C2—C23'—H23F	109.5
C1—C11'—H11F	109.5	H23D—C23'—H23F	109.5
H11D—C11'—H11F	109.5	H23E—C23'—H23F	109.5
H11E—C11'—H11F	109.5	C31—C3—C32	110.5 (2)
C1—C12'—H12D	109.5	C31—C3—P1	113.99 (19)
C1—C12'—H12E	109.5	C32—C3—P1	117.4 (2)
H12D—C12'—H12E	109.5	C31—C3—H3	104.5
C1—C12'—H12F	109.5	C32—C3—H3	104.5
H12D—C12'—H12F	109.5	P1—C3—H3	104.5
H12E—C12'—H12F	109.5	C3—C31—H31A	109.5
C1—C13'—H13D	109.5	C3—C31—H31B	109.5
C1—C13'—H13E	109.5	H31A—C31—H31B	109.5
H13D—C13'—H13E	109.5	C3—C31—H31C	109.5
C1—C13'—H13F	109.5	H31A—C31—H31C	109.5
H13D—C13'—H13F	109.5	H31B—C31—H31C	109.5
H13E—C13'—H13F	109.5	C3—C32—H32A	109.5
C23—C2—C22	111.9 (3)	C3—C32—H32B	109.5
C23—C2—C21	108.9 (3)	H32A—C32—H32B	109.5
C22—C2—C21	106.8 (3)	C3—C32—H32C	109.5
C21'—C2—C22'	110.0 (10)	H32A—C32—H32C	109.5
C21'—C2—C23'	103.9 (10)	H32B—C32—H32C	109.5
			
C3—P1—S1—Au1	−51.44 (10)	S1—P1—C2—C21'	68.9 (9)
C2—P1—S1—Au1	−172.13 (9)	C3—P1—C2—C23	171.3 (3)
C1—P1—S1—Au1	66.07 (10)	C1—P1—C2—C23	48.2 (3)
C3—P1—C1—C13'	67.3 (10)	S1—P1—C2—C23	−71.1 (3)
C2—P1—C1—C13'	−166.7 (10)	C3—P1—C2—C22	47.2 (3)
S1—P1—C1—C13'	−50.9 (10)	C1—P1—C2—C22	−75.8 (3)
C3—P1—C1—C11	−81.8 (3)	S1—P1—C2—C22	164.9 (2)
C2—P1—C1—C11	44.2 (3)	C3—P1—C2—C21	−69.9 (3)
S1—P1—C1—C11	160.0 (3)	C1—P1—C2—C21	167.1 (2)
C3—P1—C1—C12	154.2 (3)	S1—P1—C2—C21	47.8 (2)
C2—P1—C1—C12	−79.8 (3)	C3—P1—C2—C22'	82.2 (8)
S1—P1—C1—C12	36.0 (3)	C1—P1—C2—C22'	−40.9 (8)
C3—P1—C1—C13	38.5 (2)	S1—P1—C2—C22'	−160.2 (8)
C2—P1—C1—C13	164.5 (2)	C3—P1—C2—C23'	−166.3 (8)
S1—P1—C1—C13	−79.7 (2)	C1—P1—C2—C23'	70.7 (8)
C3—P1—C1—C11'	−59.9 (10)	S1—P1—C2—C23'	−48.7 (8)
C2—P1—C1—C11'	66.1 (10)	C2—P1—C3—C31	63.8 (2)
S1—P1—C1—C11'	−178.1 (10)	C1—P1—C3—C31	−170.0 (2)
C3—P1—C1—C12'	−173.1 (9)	S1—P1—C3—C31	−51.0 (2)
C2—P1—C1—C12'	−47.1 (9)	C2—P1—C3—C32	−67.6 (3)
S1—P1—C1—C12'	68.7 (9)	C1—P1—C3—C32	58.6 (3)
C3—P1—C2—C21'	−48.7 (10)	S1—P1—C3—C32	177.6 (2)
C1—P1—C2—C21'	−171.8 (9)		

### [Bis(tert-butyl)(propan-2-yl)tripropylphosphane sulfide-κS]chloridogold (3a) Hydrogen-bond geometry (Å, º)

**Table:**

*D*—H···*A*	*D*—H	H···*A*	*D*···*A*	*D*—H···*A*
C13—H13*A*···Au1	0.98	2.71	3.611 (3)	154
C12—H12*C*···S1	0.98	2.78	3.291 (4)	113
C21—H21*B*···S1	0.98	2.72	3.211 (5)	111

### Chlorido(tri-tert-butylphosphane sulfide-κS)gold (4a) Crystal data

**Table:**

[AuCl(C_12_H_27_PS)]	*F*(000) = 452
*M**_r_* = 466.78	*D*_x_ = 1.933 Mg m^−^^3^
Monoclinic, *P*2_1_	Mo *K*α radiation, λ = 0.71073 Å
*a* = 8.45319 (17) Å	Cell parameters from 9477 reflections
*b* = 10.9040 (3) Å	θ = 2.3–30.7°
*c* = 8.7166 (2) Å	µ = 9.55 mm^−^^1^
β = 93.583 (2)°	*T* = 100 K
*V* = 801.87 (3) Å^3^	Block, colourless
*Z* = 2	0.12 × 0.04 × 0.04 mm

### Chlorido(tri-tert-butylphosphane sulfide-κS)gold (4a) Data collection

**Table:**

Oxford Diffraction Xcalibur, Eos diffractometer	4617 independent reflections
Radiation source: Enhance (Mo) X-ray Source	4382 reflections with *I* > 2σ(*I*)
Detector resolution: 16.1419 pixels mm^-1^	*R*_int_ = 0.036
ω scan	θ_max_ = 30.8°, θ_min_ = 2.3°
Absorption correction: multi-scan CrysAlisPro, Version 1.171.35.21 (Rigaku OD, 2020)	*h* = −12→11
*T*_min_ = 0.707, *T*_max_ = 1.000	*k* = −15→15
22238 measured reflections	*l* = −12→12

### Chlorido(tri-tert-butylphosphane sulfide-κS)gold (4a) Refinement

**Table:**

Refinement on *F*^2^	Hydrogen site location: inferred from neighbouring sites
Least-squares matrix: full	H-atom parameters constrained
*R*[*F*^2^ > 2σ(*F*^2^)] = 0.019	*w* = 1/[σ^2^(*F*_o_^2^) + (0.011*P*)^2^] where *P* = (*F*_o_^2^ + 2*F*_c_^2^)/3
*wR*(*F*^2^) = 0.033	(Δ/σ)_max_ = 0.001
*S* = 1.03	Δρ_max_ = 0.62 e Å^−^^3^
4617 reflections	Δρ_min_ = −0.75 e Å^−^^3^
156 parameters	Extinction correction: SHELXL-2019/3 (Sheldrick, 2015), Fc^*^=kFc[1+0.001xFc^2^λ^3^/sin(2θ)]^-1/4^
1 restraint	Extinction coefficient: 0.00088 (16)
Primary atom site location: structure-invariant direct methods	Absolute structure: Refined as an inversion twin.
Secondary atom site location: difference Fourier map	Absolute structure parameter: 0.493 (8)

### Chlorido(tri-tert-butylphosphane sulfide-κS)gold (4a) Fractional atomic coordinates and isotropic or equivalent isotropic displacement parameters (Å^2^)

**Table:**

	*x*	*y*	*z*	*U*_iso_*/*U*_eq_	
Au1	0.31201 (2)	0.56415 (4)	0.17075 (2)	0.01412 (5)	
Cl1	0.16221 (10)	0.5699 (4)	−0.05619 (10)	0.0189 (2)	
P1	0.68768 (10)	0.5570 (4)	0.34887 (10)	0.0112 (3)	
S1	0.45445 (11)	0.5525 (4)	0.39973 (11)	0.0160 (4)	
C1	0.7946 (4)	0.5705 (10)	0.5465 (4)	0.0157 (8)	
C2	0.7399 (9)	0.4069 (6)	0.2529 (8)	0.0107 (13)	
C3	0.7305 (9)	0.6940 (6)	0.2248 (8)	0.0126 (14)	
C11	0.9722 (5)	0.5399 (5)	0.5433 (5)	0.0218 (14)	
H11A	1.025548	0.558348	0.643676	0.033*	
H11B	1.019336	0.589148	0.463893	0.033*	
H11C	0.984837	0.452582	0.520174	0.033*	
C12	0.7763 (6)	0.7022 (5)	0.6099 (5)	0.0217 (10)	
H12A	0.828416	0.760500	0.543936	0.033*	
H12B	0.825412	0.706776	0.714582	0.033*	
H12C	0.663482	0.722555	0.611207	0.033*	
C13	0.7204 (6)	0.4856 (5)	0.6633 (5)	0.0225 (10)	
H13A	0.784327	0.487915	0.761022	0.034*	
H13B	0.717121	0.401449	0.623621	0.034*	
H13C	0.612418	0.513184	0.679729	0.034*	
C21	0.9051 (8)	0.4118 (6)	0.1875 (6)	0.0159 (13)	
H21A	0.905982	0.475338	0.107954	0.024*	
H21B	0.929363	0.332133	0.142587	0.024*	
H21C	0.984985	0.431201	0.270260	0.024*	
C22	0.7334 (5)	0.3014 (4)	0.3689 (5)	0.0187 (9)	
H22A	0.739656	0.222865	0.314982	0.028*	
H22B	0.633712	0.305586	0.420296	0.028*	
H22C	0.822823	0.308415	0.445620	0.028*	
C23	0.6179 (5)	0.3757 (4)	0.1209 (5)	0.0180 (9)	
H23A	0.648524	0.299347	0.071520	0.027*	
H23B	0.613931	0.442353	0.045267	0.027*	
H23C	0.513170	0.365667	0.161610	0.027*	
C31	0.9079 (9)	0.7322 (6)	0.2387 (6)	0.0155 (13)	
H31A	0.938675	0.752911	0.345808	0.023*	
H31B	0.923658	0.803689	0.173256	0.023*	
H31C	0.973333	0.664016	0.205725	0.023*	
C32	0.6276 (5)	0.8046 (4)	0.2701 (5)	0.0180 (9)	
H32A	0.653075	0.876203	0.208330	0.027*	
H32B	0.649634	0.823282	0.379334	0.027*	
H32C	0.515199	0.784127	0.251279	0.027*	
C33	0.6890 (5)	0.6701 (4)	0.0529 (5)	0.0160 (9)	
H33A	0.759057	0.606472	0.015972	0.024*	
H33B	0.702814	0.745915	−0.005224	0.024*	
H33C	0.578575	0.642859	0.038511	0.024*	

### Chlorido(tri-tert-butylphosphane sulfide-κS)gold (4a) Atomic displacement parameters (Å^2^)

**Table:**

	*U*^11^	*U*^22^	*U*^33^	*U*^12^	*U*^13^	*U*^23^
Au1	0.01100 (6)	0.01472 (8)	0.01674 (7)	0.00010 (15)	0.00179 (4)	−0.00056 (16)
Cl1	0.0194 (4)	0.0200 (7)	0.0169 (4)	−0.0010 (11)	−0.0007 (3)	−0.0003 (11)
P1	0.0120 (3)	0.0113 (9)	0.0102 (4)	−0.0018 (8)	0.0004 (3)	−0.0003 (9)
S1	0.0140 (4)	0.0195 (13)	0.0149 (4)	−0.0012 (7)	0.0038 (3)	−0.0002 (7)
C1	0.0178 (15)	0.018 (2)	0.0109 (15)	−0.004 (4)	−0.0020 (12)	−0.002 (4)
C2	0.016 (3)	0.006 (3)	0.009 (3)	0.001 (2)	0.000 (2)	0.000 (2)
C3	0.014 (3)	0.009 (3)	0.015 (3)	0.002 (2)	0.004 (2)	−0.004 (2)
C11	0.0218 (19)	0.027 (4)	0.0156 (19)	−0.006 (2)	−0.0070 (15)	0.0032 (19)
C12	0.026 (2)	0.022 (3)	0.017 (2)	−0.005 (2)	0.001 (2)	−0.005 (2)
C13	0.028 (3)	0.029 (3)	0.010 (2)	−0.006 (2)	−0.0004 (19)	0.000 (2)
C21	0.016 (2)	0.013 (3)	0.019 (3)	0.002 (2)	−0.001 (3)	0.002 (2)
C22	0.022 (2)	0.013 (2)	0.021 (2)	−0.0009 (18)	0.0005 (18)	0.0008 (18)
C23	0.019 (2)	0.016 (2)	0.019 (2)	0.0015 (18)	−0.0001 (18)	−0.0061 (19)
C31	0.019 (2)	0.014 (3)	0.014 (3)	−0.001 (2)	0.003 (2)	0.000 (2)
C32	0.024 (2)	0.012 (2)	0.019 (2)	0.0024 (18)	0.0042 (18)	0.0033 (18)
C33	0.017 (2)	0.017 (2)	0.015 (2)	0.0016 (18)	0.0007 (17)	0.0029 (18)

### Chlorido(tri-tert-butylphosphane sulfide-κS)gold (4a) Geometric parameters (Å, º)

**Table:**

Au1—S1	2.2692 (10)	C13—H13A	0.9800
Au1—Cl1	2.2820 (8)	C13—H13B	0.9800
P1—C3	1.892 (8)	C13—H13C	0.9800
P1—C1	1.901 (4)	C21—H21A	0.9800
P1—C2	1.903 (8)	C21—H21B	0.9800
P1—S1	2.0482 (12)	C21—H21C	0.9800
C1—C13	1.539 (8)	C22—H22A	0.9800
C1—C11	1.540 (6)	C22—H22B	0.9800
C1—C12	1.549 (11)	C22—H22C	0.9800
C2—C23	1.535 (8)	C23—H23A	0.9800
C2—C22	1.535 (7)	C23—H23B	0.9800
C2—C21	1.542 (10)	C23—H23C	0.9800
C3—C33	1.540 (8)	C31—H31A	0.9800
C3—C32	1.553 (8)	C31—H31B	0.9800
C3—C31	1.554 (10)	C31—H31C	0.9800
C11—H11A	0.9800	C32—H32A	0.9800
C11—H11B	0.9800	C32—H32B	0.9800
C11—H11C	0.9800	C32—H32C	0.9800
C12—H12A	0.9800	C33—H33A	0.9800
C12—H12B	0.9800	C33—H33B	0.9800
C12—H12C	0.9800	C33—H33C	0.9800
			
S1—Au1—Cl1	177.70 (15)	H13A—C13—H13B	109.5
C3—P1—C1	111.1 (4)	C1—C13—H13C	109.5
C3—P1—C2	111.7 (2)	H13A—C13—H13C	109.5
C1—P1—C2	110.8 (4)	H13B—C13—H13C	109.5
C3—P1—S1	111.3 (3)	C2—C21—H21A	109.5
C1—P1—S1	102.45 (12)	C2—C21—H21B	109.5
C2—P1—S1	109.1 (3)	H21A—C21—H21B	109.5
P1—S1—Au1	105.86 (5)	C2—C21—H21C	109.5
C13—C1—C11	108.6 (6)	H21A—C21—H21C	109.5
C13—C1—C12	105.5 (4)	H21B—C21—H21C	109.5
C11—C1—C12	109.1 (5)	C2—C22—H22A	109.5
C13—C1—P1	111.2 (4)	C2—C22—H22B	109.5
C11—C1—P1	112.1 (3)	H22A—C22—H22B	109.5
C12—C1—P1	110.0 (5)	C2—C22—H22C	109.5
C23—C2—C22	106.2 (5)	H22A—C22—H22C	109.5
C23—C2—C21	108.2 (5)	H22B—C22—H22C	109.5
C22—C2—C21	109.9 (5)	C2—C23—H23A	109.5
C23—C2—P1	110.9 (4)	C2—C23—H23B	109.5
C22—C2—P1	109.6 (4)	H23A—C23—H23B	109.5
C21—C2—P1	111.9 (4)	C2—C23—H23C	109.5
C33—C3—C32	106.3 (5)	H23A—C23—H23C	109.5
C33—C3—C31	106.4 (5)	H23B—C23—H23C	109.5
C32—C3—C31	109.0 (5)	C3—C31—H31A	109.5
C33—C3—P1	112.5 (4)	C3—C31—H31B	109.5
C32—C3—P1	109.7 (5)	H31A—C31—H31B	109.5
C31—C3—P1	112.7 (5)	C3—C31—H31C	109.5
C1—C11—H11A	109.5	H31A—C31—H31C	109.5
C1—C11—H11B	109.5	H31B—C31—H31C	109.5
H11A—C11—H11B	109.5	C3—C32—H32A	109.5
C1—C11—H11C	109.5	C3—C32—H32B	109.5
H11A—C11—H11C	109.5	H32A—C32—H32B	109.5
H11B—C11—H11C	109.5	C3—C32—H32C	109.5
C1—C12—H12A	109.5	H32A—C32—H32C	109.5
C1—C12—H12B	109.5	H32B—C32—H32C	109.5
H12A—C12—H12B	109.5	C3—C33—H33A	109.5
C1—C12—H12C	109.5	C3—C33—H33B	109.5
H12A—C12—H12C	109.5	H33A—C33—H33B	109.5
H12B—C12—H12C	109.5	C3—C33—H33C	109.5
C1—C13—H13A	109.5	H33A—C33—H33C	109.5
C1—C13—H13B	109.5	H33B—C33—H33C	109.5
			
C3—P1—S1—Au1	53.2 (3)	C3—P1—C2—C22	169.3 (5)
C1—P1—S1—Au1	172.0 (4)	C1—P1—C2—C22	44.9 (5)
C2—P1—S1—Au1	−70.5 (3)	S1—P1—C2—C22	−67.2 (5)
C3—P1—C1—C13	161.2 (5)	C3—P1—C2—C21	47.1 (4)
C2—P1—C1—C13	−74.0 (5)	C1—P1—C2—C21	−77.3 (5)
S1—P1—C1—C13	42.3 (6)	S1—P1—C2—C21	170.6 (3)
C3—P1—C1—C11	−76.9 (7)	C1—P1—C3—C33	163.0 (4)
C2—P1—C1—C11	47.8 (7)	C2—P1—C3—C33	38.7 (4)
S1—P1—C1—C11	164.1 (6)	S1—P1—C3—C33	−83.5 (5)
C3—P1—C1—C12	44.6 (4)	C1—P1—C3—C32	−78.8 (5)
C2—P1—C1—C12	169.4 (4)	C2—P1—C3—C32	156.9 (5)
S1—P1—C1—C12	−74.3 (4)	S1—P1—C3—C32	34.6 (5)
C3—P1—C2—C23	−73.8 (4)	C1—P1—C3—C31	42.8 (5)
C1—P1—C2—C23	161.8 (4)	C2—P1—C3—C31	−81.5 (5)
S1—P1—C2—C23	49.7 (5)	S1—P1—C3—C31	156.2 (4)

### Chlorido(tri-tert-butylphosphane sulfide-κS)gold (4a) Hydrogen-bond geometry (Å, º)

**Table:**

*D*—H···*A*	*D*—H	H···*A*	*D*···*A*	*D*—H···*A*
C11—H11*A*···Cl1^i^	0.98	2.80	3.765 (4)	170
C21—H21*A*···Cl1^ii^	0.98	2.86	3.575 (7)	130
C23—H23*C*···Au1	0.98	2.76	3.352 (4)	120
C33—H33*C*···Au1	0.98	2.73	3.599 (4)	148
C13—H13*C*···S1	0.98	2.74	3.197 (5)	109
C32—H32*C*···S1	0.98	2.90	3.344 (6)	109

Symmetry codes: (i) *x*+1, *y*, *z*+1; (ii) *x*+1, *y*, *z*.

### Chlorido(tripropylphosphane selenide-κSe)gold (5a) Crystal data

**Table:**

[AuCl(C_9_H_21_PSe)]	*F*(000) = 880
*M**_r_* = 471.60	*D*_x_ = 2.299 Mg m^−^^3^
Monoclinic, *P*2_1_/*n*	Mo *K*α radiation, λ = 0.71073 Å
*a* = 8.0938 (2) Å	Cell parameters from 8059 reflections
*b* = 11.3088 (4) Å	θ = 2.3–30.9°
*c* = 14.9798 (6) Å	µ = 13.74 mm^−^^1^
β = 96.403 (2)°	*T* = 100 K
*V* = 1362.56 (8) Å^3^	Block, colourless
*Z* = 4	0.25 × 0.2 × 0.2 mm

### Chlorido(tripropylphosphane selenide-κSe)gold (5a) Data collection

**Table:**

Oxford Diffraction Xcalibur, Eos diffractometer	4086 independent reflections
Radiation source: Enhance (Mo) X-ray Source	3254 reflections with *I* > 2σ(*I*)
Detector resolution: 16.1419 pixels mm^-1^	*R*_int_ = 0.052
ω scan	θ_max_ = 30.9°, θ_min_ = 2.3°
Absorption correction: multi-scan CrysAlisPro, Version 1.171.35.21 (Rigaku OD, 2020)	*h* = −11→11
*T*_min_ = 0.672, *T*_max_ = 1.000	*k* = −15→16
39551 measured reflections	*l* = −21→21

### Chlorido(tripropylphosphane selenide-κSe)gold (5a) Refinement

**Table:**

Refinement on *F*^2^	Secondary atom site location: difference Fourier map
Least-squares matrix: full	Hydrogen site location: inferred from neighbouring sites
*R*[*F*^2^ > 2σ(*F*^2^)] = 0.025	H-atom parameters constrained
*wR*(*F*^2^) = 0.038	*w* = 1/[σ^2^(*F*_o_^2^) + (0.0076*P*)^2^ + 0.9306*P*] where *P* = (*F*_o_^2^ + 2*F*_c_^2^)/3
*S* = 1.06	(Δ/σ)_max_ = 0.002
4086 reflections	Δρ_max_ = 0.63 e Å^−^^3^
125 parameters	Δρ_min_ = −0.80 e Å^−^^3^
0 restraints	Extinction correction: SHELXL-2019/3 (Sheldrick, 2015), Fc^*^=kFc[1+0.001xFc^2^λ^3^/sin(2θ)]^-1/4^
Primary atom site location: structure-invariant direct methods	Extinction coefficient: 0.00043 (3)

### Chlorido(tripropylphosphane selenide-κSe)gold (5a) Special details

**Table:**

Geometry. All esds (except the esd in the dihedral angle between two l.s. planes) are estimated using the full covariance matrix. The cell esds are taken into account individually in the estimation of esds in distances, angles and torsion angles; correlations between esds in cell parameters are only used when they are defined by crystal symmetry. An approximate (isotropic) treatment of cell esds is used for estimating esds involving l.s. planes.

### Chlorido(tripropylphosphane selenide-κSe)gold (5a) Fractional atomic coordinates and isotropic or equivalent isotropic displacement parameters (Å^2^)

**Table:**

	*x*	*y*	*z*	*U*_iso_*/*U*_eq_	
Au1	0.49486 (2)	0.50427 (2)	0.29627 (2)	0.01590 (4)	
Cl1	0.29534 (9)	0.47658 (7)	0.17758 (5)	0.02236 (16)	
P1	0.78642 (9)	0.69476 (7)	0.42140 (5)	0.01221 (15)	
Se1	0.69997 (4)	0.51162 (3)	0.42250 (2)	0.02040 (7)	
C1	0.9237 (4)	0.7102 (3)	0.5263 (2)	0.0168 (6)	
H1	1.005400	0.643876	0.526460	0.020*	
C2	0.6099 (4)	0.7980 (3)	0.4075 (2)	0.0168 (6)	
H2	0.546203	0.778922	0.348157	0.020*	
C3	0.9145 (4)	0.7232 (3)	0.33009 (19)	0.0159 (6)	
H3	0.957730	0.805819	0.337845	0.019*	
C11	1.0279 (4)	0.8238 (3)	0.5314 (2)	0.0208 (7)	
H11A	0.956508	0.891723	0.540645	0.031*	
H11B	1.077755	0.834162	0.475241	0.031*	
H11C	1.116120	0.818200	0.581686	0.031*	
C12	0.8366 (4)	0.6931 (3)	0.6111 (2)	0.0241 (7)	
H12A	0.919362	0.674128	0.661924	0.036*	
H12B	0.756267	0.628247	0.601653	0.036*	
H12C	0.778368	0.766085	0.624061	0.036*	
C21	0.4891 (4)	0.7819 (3)	0.4778 (2)	0.0295 (9)	
H21A	0.540067	0.811563	0.535917	0.044*	
H21B	0.462897	0.697762	0.483150	0.044*	
H21C	0.386662	0.826020	0.459522	0.044*	
C22	0.6638 (4)	0.9269 (3)	0.4020 (3)	0.0269 (8)	
H22A	0.566625	0.976125	0.382880	0.040*	
H22B	0.745006	0.934221	0.358433	0.040*	
H22C	0.714392	0.953109	0.461181	0.040*	
C31	1.0648 (4)	0.6410 (3)	0.3356 (2)	0.0279 (8)	
H31A	1.026858	0.558853	0.328530	0.042*	
H31B	1.130730	0.650427	0.394145	0.042*	
H31C	1.133284	0.660786	0.287761	0.042*	
C32	0.8140 (4)	0.7165 (3)	0.2381 (2)	0.0231 (7)	
H32A	0.887429	0.730335	0.191386	0.035*	
H32B	0.726546	0.776774	0.233864	0.035*	
H32C	0.763367	0.637950	0.229862	0.035*	

### Chlorido(tripropylphosphane selenide-κSe)gold (5a) Atomic displacement parameters (Å^2^)

**Table:**

	*U*^11^	*U*^22^	*U*^33^	*U*^12^	*U*^13^	*U*^23^
Au1	0.01504 (6)	0.01335 (6)	0.01956 (6)	−0.00148 (5)	0.00305 (4)	−0.00202 (5)
Cl1	0.0215 (4)	0.0252 (4)	0.0204 (4)	−0.0003 (3)	0.0018 (3)	−0.0055 (3)
P1	0.0113 (3)	0.0118 (4)	0.0132 (4)	0.0002 (3)	0.0000 (3)	0.0009 (3)
Se1	0.02040 (15)	0.01185 (15)	0.02735 (17)	−0.00217 (12)	−0.00436 (12)	0.00413 (13)
C1	0.0161 (15)	0.0186 (16)	0.0152 (16)	−0.0004 (12)	−0.0007 (12)	0.0005 (12)
C2	0.0152 (15)	0.0148 (15)	0.0206 (17)	0.0018 (11)	0.0024 (13)	0.0027 (12)
C3	0.0179 (15)	0.0148 (15)	0.0156 (16)	−0.0008 (12)	0.0038 (13)	−0.0013 (12)
C11	0.0206 (16)	0.0254 (18)	0.0164 (16)	−0.0086 (13)	0.0016 (13)	−0.0016 (13)
C12	0.0286 (18)	0.0267 (19)	0.0168 (17)	−0.0053 (14)	0.0013 (14)	0.0033 (14)
C21	0.0197 (17)	0.032 (2)	0.039 (2)	0.0112 (15)	0.0124 (17)	0.0095 (16)
C22	0.0197 (17)	0.0135 (16)	0.048 (2)	0.0047 (13)	0.0049 (16)	0.0055 (15)
C31	0.0232 (18)	0.033 (2)	0.029 (2)	0.0083 (15)	0.0085 (15)	−0.0009 (15)
C32	0.0303 (19)	0.0234 (18)	0.0155 (17)	−0.0048 (14)	0.0023 (14)	−0.0004 (13)

### Chlorido(tripropylphosphane selenide-κSe)gold (5a) Geometric parameters (Å, º)

**Table:**

Au1—Cl1	2.2862 (8)	C11—H11C	0.9800
Au1—Se1	2.3745 (3)	C12—H12A	0.9800
P1—C1	1.829 (3)	C12—H12B	0.9800
P1—C3	1.834 (3)	C12—H12C	0.9800
P1—C2	1.839 (3)	C21—H21A	0.9800
P1—Se1	2.1868 (8)	C21—H21B	0.9800
C1—C12	1.533 (4)	C21—H21C	0.9800
C1—C11	1.534 (4)	C22—H22A	0.9800
C1—H1	1.0000	C22—H22B	0.9800
C2—C22	1.526 (4)	C22—H22C	0.9800
C2—C21	1.526 (4)	C31—H31A	0.9800
C2—H2	1.0000	C31—H31B	0.9800
C3—C32	1.522 (4)	C31—H31C	0.9800
C3—C31	1.526 (4)	C32—H32A	0.9800
C3—H3	1.0000	C32—H32B	0.9800
C11—H11A	0.9800	C32—H32C	0.9800
C11—H11B	0.9800		
			
Cl1—Au1—Se1	174.03 (2)	H11B—C11—H11C	109.5
C1—P1—C3	106.51 (14)	C1—C12—H12A	109.5
C1—P1—C2	115.25 (14)	C1—C12—H12B	109.5
C3—P1—C2	107.49 (14)	H12A—C12—H12B	109.5
C1—P1—Se1	104.38 (10)	C1—C12—H12C	109.5
C3—P1—Se1	112.30 (10)	H12A—C12—H12C	109.5
C2—P1—Se1	110.93 (10)	H12B—C12—H12C	109.5
P1—Se1—Au1	102.89 (2)	C2—C21—H21A	109.5
C12—C1—C11	111.4 (3)	C2—C21—H21B	109.5
C12—C1—P1	114.1 (2)	H21A—C21—H21B	109.5
C11—C1—P1	113.7 (2)	C2—C21—H21C	109.5
C12—C1—H1	105.6	H21A—C21—H21C	109.5
C11—C1—H1	105.6	H21B—C21—H21C	109.5
P1—C1—H1	105.6	C2—C22—H22A	109.5
C22—C2—C21	111.1 (3)	C2—C22—H22B	109.5
C22—C2—P1	112.9 (2)	H22A—C22—H22B	109.5
C21—C2—P1	113.3 (2)	C2—C22—H22C	109.5
C22—C2—H2	106.3	H22A—C22—H22C	109.5
C21—C2—H2	106.3	H22B—C22—H22C	109.5
P1—C2—H2	106.3	C3—C31—H31A	109.5
C32—C3—C31	111.2 (3)	C3—C31—H31B	109.5
C32—C3—P1	112.1 (2)	H31A—C31—H31B	109.5
C31—C3—P1	111.5 (2)	C3—C31—H31C	109.5
C32—C3—H3	107.3	H31A—C31—H31C	109.5
C31—C3—H3	107.3	H31B—C31—H31C	109.5
P1—C3—H3	107.3	C3—C32—H32A	109.5
C1—C11—H11A	109.5	C3—C32—H32B	109.5
C1—C11—H11B	109.5	H32A—C32—H32B	109.5
H11A—C11—H11B	109.5	C3—C32—H32C	109.5
C1—C11—H11C	109.5	H32A—C32—H32C	109.5
H11A—C11—H11C	109.5	H32B—C32—H32C	109.5
			
C1—P1—Se1—Au1	172.39 (10)	Se1—P1—C2—C22	−177.4 (2)
C3—P1—Se1—Au1	−72.63 (11)	C1—P1—C2—C21	−63.1 (3)
C2—P1—Se1—Au1	47.67 (11)	C3—P1—C2—C21	178.3 (2)
C3—P1—C1—C12	178.2 (2)	Se1—P1—C2—C21	55.2 (3)
C2—P1—C1—C12	59.1 (3)	C1—P1—C3—C32	−178.4 (2)
Se1—P1—C1—C12	−62.8 (2)	C2—P1—C3—C32	−54.4 (3)
C3—P1—C1—C11	49.0 (3)	Se1—P1—C3—C32	67.9 (2)
C2—P1—C1—C11	−70.1 (3)	C1—P1—C3—C31	56.2 (3)
Se1—P1—C1—C11	167.9 (2)	C2—P1—C3—C31	−179.8 (2)
C1—P1—C2—C22	64.3 (3)	Se1—P1—C3—C31	−57.5 (2)
C3—P1—C2—C22	−54.3 (3)		

### Chlorido(tripropylphosphane selenide-κSe)gold (5a) Hydrogen-bond geometry (Å, º)

**Table:**

*D*—H···*A*	*D*—H	H···*A*	*D*···*A*	*D*—H···*A*
C32—H32*C*···Au1	0.98	2.91	3.700 (3)	138
C3—H3···Cl1^i^	1.00	2.81	3.715 (3)	151
C12—H12*B*···Au1^ii^	0.98	3.07	3.864 (3)	139
C1—H1···Se1^iii^	1.00	2.99	3.955 (3)	162

Symmetry codes: (i) −*x*+3/2, *y*+1/2, −*z*+1/2; (ii) −*x*+1, −*y*+1, −*z*+1; (iii) −*x*+2, −*y*+1, −*z*+1.

### [(tert-Butyl)bis(propan-2-yl)phosphane selenide-κSe]chloridogold (6a) Crystal data

**Table:**

[AuCl(C_10_H_23_PSe)]	*F*(000) = 912
*M**_r_* = 485.63	*D*_x_ = 2.270 Mg m^−^^3^
Monoclinic, *P*2_1_/*n*	Mo *K*α radiation, λ = 0.71073 Å
*a* = 8.2215 (2) Å	Cell parameters from 9950 reflections
*b* = 11.3519 (3) Å	θ = 2.2–30.7°
*c* = 15.2400 (4) Å	µ = 13.18 mm^−^^1^
β = 92.389 (4)°	*T* = 100 K
*V* = 1421.11 (6) Å^3^	Prism, colourless
*Z* = 4	0.35 × 0.2 × 0.2 mm

### [(tert-Butyl)bis(propan-2-yl)phosphane selenide-κSe]chloridogold (6a) Data collection

**Table:**

Oxford Diffraction Xcalibur, Eos diffractometer	4214 independent reflections
Radiation source: Enhance (Mo) X-ray Source	3424 reflections with *I* > 2σ(*I*)
Detector resolution: 16.1419 pixels mm^-1^	*R*_int_ = 0.038
ω scan	θ_max_ = 30.7°, θ_min_ = 2.2°
Absorption correction: multi-scan CrysAlisPro, Version 1.171.35.11 (Rigaku OD, 2020)	*h* = −11→11
*T*_min_ = 0.368, *T*_max_ = 1.000	*k* = −16→16
36891 measured reflections	*l* = −21→21

### [(tert-Butyl)bis(propan-2-yl)phosphane selenide-κSe]chloridogold (6a) Refinement

**Table:**

Refinement on *F*^2^	Secondary atom site location: difference Fourier map
Least-squares matrix: full	Hydrogen site location: inferred from neighbouring sites
*R*[*F*^2^ > 2σ(*F*^2^)] = 0.021	H-atom parameters constrained
*wR*(*F*^2^) = 0.044	*w* = 1/[σ^2^(*F*_o_^2^) + (0.0131*P*)^2^ + 2.1498*P*] where *P* = (*F*_o_^2^ + 2*F*_c_^2^)/3
*S* = 1.06	(Δ/σ)_max_ = 0.002
4214 reflections	Δρ_max_ = 1.74 e Å^−^^3^
135 parameters	Δρ_min_ = −1.12 e Å^−^^3^
0 restraints	Extinction correction: SHELXL-2019/3 (Sheldrick, 2015), Fc^*^=kFc[1+0.001xFc^2^λ^3^/sin(2θ)]^-1/4^
Primary atom site location: structure-invariant direct methods	Extinction coefficient: 0.00065 (4)

### [(tert-Butyl)bis(propan-2-yl)phosphane selenide-κSe]chloridogold (6a) Special details

**Table:**

Geometry. All esds (except the esd in the dihedral angle between two l.s. planes) are estimated using the full covariance matrix. The cell esds are taken into account individually in the estimation of esds in distances, angles and torsion angles; correlations between esds in cell parameters are only used when they are defined by crystal symmetry. An approximate (isotropic) treatment of cell esds is used for estimating esds involving l.s. planes.

### [(tert-Butyl)bis(propan-2-yl)phosphane selenide-κSe]chloridogold (6a) Fractional atomic coordinates and isotropic or equivalent isotropic displacement parameters (Å^2^)

**Table:**

	*x*	*y*	*z*	*U*_iso_*/*U*_eq_	
Au1	0.51576 (2)	0.50144 (2)	0.29309 (2)	0.01255 (4)	
Cl1	0.33329 (9)	0.48370 (6)	0.17636 (5)	0.01807 (14)	
P1	0.79619 (8)	0.68697 (6)	0.41430 (5)	0.00986 (13)	
Se1	0.69999 (4)	0.50672 (2)	0.41681 (2)	0.01570 (6)	
C1	0.6364 (3)	0.8032 (2)	0.40600 (19)	0.0133 (5)	
C2	0.9170 (3)	0.6949 (3)	0.51899 (18)	0.0148 (6)	
H2	0.985417	0.622101	0.520541	0.018*	
C3	0.9432 (3)	0.7041 (3)	0.32700 (19)	0.0146 (6)	
H3	0.975457	0.788926	0.324871	0.018*	
C11	0.7097 (4)	0.9209 (3)	0.4378 (2)	0.0178 (6)	
H11A	0.806381	0.938743	0.404695	0.027*	
H11B	0.740383	0.915402	0.500469	0.027*	
H11C	0.629081	0.983776	0.428440	0.027*	
C12	0.4927 (3)	0.7699 (3)	0.4627 (2)	0.0205 (6)	
H12A	0.410620	0.832538	0.459161	0.031*	
H12B	0.531760	0.760003	0.523888	0.031*	
H12C	0.444181	0.695975	0.441148	0.031*	
C13	0.5721 (4)	0.8178 (3)	0.31115 (19)	0.0172 (6)	
H13A	0.475687	0.868705	0.309668	0.026*	
H13B	0.542759	0.740508	0.286654	0.026*	
H13C	0.656489	0.853641	0.276264	0.026*	
C21	0.8138 (4)	0.6870 (3)	0.60037 (19)	0.0204 (6)	
H21A	0.884800	0.671673	0.652359	0.031*	
H21B	0.734833	0.622782	0.592713	0.031*	
H21C	0.755855	0.761514	0.608010	0.031*	
C22	1.0385 (3)	0.7976 (3)	0.5278 (2)	0.0180 (6)	
H22A	0.983896	0.866790	0.551372	0.027*	
H22B	1.079313	0.816571	0.469956	0.027*	
H22C	1.129773	0.774999	0.567673	0.027*	
C31	1.0967 (4)	0.6316 (3)	0.3482 (2)	0.0277 (8)	
H31A	1.068368	0.547809	0.350014	0.042*	
H31B	1.144308	0.655764	0.405436	0.042*	
H31C	1.175635	0.644833	0.302818	0.042*	
C32	0.8785 (4)	0.6686 (3)	0.23553 (19)	0.0201 (6)	
H32A	0.965110	0.677020	0.193707	0.030*	
H32B	0.786762	0.719573	0.217649	0.030*	
H32C	0.841984	0.586452	0.236451	0.030*	

### [(tert-Butyl)bis(propan-2-yl)phosphane selenide-κSe]chloridogold (6a) Atomic displacement parameters (Å^2^)

**Table:**

	*U*^11^	*U*^22^	*U*^33^	*U*^12^	*U*^13^	*U*^23^
Au1	0.01313 (6)	0.01068 (6)	0.01384 (6)	−0.00204 (4)	0.00049 (4)	−0.00166 (4)
Cl1	0.0184 (3)	0.0190 (3)	0.0165 (3)	−0.0009 (3)	−0.0025 (3)	−0.0028 (3)
P1	0.0106 (3)	0.0094 (3)	0.0096 (3)	0.0000 (3)	−0.0005 (2)	0.0002 (3)
Se1	0.01910 (14)	0.00999 (13)	0.01761 (15)	−0.00313 (11)	−0.00410 (11)	0.00246 (11)
C1	0.0131 (13)	0.0122 (13)	0.0145 (14)	0.0013 (10)	−0.0006 (11)	0.0002 (11)
C2	0.0153 (13)	0.0175 (14)	0.0112 (14)	0.0008 (11)	−0.0054 (11)	0.0015 (11)
C3	0.0143 (13)	0.0144 (13)	0.0154 (15)	−0.0028 (11)	0.0043 (11)	−0.0029 (11)
C11	0.0212 (15)	0.0131 (14)	0.0192 (16)	0.0011 (11)	−0.0001 (12)	−0.0006 (11)
C12	0.0146 (14)	0.0235 (16)	0.0235 (17)	0.0026 (12)	0.0029 (12)	0.0008 (13)
C13	0.0170 (14)	0.0162 (14)	0.0180 (15)	0.0048 (11)	−0.0053 (12)	0.0002 (11)
C21	0.0267 (16)	0.0220 (15)	0.0121 (15)	−0.0032 (13)	−0.0033 (12)	0.0025 (12)
C22	0.0148 (14)	0.0204 (15)	0.0185 (15)	−0.0020 (12)	−0.0043 (11)	−0.0031 (12)
C31	0.0192 (16)	0.039 (2)	0.0256 (19)	0.0066 (14)	0.0053 (14)	−0.0058 (15)
C32	0.0236 (15)	0.0244 (16)	0.0126 (15)	−0.0009 (13)	0.0045 (12)	−0.0013 (12)

### [(tert-Butyl)bis(propan-2-yl)phosphane selenide-κSe]chloridogold (6a) Geometric parameters (Å, º)

**Table:**

Au1—Cl1	2.2877 (7)	C12—H12A	0.9800
Au1—Se1	2.3696 (3)	C12—H12B	0.9800
P1—C3	1.845 (3)	C12—H12C	0.9800
P1—C2	1.846 (3)	C13—H13A	0.9800
P1—C1	1.863 (3)	C13—H13B	0.9800
P1—Se1	2.1947 (7)	C13—H13C	0.9800
C1—C13	1.527 (4)	C21—H21A	0.9800
C1—C11	1.536 (4)	C21—H21B	0.9800
C1—C12	1.540 (4)	C21—H21C	0.9800
C2—C21	1.534 (4)	C22—H22A	0.9800
C2—C22	1.538 (4)	C22—H22B	0.9800
C2—H2	1.0000	C22—H22C	0.9800
C3—C32	1.525 (4)	C31—H31A	0.9800
C3—C31	1.529 (4)	C31—H31B	0.9800
C3—H3	1.0000	C31—H31C	0.9800
C11—H11A	0.9800	C32—H32A	0.9800
C11—H11B	0.9800	C32—H32B	0.9800
C11—H11C	0.9800	C32—H32C	0.9800
			
Cl1—Au1—Se1	176.13 (2)	H12A—C12—H12B	109.5
C3—P1—C2	105.85 (13)	C1—C12—H12C	109.5
C3—P1—C1	110.98 (13)	H12A—C12—H12C	109.5
C2—P1—C1	112.11 (13)	H12B—C12—H12C	109.5
C3—P1—Se1	110.99 (10)	C1—C13—H13A	109.5
C2—P1—Se1	102.23 (10)	C1—C13—H13B	109.5
C1—P1—Se1	114.09 (9)	H13A—C13—H13B	109.5
P1—Se1—Au1	103.21 (2)	C1—C13—H13C	109.5
C13—C1—C11	108.7 (2)	H13A—C13—H13C	109.5
C13—C1—C12	108.3 (2)	H13B—C13—H13C	109.5
C11—C1—C12	109.7 (2)	C2—C21—H21A	109.5
C13—C1—P1	110.88 (19)	C2—C21—H21B	109.5
C11—C1—P1	109.18 (19)	H21A—C21—H21B	109.5
C12—C1—P1	110.1 (2)	C2—C21—H21C	109.5
C21—C2—C22	110.7 (2)	H21A—C21—H21C	109.5
C21—C2—P1	113.6 (2)	H21B—C21—H21C	109.5
C22—C2—P1	115.9 (2)	C2—C22—H22A	109.5
C21—C2—H2	105.2	C2—C22—H22B	109.5
C22—C2—H2	105.2	H22A—C22—H22B	109.5
P1—C2—H2	105.2	C2—C22—H22C	109.5
C32—C3—C31	107.7 (2)	H22A—C22—H22C	109.5
C32—C3—P1	114.7 (2)	H22B—C22—H22C	109.5
C31—C3—P1	110.5 (2)	C3—C31—H31A	109.5
C32—C3—H3	107.9	C3—C31—H31B	109.5
C31—C3—H3	107.9	H31A—C31—H31B	109.5
P1—C3—H3	107.9	C3—C31—H31C	109.5
C1—C11—H11A	109.5	H31A—C31—H31C	109.5
C1—C11—H11B	109.5	H31B—C31—H31C	109.5
H11A—C11—H11B	109.5	C3—C32—H32A	109.5
C1—C11—H11C	109.5	C3—C32—H32B	109.5
H11A—C11—H11C	109.5	H32A—C32—H32B	109.5
H11B—C11—H11C	109.5	C3—C32—H32C	109.5
C1—C12—H12A	109.5	H32A—C32—H32C	109.5
C1—C12—H12B	109.5	H32B—C32—H32C	109.5
			
C3—P1—Se1—Au1	−74.90 (10)	C3—P1—C2—C21	177.8 (2)
C2—P1—Se1—Au1	172.61 (10)	C1—P1—C2—C21	56.7 (2)
C1—P1—Se1—Au1	51.36 (10)	Se1—P1—C2—C21	−65.9 (2)
C3—P1—C1—C13	45.6 (2)	C3—P1—C2—C22	48.0 (2)
C2—P1—C1—C13	163.8 (2)	C1—P1—C2—C22	−73.1 (2)
Se1—P1—C1—C13	−80.6 (2)	Se1—P1—C2—C22	164.26 (19)
C3—P1—C1—C11	−74.1 (2)	C2—P1—C3—C32	164.8 (2)
C2—P1—C1—C11	44.1 (2)	C1—P1—C3—C32	−73.4 (2)
Se1—P1—C1—C11	159.67 (17)	Se1—P1—C3—C32	54.6 (2)
C3—P1—C1—C12	165.5 (2)	C2—P1—C3—C31	42.8 (2)
C2—P1—C1—C12	−76.4 (2)	C1—P1—C3—C31	164.6 (2)
Se1—P1—C1—C12	39.2 (2)	Se1—P1—C3—C31	−67.4 (2)

### [(tert-Butyl)bis(propan-2-yl)phosphane selenide-κSe]chloridogold (6a) Hydrogen-bond geometry (Å, º)

**Table:**

*D*—H···*A*	*D*—H	H···*A*	*D*···*A*	*D*—H···*A*
C13—H13*B*···Au1	0.98	2.73	3.630 (3)	154
C3—H3···Au1^i^	1.00	3.01	3.861 (3)	144
C3—H3···Cl1^i^	1.00	2.71	3.669 (3)	160
C2—H2···Se1^ii^	1.00	3.09	3.982 (3)	150
C13—H13*A*···Cl1^iii^	0.98	2.87	3.839 (3)	169
C22—H22*A*···Cl1^iv^	0.98	2.87	3.802 (3)	159

Symmetry codes: (i) −*x*+3/2, *y*+1/2, −*z*+1/2; (ii) −*x*+2, −*y*+1, −*z*+1; (iii) −*x*+1/2, *y*+1/2, −*z*+1/2; (iv) *x*+1/2, −*y*+3/2, *z*+1/2.

### [Bis(tert-butyl)(propan-2-yl)phosphane selenide-κSe]chloridogold (7a) Crystal data

**Table:**

[AuCl(C_11_H_25_PSe)]	*F*(000) = 944
*M**_r_* = 499.66	*D*_x_ = 2.161 Mg m^−^^3^
Monoclinic, *P*2_1_/*c*	Mo *K*α radiation, λ = 0.71073 Å
*a* = 7.64505 (10) Å	Cell parameters from 24794 reflections
*b* = 14.6437 (2) Å	θ = 2.7–30.8°
*c* = 13.7211 (2) Å	µ = 12.20 mm^−^^1^
β = 90.4954 (12)°	*T* = 100 K
*V* = 1536.05 (4) Å^3^	Plate, colourless
*Z* = 4	0.3 × 0.2 × 0.08 mm

### [Bis(tert-butyl)(propan-2-yl)phosphane selenide-κSe]chloridogold (7a) Data collection

**Table:**

Oxford Diffration Xcalibur, Eos diffractometer	4654 independent reflections
Radiation source: Enhance (Mo) X-ray Source	4270 reflections with *I* > 2σ(*I*)
Detector resolution: 16.1419 pixels mm^-1^	*R*_int_ = 0.047
ω scan	θ_max_ = 30.9°, θ_min_ = 2.7°
Absorption correction: multi-scan CrysAlisPro, Version 1.171.35.11 (Rigaku OD, 2020)	*h* = −10→10
*T*_min_ = 0.178, *T*_max_ = 1.000	*k* = −20→20
64129 measured reflections	*l* = −19→19

### [Bis(tert-butyl)(propan-2-yl)phosphane selenide-κSe]chloridogold (7a) Refinement

**Table:**

Refinement on *F*^2^	Secondary atom site location: difference Fourier map
Least-squares matrix: full	Hydrogen site location: inferred from neighbouring sites
*R*[*F*^2^ > 2σ(*F*^2^)] = 0.020	H-atom parameters constrained
*wR*(*F*^2^) = 0.042	*w* = 1/[σ^2^(*F*_o_^2^) + (0.0167*P*)^2^ + 2.0383*P*] where *P* = (*F*_o_^2^ + 2*F*_c_^2^)/3
*S* = 1.06	(Δ/σ)_max_ = 0.001
4654 reflections	Δρ_max_ = 2.04 e Å^−^^3^
145 parameters	Δρ_min_ = −1.36 e Å^−^^3^
0 restraints	Extinction correction: SHELXL-2019/3 (Sheldrick, 2015), Fc^*^=kFc[1+0.001xFc^2^λ^3^/sin(2θ)]^-1/4^
Primary atom site location: structure-invariant direct methods	Extinction coefficient: 0.00130 (6)

### [Bis(tert-butyl)(propan-2-yl)phosphane selenide-κSe]chloridogold (7a) Fractional atomic coordinates and isotropic or equivalent isotropic displacement parameters (Å^2^)

**Table:**

	*x*	*y*	*z*	*U*_iso_*/*U*_eq_	
Au1	0.12703 (2)	0.62141 (2)	0.08973 (2)	0.01679 (4)	
Cl1	0.15337 (9)	0.77264 (4)	0.05004 (5)	0.02287 (13)	
P1	0.26432 (7)	0.42241 (4)	0.23500 (4)	0.01046 (11)	
Se1	0.08348 (3)	0.46295 (2)	0.11688 (2)	0.01764 (6)	
C1	0.2385 (3)	0.49681 (16)	0.34533 (18)	0.0157 (5)	
C2	0.4922 (3)	0.41782 (17)	0.18606 (19)	0.0178 (5)	
C3	0.1824 (3)	0.30857 (16)	0.27179 (19)	0.0187 (5)	
H3	0.086991	0.321412	0.319288	0.022*	
C11	0.3050 (4)	0.4493 (2)	0.4383 (2)	0.0256 (6)	
H11A	0.426791	0.430479	0.429488	0.038*	
H11B	0.232742	0.395501	0.451376	0.038*	
H11C	0.297788	0.491755	0.493370	0.038*	
C12	0.0418 (3)	0.51502 (17)	0.3594 (2)	0.0189 (5)	
H12A	0.025236	0.553189	0.417219	0.028*	
H12B	−0.019542	0.456852	0.367911	0.028*	
H12C	−0.005278	0.546595	0.301899	0.028*	
C13	0.3335 (4)	0.58828 (18)	0.3330 (2)	0.0234 (6)	
H13A	0.302037	0.629288	0.386532	0.035*	
H13B	0.298923	0.615991	0.270763	0.035*	
H13C	0.460131	0.577997	0.333924	0.035*	
C21	0.6285 (4)	0.3994 (3)	0.2661 (3)	0.0361 (8)	
H21A	0.744569	0.393917	0.236878	0.054*	
H21B	0.599553	0.342403	0.299840	0.054*	
H21C	0.628745	0.449941	0.312897	0.054*	
C22	0.4953 (4)	0.34047 (19)	0.1099 (2)	0.0325 (7)	
H22A	0.402133	0.350485	0.061589	0.049*	
H22B	0.476918	0.281716	0.142451	0.049*	
H22C	0.608830	0.340036	0.077301	0.049*	
C23	0.5391 (3)	0.50705 (18)	0.1344 (2)	0.0201 (5)	
H23A	0.540991	0.557103	0.181804	0.030*	
H23B	0.451620	0.519995	0.083621	0.030*	
H23C	0.654649	0.501173	0.104697	0.030*	
C31	0.3123 (4)	0.24696 (19)	0.3274 (3)	0.0340 (7)	
H31A	0.249080	0.195800	0.356711	0.051*	
H31B	0.370693	0.282460	0.378704	0.051*	
H31C	0.399812	0.223407	0.282055	0.051*	
C32	0.0945 (5)	0.2535 (2)	0.1900 (2)	0.0347 (7)	
H32A	0.183385	0.232432	0.144179	0.052*	
H32B	0.009513	0.292198	0.155597	0.052*	
H32C	0.034243	0.200684	0.217962	0.052*	

### [Bis(tert-butyl)(propan-2-yl)phosphane selenide-κSe]chloridogold (7a) Atomic displacement parameters (Å^2^)

**Table:**

	*U*^11^	*U*^22^	*U*^33^	*U*^12^	*U*^13^	*U*^23^
Au1	0.01726 (5)	0.02062 (6)	0.01251 (5)	0.00498 (3)	0.00192 (3)	0.00362 (3)
Cl1	0.0321 (3)	0.0194 (3)	0.0172 (3)	0.0056 (2)	0.0043 (2)	0.0006 (2)
P1	0.0110 (3)	0.0095 (2)	0.0109 (3)	−0.00019 (19)	0.0013 (2)	0.0001 (2)
Se1	0.01578 (11)	0.02326 (13)	0.01381 (12)	−0.00408 (9)	−0.00414 (8)	0.00283 (9)
C1	0.0191 (11)	0.0168 (11)	0.0114 (11)	−0.0044 (9)	0.0015 (8)	−0.0032 (9)
C2	0.0113 (10)	0.0212 (12)	0.0211 (13)	0.0034 (9)	0.0050 (9)	0.0050 (10)
C3	0.0246 (12)	0.0106 (10)	0.0211 (13)	−0.0015 (9)	0.0074 (10)	0.0020 (9)
C11	0.0254 (14)	0.0366 (16)	0.0147 (13)	−0.0062 (11)	−0.0017 (10)	0.0033 (11)
C12	0.0218 (12)	0.0153 (11)	0.0198 (13)	0.0019 (9)	0.0083 (9)	−0.0028 (9)
C13	0.0316 (14)	0.0198 (12)	0.0190 (13)	−0.0115 (10)	0.0046 (11)	−0.0060 (10)
C21	0.0127 (12)	0.059 (2)	0.0367 (18)	0.0078 (13)	0.0029 (11)	0.0220 (16)
C22	0.0385 (17)	0.0195 (13)	0.0401 (19)	0.0061 (12)	0.0241 (14)	−0.0013 (12)
C23	0.0166 (11)	0.0216 (12)	0.0223 (13)	−0.0033 (9)	0.0069 (9)	0.0015 (10)
C31	0.0384 (17)	0.0185 (13)	0.045 (2)	0.0126 (12)	0.0176 (14)	0.0166 (13)
C32	0.053 (2)	0.0179 (13)	0.0329 (17)	−0.0162 (13)	0.0126 (14)	−0.0085 (12)

### [Bis(tert-butyl)(propan-2-yl)phosphane selenide-κSe]chloridogold (7a) Geometric parameters (Å, º)

**Table:**

Au1—Cl1	2.2898 (6)	C12—H12C	0.9800
Au1—Se1	2.3740 (3)	C13—H13A	0.9800
P1—C3	1.853 (2)	C13—H13B	0.9800
P1—C2	1.874 (2)	C13—H13C	0.9800
P1—C1	1.877 (2)	C21—H21A	0.9800
P1—Se1	2.2027 (6)	C21—H21B	0.9800
C1—C13	1.534 (3)	C21—H21C	0.9800
C1—C11	1.535 (4)	C22—H22A	0.9800
C1—C12	1.540 (3)	C22—H22B	0.9800
C2—C23	1.531 (3)	C22—H22C	0.9800
C2—C21	1.532 (4)	C23—H23A	0.9800
C2—C22	1.541 (4)	C23—H23B	0.9800
C3—C32	1.532 (4)	C23—H23C	0.9800
C3—C31	1.539 (4)	C31—H31A	0.9800
C3—H3	1.0000	C31—H31B	0.9800
C11—H11A	0.9800	C31—H31C	0.9800
C11—H11B	0.9800	C32—H32A	0.9800
C11—H11C	0.9800	C32—H32B	0.9800
C12—H12A	0.9800	C32—H32C	0.9800
C12—H12B	0.9800		
			
Cl1—Au1—Se1	174.465 (18)	H12B—C12—H12C	109.5
C3—P1—C2	112.52 (11)	C1—C13—H13A	109.5
C3—P1—C1	105.33 (11)	C1—C13—H13B	109.5
C2—P1—C1	114.50 (12)	H13A—C13—H13B	109.5
C3—P1—Se1	103.38 (9)	C1—C13—H13C	109.5
C2—P1—Se1	109.00 (8)	H13A—C13—H13C	109.5
C1—P1—Se1	111.54 (8)	H13B—C13—H13C	109.5
P1—Se1—Au1	106.911 (18)	C2—C21—H21A	109.5
C13—C1—C11	109.5 (2)	C2—C21—H21B	109.5
C13—C1—C12	109.0 (2)	H21A—C21—H21B	109.5
C11—C1—C12	106.9 (2)	C2—C21—H21C	109.5
C13—C1—P1	111.40 (17)	H21A—C21—H21C	109.5
C11—C1—P1	111.77 (18)	H21B—C21—H21C	109.5
C12—C1—P1	108.12 (16)	C2—C22—H22A	109.5
C23—C2—C21	108.8 (2)	C2—C22—H22B	109.5
C23—C2—C22	108.0 (2)	H22A—C22—H22B	109.5
C21—C2—C22	110.0 (2)	C2—C22—H22C	109.5
C23—C2—P1	110.95 (16)	H22A—C22—H22C	109.5
C21—C2—P1	112.23 (19)	H22B—C22—H22C	109.5
C22—C2—P1	106.81 (18)	C2—C23—H23A	109.5
C32—C3—C31	109.4 (2)	C2—C23—H23B	109.5
C32—C3—P1	114.91 (19)	H23A—C23—H23B	109.5
C31—C3—P1	116.4 (2)	C2—C23—H23C	109.5
C32—C3—H3	105.0	H23A—C23—H23C	109.5
C31—C3—H3	105.0	H23B—C23—H23C	109.5
P1—C3—H3	105.0	C3—C31—H31A	109.5
C1—C11—H11A	109.5	C3—C31—H31B	109.5
C1—C11—H11B	109.5	H31A—C31—H31B	109.5
H11A—C11—H11B	109.5	C3—C31—H31C	109.5
C1—C11—H11C	109.5	H31A—C31—H31C	109.5
H11A—C11—H11C	109.5	H31B—C31—H31C	109.5
H11B—C11—H11C	109.5	C3—C32—H32A	109.5
C1—C12—H12A	109.5	C3—C32—H32B	109.5
C1—C12—H12B	109.5	H32A—C32—H32B	109.5
H12A—C12—H12B	109.5	C3—C32—H32C	109.5
C1—C12—H12C	109.5	H32A—C32—H32C	109.5
H12A—C12—H12C	109.5	H32B—C32—H32C	109.5
			
C3—P1—Se1—Au1	166.02 (8)	Se1—P1—C2—C23	52.4 (2)
C2—P1—Se1—Au1	−74.09 (9)	C3—P1—C2—C21	−71.6 (2)
C1—P1—Se1—Au1	53.31 (8)	C1—P1—C2—C21	48.6 (2)
C3—P1—C1—C13	168.86 (19)	Se1—P1—C2—C21	174.31 (19)
C2—P1—C1—C13	44.7 (2)	C3—P1—C2—C22	49.0 (2)
Se1—P1—C1—C13	−79.65 (19)	C1—P1—C2—C22	169.23 (18)
C3—P1—C1—C11	46.1 (2)	Se1—P1—C2—C22	−65.06 (19)
C2—P1—C1—C11	−78.1 (2)	C2—P1—C3—C32	−87.3 (2)
Se1—P1—C1—C11	157.55 (15)	C1—P1—C3—C32	147.3 (2)
C3—P1—C1—C12	−71.34 (19)	Se1—P1—C3—C32	30.1 (2)
C2—P1—C1—C12	164.51 (16)	C2—P1—C3—C31	42.3 (2)
Se1—P1—C1—C12	40.14 (18)	C1—P1—C3—C31	−83.0 (2)
C3—P1—C2—C23	166.43 (18)	Se1—P1—C3—C31	159.79 (19)
C1—P1—C2—C23	−73.3 (2)		

### [Bis(tert-butyl)(propan-2-yl)phosphane selenide-κSe]chloridogold (7a) Hydrogen-bond geometry (Å, º)

**Table:**

*D*—H···*A*	*D*—H	H···*A*	*D*···*A*	*D*—H···*A*
C13—H13*B*···Au1	0.98	2.80	3.712 (3)	155
C23—H23*B*···Au1	0.98	2.89	3.615 (3)	131
C32—H32*B*···Se1	0.98	2.62	3.228 (3)	120
C3—H3···Cl1^i^	1.00	2.68	3.599 (3)	154

Symmetry code: (i) −*x*, *y*−1/2, −*z*+1/2.

### Bromido(tripropylphosphane sulfide-κS)gold (1b) Crystal data

**Table:**

[AuBr(C_9_H_21_PS)]	*F*(000) = 880
*M**_r_* = 469.16	*D*_x_ = 2.250 Mg m^−^^3^
Monoclinic, *P*2_1_/*n*	Mo *K*α radiation, λ = 0.71073 Å
*a* = 8.1898 (2) Å	Cell parameters from 13927 reflections
*b* = 11.1421 (3) Å	θ = 2.3–30.8°
*c* = 15.3064 (4) Å	µ = 13.73 mm^−^^1^
β = 97.394 (2)°	*T* = 100 K
*V* = 1385.11 (6) Å^3^	Block, colourless
*Z* = 4	0.13 × 0.08 × 0.06 mm

### Bromido(tripropylphosphane sulfide-κS)gold (1b) Data collection

**Table:**

Oxford Diffraction Xcalibur, Eos diffractometer	4194 independent reflections
Radiation source: Enhance (Mo) X-ray Source	3563 reflections with *I* > 2σ(*I*)
Detector resolution: 16.1419 pixels mm^-1^	*R*_int_ = 0.040
ω scan	θ_max_ = 30.9°, θ_min_ = 2.3°
Absorption correction: multi-scan CrysAlisPro, Version 1.171.35.21 (Rigaku OD, 2020)	*h* = −11→11
*T*_min_ = 0.568, *T*_max_ = 1.000	*k* = −15→15
51956 measured reflections	*l* = −21→21

### Bromido(tripropylphosphane sulfide-κS)gold (1b) Refinement

**Table:**

Refinement on *F*^2^	Secondary atom site location: difference Fourier map
Least-squares matrix: full	Hydrogen site location: inferred from neighbouring sites
*R*[*F*^2^ > 2σ(*F*^2^)] = 0.019	H-atom parameters constrained
*wR*(*F*^2^) = 0.032	*w* = 1/[σ^2^(*F*_o_^2^) + (0.0096*P*)^2^ + 0.8742*P*] where *P* = (*F*_o_^2^ + 2*F*_c_^2^)/3
*S* = 1.06	(Δ/σ)_max_ = 0.003
4194 reflections	Δρ_max_ = 0.88 e Å^−^^3^
125 parameters	Δρ_min_ = −0.79 e Å^−^^3^
0 restraints	Extinction correction: SHELXL-2019/3 (Sheldrick, 2015), Fc^*^=kFc[1+0.001xFc^2^λ^3^/sin(2θ)]^-1/4^
Primary atom site location: structure-invariant direct methods	Extinction coefficient: 0.00097 (4)

### Bromido(tripropylphosphane sulfide-κS)gold (1b) Fractional atomic coordinates and isotropic or equivalent isotropic displacement parameters (Å^2^)

**Table:**

	*x*	*y*	*z*	*U*_iso_*/*U*_eq_	
Au1	0.50533 (2)	0.50190 (2)	0.30576 (2)	0.01676 (3)	
Br1	0.29839 (3)	0.46987 (2)	0.18238 (2)	0.02092 (5)	
P1	0.78167 (7)	0.68773 (5)	0.42436 (4)	0.01290 (11)	
S1	0.70072 (7)	0.51535 (5)	0.42538 (4)	0.02204 (12)	
C1	0.9233 (3)	0.7019 (2)	0.52658 (14)	0.0160 (4)	
H1	1.000532	0.632291	0.527387	0.019*	
C2	0.6094 (3)	0.7937 (2)	0.41329 (15)	0.0178 (5)	
H2	0.546289	0.778780	0.353912	0.021*	
C3	0.9023 (3)	0.7181 (2)	0.33447 (15)	0.0180 (5)	
H3	0.948869	0.800761	0.344034	0.022*	
C11	1.0320 (3)	0.8151 (2)	0.53096 (15)	0.0211 (5)	
H11A	0.965107	0.885742	0.540361	0.032*	
H11B	1.078683	0.824040	0.475503	0.032*	
H11C	1.121297	0.807548	0.579808	0.032*	
C12	0.8390 (3)	0.6899 (2)	0.60990 (15)	0.0236 (5)	
H12A	0.922421	0.676291	0.660851	0.035*	
H12B	0.762492	0.621870	0.603353	0.035*	
H12C	0.778233	0.763674	0.618800	0.035*	
C21	0.4881 (3)	0.7726 (3)	0.48039 (18)	0.0314 (6)	
H21A	0.538766	0.797892	0.538983	0.047*	
H21B	0.460417	0.687066	0.481676	0.047*	
H21C	0.387629	0.819273	0.463323	0.047*	
C22	0.6653 (3)	0.9251 (2)	0.41341 (19)	0.0278 (6)	
H22A	0.569370	0.976972	0.397751	0.042*	
H22B	0.742905	0.935655	0.370351	0.042*	
H22C	0.719264	0.946535	0.472219	0.042*	
C31	1.0483 (3)	0.6317 (3)	0.33672 (18)	0.0313 (6)	
H31A	1.007398	0.549818	0.325031	0.047*	
H31B	1.113945	0.634750	0.394920	0.047*	
H31C	1.116990	0.655109	0.291645	0.047*	
C32	0.7990 (3)	0.7174 (2)	0.24383 (15)	0.0263 (6)	
H32A	0.870281	0.733177	0.198351	0.039*	
H32B	0.714240	0.779769	0.241698	0.039*	
H32C	0.746443	0.638814	0.233359	0.039*	

### Bromido(tripropylphosphane sulfide-κS)gold (1b) Atomic displacement parameters (Å^2^)

**Table:**

	*U*^11^	*U*^22^	*U*^33^	*U*^12^	*U*^13^	*U*^23^
Au1	0.01631 (5)	0.01400 (5)	0.01994 (5)	−0.00208 (3)	0.00219 (3)	−0.00182 (3)
Br1	0.02089 (11)	0.02406 (12)	0.01774 (11)	−0.00058 (9)	0.00220 (8)	−0.00462 (9)
P1	0.0126 (3)	0.0119 (3)	0.0137 (3)	0.0002 (2)	−0.0004 (2)	0.0010 (2)
S1	0.0227 (3)	0.0127 (3)	0.0286 (3)	−0.0025 (2)	−0.0049 (2)	0.0037 (2)
C1	0.0152 (11)	0.0180 (11)	0.0140 (11)	0.0008 (8)	−0.0012 (8)	0.0026 (8)
C2	0.0133 (10)	0.0170 (11)	0.0224 (12)	0.0025 (8)	−0.0008 (9)	0.0042 (9)
C3	0.0172 (11)	0.0199 (12)	0.0171 (11)	−0.0014 (9)	0.0031 (9)	−0.0018 (9)
C11	0.0196 (12)	0.0282 (13)	0.0146 (11)	−0.0055 (10)	−0.0013 (9)	0.0013 (10)
C12	0.0287 (13)	0.0281 (13)	0.0136 (11)	−0.0065 (10)	0.0014 (10)	0.0038 (9)
C21	0.0206 (13)	0.0337 (15)	0.0421 (18)	0.0100 (11)	0.0123 (12)	0.0102 (12)
C22	0.0226 (13)	0.0150 (12)	0.0453 (17)	0.0048 (10)	0.0021 (11)	0.0029 (11)
C31	0.0296 (14)	0.0386 (16)	0.0276 (14)	0.0098 (12)	0.0106 (11)	−0.0014 (12)
C32	0.0335 (14)	0.0293 (14)	0.0157 (12)	−0.0105 (11)	0.0012 (10)	−0.0006 (10)

### Bromido(tripropylphosphane sulfide-κS)gold (1b) Geometric parameters (Å, º)

**Table:**

Au1—S1	2.2763 (6)	C11—H11C	0.9800
Au1—Br1	2.3963 (3)	C12—H12A	0.9800
P1—C3	1.826 (2)	C12—H12B	0.9800
P1—C2	1.831 (2)	C12—H12C	0.9800
P1—C1	1.831 (2)	C21—H21A	0.9800
P1—S1	2.0325 (8)	C21—H21B	0.9800
C1—C12	1.532 (3)	C21—H21C	0.9800
C1—C11	1.540 (3)	C22—H22A	0.9800
C1—H1	1.0000	C22—H22B	0.9800
C2—C22	1.534 (3)	C22—H22C	0.9800
C2—C21	1.536 (3)	C31—H31A	0.9800
C2—H2	1.0000	C31—H31B	0.9800
C3—C32	1.529 (3)	C31—H31C	0.9800
C3—C31	1.533 (3)	C32—H32A	0.9800
C3—H3	1.0000	C32—H32B	0.9800
C11—H11A	0.9800	C32—H32C	0.9800
C11—H11B	0.9800		
			
S1—Au1—Br1	175.134 (15)	H11B—C11—H11C	109.5
C3—P1—C2	107.21 (10)	C1—C12—H12A	109.5
C3—P1—C1	106.45 (10)	C1—C12—H12B	109.5
C2—P1—C1	114.79 (11)	H12A—C12—H12B	109.5
C3—P1—S1	112.94 (8)	C1—C12—H12C	109.5
C2—P1—S1	111.22 (8)	H12A—C12—H12C	109.5
C1—P1—S1	104.27 (8)	H12B—C12—H12C	109.5
P1—S1—Au1	104.61 (3)	C2—C21—H21A	109.5
C12—C1—C11	110.82 (19)	C2—C21—H21B	109.5
C12—C1—P1	113.58 (15)	H21A—C21—H21B	109.5
C11—C1—P1	114.01 (15)	C2—C21—H21C	109.5
C12—C1—H1	105.9	H21A—C21—H21C	109.5
C11—C1—H1	105.9	H21B—C21—H21C	109.5
P1—C1—H1	105.9	C2—C22—H22A	109.5
C22—C2—C21	111.3 (2)	C2—C22—H22B	109.5
C22—C2—P1	112.91 (15)	H22A—C22—H22B	109.5
C21—C2—P1	113.35 (16)	C2—C22—H22C	109.5
C22—C2—H2	106.2	H22A—C22—H22C	109.5
C21—C2—H2	106.2	H22B—C22—H22C	109.5
P1—C2—H2	106.2	C3—C31—H31A	109.5
C32—C3—C31	110.8 (2)	C3—C31—H31B	109.5
C32—C3—P1	113.14 (16)	H31A—C31—H31B	109.5
C31—C3—P1	111.27 (17)	C3—C31—H31C	109.5
C32—C3—H3	107.1	H31A—C31—H31C	109.5
C31—C3—H3	107.1	H31B—C31—H31C	109.5
P1—C3—H3	107.1	C3—C32—H32A	109.5
C1—C11—H11A	109.5	C3—C32—H32B	109.5
C1—C11—H11B	109.5	H32A—C32—H32B	109.5
H11A—C11—H11B	109.5	C3—C32—H32C	109.5
C1—C11—H11C	109.5	H32A—C32—H32C	109.5
H11A—C11—H11C	109.5	H32B—C32—H32C	109.5
			
C3—P1—S1—Au1	−70.35 (8)	S1—P1—C2—C22	179.27 (16)
C2—P1—S1—Au1	50.25 (9)	C3—P1—C2—C21	175.40 (18)
C1—P1—S1—Au1	174.50 (8)	C1—P1—C2—C21	−66.6 (2)
C3—P1—C1—C12	175.00 (17)	S1—P1—C2—C21	51.48 (19)
C2—P1—C1—C12	56.6 (2)	C2—P1—C3—C32	−53.2 (2)
S1—P1—C1—C12	−65.37 (17)	C1—P1—C3—C32	−176.49 (17)
C3—P1—C1—C11	46.76 (19)	S1—P1—C3—C32	69.68 (18)
C2—P1—C1—C11	−71.68 (19)	C2—P1—C3—C31	−178.74 (17)
S1—P1—C1—C11	166.39 (15)	C1—P1—C3—C31	57.95 (19)
C3—P1—C2—C22	−56.8 (2)	S1—P1—C3—C31	−55.89 (18)
C1—P1—C2—C22	61.2 (2)		

### Bromido(tripropylphosphane sulfide-κS)gold (1b) Hydrogen-bond geometry (Å, º)

**Table:**

*D*—H···*A*	*D*—H	H···*A*	*D*···*A*	*D*—H···*A*
C3—H3···Br1^i^	1.00	2.87	3.756 (2)	149
C21—H21*C*···Br1^ii^	0.98	3.04	3.878 (3)	144
C32—H32*C*···Au1	0.98	2.83	3.611 (2)	137
C1—H1···S1^iii^	1.00	2.96	3.911 (2)	159
C12—H12*B*···S1	0.98	2.95	3.495 (2)	116
C21—H21*B*···S1	0.98	2.95	3.512 (3)	117

Symmetry codes: (i) −*x*+3/2, *y*+1/2, −*z*+1/2; (ii) −*x*+1/2, *y*+1/2, −*z*+1/2; (iii) −*x*+2, −*y*+1, −*z*+1.

### [Bis(tert-butyl)(propan-2-yl)tripropylphosphane sulfide-κS]bromidogold (3b) Crystal data

**Table:**

[AuBr(C_11_H_25_PS)]	*F*(000) = 1888
*M**_r_* = 497.22	*D*_x_ = 2.121 Mg m^−^^3^*D*_m_ = 2.121 Mg m^−^^3^*D*_m_ measured by ?
Monoclinic, *C*2/*c*	Mo *K*α radiation, λ = 0.71073 Å
*a* = 27.3157 (10) Å	Cell parameters from 37811 reflections
*b* = 8.16931 (13) Å	θ = 2.7–30.7°
*c* = 18.8362 (7) Å	µ = 12.22 mm^−^^1^
β = 132.187 (7)°	*T* = 101 K
*V* = 3114.5 (3) Å^3^	Tablet, colourless
*Z* = 8	0.3 × 0.2 × 0.1 mm

### [Bis(tert-butyl)(propan-2-yl)tripropylphosphane sulfide-κS]bromidogold (3b) Data collection

**Table:**

Oxford Diffraction Xcalibur, Eos diffractometer	4712 independent reflections
Radiation source: fine-focus sealed X-ray tube	4383 reflections with *I* > 2σ(*I*)
Detector resolution: 16.1419 pixels mm^-1^	*R*_int_ = 0.034
ω scans	θ_max_ = 30.9°, θ_min_ = 2.2°
Absorption correction: multi-scan CrysAlisPro, Version 1.171.41.93a (Rigaku OD, 2020)	*h* = −39→39
*T*_min_ = 0.300, *T*_max_ = 1.000	*k* = −11→11
72942 measured reflections	*l* = −27→26

### [Bis(tert-butyl)(propan-2-yl)tripropylphosphane sulfide-κS]bromidogold (3b) Refinement

**Table:**

Refinement on *F*^2^	Hydrogen site location: inferred from neighbouring sites
Least-squares matrix: full	H-atom parameters constrained
*R*[*F*^2^ > 2σ(*F*^2^)] = 0.015	*w* = 1/[σ^2^(*F*_o_^2^) + (0.0119*P*)^2^ + 6.187*P*] where *P* = (*F*_o_^2^ + 2*F*_c_^2^)/3
*wR*(*F*^2^) = 0.032	(Δ/σ)_max_ = 0.003
*S* = 1.08	Δρ_max_ = 1.22 e Å^−^^3^
4712 reflections	Δρ_min_ = −0.87 e Å^−^^3^
145 parameters	Extinction correction: SHELXL-2019/3 (Sheldrick, 2015), Fc^*^=kFc[1+0.001xFc^2^λ^3^/sin(2θ)]^-1/4^
0 restraints	Extinction coefficient: 0.000358 (12)
Primary atom site location: structure-invariant direct methods	

### [Bis(tert-butyl)(propan-2-yl)tripropylphosphane sulfide-κS]bromidogold (3b) Special details

**Table:**

Geometry. All esds (except the esd in the dihedral angle between two l.s. planes) are estimated using the full covariance matrix. The cell esds are taken into account individually in the estimation of esds in distances, angles and torsion angles; correlations between esds in cell parameters are only used when they are defined by crystal symmetry. An approximate (isotropic) treatment of cell esds is used for estimating esds involving l.s. planes.

### [Bis(tert-butyl)(propan-2-yl)tripropylphosphane sulfide-κS]bromidogold (3b) Fractional atomic coordinates and isotropic or equivalent isotropic displacement parameters (Å^2^)

**Table:**

	*x*	*y*	*z*	*U*_iso_*/*U*_eq_	
Au1	0.32867 (2)	0.54617 (2)	0.46731 (2)	0.01580 (3)	
Br1	0.42769 (2)	0.38744 (3)	0.55782 (2)	0.02257 (5)	
S1	0.24093 (2)	0.71919 (6)	0.38834 (3)	0.01798 (9)	
P1	0.15993 (2)	0.58893 (6)	0.27968 (3)	0.01231 (9)	
C1	0.14221 (10)	0.4248 (2)	0.32985 (14)	0.0173 (4)	
C2	0.09309 (10)	0.7473 (2)	0.20816 (14)	0.0177 (4)	
C3	0.17765 (9)	0.4863 (2)	0.21154 (13)	0.0153 (4)	
H3	0.215861	0.412413	0.258958	0.018*	
C11	0.07062 (11)	0.3644 (3)	0.25725 (16)	0.0252 (4)	
H11A	0.060425	0.326571	0.199184	0.038*	
H11B	0.040780	0.454166	0.240966	0.038*	
H11C	0.064838	0.273809	0.285166	0.038*	
C12	0.15778 (12)	0.4879 (3)	0.42027 (16)	0.0253 (4)	
H12A	0.130189	0.583490	0.403761	0.038*	
H12B	0.204491	0.518804	0.468571	0.038*	
H12C	0.148574	0.401402	0.445985	0.038*	
C13	0.18831 (10)	0.2781 (2)	0.36103 (15)	0.0219 (4)	
H13A	0.181703	0.195520	0.391663	0.033*	
H13B	0.234354	0.315171	0.406539	0.033*	
H13C	0.178197	0.230266	0.304720	0.033*	
C21	0.11876 (11)	0.8966 (3)	0.19185 (16)	0.0244 (4)	
H21A	0.083550	0.978414	0.153674	0.037*	
H21B	0.132552	0.862361	0.157663	0.037*	
H21C	0.156551	0.944090	0.253814	0.037*	
C22	0.03174 (11)	0.6830 (3)	0.10901 (15)	0.0272 (5)	
H22A	0.018189	0.578048	0.116412	0.041*	
H22B	0.042329	0.667768	0.068989	0.041*	
H22C	−0.004373	0.762167	0.078498	0.041*	
C23	0.07463 (11)	0.8058 (3)	0.26499 (17)	0.0279 (5)	
H23A	0.114592	0.841094	0.328977	0.042*	
H23B	0.053782	0.715914	0.270861	0.042*	
H23C	0.043722	0.897799	0.231420	0.042*	
C31	0.20259 (11)	0.6028 (3)	0.17818 (17)	0.0241 (4)	
H31A	0.221273	0.539096	0.157105	0.036*	
H31B	0.236731	0.674458	0.231285	0.036*	
H31C	0.165768	0.669313	0.124771	0.036*	
C32	0.12394 (11)	0.3737 (3)	0.12933 (15)	0.0249 (4)	
H32A	0.085439	0.439182	0.078105	0.037*	
H32B	0.110949	0.293954	0.152946	0.037*	
H32C	0.141085	0.315860	0.104324	0.037*	

### [Bis(tert-butyl)(propan-2-yl)tripropylphosphane sulfide-κS]bromidogold (3b) Atomic displacement parameters (Å^2^)

**Table:**

	*U*^11^	*U*^22^	*U*^33^	*U*^12^	*U*^13^	*U*^23^
Au1	0.01264 (4)	0.01862 (4)	0.01150 (4)	−0.00495 (3)	0.00620 (3)	−0.00108 (3)
Br1	0.01514 (9)	0.02561 (10)	0.01862 (9)	−0.00098 (7)	0.00792 (8)	0.00264 (8)
S1	0.0155 (2)	0.0156 (2)	0.0152 (2)	−0.00548 (17)	0.00713 (18)	−0.00504 (17)
P1	0.0118 (2)	0.0117 (2)	0.0116 (2)	−0.00213 (16)	0.00715 (18)	−0.00276 (16)
C1	0.0203 (9)	0.0166 (9)	0.0172 (9)	−0.0051 (7)	0.0135 (8)	−0.0030 (7)
C2	0.0157 (9)	0.0179 (9)	0.0146 (8)	0.0015 (7)	0.0082 (8)	−0.0027 (7)
C3	0.0152 (8)	0.0146 (8)	0.0156 (8)	−0.0001 (7)	0.0101 (8)	−0.0022 (7)
C11	0.0207 (10)	0.0295 (11)	0.0258 (11)	−0.0100 (9)	0.0158 (9)	−0.0034 (9)
C12	0.0346 (12)	0.0252 (10)	0.0217 (10)	−0.0078 (9)	0.0212 (10)	−0.0045 (8)
C13	0.0232 (10)	0.0170 (9)	0.0230 (10)	−0.0039 (8)	0.0145 (9)	−0.0002 (8)
C21	0.0284 (11)	0.0152 (9)	0.0281 (11)	0.0047 (8)	0.0183 (10)	0.0033 (8)
C22	0.0175 (10)	0.0258 (11)	0.0211 (10)	0.0019 (8)	0.0060 (9)	−0.0020 (8)
C23	0.0266 (11)	0.0322 (12)	0.0282 (11)	0.0095 (9)	0.0199 (10)	0.0008 (9)
C31	0.0300 (11)	0.0234 (10)	0.0312 (11)	0.0000 (9)	0.0256 (10)	−0.0002 (9)
C32	0.0232 (10)	0.0296 (11)	0.0207 (10)	−0.0062 (9)	0.0142 (9)	−0.0122 (9)

### [Bis(tert-butyl)(propan-2-yl)tripropylphosphane sulfide-κS]bromidogold (3b) Geometric parameters (Å, º)

**Table:**

Au1—S1	2.2731 (5)	C12—H12C	0.9800
Au1—Br1	2.3896 (3)	C13—H13A	0.9800
S1—P1	2.0333 (7)	C13—H13B	0.9800
P1—C3	1.849 (2)	C13—H13C	0.9800
P1—C2	1.876 (2)	C21—H21A	0.9800
P1—C1	1.881 (2)	C21—H21B	0.9800
C1—C11	1.532 (3)	C21—H21C	0.9800
C1—C13	1.540 (3)	C22—H22A	0.9800
C1—C12	1.541 (3)	C22—H22B	0.9800
C2—C23	1.534 (3)	C22—H22C	0.9800
C2—C21	1.537 (3)	C23—H23A	0.9800
C2—C22	1.539 (3)	C23—H23B	0.9800
C3—C31	1.529 (3)	C23—H23C	0.9800
C3—C32	1.532 (3)	C31—H31A	0.9800
C3—H3	1.0000	C31—H31B	0.9800
C11—H11A	0.9800	C31—H31C	0.9800
C11—H11B	0.9800	C32—H32A	0.9800
C11—H11C	0.9800	C32—H32B	0.9800
C12—H12A	0.9800	C32—H32C	0.9800
C12—H12B	0.9800		
			
S1—Au1—Br1	174.382 (13)	H12B—C12—H12C	109.5
P1—S1—Au1	106.84 (3)	C1—C13—H13A	109.5
C3—P1—C2	113.35 (9)	C1—C13—H13B	109.5
C3—P1—C1	107.53 (9)	H13A—C13—H13B	109.5
C2—P1—C1	113.42 (9)	C1—C13—H13C	109.5
C3—P1—S1	108.72 (6)	H13A—C13—H13C	109.5
C2—P1—S1	103.79 (6)	H13B—C13—H13C	109.5
C1—P1—S1	109.91 (7)	C2—C21—H21A	109.5
C11—C1—C13	108.46 (17)	C2—C21—H21B	109.5
C11—C1—C12	109.49 (18)	H21A—C21—H21B	109.5
C13—C1—C12	106.89 (17)	C2—C21—H21C	109.5
C11—C1—P1	112.71 (14)	H21A—C21—H21C	109.5
C13—C1—P1	109.01 (14)	H21B—C21—H21C	109.5
C12—C1—P1	110.10 (14)	C2—C22—H22A	109.5
C23—C2—C21	107.45 (17)	C2—C22—H22B	109.5
C23—C2—C22	110.66 (18)	H22A—C22—H22B	109.5
C21—C2—C22	107.42 (18)	C2—C22—H22C	109.5
C23—C2—P1	109.35 (14)	H22A—C22—H22C	109.5
C21—C2—P1	109.63 (14)	H22B—C22—H22C	109.5
C22—C2—P1	112.20 (14)	C2—C23—H23A	109.5
C31—C3—C32	110.28 (17)	C2—C23—H23B	109.5
C31—C3—P1	113.58 (14)	H23A—C23—H23B	109.5
C32—C3—P1	117.06 (14)	C2—C23—H23C	109.5
C31—C3—H3	104.9	H23A—C23—H23C	109.5
C32—C3—H3	104.9	H23B—C23—H23C	109.5
P1—C3—H3	104.9	C3—C31—H31A	109.5
C1—C11—H11A	109.5	C3—C31—H31B	109.5
C1—C11—H11B	109.5	H31A—C31—H31B	109.5
H11A—C11—H11B	109.5	C3—C31—H31C	109.5
C1—C11—H11C	109.5	H31A—C31—H31C	109.5
H11A—C11—H11C	109.5	H31B—C31—H31C	109.5
H11B—C11—H11C	109.5	C3—C32—H32A	109.5
C1—C12—H12A	109.5	C3—C32—H32B	109.5
C1—C12—H12B	109.5	H32A—C32—H32B	109.5
H12A—C12—H12B	109.5	C3—C32—H32C	109.5
C1—C12—H12C	109.5	H32A—C32—H32C	109.5
H12A—C12—H12C	109.5	H32B—C32—H32C	109.5
			
Au1—S1—P1—C3	−50.79 (7)	S1—P1—C2—C23	−70.94 (15)
Au1—S1—P1—C2	−171.73 (7)	C3—P1—C2—C21	−71.15 (16)
Au1—S1—P1—C1	66.65 (7)	C1—P1—C2—C21	165.86 (13)
C3—P1—C1—C11	−82.85 (16)	S1—P1—C2—C21	46.62 (14)
C2—P1—C1—C11	43.29 (17)	C3—P1—C2—C22	48.12 (18)
S1—P1—C1—C11	158.97 (13)	C1—P1—C2—C22	−74.87 (17)
C3—P1—C1—C13	37.63 (15)	S1—P1—C2—C22	165.89 (14)
C2—P1—C1—C13	163.77 (13)	C2—P1—C3—C31	62.01 (17)
S1—P1—C1—C13	−80.56 (13)	C1—P1—C3—C31	−171.81 (14)
C3—P1—C1—C12	154.58 (15)	S1—P1—C3—C31	−52.86 (15)
C2—P1—C1—C12	−79.27 (17)	C2—P1—C3—C32	−68.36 (18)
S1—P1—C1—C12	36.40 (16)	C1—P1—C3—C32	57.83 (17)
C3—P1—C2—C23	171.29 (14)	S1—P1—C3—C32	176.78 (14)
C1—P1—C2—C23	48.30 (17)		

### [Bis(tert-butyl)(propan-2-yl)tripropylphosphane sulfide-κS]bromidogold (3b) Hydrogen-bond geometry (Å, º)

**Table:**

*D*—H···*A*	*D*—H	H···*A*	*D*···*A*	*D*—H···*A*
C13—H13*B*···Au1	0.98	2.75	3.633 (2)	151
C23—H23*A*···S1	0.98	2.99	3.515 (2)	114
C21—H21*C*···S1	0.98	2.70	3.216 (2)	114
C11—H11*C*···Br1^i^	0.98	3.12	4.019 (2)	154
C12—H12*B*···S1^ii^	0.98	2.94	3.603 (2)	126
C12—H12*C*···Br1^i^	0.98	3.12	4.039 (2)	158
C31—H31*C*···Br1^iii^	0.98	3.12	4.073 (2)	165
C23—H23*C*···Br1^iv^	0.98	3.15	4.072 (2)	158

Symmetry codes: (i) −*x*+1/2, −*y*+1/2, −*z*+1; (ii) −*x*+1/2, −*y*+3/2, −*z*+1; (iii) −*x*+1/2, *y*+1/2, −*z*+1/2; (iv) *x*−1/2, −*y*+3/2, *z*−1/2.

### Bromido(tri-tert-butylphosphane sulfide-κS)gold (4b) Crystal data

**Table:**

[AuBr(C_12_H_27_PS)]	*F*(000) = 976
*M**_r_* = 511.24	*D*_x_ = 2.053 Mg m^−^^3^
Monoclinic, *P*2_1_/*c*	Mo *K*α radiation, λ = 0.71073 Å
*a* = 8.3107 (6) Å	Cell parameters from 7046 reflections
*b* = 13.4820 (5) Å	θ = 2.5–30.8°
*c* = 14.7591 (9) Å	µ = 11.51 mm^−^^1^
β = 90.424 (6)°	*T* = 100 K
*V* = 1653.64 (17) Å^3^	Needle, colourless
*Z* = 4	0.15 × 0.04 × 0.04 mm

### Bromido(tri-tert-butylphosphane sulfide-κS)gold (4b) Data collection

**Table:**

Oxford Diffraction Xcalibur, Eos diffractometer	4445 independent reflections
Radiation source: fine-focus sealed tube	3863 reflections with *I* > 2σ(*I*)
Detector resolution: 16.1419 pixels mm^-1^	*R*_int_ = 0.079
ω scan	θ_max_ = 29.1°, θ_min_ = 2.5°
Absorption correction: multi-scan CrysAlisPro, Version 1.171.35.21 (Rigaku OD, 2020)	*h* = −11→11
*T*_min_ = 0.445, *T*_max_ = 1.000	*k* = −18→18
44298 measured reflections	*l* = −20→20

### Bromido(tri-tert-butylphosphane sulfide-κS)gold (4b) Refinement

**Table:**

Refinement on *F*^2^	Primary atom site location: structure-invariant direct methods
Least-squares matrix: full	Secondary atom site location: difference Fourier map
*R*[*F*^2^ > 2σ(*F*^2^)] = 0.037	Hydrogen site location: inferred from neighbouring sites
*wR*(*F*^2^) = 0.084	H-atom parameters constrained
*S* = 1.04	*w* = 1/[σ^2^(*F*_o_^2^) + (0.0357*P*)^2^ + 8.6603*P*] where *P* = (*F*_o_^2^ + 2*F*_c_^2^)/3
4445 reflections	(Δ/σ)_max_ < 0.001
155 parameters	Δρ_max_ = 2.68 e Å^−^^3^
0 restraints	Δρ_min_ = −1.32 e Å^−^^3^

### Bromido(tri-tert-butylphosphane sulfide-κS)gold (4b) Fractional atomic coordinates and isotropic or equivalent isotropic displacement parameters (Å^2^)

**Table:**

	*x*	*y*	*z*	*U*_iso_*/*U*_eq_	
Au1	0.26092 (4)	0.32868 (2)	0.61759 (2)	0.01906 (7)	
Br1	0.16622 (9)	0.23114 (5)	0.49272 (5)	0.02403 (16)	
P1	0.2555 (2)	0.54919 (11)	0.74196 (11)	0.0137 (3)	
S1	0.3601 (2)	0.41208 (13)	0.74016 (14)	0.0245 (4)	
C1	0.3108 (9)	0.5970 (5)	0.8589 (5)	0.0217 (15)	
C2	0.0295 (9)	0.5402 (5)	0.7268 (5)	0.0200 (15)	
C3	0.3463 (8)	0.6302 (5)	0.6502 (5)	0.0177 (13)	
C11	0.2926 (9)	0.7109 (5)	0.8681 (5)	0.0231 (16)	
H11A	0.181089	0.729814	0.854734	0.035*	
H11B	0.364619	0.743914	0.825303	0.035*	
H11C	0.320645	0.730980	0.930022	0.035*	
C12	0.4866 (10)	0.5690 (6)	0.8838 (6)	0.0341 (19)	
H12A	0.511311	0.592143	0.945293	0.051*	
H12B	0.560227	0.600398	0.840895	0.051*	
H12C	0.499294	0.496819	0.880986	0.051*	
C13	0.2073 (11)	0.5475 (6)	0.9308 (5)	0.0309 (19)	
H13A	0.240799	0.570757	0.990957	0.046*	
H13B	0.220513	0.475413	0.927302	0.046*	
H13C	0.094085	0.564670	0.920334	0.046*	
C21	−0.0521 (9)	0.6350 (6)	0.7618 (6)	0.0271 (17)	
H21A	−0.005717	0.692931	0.731479	0.041*	
H21B	−0.034629	0.640614	0.827318	0.041*	
H21C	−0.167773	0.631924	0.748830	0.041*	
C22	−0.0357 (10)	0.4489 (6)	0.7779 (6)	0.0308 (19)	
H22A	−0.152836	0.445651	0.770510	0.046*	
H22B	−0.008469	0.454596	0.842403	0.046*	
H22C	0.012919	0.388527	0.753219	0.046*	
C23	−0.0183 (9)	0.5267 (6)	0.6269 (6)	0.0267 (17)	
H23A	−0.134832	0.516853	0.622109	0.040*	
H23B	0.037342	0.468791	0.602068	0.040*	
H23C	0.012231	0.586032	0.592612	0.040*	
C31	0.2618 (10)	0.7307 (5)	0.6391 (4)	0.0232 (15)	
H31A	0.248637	0.761605	0.698667	0.035*	
H31B	0.155899	0.720763	0.610882	0.035*	
H31C	0.326927	0.773962	0.600574	0.035*	
C32	0.5246 (10)	0.6472 (6)	0.6746 (6)	0.0304 (19)	
H32A	0.576746	0.684108	0.625778	0.046*	
H32B	0.578239	0.583015	0.682606	0.046*	
H32C	0.532253	0.685230	0.731083	0.046*	
C33	0.3433 (11)	0.5764 (6)	0.5579 (5)	0.0287 (17)	
H33A	0.231596	0.567315	0.537935	0.043*	
H33B	0.395457	0.511552	0.564101	0.043*	
H33C	0.401088	0.616248	0.513129	0.043*	

### Bromido(tri-tert-butylphosphane sulfide-κS)gold (4b) Atomic displacement parameters (Å^2^)

**Table:**

	*U*^11^	*U*^22^	*U*^33^	*U*^12^	*U*^13^	*U*^23^
Au1	0.02512 (12)	0.00978 (10)	0.02229 (13)	0.00194 (11)	0.00216 (12)	−0.00021 (10)
Br1	0.0340 (4)	0.0159 (3)	0.0221 (4)	0.0005 (3)	0.0007 (3)	−0.0011 (3)
P1	0.0172 (8)	0.0095 (6)	0.0143 (7)	−0.0003 (7)	−0.0005 (7)	−0.0003 (5)
S1	0.0300 (10)	0.0137 (8)	0.0296 (10)	0.0064 (7)	−0.0078 (8)	−0.0020 (7)
C1	0.035 (4)	0.016 (3)	0.014 (3)	0.000 (3)	−0.006 (3)	−0.001 (3)
C2	0.020 (3)	0.017 (3)	0.024 (4)	0.000 (3)	0.001 (3)	−0.002 (3)
C3	0.018 (3)	0.018 (3)	0.017 (3)	0.004 (3)	0.001 (3)	−0.003 (3)
C11	0.034 (4)	0.015 (3)	0.020 (4)	−0.006 (3)	−0.005 (3)	−0.003 (3)
C12	0.041 (5)	0.034 (4)	0.027 (4)	−0.009 (4)	−0.020 (4)	0.007 (4)
C13	0.048 (5)	0.027 (4)	0.018 (4)	−0.006 (4)	0.001 (3)	0.001 (3)
C21	0.018 (4)	0.031 (4)	0.032 (5)	0.000 (3)	0.000 (3)	−0.013 (3)
C22	0.027 (4)	0.020 (4)	0.045 (5)	−0.010 (3)	0.003 (4)	−0.001 (4)
C23	0.024 (4)	0.023 (4)	0.033 (5)	0.003 (3)	−0.008 (3)	−0.011 (3)
C31	0.037 (4)	0.019 (3)	0.013 (3)	0.003 (3)	−0.001 (4)	0.005 (2)
C32	0.023 (4)	0.023 (4)	0.045 (5)	−0.003 (3)	0.007 (4)	0.000 (4)
C33	0.043 (5)	0.023 (4)	0.020 (4)	0.004 (3)	0.010 (4)	−0.002 (3)

### Bromido(tri-tert-butylphosphane sulfide-κS)gold (4b) Geometric parameters (Å, º)

**Table:**

Au1—S1	2.2791 (19)	C13—H13A	0.9800
Au1—Br1	2.3925 (8)	C13—H13B	0.9800
P1—C2	1.894 (7)	C13—H13C	0.9800
P1—C1	1.895 (7)	C21—H21A	0.9800
P1—C3	1.900 (7)	C21—H21B	0.9800
P1—S1	2.043 (2)	C21—H21C	0.9800
C1—C13	1.525 (11)	C22—H22A	0.9800
C1—C11	1.549 (9)	C22—H22B	0.9800
C1—C12	1.550 (11)	C22—H22C	0.9800
C2—C23	1.535 (11)	C23—H23A	0.9800
C2—C21	1.539 (10)	C23—H23B	0.9800
C2—C22	1.544 (10)	C23—H23C	0.9800
C3—C31	1.534 (9)	C31—H31A	0.9800
C3—C32	1.540 (11)	C31—H31B	0.9800
C3—C33	1.543 (10)	C31—H31C	0.9800
C11—H11A	0.9800	C32—H32A	0.9800
C11—H11B	0.9800	C32—H32B	0.9800
C11—H11C	0.9800	C32—H32C	0.9800
C12—H12A	0.9800	C33—H33A	0.9800
C12—H12B	0.9800	C33—H33B	0.9800
C12—H12C	0.9800	C33—H33C	0.9800
			
S1—Au1—Br1	176.04 (5)	H13A—C13—H13B	109.5
C2—P1—C1	111.3 (3)	C1—C13—H13C	109.5
C2—P1—C3	110.5 (3)	H13A—C13—H13C	109.5
C1—P1—C3	111.0 (3)	H13B—C13—H13C	109.5
C2—P1—S1	111.2 (2)	C2—C21—H21A	109.5
C1—P1—S1	102.7 (2)	C2—C21—H21B	109.5
C3—P1—S1	109.9 (2)	H21A—C21—H21B	109.5
P1—S1—Au1	107.77 (9)	C2—C21—H21C	109.5
C13—C1—C11	108.4 (6)	H21A—C21—H21C	109.5
C13—C1—C12	105.3 (6)	H21B—C21—H21C	109.5
C11—C1—C12	108.2 (6)	C2—C22—H22A	109.5
C13—C1—P1	110.5 (5)	C2—C22—H22B	109.5
C11—C1—P1	113.2 (5)	H22A—C22—H22B	109.5
C12—C1—P1	110.8 (5)	C2—C22—H22C	109.5
C23—C2—C21	108.0 (6)	H22A—C22—H22C	109.5
C23—C2—C22	106.6 (6)	H22B—C22—H22C	109.5
C21—C2—C22	109.9 (6)	C2—C23—H23A	109.5
C23—C2—P1	111.8 (5)	C2—C23—H23B	109.5
C21—C2—P1	110.2 (5)	H23A—C23—H23B	109.5
C22—C2—P1	110.2 (5)	C2—C23—H23C	109.5
C31—C3—C32	109.4 (6)	H23A—C23—H23C	109.5
C31—C3—C33	108.4 (6)	H23B—C23—H23C	109.5
C32—C3—C33	106.6 (6)	C3—C31—H31A	109.5
C31—C3—P1	113.6 (5)	C3—C31—H31B	109.5
C32—C3—P1	107.7 (5)	H31A—C31—H31B	109.5
C33—C3—P1	110.8 (5)	C3—C31—H31C	109.5
C1—C11—H11A	109.5	H31A—C31—H31C	109.5
C1—C11—H11B	109.5	H31B—C31—H31C	109.5
H11A—C11—H11B	109.5	C3—C32—H32A	109.5
C1—C11—H11C	109.5	C3—C32—H32B	109.5
H11A—C11—H11C	109.5	H32A—C32—H32B	109.5
H11B—C11—H11C	109.5	C3—C32—H32C	109.5
C1—C12—H12A	109.5	H32A—C32—H32C	109.5
C1—C12—H12B	109.5	H32B—C32—H32C	109.5
H12A—C12—H12B	109.5	C3—C33—H33A	109.5
C1—C12—H12C	109.5	C3—C33—H33B	109.5
H12A—C12—H12C	109.5	H33A—C33—H33B	109.5
H12B—C12—H12C	109.5	C3—C33—H33C	109.5
C1—C13—H13A	109.5	H33A—C33—H33C	109.5
C1—C13—H13B	109.5	H33B—C33—H33C	109.5
			
C2—P1—S1—Au1	48.4 (3)	C1—P1—C2—C21	45.8 (6)
C1—P1—S1—Au1	167.6 (3)	C3—P1—C2—C21	−78.1 (6)
C3—P1—S1—Au1	−74.3 (3)	S1—P1—C2—C21	159.6 (5)
C2—P1—C1—C13	43.1 (6)	C1—P1—C2—C22	−75.7 (6)
C3—P1—C1—C13	166.7 (5)	C3—P1—C2—C22	160.4 (5)
S1—P1—C1—C13	−76.0 (6)	S1—P1—C2—C22	38.1 (6)
C2—P1—C1—C11	−78.8 (6)	C2—P1—C3—C31	50.2 (6)
C3—P1—C1—C11	44.8 (6)	C1—P1—C3—C31	−73.8 (6)
S1—P1—C1—C11	162.1 (5)	S1—P1—C3—C31	173.4 (4)
C2—P1—C1—C12	159.4 (5)	C2—P1—C3—C32	171.6 (5)
C3—P1—C1—C12	−77.0 (6)	C1—P1—C3—C32	47.6 (6)
S1—P1—C1—C12	40.3 (5)	S1—P1—C3—C32	−65.3 (5)
C1—P1—C2—C23	165.9 (5)	C2—P1—C3—C33	−72.1 (6)
C3—P1—C2—C23	42.1 (6)	C1—P1—C3—C33	163.8 (5)
S1—P1—C2—C23	−80.3 (5)	S1—P1—C3—C33	51.0 (5)

### Bromido(tri-tert-butylphosphane sulfide-κS)gold (4b) Hydrogen-bond geometry (Å, º)

**Table:**

*D*—H···*A*	*D*—H	H···*A*	*D*···*A*	*D*—H···*A*
C13—H13*B*···Br1^i^	0.98	2.98	3.882 (8)	153
C32—H32*A*···Br1^ii^	0.98	3.00	3.936 (8)	161
C33—H33*B*···Au1	0.98	2.82	3.523 (8)	129
C23—H23*B*···Au1	0.98	2.66	3.541 (7)	150
C12—H12*C*···S1	0.98	2.63	3.169 (9)	115
C33—H33*B*···S1	0.98	2.94	3.487 (8)	116
C22—H22*C*···S1	0.98	2.91	3.377 (9)	110

Symmetry codes: (i) *x*, −*y*+1/2, *z*+1/2; (ii) −*x*+1, −*y*+1, −*z*+1.

### Bromido(tripropylphosphane selenide-κSe)gold (5b) Crystal data

**Table:**

[AuBr(C_9_H_21_PSe)]	*F*(000) = 952
*M**_r_* = 516.06	*D*_x_ = 2.438 Mg m^−^^3^*D*_m_ = 2.438 Mg m^−^^3^*D*_m_ measured by ?
Monoclinic, *P*2_1_/*n*	Mo *K*α radiation, λ = 0.71073 Å
*a* = 8.22500 (14) Å	Cell parameters from 21807 reflections
*b* = 11.31793 (17) Å	θ = 2.2–30.8°
*c* = 15.2065 (3) Å	µ = 15.97 mm^−^^1^
β = 96.6895 (16)°	*T* = 100 K
*V* = 1405.93 (4) Å^3^	Block, colourless
*Z* = 4	0.16 × 0.15 × 0.15 mm

### Bromido(tripropylphosphane selenide-κSe)gold (5b) Data collection

**Table:**

Oxford Diffraction Xcalibur, Eos diffractometer	4276 independent reflections
Radiation source: Enhance (Mo) X-ray Source	3812 reflections with *I* > 2σ(*I*)
Detector resolution: 16.1419 pixels mm^-1^	*R*_int_ = 0.029
ω scans	θ_max_ = 30.8°, θ_min_ = 2.3°
Absorption correction: multi-scan CrysAlisPro, Version 1.171.35.21 (Rigaku OD, 2020)	*h* = −11→11
*T*_min_ = 0.529, *T*_max_ = 1.000	*k* = −16→16
63480 measured reflections	*l* = −21→21

### Bromido(tripropylphosphane selenide-κSe)gold (5b) Refinement

**Table:**

Refinement on *F*^2^	Hydrogen site location: inferred from neighbouring sites
Least-squares matrix: full	H-atom parameters constrained
*R*[*F*^2^ > 2σ(*F*^2^)] = 0.015	*w* = 1/[σ^2^(*F*_o_^2^) + (0.0087*P*)^2^ + 1.4421*P*] where *P* = (*F*_o_^2^ + 2*F*_c_^2^)/3
*wR*(*F*^2^) = 0.029	(Δ/σ)_max_ = 0.003
*S* = 1.07	Δρ_max_ = 1.09 e Å^−^^3^
4276 reflections	Δρ_min_ = −0.94 e Å^−^^3^
125 parameters	Extinction correction: SHELXL-2019/3 (Sheldrick, 2015), Fc^*^=kFc[1+0.001xFc^2^λ^3^/sin(2θ)]^-1/4^
0 restraints	Extinction coefficient: 0.00114 (3)
Primary atom site location: structure-invariant direct methods	

### Bromido(tripropylphosphane selenide-κSe)gold (5b) Fractional atomic coordinates and isotropic or equivalent isotropic displacement parameters (Å^2^)

**Table:**

	*x*	*y*	*z*	*U*_iso_*/*U*_eq_	
Au1	0.49755 (2)	0.50261 (2)	0.30090 (2)	0.01658 (3)	
Br1	0.29020 (3)	0.47321 (2)	0.17815 (2)	0.02114 (5)	
P1	0.78688 (6)	0.69268 (4)	0.42405 (3)	0.01254 (9)	
Se1	0.70069 (3)	0.51007 (2)	0.42556 (2)	0.02173 (5)	
C1	0.9266 (2)	0.70575 (17)	0.52661 (13)	0.0172 (4)	
H1	1.004761	0.638194	0.525809	0.021*	
C2	0.6147 (2)	0.79671 (17)	0.41269 (14)	0.0178 (4)	
H2	0.550050	0.779283	0.354305	0.021*	
C3	0.9087 (2)	0.72149 (17)	0.33310 (13)	0.0169 (4)	
H3	0.951786	0.803891	0.341046	0.020*	
C11	1.0329 (2)	0.81786 (19)	0.53110 (14)	0.0217 (4)	
H11A	0.965870	0.886503	0.542582	0.032*	
H11B	1.077585	0.828657	0.474653	0.032*	
H11C	1.122950	0.809847	0.578930	0.032*	
C12	0.8445 (3)	0.6900 (2)	0.61111 (14)	0.0240 (4)	
H12A	0.928060	0.673823	0.661009	0.036*	
H12B	0.767477	0.623664	0.603563	0.036*	
H12C	0.785235	0.762340	0.622887	0.036*	
C21	0.4976 (3)	0.7796 (2)	0.48295 (16)	0.0309 (5)	
H21A	0.550235	0.807220	0.540416	0.046*	
H21B	0.470382	0.695645	0.487019	0.046*	
H21C	0.397278	0.825048	0.466376	0.046*	
C22	0.6705 (3)	0.92562 (18)	0.40853 (17)	0.0275 (5)	
H22A	0.575537	0.976111	0.390803	0.041*	
H22B	0.749242	0.932995	0.365170	0.041*	
H22C	0.722451	0.950168	0.466947	0.041*	
C31	1.0568 (3)	0.6395 (2)	0.33646 (17)	0.0302 (5)	
H31A	1.019434	0.557650	0.327805	0.045*	
H31B	1.122676	0.646920	0.394184	0.045*	
H31C	1.123321	0.661306	0.289531	0.045*	
C32	0.8064 (3)	0.7166 (2)	0.24266 (14)	0.0256 (5)	
H32A	0.876827	0.731850	0.196173	0.038*	
H32B	0.720164	0.776735	0.239936	0.038*	
H32C	0.756560	0.638254	0.233856	0.038*	

### Bromido(tripropylphosphane selenide-κSe)gold (5b) Atomic displacement parameters (Å^2^)

**Table:**

	*U*^11^	*U*^22^	*U*^33^	*U*^12^	*U*^13^	*U*^23^
Au1	0.01623 (4)	0.01300 (4)	0.02085 (4)	−0.00145 (3)	0.00355 (3)	−0.00179 (3)
Br1	0.02214 (9)	0.02341 (10)	0.01810 (9)	0.00032 (8)	0.00328 (7)	−0.00458 (7)
P1	0.0119 (2)	0.0115 (2)	0.0139 (2)	0.00059 (16)	0.00022 (17)	0.00121 (17)
Se1	0.02161 (10)	0.01164 (9)	0.03017 (11)	−0.00203 (7)	−0.00448 (8)	0.00471 (8)
C1	0.0162 (9)	0.0196 (9)	0.0154 (9)	0.0008 (7)	−0.0003 (7)	0.0031 (7)
C2	0.0138 (9)	0.0142 (9)	0.0254 (10)	0.0023 (7)	0.0017 (7)	0.0040 (7)
C3	0.0189 (9)	0.0166 (9)	0.0154 (9)	−0.0001 (7)	0.0029 (7)	−0.0007 (7)
C11	0.0203 (10)	0.0284 (11)	0.0158 (9)	−0.0084 (8)	0.0001 (8)	0.0007 (8)
C12	0.0297 (11)	0.0260 (11)	0.0164 (10)	−0.0058 (9)	0.0032 (8)	0.0048 (8)
C21	0.0224 (11)	0.0313 (12)	0.0415 (14)	0.0103 (9)	0.0150 (10)	0.0107 (10)
C22	0.0199 (10)	0.0142 (9)	0.0483 (14)	0.0029 (8)	0.0042 (10)	0.0036 (9)
C31	0.0292 (12)	0.0314 (12)	0.0327 (12)	0.0097 (9)	0.0145 (10)	−0.0016 (10)
C32	0.0346 (12)	0.0264 (11)	0.0156 (10)	−0.0093 (9)	0.0021 (9)	−0.0007 (8)

### Bromido(tripropylphosphane selenide-κSe)gold (5b) Geometric parameters (Å, º)

**Table:**

Au1—Se1	2.3779 (2)	C11—H11C	0.9800
Au1—Br1	2.4004 (2)	C12—H12A	0.9800
P1—C3	1.829 (2)	C12—H12B	0.9800
P1—C1	1.832 (2)	C12—H12C	0.9800
P1—C2	1.8343 (19)	C21—H21A	0.9800
P1—Se1	2.1860 (5)	C21—H21B	0.9800
C1—C12	1.530 (3)	C21—H21C	0.9800
C1—C11	1.538 (3)	C22—H22A	0.9800
C1—H1	1.0000	C22—H22B	0.9800
C2—C21	1.532 (3)	C22—H22C	0.9800
C2—C22	1.533 (3)	C31—H31A	0.9800
C2—H2	1.0000	C31—H31B	0.9800
C3—C32	1.527 (3)	C31—H31C	0.9800
C3—C31	1.528 (3)	C32—H32A	0.9800
C3—H3	1.0000	C32—H32B	0.9800
C11—H11A	0.9800	C32—H32C	0.9800
C11—H11B	0.9800		
			
Se1—Au1—Br1	173.957 (8)	H11B—C11—H11C	109.5
C3—P1—C1	106.54 (9)	C1—C12—H12A	109.5
C3—P1—C2	107.42 (9)	C1—C12—H12B	109.5
C1—P1—C2	115.41 (9)	H12A—C12—H12B	109.5
C3—P1—Se1	112.54 (7)	C1—C12—H12C	109.5
C1—P1—Se1	103.85 (6)	H12A—C12—H12C	109.5
C2—P1—Se1	111.11 (6)	H12B—C12—H12C	109.5
P1—Se1—Au1	102.931 (14)	C2—C21—H21A	109.5
C12—C1—C11	111.30 (17)	C2—C21—H21B	109.5
C12—C1—P1	114.26 (14)	H21A—C21—H21B	109.5
C11—C1—P1	113.66 (13)	C2—C21—H21C	109.5
C12—C1—H1	105.6	H21A—C21—H21C	109.5
C11—C1—H1	105.6	H21B—C21—H21C	109.5
P1—C1—H1	105.6	C2—C22—H22A	109.5
C21—C2—C22	111.39 (18)	C2—C22—H22B	109.5
C21—C2—P1	113.24 (14)	H22A—C22—H22B	109.5
C22—C2—P1	112.53 (13)	C2—C22—H22C	109.5
C21—C2—H2	106.4	H22A—C22—H22C	109.5
C22—C2—H2	106.4	H22B—C22—H22C	109.5
P1—C2—H2	106.4	C3—C31—H31A	109.5
C32—C3—C31	111.21 (18)	C3—C31—H31B	109.5
C32—C3—P1	112.43 (14)	H31A—C31—H31B	109.5
C31—C3—P1	111.80 (14)	C3—C31—H31C	109.5
C32—C3—H3	107.0	H31A—C31—H31C	109.5
C31—C3—H3	107.0	H31B—C31—H31C	109.5
P1—C3—H3	107.0	C3—C32—H32A	109.5
C1—C11—H11A	109.5	C3—C32—H32B	109.5
C1—C11—H11B	109.5	H32A—C32—H32B	109.5
H11A—C11—H11B	109.5	C3—C32—H32C	109.5
C1—C11—H11C	109.5	H32A—C32—H32C	109.5
H11A—C11—H11C	109.5	H32B—C32—H32C	109.5
			
C3—P1—Se1—Au1	−71.46 (7)	Se1—P1—C2—C21	54.33 (17)
C1—P1—Se1—Au1	173.72 (7)	C3—P1—C2—C22	−54.75 (18)
C2—P1—Se1—Au1	49.05 (7)	C1—P1—C2—C22	63.89 (18)
C3—P1—C1—C12	177.33 (14)	Se1—P1—C2—C22	−178.24 (14)
C2—P1—C1—C12	58.21 (17)	C1—P1—C3—C32	−178.55 (14)
Se1—P1—C1—C12	−63.65 (15)	C2—P1—C3—C32	−54.33 (16)
C3—P1—C1—C11	48.10 (17)	Se1—P1—C3—C32	68.27 (15)
C2—P1—C1—C11	−71.03 (17)	C1—P1—C3—C31	55.53 (17)
Se1—P1—C1—C11	167.12 (13)	C2—P1—C3—C31	179.74 (15)
C3—P1—C2—C21	177.82 (16)	Se1—P1—C3—C31	−57.65 (16)
C1—P1—C2—C21	−63.54 (18)		

### Bromido(tripropylphosphane selenide-κSe)gold (5b) Hydrogen-bond geometry (Å, º)

**Table:**

*D*—H···*A*	*D*—H	H···*A*	*D*···*A*	*D*—H···*A*
C3—H3···Br1^i^	1.00	2.90	3.792 (2)	149
C1—H1···Se1^ii^	1.00	2.97	3.9210 (19)	159
C12—H12*B*···Se1	0.98	2.99	3.568 (2)	119
C21—H21*B*···Se1	0.98	3.05	3.633 (2)	120

Symmetry codes: (i) −*x*+3/2, *y*+1/2, −*z*+1/2; (ii) −*x*+2, −*y*+1, −*z*+1.

### Bromido[(tert-butyl)bis(propan-2-yl)phosphane selenide-κSe]gold (6b) Crystal data

**Table:**

[AuBr(C_10_H_23_PSe)]	*F*(000) = 1968
*M**_r_* = 530.09	*D*_x_ = 2.385 Mg m^−^^3^
Monoclinic, *P*2_1_/*c*	Mo *K*α radiation, λ = 0.71073 Å
*a* = 11.5037 (2) Å	Cell parameters from 36701 reflections
*b* = 15.2440 (2) Å	θ = 2.4–30.6°
*c* = 16.8366 (2) Å	µ = 15.21 mm^−^^1^
β = 90.053 (2)°	*T* = 100 K
*V* = 2952.52 (7) Å^3^	Prism, dichroic colourless/orange
*Z* = 8	0.3 × 0.15 × 0.15 mm

### Bromido[(tert-butyl)bis(propan-2-yl)phosphane selenide-κSe]gold (6b) Data collection

**Table:**

Oxford Diffraction Xcalibur, Eos diffractometer	8952 independent reflections
Radiation source: fine-focus sealed tube	8049 reflections with *I* > 2σ(*I*)
Detector resolution: 16.1419 pixels mm^-1^	*R*_int_ = 0.044
ω–scan	θ_max_ = 30.9°, θ_min_ = 2.2°
Absorption correction: multi-scan CrysAlisPro, Version 1.171.37.35 (Rigaku OD, 2020)	*h* = −16→16
*T*_min_ = 0.427, *T*_max_ = 1.000	*k* = −21→21
117465 measured reflections	*l* = −24→24

### Bromido[(tert-butyl)bis(propan-2-yl)phosphane selenide-κSe]gold (6b) Refinement

**Table:**

Refinement on *F*^2^	Primary atom site location: structure-invariant direct methods
Least-squares matrix: full	Secondary atom site location: difference Fourier map
*R*[*F*^2^ > 2σ(*F*^2^)] = 0.022	Hydrogen site location: inferred from neighbouring sites
*wR*(*F*^2^) = 0.040	H-atom parameters constrained
*S* = 1.09	*w* = 1/[σ^2^(*F*_o_^2^) + (0.012*P*)^2^ + 5.1823*P*] where *P* = (*F*_o_^2^ + 2*F*_c_^2^)/3
8952 reflections	(Δ/σ)_max_ = 0.004
267 parameters	Δρ_max_ = 1.84 e Å^−^^3^
0 restraints	Δρ_min_ = −1.22 e Å^−^^3^

### Bromido[(tert-butyl)bis(propan-2-yl)phosphane selenide-κSe]gold (6b) Fractional atomic coordinates and isotropic or equivalent isotropic displacement parameters (Å^2^)

**Table:**

	*x*	*y*	*z*	*U*_iso_*/*U*_eq_	
Au1	0.02177 (2)	0.28535 (2)	0.62749 (2)	0.01448 (3)	
Br1	0.03797 (3)	0.22537 (2)	0.49594 (2)	0.02124 (6)	
Se1	0.01257 (3)	0.33941 (2)	0.76041 (2)	0.01625 (6)	
P1	0.00117 (6)	0.48111 (4)	0.73996 (4)	0.00900 (12)	
C1	−0.1221 (2)	0.51372 (18)	0.67384 (16)	0.0121 (5)	
C2	−0.0212 (2)	0.53038 (18)	0.83845 (16)	0.0132 (5)	
H2	−0.028659	0.595169	0.830689	0.016*	
C3	0.1436 (2)	0.52303 (18)	0.70749 (16)	0.0135 (5)	
H3	0.199686	0.502779	0.749055	0.016*	
C11	−0.1610 (3)	0.60789 (19)	0.69365 (18)	0.0192 (6)	
H11A	−0.094827	0.647901	0.687899	0.029*	
H11B	−0.189597	0.610078	0.748411	0.029*	
H11C	−0.223183	0.625604	0.657229	0.029*	
C12	−0.2235 (2)	0.4494 (2)	0.68408 (19)	0.0198 (6)	
H12A	−0.287533	0.466448	0.648866	0.030*	
H12B	−0.250217	0.450616	0.739328	0.030*	
H12C	−0.197642	0.389982	0.670584	0.030*	
C13	−0.0837 (3)	0.5116 (2)	0.58690 (17)	0.0204 (6)	
H13A	−0.051074	0.453770	0.574561	0.031*	
H13B	−0.024631	0.556771	0.577927	0.031*	
H13C	−0.150905	0.522737	0.552522	0.031*	
C21	−0.1324 (3)	0.4988 (2)	0.87964 (17)	0.0217 (6)	
H21A	−0.128246	0.435221	0.887805	0.033*	
H21B	−0.199824	0.512747	0.846338	0.033*	
H21C	−0.140210	0.528234	0.931105	0.033*	
C22	0.0831 (3)	0.5149 (2)	0.89326 (17)	0.0207 (6)	
H22A	0.068117	0.542017	0.945045	0.031*	
H22B	0.152734	0.541182	0.869657	0.031*	
H22C	0.095158	0.451766	0.900163	0.031*	
C31	0.1522 (3)	0.62356 (18)	0.70859 (18)	0.0184 (6)	
H31A	0.233543	0.641183	0.702186	0.028*	
H31B	0.122614	0.645787	0.759323	0.028*	
H31C	0.105786	0.647854	0.664978	0.028*	
C32	0.1907 (3)	0.4859 (2)	0.62945 (18)	0.0214 (6)	
H32A	0.156156	0.517804	0.584717	0.032*	
H32B	0.170577	0.423595	0.625493	0.032*	
H32C	0.275365	0.492586	0.628141	0.032*	
Au2	0.49087 (2)	0.42333 (2)	0.35333 (2)	0.01308 (3)	
Br2	0.53893 (3)	0.46757 (2)	0.22030 (2)	0.02186 (6)	
Se2	0.43625 (2)	0.39463 (2)	0.48744 (2)	0.01454 (6)	
P2	0.47987 (6)	0.25612 (4)	0.50370 (4)	0.00837 (12)	
C4	0.6322 (2)	0.22881 (17)	0.47325 (16)	0.0112 (5)	
C5	0.4635 (2)	0.23505 (18)	0.61056 (15)	0.0126 (5)	
H5	0.486436	0.172650	0.620038	0.015*	
C6	0.3685 (2)	0.18711 (17)	0.45599 (16)	0.0119 (5)	
H6	0.293386	0.205944	0.480467	0.014*	
C41	0.6760 (2)	0.1496 (2)	0.52158 (18)	0.0187 (6)	
H41A	0.753378	0.132560	0.502816	0.028*	
H41B	0.622180	0.100268	0.514740	0.028*	
H41C	0.680154	0.165417	0.577913	0.028*	
C42	0.7118 (2)	0.3078 (2)	0.48810 (18)	0.0179 (6)	
H42A	0.792363	0.291604	0.476156	0.027*	
H42B	0.705890	0.325726	0.543846	0.027*	
H42C	0.688047	0.356547	0.453794	0.027*	
C43	0.6357 (2)	0.20605 (19)	0.38448 (17)	0.0166 (6)	
H43A	0.600368	0.253809	0.353854	0.025*	
H43B	0.592291	0.151684	0.375167	0.025*	
H43C	0.716563	0.198132	0.367729	0.025*	
C51	0.5426 (3)	0.2924 (2)	0.66223 (17)	0.0229 (6)	
H51A	0.526539	0.354427	0.651489	0.034*	
H51B	0.624119	0.279628	0.649822	0.034*	
H51C	0.527911	0.279888	0.718416	0.034*	
C52	0.3367 (3)	0.2447 (2)	0.63743 (18)	0.0218 (6)	
H52A	0.331457	0.233764	0.694676	0.033*	
H52B	0.288191	0.202223	0.608944	0.033*	
H52C	0.309532	0.304270	0.625850	0.033*	
C61	0.3797 (3)	0.08939 (18)	0.47667 (17)	0.0175 (6)	
H61A	0.310551	0.057986	0.457986	0.026*	
H61B	0.386740	0.082649	0.534363	0.026*	
H61C	0.448941	0.065143	0.450873	0.026*	
C62	0.3514 (2)	0.20028 (19)	0.36642 (16)	0.0159 (5)	
H62A	0.406334	0.163125	0.337336	0.024*	
H62B	0.365206	0.261926	0.352887	0.024*	
H62C	0.271723	0.184190	0.351801	0.024*	

### Bromido[(tert-butyl)bis(propan-2-yl)phosphane selenide-κSe]gold (6b) Atomic displacement parameters (Å^2^)

**Table:**

	*U*^11^	*U*^22^	*U*^33^	*U*^12^	*U*^13^	*U*^23^
Au1	0.01496 (5)	0.00911 (5)	0.01935 (6)	0.00057 (4)	−0.00121 (4)	−0.00208 (4)
Br1	0.01947 (14)	0.02124 (15)	0.02301 (15)	0.00216 (11)	0.00056 (11)	−0.00789 (12)
Se1	0.02462 (14)	0.00794 (12)	0.01617 (14)	−0.00090 (10)	−0.00295 (11)	0.00250 (10)
P1	0.0112 (3)	0.0081 (3)	0.0078 (3)	−0.0005 (2)	−0.0001 (2)	0.0001 (2)
C1	0.0138 (12)	0.0118 (12)	0.0107 (12)	0.0027 (10)	−0.0045 (10)	−0.0006 (10)
C2	0.0154 (13)	0.0138 (13)	0.0103 (12)	−0.0034 (10)	0.0007 (10)	−0.0020 (10)
C3	0.0134 (12)	0.0130 (13)	0.0141 (13)	−0.0018 (10)	0.0011 (10)	−0.0005 (10)
C11	0.0185 (14)	0.0174 (14)	0.0216 (15)	0.0058 (11)	−0.0053 (11)	−0.0037 (12)
C12	0.0142 (13)	0.0214 (15)	0.0239 (16)	−0.0007 (11)	−0.0059 (11)	−0.0046 (12)
C13	0.0296 (16)	0.0200 (15)	0.0117 (13)	0.0078 (12)	−0.0031 (12)	−0.0010 (11)
C21	0.0237 (15)	0.0288 (17)	0.0128 (14)	−0.0064 (12)	0.0043 (11)	−0.0029 (12)
C22	0.0263 (15)	0.0238 (16)	0.0119 (13)	−0.0049 (12)	−0.0055 (11)	0.0002 (12)
C31	0.0204 (14)	0.0122 (13)	0.0227 (15)	−0.0048 (11)	0.0071 (11)	−0.0037 (11)
C32	0.0200 (14)	0.0199 (15)	0.0245 (16)	−0.0029 (11)	0.0094 (12)	−0.0051 (12)
Au2	0.01609 (5)	0.00896 (5)	0.01419 (5)	0.00013 (4)	−0.00107 (4)	0.00259 (4)
Br2	0.03473 (17)	0.01653 (14)	0.01433 (14)	−0.00345 (12)	0.00149 (12)	0.00132 (11)
Se2	0.02155 (14)	0.00709 (12)	0.01500 (13)	0.00347 (10)	0.00236 (10)	0.00092 (10)
P2	0.0101 (3)	0.0067 (3)	0.0083 (3)	0.0007 (2)	−0.0001 (2)	0.0007 (2)
C4	0.0099 (11)	0.0123 (12)	0.0115 (12)	0.0016 (9)	0.0008 (9)	0.0011 (10)
C5	0.0138 (12)	0.0138 (13)	0.0101 (12)	−0.0005 (10)	0.0008 (10)	0.0025 (10)
C6	0.0099 (11)	0.0106 (12)	0.0153 (13)	−0.0028 (9)	−0.0005 (9)	−0.0002 (10)
C41	0.0152 (13)	0.0193 (15)	0.0217 (15)	0.0065 (11)	0.0020 (11)	0.0071 (12)
C42	0.0123 (13)	0.0211 (15)	0.0202 (15)	−0.0031 (11)	0.0001 (11)	0.0008 (12)
C43	0.0155 (13)	0.0173 (14)	0.0168 (14)	0.0015 (10)	0.0042 (10)	−0.0017 (11)
C51	0.0297 (16)	0.0277 (17)	0.0114 (14)	−0.0067 (13)	−0.0009 (12)	−0.0011 (12)
C52	0.0220 (15)	0.0272 (17)	0.0161 (15)	0.0011 (12)	0.0074 (12)	0.0026 (12)
C61	0.0243 (15)	0.0105 (13)	0.0178 (14)	−0.0057 (11)	−0.0023 (11)	0.0014 (11)
C62	0.0172 (13)	0.0167 (14)	0.0139 (13)	−0.0006 (10)	−0.0055 (10)	0.0004 (11)

### Bromido[(tert-butyl)bis(propan-2-yl)phosphane selenide-κSe]gold (6b) Geometric parameters (Å, º)

**Table:**

Au1—Se1	2.3872 (3)	Au2—Se2	2.3848 (3)
Au1—Br1	2.4036 (3)	Au2—Br2	2.4040 (3)
Se1—P1	2.1911 (7)	Se2—P2	2.1875 (7)
P1—C2	1.839 (3)	P2—C5	1.837 (3)
P1—C3	1.842 (3)	P2—C6	1.841 (3)
P1—C1	1.869 (3)	P2—C4	1.874 (3)
C1—C13	1.530 (4)	C4—C42	1.533 (4)
C1—C12	1.534 (4)	C4—C43	1.535 (4)
C1—C11	1.540 (4)	C4—C41	1.541 (4)
C2—C22	1.531 (4)	C5—C51	1.533 (4)
C2—C21	1.533 (4)	C5—C52	1.534 (4)
C2—H2	1.0000	C5—H5	1.0000
C3—C32	1.530 (4)	C6—C62	1.534 (4)
C3—C31	1.536 (4)	C6—C61	1.535 (4)
C3—H3	1.0000	C6—H6	1.0000
C11—H11A	0.9800	C41—H41A	0.9800
C11—H11B	0.9800	C41—H41B	0.9800
C11—H11C	0.9800	C41—H41C	0.9800
C12—H12A	0.9800	C42—H42A	0.9800
C12—H12B	0.9800	C42—H42B	0.9800
C12—H12C	0.9800	C42—H42C	0.9800
C13—H13A	0.9800	C43—H43A	0.9800
C13—H13B	0.9800	C43—H43B	0.9800
C13—H13C	0.9800	C43—H43C	0.9800
C21—H21A	0.9800	C51—H51A	0.9800
C21—H21B	0.9800	C51—H51B	0.9800
C21—H21C	0.9800	C51—H51C	0.9800
C22—H22A	0.9800	C52—H52A	0.9800
C22—H22B	0.9800	C52—H52B	0.9800
C22—H22C	0.9800	C52—H52C	0.9800
C31—H31A	0.9800	C61—H61A	0.9800
C31—H31B	0.9800	C61—H61B	0.9800
C31—H31C	0.9800	C61—H61C	0.9800
C32—H32A	0.9800	C62—H62A	0.9800
C32—H32B	0.9800	C62—H62B	0.9800
C32—H32C	0.9800	C62—H62C	0.9800
			
Se1—Au1—Br1	177.083 (11)	Se2—Au2—Br2	174.050 (11)
P1—Se1—Au1	101.29 (2)	P2—Se2—Au2	103.59 (2)
C2—P1—C3	104.54 (12)	C5—P2—C6	104.78 (12)
C2—P1—C1	108.77 (12)	C5—P2—C4	109.03 (12)
C3—P1—C1	113.93 (13)	C6—P2—C4	113.85 (12)
C2—P1—Se1	105.63 (9)	C5—P2—Se2	105.52 (9)
C3—P1—Se1	109.60 (9)	C6—P2—Se2	109.72 (9)
C1—P1—Se1	113.64 (9)	C4—P2—Se2	113.25 (9)
C13—C1—C12	108.3 (2)	C42—C4—C43	108.7 (2)
C13—C1—C11	108.1 (2)	C42—C4—C41	109.6 (2)
C12—C1—C11	110.5 (2)	C43—C4—C41	109.2 (2)
C13—C1—P1	110.16 (19)	C42—C4—P2	109.85 (18)
C12—C1—P1	109.86 (19)	C43—C4—P2	109.97 (18)
C11—C1—P1	109.82 (18)	C41—C4—P2	109.56 (18)
C22—C2—C21	109.4 (2)	C51—C5—C52	110.0 (2)
C22—C2—P1	111.75 (19)	C51—C5—P2	113.23 (19)
C21—C2—P1	113.40 (19)	C52—C5—P2	111.75 (19)
C22—C2—H2	107.3	C51—C5—H5	107.2
C21—C2—H2	107.3	C52—C5—H5	107.2
P1—C2—H2	107.3	P2—C5—H5	107.2
C32—C3—C31	110.9 (2)	C62—C6—C61	111.1 (2)
C32—C3—P1	116.2 (2)	C62—C6—P2	116.28 (19)
C31—C3—P1	113.55 (19)	C61—C6—P2	113.41 (19)
C32—C3—H3	105.0	C62—C6—H6	104.9
C31—C3—H3	105.0	C61—C6—H6	104.9
P1—C3—H3	105.0	P2—C6—H6	104.9
C1—C11—H11A	109.5	C4—C41—H41A	109.5
C1—C11—H11B	109.5	C4—C41—H41B	109.5
H11A—C11—H11B	109.5	H41A—C41—H41B	109.5
C1—C11—H11C	109.5	C4—C41—H41C	109.5
H11A—C11—H11C	109.5	H41A—C41—H41C	109.5
H11B—C11—H11C	109.5	H41B—C41—H41C	109.5
C1—C12—H12A	109.5	C4—C42—H42A	109.5
C1—C12—H12B	109.5	C4—C42—H42B	109.5
H12A—C12—H12B	109.5	H42A—C42—H42B	109.5
C1—C12—H12C	109.5	C4—C42—H42C	109.5
H12A—C12—H12C	109.5	H42A—C42—H42C	109.5
H12B—C12—H12C	109.5	H42B—C42—H42C	109.5
C1—C13—H13A	109.5	C4—C43—H43A	109.5
C1—C13—H13B	109.5	C4—C43—H43B	109.5
H13A—C13—H13B	109.5	H43A—C43—H43B	109.5
C1—C13—H13C	109.5	C4—C43—H43C	109.5
H13A—C13—H13C	109.5	H43A—C43—H43C	109.5
H13B—C13—H13C	109.5	H43B—C43—H43C	109.5
C2—C21—H21A	109.5	C5—C51—H51A	109.5
C2—C21—H21B	109.5	C5—C51—H51B	109.5
H21A—C21—H21B	109.5	H51A—C51—H51B	109.5
C2—C21—H21C	109.5	C5—C51—H51C	109.5
H21A—C21—H21C	109.5	H51A—C51—H51C	109.5
H21B—C21—H21C	109.5	H51B—C51—H51C	109.5
C2—C22—H22A	109.5	C5—C52—H52A	109.5
C2—C22—H22B	109.5	C5—C52—H52B	109.5
H22A—C22—H22B	109.5	H52A—C52—H52B	109.5
C2—C22—H22C	109.5	C5—C52—H52C	109.5
H22A—C22—H22C	109.5	H52A—C52—H52C	109.5
H22B—C22—H22C	109.5	H52B—C52—H52C	109.5
C3—C31—H31A	109.5	C6—C61—H61A	109.5
C3—C31—H31B	109.5	C6—C61—H61B	109.5
H31A—C31—H31B	109.5	H61A—C61—H61B	109.5
C3—C31—H31C	109.5	C6—C61—H61C	109.5
H31A—C31—H31C	109.5	H61A—C61—H61C	109.5
H31B—C31—H31C	109.5	H61B—C61—H61C	109.5
C3—C32—H32A	109.5	C6—C62—H62A	109.5
C3—C32—H32B	109.5	C6—C62—H62B	109.5
H32A—C32—H32B	109.5	H62A—C62—H62B	109.5
C3—C32—H32C	109.5	C6—C62—H62C	109.5
H32A—C32—H32C	109.5	H62A—C62—H62C	109.5
H32B—C32—H32C	109.5	H62B—C62—H62C	109.5
			
Au1—Se1—P1—C2	174.51 (9)	Au2—Se2—P2—C5	170.47 (9)
Au1—Se1—P1—C3	−73.38 (10)	Au2—Se2—P2—C6	−77.14 (9)
Au1—Se1—P1—C1	55.36 (9)	Au2—Se2—P2—C4	51.29 (9)
C2—P1—C1—C13	154.4 (2)	C5—P2—C4—C42	−85.4 (2)
C3—P1—C1—C13	38.3 (2)	C6—P2—C4—C42	158.01 (18)
Se1—P1—C1—C13	−88.2 (2)	Se2—P2—C4—C42	31.7 (2)
C2—P1—C1—C12	−86.4 (2)	C5—P2—C4—C43	155.01 (19)
C3—P1—C1—C12	157.48 (19)	C6—P2—C4—C43	38.4 (2)
Se1—P1—C1—C12	31.0 (2)	Se2—P2—C4—C43	−87.85 (19)
C2—P1—C1—C11	35.4 (2)	C5—P2—C4—C41	35.0 (2)
C3—P1—C1—C11	−80.7 (2)	C6—P2—C4—C41	−81.6 (2)
Se1—P1—C1—C11	152.76 (17)	Se2—P2—C4—C41	152.16 (17)
C3—P1—C2—C22	−51.7 (2)	C6—P2—C5—C51	−175.3 (2)
C1—P1—C2—C22	−173.72 (19)	C4—P2—C5—C51	62.4 (2)
Se1—P1—C2—C22	64.0 (2)	Se2—P2—C5—C51	−59.5 (2)
C3—P1—C2—C21	−175.9 (2)	C6—P2—C5—C52	−50.4 (2)
C1—P1—C2—C21	62.0 (2)	C4—P2—C5—C52	−172.7 (2)
Se1—P1—C2—C21	−60.3 (2)	Se2—P2—C5—C52	65.4 (2)
C2—P1—C3—C32	174.6 (2)	C5—P2—C6—C62	172.4 (2)
C1—P1—C3—C32	−66.8 (2)	C4—P2—C6—C62	−68.6 (2)
Se1—P1—C3—C32	61.8 (2)	Se2—P2—C6—C62	59.5 (2)
C2—P1—C3—C31	−54.9 (2)	C5—P2—C6—C61	−56.9 (2)
C1—P1—C3—C31	63.7 (2)	C4—P2—C6—C61	62.1 (2)
Se1—P1—C3—C31	−167.75 (18)	Se2—P2—C6—C61	−169.77 (17)

### Bromido[(tert-butyl)bis(propan-2-yl)phosphane selenide-κSe]gold (6b) Hydrogen-bond geometry (Å, º)

**Table:**

*D*—H···*A*	*D*—H	H···*A*	*D*···*A*	*D*—H···*A*
C13—H13*A*···Au1	0.98	2.84	3.719 (3)	149
C32—H32*B*···Au1	0.98	2.72	3.623 (3)	154
C43—H43*A*···Au2	0.98	2.87	3.744 (3)	148
C62—H62*B*···Au2	0.98	2.85	3.766 (3)	155
C5—H5···Br2^i^	1.00	2.79	3.702 (3)	152
C6—H6···Br1	1.00	2.96	3.906 (3)	157
C3—H3···Br2^ii^	1.00	3.08	3.850 (3)	134
C42—H42*A*···Br1^iii^	0.98	3.02	3.959 (3)	161
C2—H2···Au1^iv^	1.00	2.98	3.929 (3)	158

Symmetry codes: (i) *x*, −*y*+1/2, *z*+1/2; (ii) −*x*+1, −*y*+1, −*z*+1; (iii) *x*+1, *y*, *z*; (iv) −*x*, *y*+1/2, −*z*+3/2.

### [Bis(tert-butyl)(propan-2-yl)phosphane selenide-κSe]bromidogold (7b) Crystal data

**Table:**

[AuBr(C_11_H_25_PSe)]	*F*(000) = 1016
*M**_r_* = 544.11	*D*_x_ = 2.288 Mg m^−^^3^
Monoclinic, *P*2_1_/*c*	Mo *K*α radiation, λ = 0.71073 Å
*a* = 7.66804 (8) Å	Cell parameters from 31967 reflections
*b* = 14.77026 (16) Å	θ = 2.7–30.9°
*c* = 13.94963 (15) Å	µ = 14.22 mm^−^^1^
β = 90.4697 (10)°	*T* = 100 K
*V* = 1579.87 (3) Å^3^	Plate, colourless
*Z* = 4	0.4 × 0.35 × 0.25 mm

### [Bis(tert-butyl)(propan-2-yl)phosphane selenide-κSe]bromidogold (7b) Data collection

**Table:**

Oxford Diffraction Xcalibur, Eos diffractometer	4774 independent reflections
Radiation source: fine-focus sealed tube	4380 reflections with *I* > 2σ(*I*)
Detector resolution: 16.1419 pixels mm^-1^	*R*_int_ = 0.072
ω scans	θ_max_ = 30.9°, θ_min_ = 2.7°
Absorption correction: multi-scan CrysAlisPro, Version 1.171.35.11 (Rigaku OD, 2020)	*h* = −11→10
*T*_min_ = 0.070, *T*_max_ = 0.125	*k* = −21→21
86680 measured reflections	*l* = −19→20

### [Bis(tert-butyl)(propan-2-yl)phosphane selenide-κSe]bromidogold (7b) Refinement

**Table:**

Refinement on *F*^2^	Secondary atom site location: difference Fourier map
Least-squares matrix: full	Hydrogen site location: inferred from neighbouring sites
*R*[*F*^2^ > 2σ(*F*^2^)] = 0.033	H-atom parameters constrained
*wR*(*F*^2^) = 0.083	*w* = 1/[σ^2^(*F*_o_^2^) + (0.0399*P*)^2^ + 11.7761*P*] where *P* = (*F*_o_^2^ + 2*F*_c_^2^)/3
*S* = 1.07	(Δ/σ)_max_ = 0.001
4774 reflections	Δρ_max_ = 3.43 e Å^−^^3^
145 parameters	Δρ_min_ = −2.67 e Å^−^^3^
0 restraints	Extinction correction: SHELXL-2019/3 (Sheldrick, 2015), Fc^*^=kFc[1+0.001xFc^2^λ^3^/sin(2θ)]^-1/4^
Primary atom site location: structure-invariant direct methods	Extinction coefficient: 0.00170 (12)

### [Bis(tert-butyl)(propan-2-yl)phosphane selenide-κSe]bromidogold (7b) Fractional atomic coordinates and isotropic or equivalent isotropic displacement parameters (Å^2^)

**Table:**

	*x*	*y*	*z*	*U*_iso_*/*U*_eq_	
Au1	0.12154 (2)	0.61614 (2)	0.08770 (2)	0.01304 (7)	
Br1	0.14892 (6)	0.77340 (3)	0.04695 (3)	0.01770 (10)	
P1	0.26695 (13)	0.42557 (7)	0.23413 (7)	0.00711 (18)	
Se1	0.08490 (6)	0.45875 (3)	0.11646 (3)	0.01459 (10)	
C1	0.2357 (6)	0.5025 (3)	0.3393 (3)	0.0136 (8)	
C2	0.4942 (6)	0.4238 (3)	0.1859 (4)	0.0164 (9)	
C3	0.1932 (6)	0.3128 (3)	0.2746 (3)	0.0154 (8)	
H3	0.097130	0.325296	0.320828	0.018*	
C11	0.3046 (7)	0.4592 (4)	0.4329 (3)	0.0230 (10)	
H11A	0.426159	0.440626	0.424478	0.035*	
H11B	0.233577	0.406181	0.448613	0.035*	
H11C	0.297718	0.503428	0.485176	0.035*	
C12	0.0399 (6)	0.5180 (3)	0.3520 (3)	0.0161 (9)	
H12A	0.020744	0.557239	0.407602	0.024*	
H12B	−0.018259	0.459758	0.362172	0.024*	
H12C	−0.008252	0.546981	0.294382	0.024*	
C13	0.3256 (7)	0.5940 (4)	0.3238 (4)	0.0219 (10)	
H13A	0.290841	0.636186	0.374453	0.033*	
H13B	0.291000	0.618631	0.261247	0.033*	
H13C	0.452390	0.585811	0.325878	0.033*	
C21	0.6313 (7)	0.4103 (5)	0.2660 (5)	0.0351 (15)	
H21A	0.747563	0.406331	0.237691	0.053*	
H21B	0.606056	0.354296	0.300892	0.053*	
H21C	0.627351	0.461717	0.310332	0.053*	
C22	0.5029 (9)	0.3448 (4)	0.1148 (5)	0.0317 (14)	
H22A	0.414418	0.353206	0.064526	0.048*	
H22B	0.481016	0.287862	0.148695	0.048*	
H22C	0.618833	0.342905	0.085868	0.048*	
C23	0.5365 (6)	0.5109 (3)	0.1325 (4)	0.0178 (9)	
H23A	0.531425	0.562147	0.176989	0.027*	
H23B	0.451477	0.519912	0.080555	0.027*	
H23C	0.653900	0.506629	0.105615	0.027*	
C31	0.3266 (8)	0.2557 (4)	0.3313 (5)	0.0313 (13)	
H31A	0.266005	0.206696	0.364621	0.047*	
H31B	0.386743	0.294200	0.378289	0.047*	
H31C	0.411886	0.229995	0.287031	0.047*	
C32	0.1103 (10)	0.2537 (4)	0.1965 (4)	0.0336 (14)	
H32A	0.200364	0.233931	0.151647	0.050*	
H32B	0.021462	0.288769	0.161767	0.050*	
H32C	0.055888	0.200630	0.225905	0.050*	

### [Bis(tert-butyl)(propan-2-yl)phosphane selenide-κSe]bromidogold (7b) Atomic displacement parameters (Å^2^)

**Table:**

	*U*^11^	*U*^22^	*U*^33^	*U*^12^	*U*^13^	*U*^23^
Au1	0.01544 (10)	0.01557 (10)	0.00815 (10)	0.00387 (5)	0.00217 (6)	0.00420 (6)
Br1	0.0288 (2)	0.0135 (2)	0.0109 (2)	0.00579 (17)	0.00564 (16)	0.00103 (15)
P1	0.0098 (4)	0.0051 (4)	0.0064 (4)	−0.0002 (3)	0.0019 (3)	0.0002 (3)
Se1	0.0162 (2)	0.0180 (2)	0.0094 (2)	−0.00575 (16)	−0.00396 (15)	0.00316 (16)
C1	0.017 (2)	0.016 (2)	0.0078 (18)	−0.0067 (16)	0.0044 (14)	−0.0039 (16)
C2	0.0135 (19)	0.017 (2)	0.019 (2)	0.0043 (16)	0.0093 (16)	0.0048 (18)
C3	0.024 (2)	0.0070 (18)	0.015 (2)	−0.0013 (16)	0.0093 (17)	0.0035 (16)
C11	0.025 (2)	0.035 (3)	0.009 (2)	−0.009 (2)	−0.0027 (17)	0.0015 (19)
C12	0.023 (2)	0.0109 (19)	0.015 (2)	0.0016 (16)	0.0096 (17)	−0.0023 (16)
C13	0.032 (3)	0.017 (2)	0.017 (2)	−0.0115 (19)	0.0070 (19)	−0.0048 (18)
C21	0.012 (2)	0.060 (4)	0.033 (3)	0.008 (2)	0.003 (2)	0.025 (3)
C22	0.043 (3)	0.015 (2)	0.037 (3)	0.008 (2)	0.028 (3)	−0.001 (2)
C23	0.016 (2)	0.019 (2)	0.019 (2)	−0.0043 (16)	0.0095 (16)	0.0027 (18)
C31	0.035 (3)	0.018 (2)	0.041 (3)	0.013 (2)	0.015 (3)	0.018 (2)
C32	0.064 (4)	0.012 (2)	0.025 (3)	−0.016 (2)	0.012 (3)	−0.009 (2)

### [Bis(tert-butyl)(propan-2-yl)phosphane selenide-κSe]bromidogold (7b) Geometric parameters (Å, º)

**Table:**

Au1—Se1	2.3761 (5)	C12—H12C	0.9800
Au1—Br1	2.4009 (5)	C13—H13A	0.9800
P1—C3	1.848 (4)	C13—H13B	0.9800
P1—C1	1.873 (4)	C13—H13C	0.9800
P1—C2	1.873 (4)	C21—H21A	0.9800
P1—Se1	2.2013 (11)	C21—H21B	0.9800
C1—C12	1.531 (6)	C21—H21C	0.9800
C1—C13	1.533 (6)	C22—H22A	0.9800
C1—C11	1.544 (7)	C22—H22B	0.9800
C2—C23	1.523 (7)	C22—H22C	0.9800
C2—C22	1.532 (7)	C23—H23A	0.9800
C2—C21	1.541 (8)	C23—H23B	0.9800
C3—C32	1.531 (7)	C23—H23C	0.9800
C3—C31	1.539 (7)	C31—H31A	0.9800
C3—H3	1.0000	C31—H31B	0.9800
C11—H11A	0.9800	C31—H31C	0.9800
C11—H11B	0.9800	C32—H32A	0.9800
C11—H11C	0.9800	C32—H32B	0.9800
C12—H12A	0.9800	C32—H32C	0.9800
C12—H12B	0.9800		
			
Se1—Au1—Br1	175.696 (17)	H12B—C12—H12C	109.5
C3—P1—C1	105.4 (2)	C1—C13—H13A	109.5
C3—P1—C2	112.6 (2)	C1—C13—H13B	109.5
C1—P1—C2	114.5 (2)	H13A—C13—H13B	109.5
C3—P1—Se1	103.58 (16)	C1—C13—H13C	109.5
C1—P1—Se1	111.39 (16)	H13A—C13—H13C	109.5
C2—P1—Se1	108.73 (16)	H13B—C13—H13C	109.5
P1—Se1—Au1	105.58 (3)	C2—C21—H21A	109.5
C12—C1—C13	109.1 (4)	C2—C21—H21B	109.5
C12—C1—C11	107.0 (4)	H21A—C21—H21B	109.5
C13—C1—C11	109.5 (4)	C2—C21—H21C	109.5
C12—C1—P1	108.3 (3)	H21A—C21—H21C	109.5
C13—C1—P1	111.4 (3)	H21B—C21—H21C	109.5
C11—C1—P1	111.5 (3)	C2—C22—H22A	109.5
C23—C2—C22	108.4 (4)	C2—C22—H22B	109.5
C23—C2—C21	108.5 (4)	H22A—C22—H22B	109.5
C22—C2—C21	109.7 (5)	C2—C22—H22C	109.5
C23—C2—P1	111.5 (3)	H22A—C22—H22C	109.5
C22—C2—P1	106.8 (4)	H22B—C22—H22C	109.5
C21—C2—P1	111.9 (3)	C2—C23—H23A	109.5
C32—C3—C31	108.9 (5)	C2—C23—H23B	109.5
C32—C3—P1	115.0 (3)	H23A—C23—H23B	109.5
C31—C3—P1	116.6 (4)	C2—C23—H23C	109.5
C32—C3—H3	105.1	H23A—C23—H23C	109.5
C31—C3—H3	105.1	H23B—C23—H23C	109.5
P1—C3—H3	105.1	C3—C31—H31A	109.5
C1—C11—H11A	109.5	C3—C31—H31B	109.5
C1—C11—H11B	109.5	H31A—C31—H31B	109.5
H11A—C11—H11B	109.5	C3—C31—H31C	109.5
C1—C11—H11C	109.5	H31A—C31—H31C	109.5
H11A—C11—H11C	109.5	H31B—C31—H31C	109.5
H11B—C11—H11C	109.5	C3—C32—H32A	109.5
C1—C12—H12A	109.5	C3—C32—H32B	109.5
C1—C12—H12B	109.5	H32A—C32—H32B	109.5
H12A—C12—H12B	109.5	C3—C32—H32C	109.5
C1—C12—H12C	109.5	H32A—C32—H32C	109.5
H12A—C12—H12C	109.5	H32B—C32—H32C	109.5
			
C3—P1—Se1—Au1	165.57 (15)	Se1—P1—C2—C23	52.7 (4)
C1—P1—Se1—Au1	52.70 (15)	C3—P1—C2—C22	48.6 (4)
C2—P1—Se1—Au1	−74.43 (17)	C1—P1—C2—C22	169.1 (4)
C3—P1—C1—C12	−71.7 (4)	Se1—P1—C2—C22	−65.6 (4)
C2—P1—C1—C12	163.9 (3)	C3—P1—C2—C21	−71.4 (5)
Se1—P1—C1—C12	40.0 (3)	C1—P1—C2—C21	49.1 (5)
C3—P1—C1—C13	168.3 (4)	Se1—P1—C2—C21	174.4 (4)
C2—P1—C1—C13	43.9 (4)	C1—P1—C3—C32	147.5 (4)
Se1—P1—C1—C13	−80.0 (4)	C2—P1—C3—C32	−87.0 (5)
C3—P1—C1—C11	45.7 (4)	Se1—P1—C3—C32	30.3 (4)
C2—P1—C1—C11	−78.7 (4)	C1—P1—C3—C31	−83.3 (4)
Se1—P1—C1—C11	157.4 (3)	C2—P1—C3—C31	42.2 (4)
C3—P1—C2—C23	166.9 (3)	Se1—P1—C3—C31	159.5 (4)
C1—P1—C2—C23	−72.6 (4)		

### [Bis(tert-butyl)(propan-2-yl)phosphane selenide-κSe]bromidogold (7b) Hydrogen-bond geometry (Å, º)

**Table:**

*D*—H···*A*	*D*—H	H···*A*	*D*···*A*	*D*—H···*A*
C13—H13*B*···Au1	0.98	2.74	3.650 (6)	155
C23—H23*B*···Au1	0.98	2.90	3.592 (5)	128
C32—H32*B*···Se1	0.98	2.64	3.233 (6)	120
C3—H3···Br1^i^	1.00	2.76	3.677 (4)	153
C13—H13*A*···Br1^ii^	0.98	2.97	3.928 (5)	167
C21—H21*B*···Br1^iii^	0.98	3.07	3.697 (6)	123
C22—H22*C*···Br1^iv^	0.98	3.10	3.921 (5)	142

Symmetry codes: (i) −*x*, *y*−1/2, −*z*+1/2; (ii) *x*, −*y*+3/2, *z*+1/2; (iii) −*x*+1, *y*−1/2, −*z*+1/2; (iv) −*x*+1, −*y*+1, −*z*.

### Bromido(tri-tert-butylphosphane selenide-κSe)gold (8b) Crystal data

**Table:**

[AuBr(C_12_H_27_PSe)]	*F*(000) = 1048
*M**_r_* = 558.14	*D*_x_ = 2.228 Mg m^−^^3^
Monoclinic, *P*2_1_/*c*	Mo *K*α radiation, λ = 0.71073 Å
*a* = 8.29705 (16) Å	Cell parameters from 15187 reflections
*b* = 13.6959 (3) Å	θ = 2.5–30.8°
*c* = 14.6444 (3) Å	µ = 13.50 mm^−^^1^
β = 90.0892 (18)°	*T* = 100 K
*V* = 1664.11 (5) Å^3^	Block, pale orange
*Z* = 4	0.2 × 0.1 × 0.1 mm

### Bromido(tri-tert-butylphosphane selenide-κSe)gold (8b) Data collection

**Table:**

Oxford Diffraction Xcalibur, Eos diffractometer	5005 independent reflections
Radiation source: fine-focus sealed tube	4493 reflections with *I* > 2σ(*I*)
Detector resolution: 16.1419 pixels mm^-1^	*R*_int_ = 0.041
ω scan	θ_max_ = 30.9°, θ_min_ = 2.5°
Absorption correction: multi-scan CrysAlisPro, Version 1.171.35.21 (Rigaku OD, 2020)	*h* = −11→11
*T*_min_ = 0.398, *T*_max_ = 1.000	*k* = −19→19
48617 measured reflections	*l* = −21→20

### Bromido(tri-tert-butylphosphane selenide-κSe)gold (8b) Refinement

**Table:**

Refinement on *F*^2^	Primary atom site location: structure-invariant direct methods
Least-squares matrix: full	Secondary atom site location: difference Fourier map
*R*[*F*^2^ > 2σ(*F*^2^)] = 0.023	Hydrogen site location: inferred from neighbouring sites
*wR*(*F*^2^) = 0.043	H-atom parameters constrained
*S* = 1.11	*w* = 1/[σ^2^(*F*_o_^2^) + (0.0129*P*)^2^ + 2.7511*P*] where *P* = (*F*_o_^2^ + 2*F*_c_^2^)/3
5005 reflections	(Δ/σ)_max_ = 0.003
154 parameters	Δρ_max_ = 1.39 e Å^−^^3^
0 restraints	Δρ_min_ = −0.91 e Å^−^^3^

### Bromido(tri-tert-butylphosphane selenide-κSe)gold (8b) Fractional atomic coordinates and isotropic or equivalent isotropic displacement parameters (Å^2^)

**Table:**

	*x*	*y*	*z*	*U*_iso_*/*U*_eq_	
Au1	0.26368 (2)	0.32467 (2)	0.61575 (2)	0.01604 (3)	
Br1	0.16630 (4)	0.23290 (2)	0.48775 (2)	0.02017 (6)	
P1	0.25185 (8)	0.54948 (5)	0.74590 (5)	0.01046 (13)	
Se1	0.37034 (4)	0.40571 (2)	0.74633 (2)	0.02129 (7)	
C1	0.3046 (4)	0.5991 (2)	0.86343 (18)	0.0166 (6)	
C2	0.0258 (3)	0.5366 (2)	0.7309 (2)	0.0167 (6)	
C3	0.3426 (3)	0.6278 (2)	0.65252 (19)	0.0151 (5)	
C11	0.2808 (4)	0.7110 (2)	0.8708 (2)	0.0209 (6)	
H11A	0.171135	0.728031	0.851524	0.031*	
H11B	0.358748	0.744250	0.831364	0.031*	
H11C	0.297302	0.731656	0.934224	0.031*	
C12	0.4803 (4)	0.5754 (3)	0.8886 (2)	0.0262 (7)	
H12A	0.505662	0.603505	0.948464	0.039*	
H12B	0.552545	0.603058	0.842504	0.039*	
H12C	0.494643	0.504384	0.890925	0.039*	
C13	0.2007 (4)	0.5493 (2)	0.93707 (19)	0.0242 (7)	
H13A	0.235963	0.570954	0.997677	0.036*	
H13B	0.212691	0.478314	0.932374	0.036*	
H13C	0.087356	0.567012	0.927980	0.036*	
C21	−0.0612 (4)	0.6293 (2)	0.7649 (2)	0.0240 (7)	
H21A	−0.015004	0.686781	0.734855	0.036*	
H21B	−0.047493	0.635167	0.831124	0.036*	
H21C	−0.176214	0.624804	0.750205	0.036*	
C22	−0.0362 (4)	0.4462 (3)	0.7834 (2)	0.0283 (7)	
H22A	−0.153537	0.441677	0.776778	0.042*	
H22B	−0.008471	0.452570	0.848202	0.042*	
H22C	0.013995	0.387218	0.758531	0.042*	
C23	−0.0190 (4)	0.5217 (2)	0.6304 (2)	0.0220 (6)	
H23A	−0.134654	0.508052	0.625439	0.033*	
H23B	0.042213	0.466624	0.605609	0.033*	
H23C	0.006633	0.581000	0.595900	0.033*	
C31	0.2556 (4)	0.7263 (2)	0.6405 (2)	0.0211 (6)	
H31A	0.245375	0.758435	0.699948	0.032*	
H31B	0.148198	0.715064	0.614624	0.032*	
H31C	0.317965	0.767955	0.599159	0.032*	
C32	0.5199 (4)	0.6478 (3)	0.6764 (2)	0.0250 (7)	
H32A	0.571076	0.683357	0.626157	0.038*	
H32B	0.576032	0.585706	0.686109	0.038*	
H32C	0.525617	0.687100	0.732260	0.038*	
C33	0.3432 (4)	0.5742 (2)	0.5605 (2)	0.0241 (7)	
H33A	0.232050	0.564088	0.539909	0.036*	
H33B	0.396690	0.510808	0.567588	0.036*	
H33C	0.401388	0.613363	0.515287	0.036*	

### Bromido(tri-tert-butylphosphane selenide-κSe)gold (8b) Atomic displacement parameters (Å^2^)

**Table:**

	*U*^11^	*U*^22^	*U*^33^	*U*^12^	*U*^13^	*U*^23^
Au1	0.01950 (6)	0.00993 (5)	0.01870 (5)	0.00180 (4)	0.00244 (4)	−0.00047 (4)
Br1	0.02619 (15)	0.01570 (14)	0.01861 (13)	−0.00027 (11)	0.00072 (11)	−0.00063 (11)
P1	0.0114 (3)	0.0093 (3)	0.0107 (3)	0.0004 (2)	−0.0003 (2)	0.0001 (2)
Se1	0.02680 (16)	0.01297 (14)	0.02409 (15)	0.00737 (12)	−0.00708 (12)	−0.00134 (12)
C1	0.0213 (14)	0.0168 (14)	0.0116 (12)	−0.0014 (11)	−0.0037 (10)	0.0002 (11)
C2	0.0119 (13)	0.0162 (14)	0.0221 (14)	−0.0021 (11)	0.0003 (11)	−0.0057 (11)
C3	0.0153 (13)	0.0156 (14)	0.0144 (12)	0.0002 (11)	0.0021 (10)	0.0002 (11)
C11	0.0310 (17)	0.0155 (14)	0.0162 (14)	−0.0050 (13)	−0.0023 (12)	−0.0036 (11)
C12	0.0271 (17)	0.0309 (18)	0.0206 (15)	−0.0028 (14)	−0.0128 (13)	0.0012 (13)
C13	0.0391 (19)	0.0226 (16)	0.0110 (13)	−0.0055 (14)	0.0014 (12)	0.0025 (12)
C21	0.0120 (14)	0.0257 (17)	0.0343 (17)	0.0041 (12)	0.0003 (12)	−0.0118 (14)
C22	0.0254 (17)	0.0253 (18)	0.0341 (18)	−0.0118 (14)	0.0065 (14)	−0.0033 (14)
C23	0.0168 (14)	0.0234 (16)	0.0258 (15)	0.0042 (12)	−0.0073 (12)	−0.0095 (13)
C31	0.0344 (18)	0.0149 (15)	0.0140 (13)	0.0024 (12)	0.0005 (12)	0.0031 (11)
C32	0.0172 (15)	0.0252 (17)	0.0326 (17)	−0.0050 (13)	0.0065 (13)	0.0019 (14)
C33	0.0356 (18)	0.0217 (16)	0.0150 (14)	−0.0007 (14)	0.0094 (13)	−0.0018 (12)

### Bromido(tri-tert-butylphosphane selenide-κSe)gold (8b) Geometric parameters (Å, º)

**Table:**

Au1—Se1	2.3805 (3)	C13—H13A	0.9800
Au1—Br1	2.3961 (3)	C13—H13B	0.9800
P1—C3	1.895 (3)	C13—H13C	0.9800
P1—C2	1.896 (3)	C21—H21A	0.9800
P1—C1	1.901 (3)	C21—H21B	0.9800
P1—Se1	2.2008 (7)	C21—H21C	0.9800
C1—C12	1.538 (4)	C22—H22A	0.9800
C1—C13	1.541 (4)	C22—H22B	0.9800
C1—C11	1.549 (4)	C22—H22C	0.9800
C2—C23	1.530 (4)	C23—H23A	0.9800
C2—C21	1.543 (4)	C23—H23B	0.9800
C2—C22	1.545 (4)	C23—H23C	0.9800
C3—C33	1.535 (4)	C31—H31A	0.9800
C3—C32	1.536 (4)	C31—H31B	0.9800
C3—C31	1.539 (4)	C31—H31C	0.9800
C11—H11A	0.9800	C32—H32A	0.9800
C11—H11B	0.9800	C32—H32B	0.9800
C11—H11C	0.9800	C32—H32C	0.9800
C12—H12A	0.9800	C33—H33A	0.9800
C12—H12B	0.9800	C33—H33B	0.9800
C12—H12C	0.9800	C33—H33C	0.9800
			
Se1—Au1—Br1	175.936 (11)	H13A—C13—H13B	109.5
C3—P1—C2	111.30 (13)	C1—C13—H13C	109.5
C3—P1—C1	111.06 (13)	H13A—C13—H13C	109.5
C2—P1—C1	111.39 (13)	H13B—C13—H13C	109.5
C3—P1—Se1	109.31 (9)	C2—C21—H21A	109.5
C2—P1—Se1	111.01 (10)	C2—C21—H21B	109.5
C1—P1—Se1	102.43 (9)	H21A—C21—H21B	109.5
P1—Se1—Au1	104.44 (2)	C2—C21—H21C	109.5
C12—C1—C13	105.6 (2)	H21A—C21—H21C	109.5
C12—C1—C11	108.3 (3)	H21B—C21—H21C	109.5
C13—C1—C11	108.5 (2)	C2—C22—H22A	109.5
C12—C1—P1	111.0 (2)	C2—C22—H22B	109.5
C13—C1—P1	110.3 (2)	H22A—C22—H22B	109.5
C11—C1—P1	112.82 (19)	C2—C22—H22C	109.5
C23—C2—C21	107.8 (3)	H22A—C22—H22C	109.5
C23—C2—C22	107.0 (2)	H22B—C22—H22C	109.5
C21—C2—C22	110.0 (2)	C2—C23—H23A	109.5
C23—C2—P1	111.3 (2)	C2—C23—H23B	109.5
C21—C2—P1	110.4 (2)	H23A—C23—H23B	109.5
C22—C2—P1	110.3 (2)	C2—C23—H23C	109.5
C33—C3—C32	106.3 (2)	H23A—C23—H23C	109.5
C33—C3—C31	108.7 (2)	H23B—C23—H23C	109.5
C32—C3—C31	108.6 (3)	C3—C31—H31A	109.5
C33—C3—P1	111.4 (2)	C3—C31—H31B	109.5
C32—C3—P1	108.5 (2)	H31A—C31—H31B	109.5
C31—C3—P1	113.07 (19)	C3—C31—H31C	109.5
C1—C11—H11A	109.5	H31A—C31—H31C	109.5
C1—C11—H11B	109.5	H31B—C31—H31C	109.5
H11A—C11—H11B	109.5	C3—C32—H32A	109.5
C1—C11—H11C	109.5	C3—C32—H32B	109.5
H11A—C11—H11C	109.5	H32A—C32—H32B	109.5
H11B—C11—H11C	109.5	C3—C32—H32C	109.5
C1—C12—H12A	109.5	H32A—C32—H32C	109.5
C1—C12—H12B	109.5	H32B—C32—H32C	109.5
H12A—C12—H12B	109.5	C3—C33—H33A	109.5
C1—C12—H12C	109.5	C3—C33—H33B	109.5
H12A—C12—H12C	109.5	H33A—C33—H33B	109.5
H12B—C12—H12C	109.5	C3—C33—H33C	109.5
C1—C13—H13A	109.5	H33A—C33—H33C	109.5
C1—C13—H13B	109.5	H33B—C33—H33C	109.5
			
C3—P1—Se1—Au1	−74.10 (10)	C3—P1—C2—C21	−78.5 (2)
C2—P1—Se1—Au1	49.06 (10)	C1—P1—C2—C21	46.0 (3)
C1—P1—Se1—Au1	168.05 (9)	Se1—P1—C2—C21	159.45 (19)
C3—P1—C1—C12	−75.8 (2)	C3—P1—C2—C22	159.7 (2)
C2—P1—C1—C12	159.5 (2)	C1—P1—C2—C22	−75.8 (2)
Se1—P1—C1—C12	40.8 (2)	Se1—P1—C2—C22	37.7 (2)
C3—P1—C1—C13	167.5 (2)	C2—P1—C3—C33	−71.6 (2)
C2—P1—C1—C13	42.8 (3)	C1—P1—C3—C33	163.7 (2)
Se1—P1—C1—C13	−75.9 (2)	Se1—P1—C3—C33	51.4 (2)
C3—P1—C1—C11	45.9 (2)	C2—P1—C3—C32	171.7 (2)
C2—P1—C1—C11	−78.7 (2)	C1—P1—C3—C32	47.0 (2)
Se1—P1—C1—C11	162.5 (2)	Se1—P1—C3—C32	−65.3 (2)
C3—P1—C2—C23	41.1 (2)	C2—P1—C3—C31	51.2 (2)
C1—P1—C2—C23	165.7 (2)	C1—P1—C3—C31	−73.5 (2)
Se1—P1—C2—C23	−80.9 (2)	Se1—P1—C3—C31	174.16 (18)

### Bromido(tri-tert-butylphosphane selenide-κSe)gold (8b) Hydrogen-bond geometry (Å, º)

**Table:**

*D*—H···*A*	*D*—H	H···*A*	*D*···*A*	*D*—H···*A*
C13—H13*B*···Br1^i^	0.98	3.03	3.946 (3)	156
C32—H32*A*···Br1^ii^	0.98	2.98	3.905 (3)	159
C33—H33*B*···Au1	0.98	2.87	3.573 (3)	130
C23—H23*B*···Au1	0.98	2.68	3.582 (3)	153
C12—H12*C*···Se1	0.98	2.71	3.251 (3)	115
C33—H33*B*···Se1	0.98	3.00	3.574 (3)	119
C22—H22*C*···Se1	0.98	2.97	3.462 (3)	112

Symmetry codes: (i) *x*, −*y*+1/2, *z*+1/2; (ii) −*x*+1, −*y*+1, −*z*+1.

### [(tert-Butyl)bis(propan-2-yl)phosphane sulfide-κS]iodidogold (2c) Crystal data

**Table:**

[AuI(C_10_H_23_PS)]	*F*(000) = 984
*M**_r_* = 530.18	*D*_x_ = 2.322 Mg m^−^^3^
Monoclinic, *P*2_1_/*n*	Mo *K*α radiation, λ = 0.71073 Å
*a* = 8.6010 (2) Å	Cell parameters from 18460 reflections
*b* = 15.0435 (3) Å	θ = 2.2–30.8°
*c* = 11.7218 (2) Å	µ = 11.95 mm^−^^1^
β = 91.202 (2)°	*T* = 100 K
*V* = 1516.34 (5) Å^3^	Block, pale yellow
*Z* = 4	0.25 × 0.2 × 0.15 mm

### [(tert-Butyl)bis(propan-2-yl)phosphane sulfide-κS]iodidogold (2c) Data collection

**Table:**

Oxford Diffraction Xcalibur, Eos diffractometer	4580 independent reflections
Radiation source: fine-focus sealed tube	4213 reflections with *I* > 2σ(*I*)
Detector resolution: 16.1419 pixels mm^-1^	*R*_int_ = 0.044
ω scans	θ_max_ = 30.9°, θ_min_ = 2.2°
Absorption correction: multi-scan CrysAlisPro, Version 1.171.35.19 (Rigaku OD, 2020)	*h* = −12→12
*T*_min_ = 0.435, *T*_max_ = 1.000	*k* = −21→21
54148 measured reflections	*l* = −16→16

### [(tert-Butyl)bis(propan-2-yl)phosphane sulfide-κS]iodidogold (2c) Refinement

**Table:**

Refinement on *F*^2^	Secondary atom site location: difference Fourier map
Least-squares matrix: full	Hydrogen site location: inferred from neighbouring sites
*R*[*F*^2^ > 2σ(*F*^2^)] = 0.018	H-atom parameters constrained
*wR*(*F*^2^) = 0.033	*w* = 1/[σ^2^(*F*_o_^2^) + (0.0088*P*)^2^ + 1.0273*P*] where *P* = (*F*_o_^2^ + 2*F*_c_^2^)/3
*S* = 1.12	(Δ/σ)_max_ = 0.001
4580 reflections	Δρ_max_ = 0.92 e Å^−^^3^
135 parameters	Δρ_min_ = −0.68 e Å^−^^3^
0 restraints	Extinction correction: SHELXL-2019/3 (Sheldrick, 2015), Fc^*^=kFc[1+0.001xFc^2^λ^3^/sin(2θ)]^-1/4^
Primary atom site location: structure-invariant direct methods	Extinction coefficient: 0.00148 (4)

### [(tert-Butyl)bis(propan-2-yl)phosphane sulfide-κS]iodidogold (2c) Fractional atomic coordinates and isotropic or equivalent isotropic displacement parameters (Å^2^)

**Table:**

	*x*	*y*	*z*	*U*_iso_*/*U*_eq_	
Au1	0.47494 (2)	0.40378 (2)	0.28431 (2)	0.01328 (3)	
I1	0.74408 (2)	0.33406 (2)	0.27831 (2)	0.01669 (4)	
S1	0.22595 (7)	0.45551 (4)	0.30480 (5)	0.01601 (12)	
P1	0.23375 (7)	0.58788 (4)	0.27079 (5)	0.00961 (11)	
C1	0.3011 (3)	0.61276 (16)	0.12332 (19)	0.0122 (4)	
C2	0.0321 (3)	0.62736 (17)	0.2848 (2)	0.0135 (5)	
H2	0.031468	0.692691	0.269023	0.016*	
C3	0.3454 (3)	0.64662 (16)	0.38423 (19)	0.0128 (4)	
H3	0.294656	0.629094	0.456854	0.015*	
C11	0.2360 (3)	0.70321 (17)	0.0835 (2)	0.0182 (5)	
H11A	0.274795	0.750085	0.134732	0.027*	
H11B	0.122165	0.701820	0.084786	0.027*	
H11C	0.269733	0.715298	0.005688	0.027*	
C12	0.2443 (3)	0.53869 (17)	0.0413 (2)	0.0193 (5)	
H12A	0.276013	0.552714	−0.036485	0.029*	
H12B	0.130673	0.534550	0.043301	0.029*	
H12C	0.290158	0.481811	0.064982	0.029*	
C13	0.4788 (3)	0.61594 (17)	0.1207 (2)	0.0172 (5)	
H13A	0.512145	0.620595	0.041526	0.026*	
H13B	0.521687	0.561583	0.155051	0.026*	
H13C	0.516379	0.667689	0.163971	0.026*	
C21	−0.0821 (3)	0.58348 (18)	0.1996 (2)	0.0195 (5)	
H21A	−0.075648	0.518716	0.207366	0.029*	
H21B	−0.055407	0.600599	0.121796	0.029*	
H21C	−0.188143	0.603120	0.215394	0.029*	
C22	−0.0260 (3)	0.6138 (2)	0.4063 (2)	0.0237 (6)	
H22A	−0.130189	0.639457	0.412544	0.036*	
H22B	0.045174	0.643261	0.460694	0.036*	
H22C	−0.029986	0.550107	0.423391	0.036*	
C31	0.3296 (3)	0.74827 (17)	0.3790 (2)	0.0203 (5)	
H31A	0.368103	0.774178	0.450869	0.030*	
H31B	0.220047	0.764234	0.366862	0.030*	
H31C	0.390690	0.771245	0.315753	0.030*	
C32	0.5158 (3)	0.61983 (17)	0.4007 (2)	0.0177 (5)	
H32A	0.578901	0.650631	0.344340	0.027*	
H32B	0.526015	0.555442	0.390538	0.027*	
H32C	0.551796	0.636229	0.477722	0.027*	

### [(tert-Butyl)bis(propan-2-yl)phosphane sulfide-κS]iodidogold (2c) Atomic displacement parameters (Å^2^)

**Table:**

	*U*^11^	*U*^22^	*U*^33^	*U*^12^	*U*^13^	*U*^23^
Au1	0.01542 (5)	0.00842 (5)	0.01597 (5)	0.00169 (3)	0.00001 (3)	0.00117 (3)
I1	0.01663 (8)	0.01449 (8)	0.01896 (8)	0.00388 (6)	0.00109 (6)	0.00049 (6)
S1	0.0147 (3)	0.0091 (3)	0.0242 (3)	0.0000 (2)	0.0014 (2)	0.0032 (2)
P1	0.0096 (3)	0.0077 (3)	0.0114 (3)	0.0004 (2)	−0.0004 (2)	−0.0001 (2)
C1	0.0146 (11)	0.0111 (11)	0.0109 (10)	0.0008 (9)	−0.0001 (8)	−0.0008 (8)
C2	0.0105 (10)	0.0134 (12)	0.0167 (11)	0.0029 (9)	−0.0011 (8)	−0.0008 (9)
C3	0.0151 (11)	0.0128 (12)	0.0105 (10)	0.0007 (9)	−0.0015 (8)	−0.0014 (9)
C11	0.0254 (13)	0.0139 (12)	0.0154 (11)	0.0012 (10)	0.0005 (10)	0.0037 (9)
C12	0.0265 (13)	0.0180 (13)	0.0135 (11)	0.0000 (11)	−0.0001 (10)	−0.0042 (10)
C13	0.0163 (11)	0.0188 (13)	0.0167 (11)	−0.0009 (10)	0.0043 (9)	−0.0001 (10)
C21	0.0116 (11)	0.0202 (14)	0.0264 (13)	−0.0002 (10)	−0.0029 (10)	−0.0019 (11)
C22	0.0154 (12)	0.0359 (17)	0.0200 (13)	0.0064 (11)	0.0048 (10)	0.0015 (11)
C31	0.0212 (12)	0.0154 (13)	0.0238 (13)	0.0010 (10)	−0.0070 (10)	−0.0039 (10)
C32	0.0157 (11)	0.0174 (13)	0.0198 (12)	−0.0012 (10)	−0.0056 (9)	−0.0036 (10)

### [(tert-Butyl)bis(propan-2-yl)phosphane sulfide-κS]iodidogold (2c) Geometric parameters (Å, º)

**Table:**

Au1—S1	2.2959 (6)	C12—H12A	0.9800
Au1—I1	2.5437 (2)	C12—H12B	0.9800
S1—P1	2.0322 (8)	C12—H12C	0.9800
P1—C2	1.844 (2)	C13—H13A	0.9800
P1—C3	1.848 (2)	C13—H13B	0.9800
P1—C1	1.872 (2)	C13—H13C	0.9800
C1—C13	1.530 (3)	C21—H21A	0.9800
C1—C11	1.540 (3)	C21—H21B	0.9800
C1—C12	1.544 (3)	C21—H21C	0.9800
C2—C22	1.533 (3)	C22—H22A	0.9800
C2—C21	1.535 (3)	C22—H22B	0.9800
C2—H2	1.0000	C22—H22C	0.9800
C3—C32	1.529 (3)	C31—H31A	0.9800
C3—C31	1.536 (3)	C31—H31B	0.9800
C3—H3	1.0000	C31—H31C	0.9800
C11—H11A	0.9800	C32—H32A	0.9800
C11—H11B	0.9800	C32—H32B	0.9800
C11—H11C	0.9800	C32—H32C	0.9800
			
S1—Au1—I1	173.747 (16)	H12A—C12—H12B	109.5
P1—S1—Au1	106.08 (3)	C1—C12—H12C	109.5
C2—P1—C3	104.91 (11)	H12A—C12—H12C	109.5
C2—P1—C1	109.11 (10)	H12B—C12—H12C	109.5
C3—P1—C1	113.74 (11)	C1—C13—H13A	109.5
C2—P1—S1	105.26 (8)	C1—C13—H13B	109.5
C3—P1—S1	110.25 (8)	H13A—C13—H13B	109.5
C1—P1—S1	112.90 (8)	C1—C13—H13C	109.5
C13—C1—C11	108.9 (2)	H13A—C13—H13C	109.5
C13—C1—C12	108.25 (19)	H13B—C13—H13C	109.5
C11—C1—C12	109.92 (19)	C2—C21—H21A	109.5
C13—C1—P1	110.67 (15)	C2—C21—H21B	109.5
C11—C1—P1	109.82 (16)	H21A—C21—H21B	109.5
C12—C1—P1	109.31 (16)	C2—C21—H21C	109.5
C22—C2—C21	109.3 (2)	H21A—C21—H21C	109.5
C22—C2—P1	111.42 (16)	H21B—C21—H21C	109.5
C21—C2—P1	113.20 (17)	C2—C22—H22A	109.5
C22—C2—H2	107.6	C2—C22—H22B	109.5
C21—C2—H2	107.6	H22A—C22—H22B	109.5
P1—C2—H2	107.6	C2—C22—H22C	109.5
C32—C3—C31	110.5 (2)	H22A—C22—H22C	109.5
C32—C3—P1	116.54 (16)	H22B—C22—H22C	109.5
C31—C3—P1	113.76 (16)	C3—C31—H31A	109.5
C32—C3—H3	104.9	C3—C31—H31B	109.5
C31—C3—H3	104.9	H31A—C31—H31B	109.5
P1—C3—H3	104.9	C3—C31—H31C	109.5
C1—C11—H11A	109.5	H31A—C31—H31C	109.5
C1—C11—H11B	109.5	H31B—C31—H31C	109.5
H11A—C11—H11B	109.5	C3—C32—H32A	109.5
C1—C11—H11C	109.5	C3—C32—H32B	109.5
H11A—C11—H11C	109.5	H32A—C32—H32B	109.5
H11B—C11—H11C	109.5	C3—C32—H32C	109.5
C1—C12—H12A	109.5	H32A—C32—H32C	109.5
C1—C12—H12B	109.5	H32B—C32—H32C	109.5
			
Au1—S1—P1—C2	178.84 (8)	C3—P1—C2—C22	−54.3 (2)
Au1—S1—P1—C3	−68.53 (8)	C1—P1—C2—C22	−176.55 (18)
Au1—S1—P1—C1	59.91 (8)	S1—P1—C2—C22	62.02 (19)
C2—P1—C1—C13	157.71 (17)	C3—P1—C2—C21	−178.02 (18)
C3—P1—C1—C13	41.0 (2)	C1—P1—C2—C21	59.8 (2)
S1—P1—C1—C13	−85.62 (17)	S1—P1—C2—C21	−61.66 (18)
C2—P1—C1—C11	37.51 (19)	C2—P1—C3—C32	174.22 (18)
C3—P1—C1—C11	−79.22 (18)	C1—P1—C3—C32	−66.6 (2)
S1—P1—C1—C11	154.18 (14)	S1—P1—C3—C32	61.36 (19)
C2—P1—C1—C12	−83.15 (18)	C2—P1—C3—C31	−55.3 (2)
C3—P1—C1—C12	160.11 (16)	C1—P1—C3—C31	63.8 (2)
S1—P1—C1—C12	33.51 (18)	S1—P1—C3—C31	−168.21 (15)

### [(tert-Butyl)bis(propan-2-yl)phosphane sulfide-κS]iodidogold (2c) Hydrogen-bond geometry (Å, º)

**Table:**

*D*—H···*A*	*D*—H	H···*A*	*D*···*A*	*D*—H···*A*
C13—H13*B*···Au1	0.98	2.85	3.724 (3)	149
C32—H32*B*···Au1	0.98	2.63	3.539 (3)	154
C32—H32*C*···Au1^i^	0.98	2.87	3.709 (3)	144
C3—H3···I1^i^	1.00	3.18	4.056 (2)	147
C2—H2···I1^ii^	1.00	3.22	3.973 (2)	133
C2—H2···Au1^ii^	1.00	3.24	4.237 (3)	179

Symmetry codes: (i) −*x*+1, −*y*+1, −*z*+1; (ii) −*x*+1/2, *y*+1/2, −*z*+1/2.

### [(tert-Butyl)bis(propan-2-yl)phosphane selenide-κSe]iodidogold (6c) Crystal data

**Table:**

[AuI(C_10_H_23_PSe)]	*F*(000) = 2112
*M**_r_* = 577.08	*D*_x_ = 2.493 Mg m^−^^3^
Monoclinic, *P*2_1_/*c*	Mo *K*α radiation, λ = 0.71073 Å
*a* = 11.70073 (16) Å	Cell parameters from 37382 reflections
*b* = 15.4167 (2) Å	θ = 2.4–30.8°
*c* = 17.0480 (2) Å	µ = 14.02 mm^−^^1^
β = 89.6296 (12)°	*T* = 100 K
*V* = 3075.16 (7) Å^3^	Plate, dichroic yellow/orange
*Z* = 8	0.2 × 0.15 × 0.05 mm

### [(tert-Butyl)bis(propan-2-yl)phosphane selenide-κSe]iodidogold (6c) Data collection

**Table:**

Oxford Diffraction Xcalibur, Eos diffractometer	9346 independent reflections
Radiation source: fine-focus sealed tube	8443 reflections with *I* > 2σ(*I*)
Detector resolution: 16.1419 pixels mm^-1^	*R*_int_ = 0.042
ω scans	θ_max_ = 30.9°, θ_min_ = 2.4°
Absorption correction: multi-scan CrysAlisPro, Version 1.171.35.21 (Rigaku OD, 2020)	*h* = −16→16
*T*_min_ = 0.218, *T*_max_ = 1.000	*k* = −22→21
124405 measured reflections	*l* = −24→24

### [(tert-Butyl)bis(propan-2-yl)phosphane selenide-κSe]iodidogold (6c) Refinement

**Table:**

Refinement on *F*^2^	Primary atom site location: structure-invariant direct methods
Least-squares matrix: full	Secondary atom site location: difference Fourier map
*R*[*F*^2^ > 2σ(*F*^2^)] = 0.022	Hydrogen site location: inferred from neighbouring sites
*wR*(*F*^2^) = 0.040	H-atom parameters constrained
*S* = 1.06	*w* = 1/[σ^2^(*F*_o_^2^) + (0.0114*P*)^2^ + 8.2184*P*] where *P* = (*F*_o_^2^ + 2*F*_c_^2^)/3
9346 reflections	(Δ/σ)_max_ = 0.005
267 parameters	Δρ_max_ = 2.96 e Å^−^^3^
0 restraints	Δρ_min_ = −1.28 e Å^−^^3^

### [(tert-Butyl)bis(propan-2-yl)phosphane selenide-κSe]iodidogold (6c) Fractional atomic coordinates and isotropic or equivalent isotropic displacement parameters (Å^2^)

**Table:**

	*x*	*y*	*z*	*U*_iso_*/*U*_eq_	
Au1	0.02373 (2)	0.28708 (2)	0.62927 (2)	0.01457 (3)	
I1	0.03730 (2)	0.22686 (2)	0.49017 (2)	0.01826 (4)	
Se1	0.01777 (3)	0.34130 (2)	0.76145 (2)	0.01670 (6)	
P1	0.00031 (6)	0.48087 (5)	0.73953 (4)	0.01007 (13)	
C1	−0.1253 (2)	0.50960 (19)	0.67729 (17)	0.0148 (5)	
C2	−0.0185 (2)	0.53016 (19)	0.83730 (16)	0.0142 (5)	
H2	−0.029151	0.593898	0.829239	0.017*	
C3	0.1368 (2)	0.52481 (19)	0.70298 (17)	0.0139 (5)	
H3	0.194490	0.507007	0.742897	0.017*	
C11	−0.1667 (3)	0.6019 (2)	0.69746 (19)	0.0201 (6)	
H11A	−0.103484	0.642965	0.690561	0.030*	
H11B	−0.193436	0.603548	0.752033	0.030*	
H11C	−0.229543	0.617926	0.662590	0.030*	
C12	−0.2221 (3)	0.4440 (2)	0.6908 (2)	0.0218 (6)	
H12A	−0.288592	0.460660	0.659558	0.033*	
H12B	−0.243276	0.443431	0.746546	0.033*	
H12C	−0.196330	0.386070	0.675042	0.033*	
C13	−0.0924 (3)	0.5072 (2)	0.59001 (18)	0.0214 (6)	
H13A	−0.056035	0.451559	0.577750	0.032*	
H13B	−0.039042	0.554557	0.578396	0.032*	
H13C	−0.161270	0.514040	0.558146	0.032*	
C21	−0.1242 (3)	0.4970 (2)	0.88149 (18)	0.0217 (6)	
H21A	−0.116297	0.434618	0.891089	0.033*	
H21B	−0.192507	0.507614	0.849970	0.033*	
H21C	−0.131320	0.527547	0.931696	0.033*	
C22	0.0884 (3)	0.5185 (2)	0.88792 (18)	0.0204 (6)	
H22A	0.076079	0.546029	0.939081	0.031*	
H22B	0.154145	0.545453	0.861656	0.031*	
H22C	0.103321	0.456466	0.895340	0.031*	
C31	0.1410 (3)	0.6245 (2)	0.70240 (19)	0.0199 (6)	
H31A	0.219844	0.643676	0.693236	0.030*	
H31B	0.113974	0.646682	0.753076	0.030*	
H31C	0.092012	0.646582	0.660520	0.030*	
C32	0.1816 (3)	0.4879 (2)	0.62553 (19)	0.0216 (6)	
H32A	0.145100	0.518119	0.581728	0.032*	
H32B	0.163873	0.425895	0.622762	0.032*	
H32C	0.264549	0.496161	0.622391	0.032*	
Au2	0.48954 (2)	0.42652 (2)	0.36350 (2)	0.01453 (3)	
I2	0.54320 (2)	0.47382 (2)	0.22472 (2)	0.01969 (4)	
Se2	0.43272 (3)	0.39455 (2)	0.49591 (2)	0.01620 (6)	
P2	0.47803 (6)	0.25760 (5)	0.50926 (4)	0.00976 (13)	
C4	0.6276 (2)	0.23204 (19)	0.47698 (17)	0.0131 (5)	
C5	0.4635 (2)	0.23485 (19)	0.61467 (16)	0.0141 (5)	
H5	0.483716	0.172435	0.622506	0.017*	
C6	0.3689 (2)	0.18956 (19)	0.46232 (17)	0.0140 (5)	
H6	0.295298	0.207261	0.487758	0.017*	
C41	0.6717 (3)	0.1523 (2)	0.52198 (19)	0.0198 (6)	
H41A	0.748070	0.136909	0.502561	0.030*	
H41B	0.619560	0.103450	0.513965	0.030*	
H41C	0.675619	0.165933	0.578052	0.030*	
C42	0.7056 (2)	0.3105 (2)	0.49220 (19)	0.0194 (6)	
H42A	0.784647	0.295450	0.478628	0.029*	
H42B	0.701058	0.326614	0.547749	0.029*	
H42C	0.680752	0.359547	0.459914	0.029*	
C43	0.6306 (3)	0.2116 (2)	0.38882 (18)	0.0178 (6)	
H43A	0.595806	0.259612	0.359763	0.027*	
H43B	0.587848	0.158149	0.378860	0.027*	
H43C	0.710046	0.204177	0.371563	0.027*	
C51	0.5445 (3)	0.2885 (2)	0.66618 (18)	0.0220 (7)	
H51A	0.530862	0.350399	0.657231	0.033*	
H51B	0.623891	0.274446	0.652607	0.033*	
H51C	0.530385	0.274767	0.721530	0.033*	
C52	0.3399 (3)	0.2466 (2)	0.64344 (19)	0.0252 (7)	
H52A	0.334454	0.230424	0.698938	0.038*	
H52B	0.289075	0.209464	0.612637	0.038*	
H52C	0.317131	0.307351	0.637118	0.038*	
C61	0.3802 (3)	0.0926 (2)	0.48032 (19)	0.0200 (6)	
H61A	0.313277	0.061776	0.460127	0.030*	
H61B	0.384871	0.084174	0.537187	0.030*	
H61C	0.449488	0.069870	0.455182	0.030*	
C62	0.3505 (3)	0.2053 (2)	0.37438 (17)	0.0177 (6)	
H62A	0.407836	0.172956	0.344128	0.027*	
H62B	0.357873	0.267345	0.363065	0.027*	
H62C	0.273859	0.185530	0.359804	0.027*	

### [(tert-Butyl)bis(propan-2-yl)phosphane selenide-κSe]iodidogold (6c) Atomic displacement parameters (Å^2^)

**Table:**

	*U*^11^	*U*^22^	*U*^33^	*U*^12^	*U*^13^	*U*^23^
Au1	0.01684 (5)	0.01111 (5)	0.01576 (5)	0.00094 (4)	−0.00074 (4)	−0.00190 (4)
I1	0.01677 (9)	0.01974 (10)	0.01825 (9)	0.00175 (7)	0.00029 (7)	−0.00359 (7)
Se1	0.02671 (15)	0.01004 (13)	0.01338 (13)	0.00031 (11)	−0.00245 (11)	0.00175 (10)
P1	0.0116 (3)	0.0101 (3)	0.0085 (3)	0.0000 (2)	−0.0002 (2)	0.0004 (2)
C1	0.0143 (13)	0.0160 (14)	0.0142 (13)	0.0032 (11)	−0.0041 (10)	−0.0011 (11)
C2	0.0169 (13)	0.0151 (14)	0.0106 (12)	−0.0034 (11)	0.0012 (10)	−0.0015 (11)
C3	0.0131 (12)	0.0132 (13)	0.0153 (13)	−0.0004 (10)	0.0010 (10)	−0.0001 (11)
C11	0.0197 (14)	0.0190 (15)	0.0217 (15)	0.0077 (12)	−0.0040 (12)	−0.0030 (12)
C12	0.0159 (14)	0.0252 (17)	0.0243 (16)	−0.0009 (12)	−0.0046 (12)	−0.0020 (13)
C13	0.0265 (16)	0.0254 (17)	0.0124 (14)	0.0050 (13)	−0.0059 (12)	−0.0008 (12)
C21	0.0225 (15)	0.0282 (17)	0.0145 (14)	−0.0026 (13)	0.0064 (12)	−0.0028 (13)
C22	0.0260 (16)	0.0227 (16)	0.0127 (14)	−0.0024 (13)	−0.0035 (12)	−0.0028 (12)
C31	0.0226 (15)	0.0146 (14)	0.0224 (16)	−0.0059 (12)	0.0032 (12)	−0.0002 (12)
C32	0.0193 (14)	0.0224 (16)	0.0229 (16)	−0.0036 (12)	0.0074 (12)	−0.0038 (13)
Au2	0.01696 (5)	0.01136 (5)	0.01528 (5)	0.00049 (4)	−0.00074 (4)	0.00295 (4)
I2	0.02684 (10)	0.01703 (9)	0.01519 (9)	−0.00158 (8)	0.00086 (7)	0.00009 (7)
Se2	0.02334 (14)	0.00966 (13)	0.01557 (14)	0.00423 (11)	0.00330 (11)	0.00110 (10)
P2	0.0110 (3)	0.0089 (3)	0.0093 (3)	0.0004 (2)	0.0000 (2)	0.0004 (2)
C4	0.0118 (12)	0.0139 (13)	0.0136 (13)	0.0031 (10)	−0.0003 (10)	0.0012 (10)
C5	0.0165 (13)	0.0162 (14)	0.0097 (12)	0.0003 (11)	0.0008 (10)	0.0010 (10)
C6	0.0129 (12)	0.0147 (14)	0.0145 (13)	−0.0017 (10)	−0.0016 (10)	−0.0011 (11)
C41	0.0162 (13)	0.0200 (15)	0.0231 (16)	0.0076 (11)	0.0020 (11)	0.0053 (12)
C42	0.0141 (13)	0.0226 (16)	0.0217 (15)	−0.0024 (12)	−0.0001 (11)	0.0014 (12)
C43	0.0181 (14)	0.0198 (15)	0.0153 (14)	0.0012 (11)	0.0038 (11)	−0.0004 (12)
C51	0.0283 (16)	0.0250 (17)	0.0127 (14)	−0.0048 (13)	−0.0026 (12)	−0.0019 (12)
C52	0.0230 (16)	0.035 (2)	0.0175 (15)	0.0029 (14)	0.0067 (12)	0.0051 (14)
C61	0.0247 (15)	0.0148 (14)	0.0205 (15)	−0.0071 (12)	−0.0035 (12)	−0.0002 (12)
C62	0.0197 (14)	0.0189 (15)	0.0145 (14)	0.0001 (12)	−0.0064 (11)	−0.0013 (11)

### [(tert-Butyl)bis(propan-2-yl)phosphane selenide-κSe]iodidogold (6c) Geometric parameters (Å, º)

**Table:**

Au1—Se1	2.4040 (3)	Au2—Se2	2.4002 (3)
Au1—I1	2.5508 (2)	Au2—I2	2.5503 (2)
Se1—P1	2.1938 (8)	Se2—P2	2.1890 (8)
P1—C3	1.840 (3)	P2—C5	1.838 (3)
P1—C2	1.844 (3)	P2—C6	1.839 (3)
P1—C1	1.871 (3)	P2—C4	1.873 (3)
C1—C13	1.535 (4)	C4—C43	1.536 (4)
C1—C12	1.535 (4)	C4—C42	1.539 (4)
C1—C11	1.542 (4)	C4—C41	1.539 (4)
C2—C21	1.531 (4)	C5—C52	1.535 (4)
C2—C22	1.535 (4)	C5—C51	1.537 (4)
C2—H2	1.0000	C5—H5	1.0000
C3—C32	1.527 (4)	C6—C61	1.532 (4)
C3—C31	1.537 (4)	C6—C62	1.535 (4)
C3—H3	1.0000	C6—H6	1.0000
C11—H11A	0.9800	C41—H41A	0.9800
C11—H11B	0.9800	C41—H41B	0.9800
C11—H11C	0.9800	C41—H41C	0.9800
C12—H12A	0.9800	C42—H42A	0.9800
C12—H12B	0.9800	C42—H42B	0.9800
C12—H12C	0.9800	C42—H42C	0.9800
C13—H13A	0.9800	C43—H43A	0.9800
C13—H13B	0.9800	C43—H43B	0.9800
C13—H13C	0.9800	C43—H43C	0.9800
C21—H21A	0.9800	C51—H51A	0.9800
C21—H21B	0.9800	C51—H51B	0.9800
C21—H21C	0.9800	C51—H51C	0.9800
C22—H22A	0.9800	C52—H52A	0.9800
C22—H22B	0.9800	C52—H52B	0.9800
C22—H22C	0.9800	C52—H52C	0.9800
C31—H31A	0.9800	C61—H61A	0.9800
C31—H31B	0.9800	C61—H61B	0.9800
C31—H31C	0.9800	C61—H61C	0.9800
C32—H32A	0.9800	C62—H62A	0.9800
C32—H32B	0.9800	C62—H62B	0.9800
C32—H32C	0.9800	C62—H62C	0.9800
			
Se1—Au1—I1	177.832 (10)	Se2—Au2—I2	175.009 (10)
P1—Se1—Au1	100.57 (2)	P2—Se2—Au2	103.30 (2)
C3—P1—C2	104.65 (13)	C5—P2—C6	104.86 (13)
C3—P1—C1	113.83 (14)	C5—P2—C4	109.11 (13)
C2—P1—C1	108.98 (13)	C6—P2—C4	113.70 (13)
C3—P1—Se1	109.71 (10)	C5—P2—Se2	105.35 (10)
C2—P1—Se1	105.13 (10)	C6—P2—Se2	109.60 (10)
C1—P1—Se1	113.77 (10)	C4—P2—Se2	113.51 (9)
C13—C1—C12	108.1 (3)	C43—C4—C42	108.4 (2)
C13—C1—C11	108.3 (3)	C43—C4—C41	108.6 (2)
C12—C1—C11	110.1 (2)	C42—C4—C41	110.0 (2)
C13—C1—P1	110.6 (2)	C43—C4—P2	110.23 (19)
C12—C1—P1	109.9 (2)	C42—C4—P2	109.8 (2)
C11—C1—P1	109.8 (2)	C41—C4—P2	109.71 (19)
C21—C2—C22	110.0 (3)	C52—C5—C51	109.7 (3)
C21—C2—P1	113.5 (2)	C52—C5—P2	111.8 (2)
C22—C2—P1	111.5 (2)	C51—C5—P2	113.7 (2)
C21—C2—H2	107.2	C52—C5—H5	107.1
C22—C2—H2	107.2	C51—C5—H5	107.1
P1—C2—H2	107.2	P2—C5—H5	107.1
C32—C3—C31	110.8 (2)	C61—C6—C62	111.3 (2)
C32—C3—P1	116.6 (2)	C61—C6—P2	114.1 (2)
C31—C3—P1	113.5 (2)	C62—C6—P2	115.9 (2)
C32—C3—H3	104.9	C61—C6—H6	104.7
C31—C3—H3	104.9	C62—C6—H6	104.7
P1—C3—H3	104.9	P2—C6—H6	104.7
C1—C11—H11A	109.5	C4—C41—H41A	109.5
C1—C11—H11B	109.5	C4—C41—H41B	109.5
H11A—C11—H11B	109.5	H41A—C41—H41B	109.5
C1—C11—H11C	109.5	C4—C41—H41C	109.5
H11A—C11—H11C	109.5	H41A—C41—H41C	109.5
H11B—C11—H11C	109.5	H41B—C41—H41C	109.5
C1—C12—H12A	109.5	C4—C42—H42A	109.5
C1—C12—H12B	109.5	C4—C42—H42B	109.5
H12A—C12—H12B	109.5	H42A—C42—H42B	109.5
C1—C12—H12C	109.5	C4—C42—H42C	109.5
H12A—C12—H12C	109.5	H42A—C42—H42C	109.5
H12B—C12—H12C	109.5	H42B—C42—H42C	109.5
C1—C13—H13A	109.5	C4—C43—H43A	109.5
C1—C13—H13B	109.5	C4—C43—H43B	109.5
H13A—C13—H13B	109.5	H43A—C43—H43B	109.5
C1—C13—H13C	109.5	C4—C43—H43C	109.5
H13A—C13—H13C	109.5	H43A—C43—H43C	109.5
H13B—C13—H13C	109.5	H43B—C43—H43C	109.5
C2—C21—H21A	109.5	C5—C51—H51A	109.5
C2—C21—H21B	109.5	C5—C51—H51B	109.5
H21A—C21—H21B	109.5	H51A—C51—H51B	109.5
C2—C21—H21C	109.5	C5—C51—H51C	109.5
H21A—C21—H21C	109.5	H51A—C51—H51C	109.5
H21B—C21—H21C	109.5	H51B—C51—H51C	109.5
C2—C22—H22A	109.5	C5—C52—H52A	109.5
C2—C22—H22B	109.5	C5—C52—H52B	109.5
H22A—C22—H22B	109.5	H52A—C52—H52B	109.5
C2—C22—H22C	109.5	C5—C52—H52C	109.5
H22A—C22—H22C	109.5	H52A—C52—H52C	109.5
H22B—C22—H22C	109.5	H52B—C52—H52C	109.5
C3—C31—H31A	109.5	C6—C61—H61A	109.5
C3—C31—H31B	109.5	C6—C61—H61B	109.5
H31A—C31—H31B	109.5	H61A—C61—H61B	109.5
C3—C31—H31C	109.5	C6—C61—H61C	109.5
H31A—C31—H31C	109.5	H61A—C61—H61C	109.5
H31B—C31—H31C	109.5	H61B—C61—H61C	109.5
C3—C32—H32A	109.5	C6—C62—H62A	109.5
C3—C32—H32B	109.5	C6—C62—H62B	109.5
H32A—C32—H32B	109.5	H62A—C62—H62B	109.5
C3—C32—H32C	109.5	C6—C62—H62C	109.5
H32A—C32—H32C	109.5	H62A—C62—H62C	109.5
H32B—C32—H32C	109.5	H62B—C62—H62C	109.5
			
Au1—Se1—P1—C3	−72.96 (10)	Au2—Se2—P2—C5	169.01 (9)
Au1—Se1—P1—C2	175.01 (9)	Au2—Se2—P2—C6	−78.65 (10)
Au1—Se1—P1—C1	55.84 (10)	Au2—Se2—P2—C4	49.69 (10)
C3—P1—C1—C13	38.2 (3)	C5—P2—C4—C43	155.4 (2)
C2—P1—C1—C13	154.6 (2)	C6—P2—C4—C43	38.7 (2)
Se1—P1—C1—C13	−88.5 (2)	Se2—P2—C4—C43	−87.5 (2)
C3—P1—C1—C12	157.4 (2)	C5—P2—C4—C42	−85.2 (2)
C2—P1—C1—C12	−86.2 (2)	C6—P2—C4—C42	158.2 (2)
Se1—P1—C1—C12	30.8 (2)	Se2—P2—C4—C42	32.0 (2)
C3—P1—C1—C11	−81.3 (2)	C5—P2—C4—C41	35.9 (2)
C2—P1—C1—C11	35.1 (2)	C6—P2—C4—C41	−80.8 (2)
Se1—P1—C1—C11	152.03 (18)	Se2—P2—C4—C41	153.02 (18)
C3—P1—C2—C21	−175.8 (2)	C6—P2—C5—C52	−52.7 (3)
C1—P1—C2—C21	62.1 (3)	C4—P2—C5—C52	−174.8 (2)
Se1—P1—C2—C21	−60.2 (2)	Se2—P2—C5—C52	63.0 (2)
C3—P1—C2—C22	−50.9 (2)	C6—P2—C5—C51	−177.6 (2)
C1—P1—C2—C22	−173.0 (2)	C4—P2—C5—C51	60.3 (3)
Se1—P1—C2—C22	64.7 (2)	Se2—P2—C5—C51	−61.9 (2)
C2—P1—C3—C32	173.9 (2)	C5—P2—C6—C61	−57.1 (2)
C1—P1—C3—C32	−67.2 (3)	C4—P2—C6—C61	62.0 (3)
Se1—P1—C3—C32	61.6 (2)	Se2—P2—C6—C61	−169.73 (19)
C2—P1—C3—C31	−55.5 (2)	C5—P2—C6—C62	171.7 (2)
C1—P1—C3—C31	63.4 (3)	C4—P2—C6—C62	−69.2 (2)
Se1—P1—C3—C31	−167.81 (19)	Se2—P2—C6—C62	59.0 (2)

### [(tert-Butyl)bis(propan-2-yl)phosphane selenide-κSe]iodidogold (6c) Hydrogen-bond geometry (Å, º)

**Table:**

*D*—H···*A*	*D*—H	H···*A*	*D*···*A*	*D*—H···*A*
C13—H13*A*···Au1	0.98	2.84	3.718 (3)	149
C32—H32*B*···Au1	0.98	2.70	3.606 (3)	154
C43—H43*A*···Au2	0.98	2.86	3.727 (3)	148
C62—H62*B*···Au2	0.98	2.90	3.783 (3)	151
C5—H5···I2^i^	1.00	2.94	3.842 (3)	151
C6—H6···I1	1.00	3.03	3.948 (3)	153
C3—H3···I2^ii^	1.00	3.14	3.949 (3)	139
C42—H42*A*···I1^iii^	0.98	3.15	4.090 (3)	162
C2—H2···Au1^iv^	1.00	3.06	4.002 (3)	157

Symmetry codes: (i) *x*, −*y*+1/2, *z*+1/2; (ii) −*x*+1, −*y*+1, −*z*+1; (iii) *x*+1, *y*, *z*; (iv) −*x*, *y*+1/2, −*z*+3/2.

### [Bis(tert-butyl)(propan-2-yl)phosphane selenide-κSe]iodidogold (7c) Crystal data

**Table:**

[AuI(C_11_H_25_PSe)]	*D*_x_ = 2.383 Mg m^−^^3^
*M**_r_* = 591.10	Mo *K*α radiation, λ = 0.71073 Å
Tetragonal, *P*4_3_2_1_2	Cell parameters from 52249 reflections
*a* = 10.7755 (2) Å	θ = 2.2–30.8°
*c* = 28.3769 (5) Å	µ = 13.09 mm^−^^1^
*V* = 3294.86 (14) Å^3^	*T* = 100 K
*Z* = 8	Plate, colourless
*F*(000) = 2176	0.35 × 0.2 × 0.15 mm

### [Bis(tert-butyl)(propan-2-yl)phosphane selenide-κSe]iodidogold (7c) Data collection

**Table:**

Oxford Diffraction Xcalibur, Eos diffractometer	4829 independent reflections
Radiation source: fine-focus sealed tube	4768 reflections with *I* > 2σ(*I*)
Detector resolution: 16.1419 pixels mm^-1^	*R*_int_ = 0.044
ω scans	θ_max_ = 30.0°, θ_min_ = 2.4°
Absorption correction: multi-scan CrysAlisPro, Version 1.171.35.11 (Rigaku OD, 2020)	*h* = −15→15
*T*_min_ = 0.092, *T*_max_ = 0.244	*k* = −15→15
140787 measured reflections	*l* = −39→39

### [Bis(tert-butyl)(propan-2-yl)phosphane selenide-κSe]iodidogold (7c) Refinement

**Table:**

Refinement on *F*^2^	Secondary atom site location: difference Fourier map
Least-squares matrix: full	Hydrogen site location: inferred from neighbouring sites
*R*[*F*^2^ > 2σ(*F*^2^)] = 0.032	H-atom parameters constrained
*wR*(*F*^2^) = 0.078	*w* = 1/[σ^2^(*F*_o_^2^) + 46.6959*P*] where *P* = (*F*_o_^2^ + 2*F*_c_^2^)/3
*S* = 1.42	(Δ/σ)_max_ = 0.001
4829 reflections	Δρ_max_ = 1.40 e Å^−^^3^
145 parameters	Δρ_min_ = −1.51 e Å^−^^3^
66 restraints	Absolute structure: Refined as an inversion twin
Primary atom site location: structure-invariant direct methods	Absolute structure parameter: 0.086 (12)

### [Bis(tert-butyl)(propan-2-yl)phosphane selenide-κSe]iodidogold (7c) Special details

**Table:**

Geometry. All esds (except the esd in the dihedral angle between two l.s. planes) are estimated using the full covariance matrix. The cell esds are taken into account individually in the estimation of esds in distances, angles and torsion angles; correlations between esds in cell parameters are only used when they are defined by crystal symmetry. An approximate (isotropic) treatment of cell esds is used for estimating esds involving l.s. planes.

### [Bis(tert-butyl)(propan-2-yl)phosphane selenide-κSe]iodidogold (7c) Fractional atomic coordinates and isotropic or equivalent isotropic displacement parameters (Å^2^)

**Table:**

	*x*	*y*	*z*	*U*_iso_*/*U*_eq_	
Au1	0.10475 (3)	0.01896 (4)	0.29317 (2)	0.01310 (8)	
I1	−0.06625 (6)	0.01598 (7)	0.35532 (2)	0.01677 (13)	
P1	0.4384 (2)	0.0280 (3)	0.27711 (9)	0.0108 (4)	
Se1	0.26671 (10)	0.03495 (11)	0.23536 (4)	0.0154 (2)	
C1	0.4696 (11)	−0.1368 (10)	0.2952 (4)	0.021 (2)	
C2	0.4370 (11)	0.1386 (11)	0.3281 (4)	0.017 (2)	
C3	0.5552 (10)	0.0711 (11)	0.2319 (4)	0.017 (2)	
H3	0.535450	0.016609	0.204401	0.021*	
C11	0.5839 (11)	−0.1471 (13)	0.3288 (5)	0.026 (3)	
H11A	0.563922	−0.108623	0.359150	0.039*	
H11B	0.655008	−0.104467	0.314634	0.039*	
H11C	0.604403	−0.234785	0.333673	0.039*	
C12	0.4959 (12)	−0.2106 (12)	0.2497 (5)	0.032 (3)	
H12A	0.502361	−0.299102	0.257209	0.048*	
H12B	0.574083	−0.181913	0.235796	0.048*	
H12C	0.428115	−0.197522	0.227246	0.048*	
C13	0.3540 (13)	−0.1964 (12)	0.3179 (5)	0.029 (3)	
H13A	0.285826	−0.197354	0.294975	0.044*	
H13B	0.329194	−0.148209	0.345581	0.044*	
H13C	0.373363	−0.281666	0.327387	0.044*	
C21	0.5720 (11)	0.1712 (12)	0.3417 (4)	0.022 (2)	
H21A	0.612486	0.213461	0.315245	0.034*	
H21B	0.617442	0.094919	0.349167	0.034*	
H21C	0.571818	0.225956	0.369277	0.034*	
C22	0.3696 (11)	0.2575 (12)	0.3139 (5)	0.027 (3)	
H22A	0.280471	0.240883	0.311313	0.040*	
H22B	0.401378	0.286347	0.283482	0.040*	
H22C	0.383792	0.321443	0.337872	0.040*	
C23	0.3693 (11)	0.0844 (13)	0.3704 (4)	0.024 (3)	
H23A	0.383259	0.137216	0.397969	0.037*	
H23B	0.400907	0.000742	0.376757	0.037*	
H23C	0.280248	0.080119	0.363613	0.037*	
C31	0.6937 (10)	0.0481 (13)	0.2414 (5)	0.025 (3)	
H31A	0.741704	0.068017	0.213067	0.038*	
H31B	0.706479	−0.039266	0.249700	0.038*	
H31C	0.721297	0.100862	0.267518	0.038*	
C32	0.5353 (14)	0.2050 (13)	0.2131 (4)	0.031 (3)	
H32A	0.572170	0.264555	0.235183	0.046*	
H32B	0.446257	0.221538	0.210131	0.046*	
H32C	0.575086	0.213473	0.182239	0.046*	

### [Bis(tert-butyl)(propan-2-yl)phosphane selenide-κSe]iodidogold (7c) Atomic displacement parameters (Å^2^)

**Table:**

	*U*^11^	*U*^22^	*U*^33^	*U*^12^	*U*^13^	*U*^23^
Au1	0.01105 (16)	0.01416 (16)	0.01408 (15)	0.00213 (13)	−0.00131 (13)	−0.00219 (13)
I1	0.0170 (3)	0.0162 (3)	0.0172 (3)	0.0034 (2)	0.0030 (2)	−0.0005 (2)
P1	0.0105 (10)	0.0121 (11)	0.0097 (10)	0.0007 (9)	0.0008 (8)	−0.0005 (9)
Se1	0.0131 (4)	0.0235 (5)	0.0096 (4)	−0.0014 (4)	−0.0020 (3)	−0.0017 (4)
C1	0.019 (5)	0.017 (5)	0.028 (5)	0.003 (4)	0.000 (5)	0.002 (4)
C2	0.019 (5)	0.022 (5)	0.011 (4)	−0.004 (4)	−0.003 (4)	−0.002 (4)
C3	0.014 (4)	0.023 (5)	0.015 (4)	−0.009 (4)	0.003 (4)	0.001 (4)
C11	0.010 (5)	0.026 (6)	0.043 (7)	0.004 (4)	−0.006 (5)	0.009 (5)
C12	0.022 (6)	0.019 (5)	0.055 (8)	−0.007 (5)	0.010 (6)	−0.012 (5)
C13	0.023 (6)	0.020 (6)	0.045 (7)	−0.003 (5)	0.009 (5)	0.014 (5)
C21	0.020 (6)	0.024 (6)	0.024 (6)	0.000 (4)	−0.007 (4)	−0.013 (4)
C22	0.019 (6)	0.025 (6)	0.036 (6)	0.007 (4)	−0.006 (5)	−0.013 (5)
C23	0.018 (5)	0.039 (7)	0.017 (5)	0.006 (5)	0.000 (4)	−0.004 (5)
C31	0.010 (5)	0.038 (7)	0.028 (6)	−0.002 (5)	0.006 (4)	0.003 (5)
C32	0.034 (7)	0.035 (7)	0.023 (6)	−0.014 (6)	0.000 (5)	0.012 (5)

### [Bis(tert-butyl)(propan-2-yl)phosphane selenide-κSe]iodidogold (7c) Geometric parameters (Å, º)

**Table:**

Au1—Se1	2.4014 (11)	C12—H12B	0.9800
Au1—I1	2.5509 (8)	C12—H12C	0.9800
Au1—Au1^i^	3.0914 (8)	C13—H13A	0.9800
P1—C3	1.856 (11)	C13—H13B	0.9800
P1—C2	1.876 (11)	C13—H13C	0.9800
P1—C1	1.878 (11)	C21—H21A	0.9800
P1—Se1	2.198 (3)	C21—H21B	0.9800
C1—C13	1.542 (17)	C21—H21C	0.9800
C1—C12	1.543 (18)	C22—H22A	0.9800
C1—C11	1.561 (17)	C22—H22B	0.9800
C2—C23	1.520 (16)	C22—H22C	0.9800
C2—C22	1.526 (17)	C23—H23A	0.9800
C2—C21	1.546 (16)	C23—H23B	0.9800
C3—C31	1.537 (16)	C23—H23C	0.9800
C3—C32	1.553 (17)	C31—H31A	0.9800
C3—H3	1.0000	C31—H31B	0.9800
C11—H11A	0.9800	C31—H31C	0.9800
C11—H11B	0.9800	C32—H32A	0.9800
C11—H11C	0.9800	C32—H32B	0.9800
C12—H12A	0.9800	C32—H32C	0.9800
			
Se1—Au1—I1	176.56 (4)	H12A—C12—H12C	109.5
Se1—Au1—Au1^i^	78.63 (3)	H12B—C12—H12C	109.5
I1—Au1—Au1^i^	103.36 (2)	C1—C13—H13A	109.5
C3—P1—C2	112.3 (5)	C1—C13—H13B	109.5
C3—P1—C1	107.7 (5)	H13A—C13—H13B	109.5
C2—P1—C1	113.0 (5)	C1—C13—H13C	109.5
C3—P1—Se1	100.9 (4)	H13A—C13—H13C	109.5
C2—P1—Se1	112.8 (4)	H13B—C13—H13C	109.5
C1—P1—Se1	109.3 (4)	C2—C21—H21A	109.5
P1—Se1—Au1	103.94 (8)	C2—C21—H21B	109.5
C13—C1—C12	106.4 (10)	H21A—C21—H21B	109.5
C13—C1—C11	110.7 (10)	C2—C21—H21C	109.5
C12—C1—C11	109.2 (10)	H21A—C21—H21C	109.5
C13—C1—P1	111.3 (8)	H21B—C21—H21C	109.5
C12—C1—P1	106.9 (9)	C2—C22—H22A	109.5
C11—C1—P1	112.1 (8)	C2—C22—H22B	109.5
C23—C2—C22	107.6 (10)	H22A—C22—H22B	109.5
C23—C2—C21	110.0 (9)	C2—C22—H22C	109.5
C22—C2—C21	108.8 (10)	H22A—C22—H22C	109.5
C23—C2—P1	111.6 (8)	H22B—C22—H22C	109.5
C22—C2—P1	109.5 (8)	C2—C23—H23A	109.5
C21—C2—P1	109.2 (8)	C2—C23—H23B	109.5
C31—C3—C32	110.1 (10)	H23A—C23—H23B	109.5
C31—C3—P1	119.8 (8)	C2—C23—H23C	109.5
C32—C3—P1	112.1 (9)	H23A—C23—H23C	109.5
C31—C3—H3	104.4	H23B—C23—H23C	109.5
C32—C3—H3	104.4	C3—C31—H31A	109.5
P1—C3—H3	104.4	C3—C31—H31B	109.5
C1—C11—H11A	109.5	H31A—C31—H31B	109.5
C1—C11—H11B	109.5	C3—C31—H31C	109.5
H11A—C11—H11B	109.5	H31A—C31—H31C	109.5
C1—C11—H11C	109.5	H31B—C31—H31C	109.5
H11A—C11—H11C	109.5	C3—C32—H32A	109.5
H11B—C11—H11C	109.5	C3—C32—H32B	109.5
C1—C12—H12A	109.5	H32A—C32—H32B	109.5
C1—C12—H12B	109.5	C3—C32—H32C	109.5
H12A—C12—H12B	109.5	H32A—C32—H32C	109.5
C1—C12—H12C	109.5	H32B—C32—H32C	109.5
			
C3—P1—Se1—Au1	−169.6 (4)	C3—P1—C2—C23	−164.0 (8)
C2—P1—Se1—Au1	−49.5 (4)	C1—P1—C2—C23	−41.8 (10)
C1—P1—Se1—Au1	77.1 (4)	Se1—P1—C2—C23	82.8 (8)
C3—P1—C1—C13	−159.2 (9)	C3—P1—C2—C22	77.0 (9)
I1—Au1—Au1^i^—I1^i^	117.89 (4)	C1—P1—C2—C22	−160.9 (8)
Se1—Au1—Au1^i^—Se1^i^	123.62 (6)	Se1—P1—C2—C22	−36.3 (9)
I1—Au1—Au1^i^—Se1^i^	−59.25 (3)	C3—P1—C2—C21	−42.1 (10)
C2—P1—C1—C13	76.2 (10)	C1—P1—C2—C21	80.0 (9)
Se1—P1—C1—C13	−50.3 (10)	Se1—P1—C2—C21	−155.4 (7)
C3—P1—C1—C12	−43.4 (9)	C2—P1—C3—C31	74.6 (11)
C2—P1—C1—C12	−168.0 (8)	C1—P1—C3—C31	−50.5 (11)
Se1—P1—C1—C12	65.5 (9)	Se1—P1—C3—C31	−165.0 (9)
C3—P1—C1—C11	76.3 (10)	C2—P1—C3—C32	−56.8 (10)
C2—P1—C1—C11	−48.4 (10)	C1—P1—C3—C32	178.1 (8)
Se1—P1—C1—C11	−174.9 (8)	Se1—P1—C3—C32	63.6 (8)

Symmetry code: (i) −*y*, −*x*, −*z*+1/2.

### [Bis(tert-butyl)(propan-2-yl)phosphane selenide-κSe]iodidogold (7c) Hydrogen-bond geometry (Å, º)

**Table:**

*D*—H···*A*	*D*—H	H···*A*	*D*···*A*	*D*—H···*A*
C23—H23*C*···Au1	0.98	2.83	3.664 (12)	144
C32—H32*B*···Se1	0.98	2.88	3.483 (13)	121
C11—H11*C*···I1^ii^	0.98	3.22	4.088 (13)	148
C21—H21*C*···I1^iii^	0.98	3.21	4.008 (12)	140
C22—H22*C*···I1^iii^	0.98	3.30	4.183 (12)	152
C31—H31*A*···I1^iv^	0.98	3.25	4.166 (11)	156
C31—H31*A*···Au1^iv^	0.98	3.19	3.642 (12)	110

Symmetry codes: (ii) −*x*+1/2, *y*−1/2, −*z*+3/4; (iii) −*x*+1/2, *y*+1/2, −*z*+3/4; (iv) −*y*+1, −*x*, −*z*+1/2.
